# Genome sequencing and analysis reveals possible determinants of *Staphylococcus aureus *nasal carriage

**DOI:** 10.1186/1471-2164-9-433

**Published:** 2008-09-22

**Authors:** Karthikeyan Sivaraman, Nitya Venkataraman, Jennifer Tsai, Scott Dewell, Alexander M Cole

**Affiliations:** 1 Department of Molecular Biology & Microbiology, Biomolecular Science Center, Burnett School of Biomedical Sciences, College of Medicine, University of Central Florida, Orlando, USA; 2 454 Life Sciences, Branford, USA

## Abstract

**Background:**

Nasal carriage of *Staphylococcus aureus *is a major risk factor in clinical and community settings due to the range of etiologies caused by the organism. We have identified unique immunological and ultrastructural properties associated with nasal carriage isolates denoting a role for bacterial factors in nasal carriage. However, despite extensive molecular level characterizations by several groups suggesting factors necessary for colonization on nasal epithelium, genetic determinants of nasal carriage are unknown. Herein, we have set a genomic foundation for unraveling the bacterial determinants of nasal carriage in *S. aureus.*

**Results:**

MLST analysis revealed no lineage specific differences between carrier and non-carrier strains suggesting a role for mobile genetic elements. We completely sequenced a model carrier isolate (D30) and a model non-carrier strain (930918-3) to identify differential gene content. Comparison revealed the presence of 84 genes unique to the carrier strain and strongly suggests a role for Type VII secretion systems in nasal carriage. These genes, along with a putative pathogenicity island (SaPIBov) present uniquely in the carrier strains are likely important in affecting carriage. Further, PCR-based genotyping of other clinical isolates for a specific subset of these 84 genes raise the possibility of nasal carriage being caused by multiple gene sets.

**Conclusion:**

Our data suggest that carriage is likely a heterogeneic phenotypic trait and implies a role for nucleotide level polymorphism in carriage. Complete genome level analyses of multiple carriage strains of *S. aureus *will be important in clarifying molecular determinants of *S. aureus *nasal carriage.

## Background

*Staphylococcus aureus *is a versatile pathogen capable of a wide spectrum of etiologies ranging from benign colonization of epithelia to fatal cases of septicemia. *S. aureus *has been identified as a global risk with the emergence of multi-drug resistant (MDR) strains. The majority of staphylococcal infections are nosocomial or community-acquired, and for both types, there is a strong correlation between staphylococcal infection and its colonization of the human nasal epithelium. Asymptomatic carriage of *S. aureus *in the anterior nasal vestibule occurs in approximately a quarter of the population to different degrees of severity [1], and can be either temporary or persist over many years. Nasal carriage of *S. aureus *has been identified as a risk factor in the clinical treatment of diabetic foot ulcers [2], post-operative recovery from heart surgery [3], and hemodialysis [4] amongst others.

Nasal carriage of *S. aureus *is multifactorial and likely involves both host and bacterial determinants [5-8]. One primary determinant of nasal carriage is the permissibility of host nasal fluid for bacterial growth. Indeed, a single strain of *S. aureus *(502-A) was shown to differentially colonize various hosts, underscoring the importance of host factors in nasal carriage [9]. Apart from host factors, several studies, including ours [1,10] and others [11,12], attribute a role for bacterial factors in carriage. It is notable that carrier strains, but not non-carrier strains, of *S. aureus *were able to persist and replicate within nasal fluids from carrier donors and on the surface of organotypic nasal epithelia [13], suggesting that carrier strains of *S. aureus *elaborate factors to aid in their nasal colonization.

Our group has extensively characterized two strains of *S. aureus*, one a clinical nasal carriage strain isolated from a persistent carrier (strain D30) [1,13,14] and another was isolated from a burn victim (strain 930918-3) [1,15-17]. Of these two strains, D30 was able to survive in the nasal fluid extracted from carrier host [1]. It was also shown to produce a capsular covering upon incubation with nasal fluid [1], which is likely a protective biofilm [14]. Contrarily, the strain 930918-3 was not capable of surviving in the nasal fluid of carriers and did not produce biofilms [1,14]. Most importantly, we revealed that D30 suppressed the innate immune response by downregulating TLR expression and TLR-mediated signaling in primary nasal epithelial cells while 930918-3 did not [13].

Several bacterial genes have been identified, which can potentially influence colonization on nasal epithelia. Notable amongst them are sortase A (srtA) [18,19], clumping factor B (clfB) [19-23] and tagX [24], which are involved in cell adhesion (srtA and clfB) and cell wall biosynthesis (tagX), respectively. In addition, studies implicating enterotoxins in *S. aureus *nasal carriage [11,12,25-28] show that enterotoxins were found in most but not all carrier strains. However, as we reveal herein, these collective genes may be necessary but not sufficient factors for nasal carriage. Taken together, these reports suggest that nasal carriage is a multifactorial process, although our knowledge of bacterial factors responsible for nasal carriage of *S. aureus *is still quite limited.

In this work, we have strived to bridge the genome level knowledge gap that exists in *S. aureus *nasal carriage. First, we assessed the lineage specific differences between carrier strains and non-carrier strains using multi-locus sequence typing (MLST) [29]. Subsequently, in order to determine the genome level differences between the model carrier strain and non carrier strain, we undertook a complete genome sequencing effort for our two highly characterized strains, D30 (carrier) and 930918-3 (non-carrier), and identified differential gene complements in both strains. Furthermore, for a specific subset of 76 genes that are non-fragmented and unique to either strain, we performed comparative PCR-based multi-locus genotyping. Notably, the results revealed the unique presence of genes in the carrier strain derived from bovine pathogenicity islands (SaPIBOV) [30,31], possible constituents of the type VII secretion system (T7SS) [32] and various other genes that are likely involved in pathogenesis. These studies provide a foundation for genome level analyses of *S. aureus *specific to human nasal carriage and will be instrumental in furthering our understanding of the carriage trait.

## Results and discussion

### MLST Analysis reveals no lineage specific differences between carrier and non-carrier strains of *S. aureus*

MLST analysis has been used as a powerful tool to identify lineage specific differences between various strains of a given bacterial species [33]. This technique has been applied extensively to *S. aureus *by several groups to characterize lineage specific differences in epidemiology [34-39] and infectivity [40-48]. We used MLST to identify possible differences between carrier and non-carrier strains of *S. aureus *(strain details provided in Table 1). For the test, we used the following strains D20, D30, D38, D85, D94, D98 and 930918-3. The strains whose names start with "D" are clinical nasal isolates while 930918-3 served as the non-carrier control. We also analyzed 9 completely sequenced strains of *S. aureus *for their allele types (Table 1 – **marked in italics**).

**Table 1:** Strains of *S. aureus *used in the study

Strain	Status	Notes/Accession No.
D20	Carrier	Clinical isolate/carrier strain
D30*	Carrier	Clinical Isolate/Model Carrier
D37	Carrier	Clinical isolate/carrier strain
D39	Carrier	Clinical isolate/carrier strain
D85	Carrier	Clinical isolate/carrier strain
D94	Carrier	Clinical isolate/carrier strain
D98	Carrier	Clinical isolate/carrier strain
930918-3*	non-carrier	Model Non-carrier
502A	opportunistic carrier	host dependent carriage persistence
		
N315	Not known	NC_002745
Mu50	Not known	NC_002758
COL	Not known	NC_002951
MRSA252	Not known	NC_002952
MSSA476	Not known	NC_002953
MW2	Not known	NC_003923
RF122	Not known	NC_007622
USA300	Not known	NC_007793
NCTC8325	Not known	NC_007795
JH9	Not known	NC_009487
JH1	Not known	NC_009632
Newman	Not known	NC_009641

* denotes strains sequenced in the study.

Italicized strain names denote completely sequenced strains whose carriage status is unknown.

**Figure 1:**
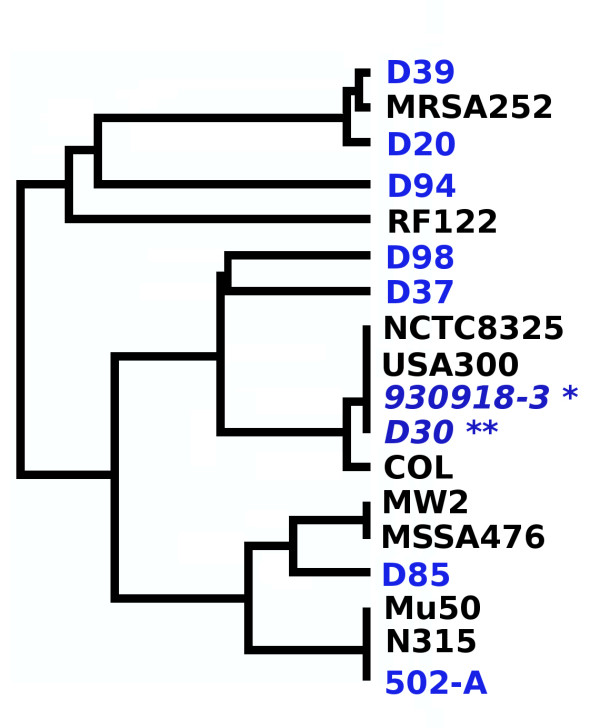
**MLST analysis of carrier and non-carrier strains reveal no lineage specific differences**. The dendrogram shows the various lineages of *S. aureus *tested by MLST. In all seven genes tested for MLST, D30 (double asterisk) and 930918-3 (single asterisk) were identical. Interestingly, strains D30 and 930918-3 belong to the same clonal complex as strain USA300 and NCTC8325. It can also be seen that MRSA252 is similar to the carrier strains D39 and D20. Strain 502-A, which is capable of experimentally induced intermittent nasal carriage, was seen to belong to the same clonal complex as strains Mu50 and N315 (found in the same clade in the dendrogram) and the carrier strain D94. Such varied clustering of carrier strains shows nasal carriage is not dependent on vertical lineage of the strains.

The dendrogram in Figure 1 presents the evolutionary relationship between all strains of *S. aureus*. It is readily apparent from the dendrogram that there is no lineage distinction between carrier strains and the non-carrier strain (930918-3). Importantly, our model carrier strain (D30) was on the same clade as was the model non-carrier strain (930918-3). These results raised the possibility that bacterial determinants of nasal carriage result from the variable genome content of *S. aureus*. It is important to note that the variable genome of *S. aureus *is not entirely made of mobile genetic elements and presence of a gene in the chromosomal backbone does not establish it as a core genome component. Thus, the determinants of nasal carriage, though necessarily a part of the variable genome, need not be restricted to those borne on mobile genetic elements.

### Genome sequencing and analysis of D30 and 930918-3 to identify differential gene content

MLST results revealed no lineage specific differences between the carrier and non-carrier strains. An analysis of *S. aureus *core and variable genomes revealed the presence of 1970 genes in all 12 previously sequenced genomes. The remaining ~700 genes were derived from the variable genome repertoire. Such a large number of variable genes, many of which are borne on mobile genetic elements, generates staggering diversity and variability in the *S. aureus *genome [10]. Such variability is compounded by the lack of carriage information for the completely sequenced strains and the lack of functional knowledge for most *S. aureus *genes. Due to these reasons, meaningful inference regarding the genetic determinants of nasal carriage from these strains is not possible.

Furthermore, genes that are reportedly necessary for nasal colonization [6-9,20] were, as we determined, in fact a part of the common minimal genome (CMG), implying an innate capability for any *S. aureus *strain to colonize nasal epithelium. Therefore, additional bacterial determinants are likely involved that can regulate the extent of nasal carriage. Indeed, we have experimentally shown that not all strains are equally capable of establishing carriage or survival in presence of nasal fluids [1]. Therefore, while certain genes necessary for nasal colonization have been functionally characterized, they may not be sufficient to establish carriage by themselves. To begin delineating genetic determinants of nasal carriage, we sequenced and compared the genomes of highly characterized carrier (D30) and non-carrier (930918-3) strain of *S. aureus*.

Pyrosequencing was utilized to sequence strains D30 and 930918-3 to 15× and 16× coverage, respectively. To identify the genes that were found in these two strains, we mapped the raw sequence reads to all the genes in the staphylococcal pan-genome. We defined the staphylococcal pan-genome as the non-redundant ORF set derived from all the Staphylococcal genomes sequenced at the time of this report (12 *S. aureus*, 2 *S. epidermidis*, 1 *S. haemolyticus*, and 1 *S. saphrophyticus*: described in methods and Table 1). For each orthologous set of genes identified by *B*i*D*irectional *B*est *H*it BLAST [49], one representative gene was retained. The final pan-genome for the genus consisted of 6122 ORFs (Additional file 1). We analyzed the whole staphylococcal pan-genome instead of the *S. aureus *pan-genome since an instance of horizontal gene transfer from *S. epidermidis *to *S. aureus *has been documented [50].

The mapping of raw reads to ORF's was a two-step process wherein we first removed ORFs that were not represented in the raw reads. This step reduced the probable number of ORFs from 6122 to less than 3600 (3359 in D30 and 3582 in 930918-3). In the second step, each gene that had matching raw sequence reads was analyzed individually for its coverage and assembly gaps in both genomes.

**Figure 2:**
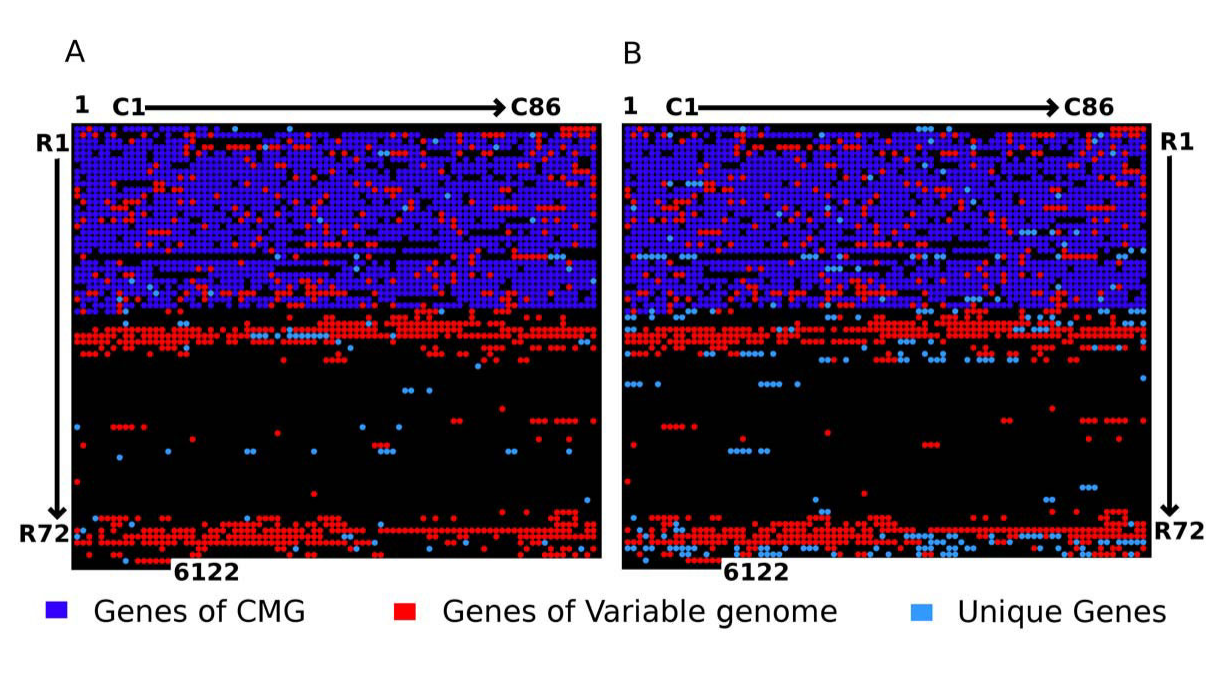
**Genome content of *S. aureus *strains D30 and 930918-3**. The figure illustrates the occurrence of genes in strains D30 (panel A) and strain 930918-3 (panel B). We also depict genes belonging to core, variable and unique gene complements of the two strains in their respective panels. The genes are represented in the array where the rows are named R1 through R72 and the columns are named C1 through C86. Each array element indexes a single gene. For example the index R1:C1, or simply (1:1) represents the first gene and (1:86) represents gene 86 and so forth. The number of genes in D30 and 930918-3 are calculated to be 2714 and 2879 respectively. The dark blue dots represent the core genome of *S. aureus*, the red dots represent the ORFs from the variable genome that are shared between the strains. The light blue dots represent ORFs unique to each strain with respect to the other and the black dots represent absence. A complete list of gene IDs (this study) and matrix positions are provided in Additional file 1, wherein the information is similarly color coded for the ease of identification.

All genes that could be assembled without gaps in the two genomes were considered to be present ("Tier 1"; definition of tiers is found in the *Methods *section). In total, 1952 genes in D30 and 2128 genes in 930918-3 belonged to this group. The remaining genes (~1500 in each genome) had varying degrees of gaps and coverage. For a gene with gaps, to be considered present in a given genome, the coverage threshold was set at 90%. Applying the threshold to "Tier 2" assemblies resulted in the selection of 751 genes in D30 and 746 genes in 930918-3. In a very small number of cases (8 genes in D30 and 5 genes in 930918-3) genes in "Tier 3" had coverage of 90% or greater. Owing to their high coverage, these Tier 3 genes were also considered present in their respective genomes. Table 2 summarizes the three tiers, the assembly and the gene coverage in the two strains. When the final gene content of Tiers 1–3 were tabulated for any coverage >90%, the genomes of D30 and 930918-3 contained 2715 and 2879 genes respectively (Table 2), of which 2631 genes were common to both strains. The overall gene content of the two strains depicted in Figure 2 suggests that the non-carrier strain 930918-3 has a wider variable repertoire than does the carrier strain D30.

**Table 2:** Overall ORF Content Statistics

Strain D30		Coverage(%)	
Group	100%	>90%	<= 90%
Tier1	1952	*	*
Tier2	*	754	343
Tier3	*	8	305
			
Total ORF	3355	Total ORF considered	2714

Strain 930918-3		Coverage(%)	
Group	100%	>90%	<= 90%

Tier1	2128	*	*
Tier2	*	746	355
Tier3	*	5	348
			
Total ORF	3582	Total ORF Considered	2879

Total number of ORFs considered in each strain is the sum of numbers represented in boldface for each tier.

**Figure 3:**
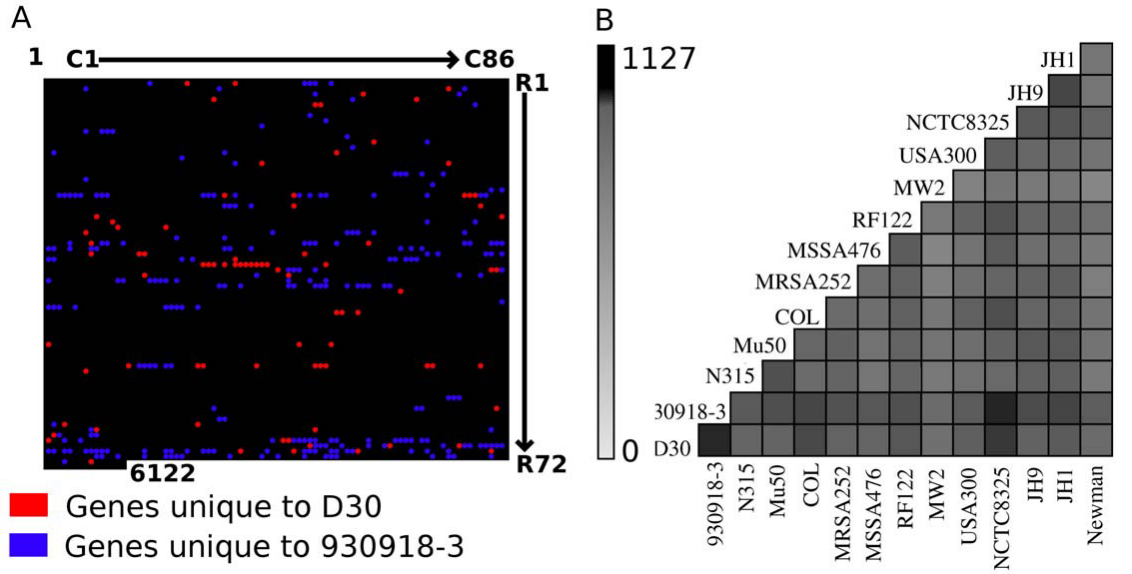
**A comparison and analysis of D30 and 930918-3 variable genome content**. In this figure, we illustrate the differential gene content of D30 and 930918-3 (Panel A) and compared the variable genome content of 1127 genes to all other completely sequenced *S. aureus *strains. Panel A depicts the differential gene content of D30 (red spots) and 930918-3 (blue spots) as mapped on to the staphylococcal pan-genome. The genes are represented in the array where the rows are named R1 through R72 and the columns are named C1 through C86 and each array element indexes a single gene (R#:C#). Panel B compares the combined variable gene content of strains D30 and 930918-3 to other 12 completely sequenced strains of *S. aureus*. The grayscale denotes the number of shared genes in the variable genome. The strain 930918-3 is the closest in variable gene content to D30, followed by strains NCTC8325 and USA300. This result is reminiscent of the MLST dendrogram (Figure 1) where these four strains were present on the same clade.

Once we had identified the genes for the each genome, we revised the CMG to include these two strains. Now with 14 sequenced genomes, we observed that the overall CMG content had fallen to 1792 genes from a previous estimate of 1970 genes. On the other hand, the combined non-redundant variable genome of these two strains (D30 and 930918-3) comprised of 1127 genes of which 835 were shared. Thus the genomes of D30 and 930918-3 differed from each other by 84 and 248 genes respectively (Figure 3A). As a next step, we used the combined variable genome set (1127 genes) to assess the relationship between these strains and all previously sequenced strains. The results shown in Figure 3B reiterate the trends observed in MLST dendrogram where D30 and 930918-3 are in the same clade as NCTC8325 and USA300 with COL being a neighbor in the tree. However, the strains D30 and 930918-3 were closest to each other in their variable genome content.

### Analysis of D30 unique gene content reveals potential effectors of nasal carriage

**Figure 4:**
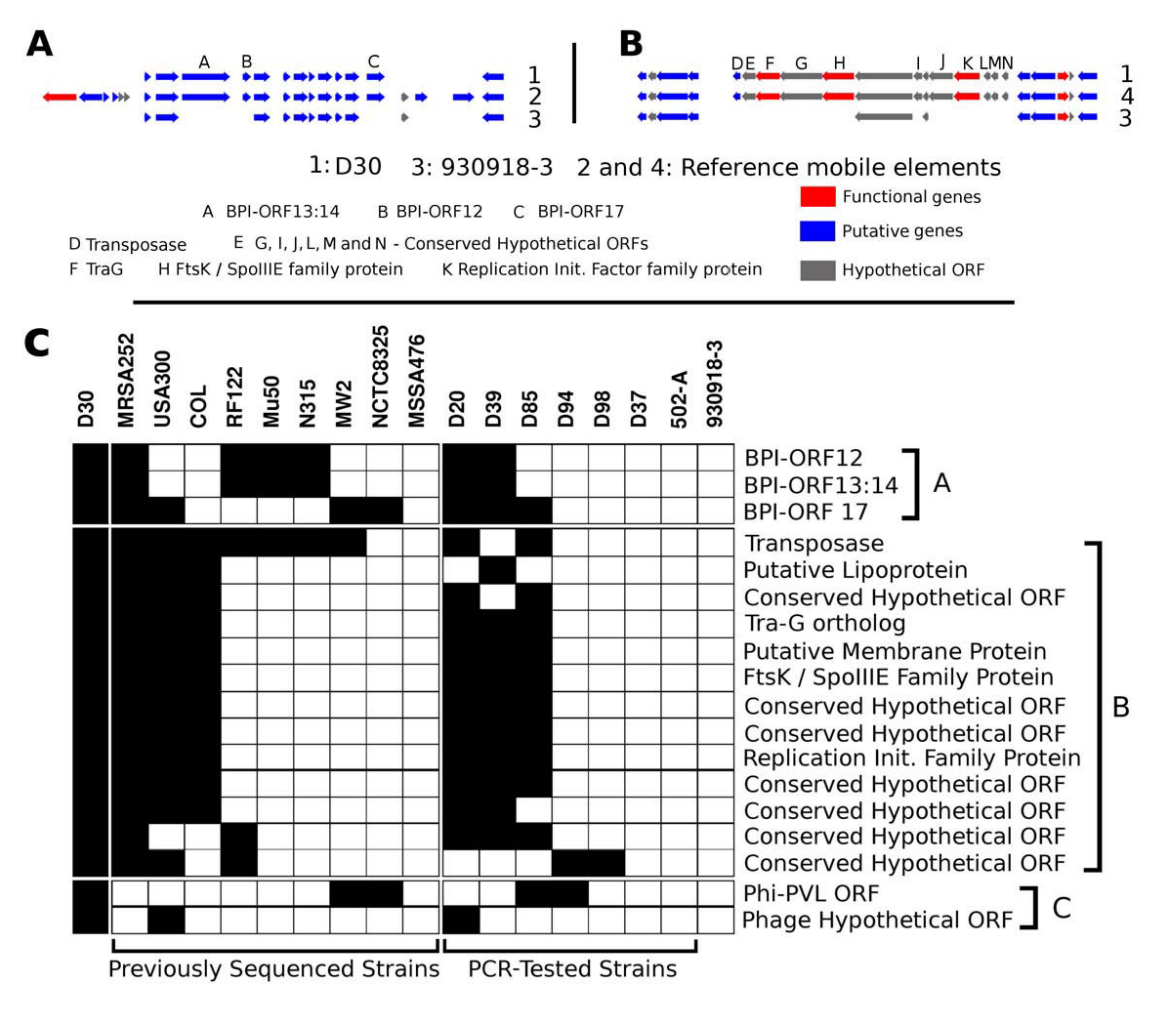
**PCR-based genotyping of carrier strains for 18 genes absolutely unique to D30 and *in-silico *comparison with other genomes**. Panel A represents the bovine pathogenicity island present in D30 and 930918-3 as compared to the complete BPI described. Polymorphism in BPI is common and it is apparent in the two strains. Panel B details the transposon that carries the putative T7SS in D30. Many genes of this transposon are absent in 930918-3. Panel C in the figure shows the presence (black) or absence (white) of the 18 genes that constitute the Unique Non-Fragmented (UNF) gene group in D30. The genes are divided based on the mobile element of origin. It can be seen majority of these genes are borne on a transposable element. Also the bovine pathogenicity element (SaPIBov) contributes to four genes in three reading frames where genes BPI13 and BPI14 are fused. It is interesting to note the placement of strains USA300 and NCTC8325 in this figure. USA300 is similar to D30 (14 of 18 genes present) while NCTC8325 has a profile very similar to 930918-3. This placement is in contrast with their overall proximity to each other (Figures 1 and 3B). Such disparity between overall proximity and carriage status stresses the importance of mobile genetic elements in nasal carriage.

There were 84 genes unique to the carrier strain D30 (Figure 3A red spots) out of which only 25 genes could be assigned functions based on their sequence similarity to genes in the non-redundant NCBI sequence database. We found 4 genes that were genetically linked to bovine pathogenicity island (SaPIBov ORF BPI12, BPI13:14 and BPI17) (Figure 4A). The genes BPI13:14 represent a fusion gene product in a single open reading frame. Further, there were two genes encoding superantigen-like proteins and a capsular polysaccharide synthesis enzyme Cap5P. Other genes included oligopeptide transporter protein and an ATP dependent proteinase, *tra*G and *Fts*K/*Spo*IIIE family protein, a phiSLT lysin amongst others. The remaining 59 genes comprised of either hypothetical open reading frames (30 genes) or transposons (29 genes). The functional genes are discussed in detail below with implications for nasal carriage. A roster of these genes along with their annotation is given in Additional file 2.

Bovine pathogenicity island (SaPIBov) was first observed in *S. aureus *isolates extracted from teats of milk cows. It was described as a mobile element capable of exhibiting high variation and was shown to encode several super antigens including toxic shock antigen (*tst*), and enterotoxin C (*sec*) and was shown to modulate the host immune response [30]. However, the roles of other SaPIBov genes, including BPI12, BPI13:14 and BPI17 are not known. Presence of these genes uniquely in the strain D30 raises the possibility of their involvement in nasal carriage. The difference in BPI between D30 and 930918-3 are illustrated in Figure 4A where the reference BPI map is adopted from citation [30].

Apart from the SaPIBov genes, the presence of TraG and FtsK/SpoIIIE in D30 is particularly interesting. In a recent review discussing the presence of Type VII secretion systems, Abdallah and coworkers [32] argue that FtsK/SpoIIIE family proteins are homologous to the type VII secretion systems (T7SS) in mycobacteria (the Esx system). An independent report [51] shows that a FtsK/SpoIIIE family member (*tra*J) and *tra*G product act as a bipartite translocation system in an *E. coli *plasmid. Further, studies by Burts and coworkers show that the Esx system is present in *S. aureus *[52] and is necessary for pathogenesis in the *S. aureus *Newman strain [53]. In both these reports, the authors mention the need for the FtsK/SpoIIIE family protein (FtsK/SpoIIIE Domain/FSD protein) for Esx effector translocation. These reports, in conjunction with our results, suggest a role in nasal carriage for protein translocation systems in general and for T7SS in particular. While the T7SS transporter system was found uniquely in D30, we did not find the known effector molecules in both strains. This is not to be construed as the presence of only a partial T7SS since the complete secretome associated with the T7SS in *S. aureus *is yet unknown and only a third of the genes in this pathogen have been assigned a function. The mobile genetic element that carries the *tra*G and FtsK/SpoIIIE genes is shown in Figure 4B.

Another notable pair of genes that were unique to D30 was the urease (alpha subunit – *ure*A) and *clp*L protease. Ureases are important survival factors in various pathogens including *Helicobacteri pylori *[54], *Staphylococcus saphrophyticus *[55], and *Proteus mirabilis *[56]. All these organisms use urease to withstand acidity or to enhance nitrogen utilization. Further, in a genome wide transcriptome analysis, Bore and colleagues [57] show that acid shock response is tightly linked to antioxidant mechanisms in *S. aureus *and the *ure*A gene shows considerable upregulation under acid stress.

While *ure*A is directly linked to acid response specifically, the *clp*L gene is involved at a global level in mediating stress responses in *S. aureus*. The *clp *family of genes is implicated in a variety of stress responses, including but not limited to biofilm formation and heat shock responses [58]. This comprehensive analysis of responses mediated by various *clp *members demonstrated the role of *clp*B and *clp*L in conferring induced thermotolerance. It is possible that these genes would allow the strain D30 to counter a myriad of host innate immune responses in the nasal epithelium.

Last in the list of putative nasal carriage modulators is the *phiSLT ORF484 like protein *(phiSLT lysin) gene. The phage phiSLT is known to be a helper phage for the pathogenic phage phi-PVL in *S. aureus *[59]. phiSLT is implicated in leukocytotoxicity and is known to lysogenize PVL phages and render them active. This phage carries a lytic locus that is similar to that of phi-PVL and may also be involved in pathogenesis directly. Presence of these genes in D30, and their absence in 930918-3, suggests a role for many or all of them in nasal carriage.

The strain 930918-3 was larger than the strain D30 and shared 2631 genes with the latter. Strain 930918-3 has 248 unique genes of which 125 (50%) were hypothetical ORFs. However, contrary to unique gene set of D30, the overall share of transposons was much less (15 of 248, 6%) and phages were the predominant mobile elements (65 of 248, 26.2%). Of the remaining 43 genes, most were metabolic enzymes (Additional file 1). There were also some pathogenesis related genes like the SaPI protein, SaPI integrase, phiPVL encoded proteins and Type I staphylococcal enterotoxin. The occurrence of these 248 genes in the pan-genome is illustrated in Figure 3A (blue spots).

The revised CMG, which incorporated the D30 and 930918-3 strains as well as the 12 previously sequenced strains of *S. aureus*, included 1792 genes. Although the size of the CMG was reduced by 178 genes once D30 and 930918-3 were added, genes necessary for globally mediating pathogenesis like the enterotoxins, TSST and the agr locus and many others were still a part of the CMG. Further, comparison of CMG genes lost in D30 and 930918-3 showed that they shared 169 of the 178 missing genes of which 129 belonged to the same **tier **in either genome with gaps in very similar loci. In the 40 genes that did belong to different tiers, 21 genes had the same coverage in both strains. Finally, only 4 of the remaining 19 genes had a higher coverage (>1% difference in coverage) in D30 as compared to 930918-3. All four of these genes were hypothetical ORFs whose functions are yet unknown. Thus, the drastic reduction in CMG while stringent is unlikely to affect the current interpretation of the results.

As a next step, we stringently filtered the unique gene set to identify a specific subset of genes unique to D30, and tested their distribution in 7 other strains capable of nasal carriage.

### Evidence for existence of multiple gene sets determining nasal carriage: PCR-based multilocus genotyping of carrier strains

There were 84 intact genes that were unique to the carrier strain, but fragments of a majority of these genes (66 of 84) were found in the strain 930918-3. Similarly, there were 190 intact genes in 930918-3 whose fragments were found in D30. Most of these genes were borne on mobile genetic elements and hence this observation was not completely unexpected. However, a set of 18 genes in D30 and 58 in 930918-3, which had no fragments or vestiges in the other genome was termed the *Unique Non-Fragmented *(UNF) genes. The list of the 18 UNF genes in D30 is given in Figure 4C while the complete list of UNF genes in both genomes is provided in Additional file 2. We analyzed the UNF set by PCR in six other carrier isolates and one strain (502-A) that when experimentally applied to the nose can intermittently colonize the nasal epithelium in certain individuals [9]. The result of the PCR-based genetic profiling of these 18 genes is summarized in Figure 4C.

Of the seven tested carrier strains, four (D30, D20, D39 and D85) strains contained a majority these 18 genes. The closest was D20, which contained 15 of the 18 genes while D85 and D39 contained 12 and 13 of the 18 genes respectively. Of particular importance was the presence of the transposon carrying traG and FtsK/SpoIIIE proteins, which are likely a part of the T7SS in *S. aureus*. Presence of these genes in 4 carrier strains reinforces the possible role for T7SS in nasal carriage. Further, presence of most of these genes in strains MRSA252 and USA300 may imply a carriage status for these strains. Statistical tests based on randomly generated genomes (in-silico) of *S. aureus *show that the presence of these genes in D30, D20, D39 and D85 is highly significant (P-value = 0.0 after Bonferroni correction).

On the other hand, strains D37, D94 and D98 contained very few (2 genes of 18 in D94 and D98) or none of the 18 genes (D37). A similar trend was seen for the intermittent carrier strain 502-A (0 of 18). Absence of the *tra*G/FtsK/SpoIIIE system in these strains might denote the existence of more than one set of genes responsible for nasal carriage, or may signify that this system is required for *persistent *carriage in certain host types. Overall, our results raise the possibility that nasal carriage is brought about by more than one set of genes. Further, it is interesting that the strains 930918-3 and NCTC8325 share a similar profile in this clustering (0 of 18). These two strains, along with strain USA300 belonged to the same allotype as strain D30 (Figure 1). This observation reinforces our hypothesis that genetic determinants of nasal carriage are borne on mobile elements. The presence of these genes in 4 of the 7 carrier strains along with the functionality reported for the transporter complex [32,52,53] argues against random inclusion of these genes in carrier strains, and reinforces their role in nasal carriage.

Apart from being contributed to by whole genes and sets of genes, nasal carriage may also depend on other factors in the genome. Foremost of these is the presence of nucleotide level polymorphisms in the genome. Genes that play a role in nasal colonization of *S. aureus *like clumping factors [19,23] and *tag*X [24] also accumulate a high proportion of non-synonymous mutations (KS and AMC, manuscript in preparation). Such variation in these genes could possibly affect the carriage capabilities of a given strain. Additionally, nasal carriage involves a dynamic interplay between the host and the bacterium and as several reports indicate, there are host components involved. A comprehensive understanding of nasal carriage would require that these collective factors be addressed in context.

## Conclusion

We have completely sequenced and compared the genomes of a highly characterized clinical nasal isolate of *S. aureus *(D30) and a non-carrier *S. aureus *strain (930918-3). Comparison of ORF content of these two strains revealed the presence of several genes in D30 that might be critical determinants of nasal carriage. The presence of FtsK/SpoIIIE family member and an associated protein TraG uniquely in D30 implies a role for Type VII secretion systems in *S. aureus *nasal carriage. While our work suggests a possible role for a Type VII Secretion System in nasal carriage, a complete genomic analysis of a large number of carrier strains, properly stratified from both intermittent carriers and persistent carriers, will be necessary to confirm this and elucidate other bacterial determinants of *S. aureus *nasal carriage.

## Methods

### Bacterial Strains used in this study

*S. aureus *strains D20, D30, D37, D39, D85, D94, D98, 930918-3 are described in previous studies [1,13-17]. Apart from these strains, we also used an opportunistic carrier strain (502-A) [60] in our analyses. 502-A has been shown to be capable of carriage in certain hosts [9] in both persistent and intermittent carriers. All strains were propagated on Trypticase Soy Agar (TSA) plates and liquid cultures in Trypticase Soy Broth (TSB). D30 and 930918-3 have been confirmed as being *S. aureus *by using *S. aureus *specific 16S-rRNA primers [61] and others by using a Staphyloslide kit (BD and Co., MD, USA) that has been previously validated [1].

### MLST analysis of *S. aureus *strains

Primers reported by Enright and colleagues [29] were obtained from Invitrogen (Carlsbad, CA) and PCR performed using conditions reported therein. The amplicons were cloned into TOPO-4 (Invitrogen, Carlsbad, CA) and transformed into chemically competent Top10 *E. coli *cells. Transformants were selected on LB-Agar plates with 75 μg/mL Ampicillin and cultures of transformants grown overnight in LB broth with 75 μg/mL ampicillin at 37°C, shaken at 270 rpm. Plasmids were extracted from the overnight cultures using a QuickLyse kit (Qiagen, Valencia, CA) as per the manufacturer's instructions, and the extracted plasmid was assessed for quantity and purity by O.D. measurements using a SpectraMax spectrophotometer (Molecular Systems, Sunnyvale, CA). These plasmids were then sequenced by the dye-terminator Sanger double barrel sequencing method using standard T3 and T7-Forward primers, and the sequence was used to identify the allotype by querying the MLST database .

### Genomic DNA extraction from *S. aureus *strains

*S. aureus *was plated on to TSA plates and incubated overnight at 37°C. Single colonies were subsequently inoculated in 3 ml TSB and grown overnight at 37°C, diluted 1:200 in TSB, and incubated an additional 3 hrs to obtain cells in log phase growth. Cells were sedimented and genomic DNA was extracted using Genomic Tip kits (Qiagen, Valencia, CA). Lysostaphin (Sigma-Aldrich, St. Louis, MO) from *S. staphylolyticus *was used to lyse the cell wall of *S. aureus*. Genomic DNA from *S. aureus *strains D30 and 930918-3 were extracted and resuspended in Tris-EDTA (TE) to a final concentration of 382 μg/ml and 500 μg/ml respectively. Prior to genomic sequencing, the two strains were confirmed as being *S. aureus *by using species-specific 16S-rRNA primers [61].

### Genome Sequencing, Assembly and Sequence Analysis

Genomic DNA from *S. aureus *strains D30 and 930918-3 were subjected to pyrosequencing [62]. De novo sequence assembly of the sequence reads into large contigs (>500 bases) was performed using Newbler. We created a non-redundant set of ORFs representing the staphylococcal pan-genome using bidirectional best hit. For this, each of the available *Staphylococcus *ORFs (12 *S. aureus *genomes, two *S. epidermidis *genomes, and one *S. haemolyticus *genomes [50,63-68] – details given in Table 1) were taken in chronological order and orthologs were identified in the remaining set using BLAST [49]. While the ORF itself was retained, the orthologs were removed from further consideration. We considered all the Staphylococcal plasmids that have been sequenced as separate genetic entities and analyzed them similarly. The staphylococcal pan-genome was then used to identify the core and variable genome constituents of the sequenced strains D30 and 930918-3. Further, the contig assemblies were subject to gene prediction using Glimmer trained on the staphylococcal pan-genome. These contigs were submitted to NCBI (ABFA00000000 and ABFB00000000 for D30 and 930918-3 respectively). The statistics of contig assembly are provided in Table 3.

**Table 3:** Genome sequencing and contig assembly statistics.

Parameters	D30	930918-3
Total No. of Reads	818689	862963
No. of Assembled Reads	802249	839907
No. of Large Contigs	75	139
Total No. of Bases	2727791	2838563
Average Contig size (bases)	36370	20421
N_50 _Contig size (bases)	73183	42932
Largest Contig size (bases)	175032	148157

**Figure 5:**
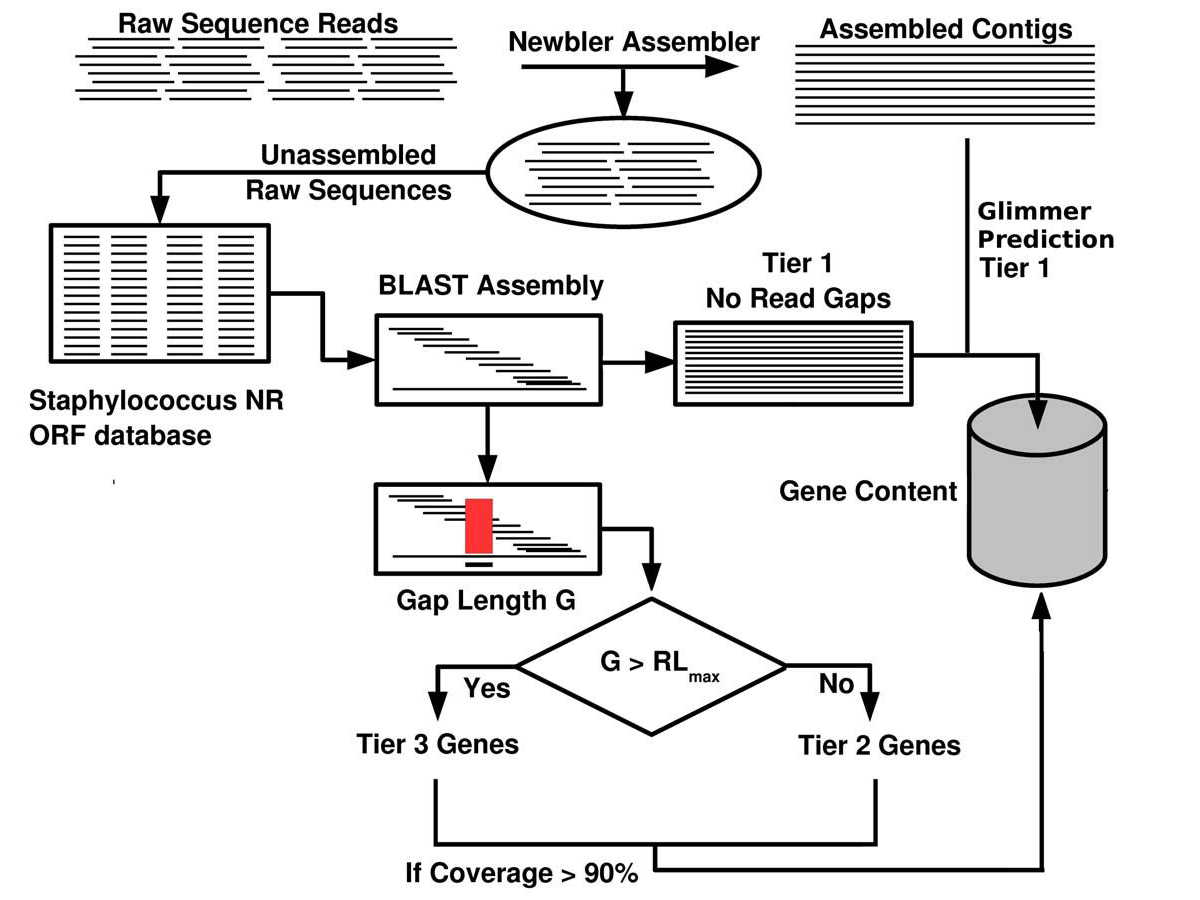
**Illustration of the method used for gene identification in *S. aureus *D30 and *S. aureus *930918-3**. The raw sequences were assembled into contigs using Newbler with a contig size threshold of 500 bases. The raw reads that were not assembled were then aligned against individual matching genes and the assemblies were tested for completeness. Those genes that were covered over at least 90% of their length were considered present. The sum total of genes thus identified in each genome was subjected to comparative genome analysis.

Figure 5 illustrates the method we adopted to identify ORFs. In brief, each ORF in the pan-genome was used to comparatively map and assemble matching raw sequence reads (using BLAST [49]) and in the first iteration, all the ORFs that could be tested affirmatively for consistency (no in-frame stop codons in at least one frame) and completeness (no gaps/read gaps) were pooled and designated "Tier 1". Further analysis included only those genes that were a part of the *S. aureus *common minimal genome (CMG). The CMG was derived from the pan-genome and is defined as the set of genes that are present in all *S. aureus *strains. For those CMG genes that were not present in Tier 1 (due to read gaps), we identified those with small (gap < maximum read length) and large gaps (gap > maximum read length). The former group was designated "Tier 2" while the latter was designated "Tier 3" (Table 2). Those ORFs, which had at least 90% coverage were included in comparative analysis studies.

In order to define the set of Unique Non-Fragmented (UNF) genes, we used subtractive BLAST analysis on the raw sequence data sets and removed all matching sequences from both genomes. The remaining sequence reads were then searched using the staphylococcal pan-genome and genes were identified.

### PCR for analyzing differential gene complement in carrier strains

All genes that were identified by sequence analysis to be a part of the UNF between strains D30 and 930918-3 were confirmed by PCR analysis. The primers and conditions for each gene are given in Additional file 3. The primers were designed using Primer-3 software at the MIT web server  and PCR was performed for the 76 unique genes using 10 different strains of *S. aureus *(D20, D20-2006, D30, D37, D39, D85, D94, D98, 930918-3 and 502-A). The amplicons were subject to electrophoresis in a 2% agarose gel in Tris-Borate EDTA buffer and visualized using Ethidium bromide stain in a Bio-Rad gel documentation system (Hercules, CA).

### Statistical analysis to test significance of PCR genotyping

We created 1000 random *S. aureus *genomes using the core-genome (1792 genes) and 850 genes (average variable genome size) derived from the variable repertoire. Using these random genomes, we calculated the probability of accumulating all the 18 UNF genes within a single strain. All the raw probabilities were adjusted and Bonferroni correction applied to estimate the P-values. All statistical analyses were performed using R package on a Linux platform.

## Authors' contributions

SK and AMC designed the study. SK and NV performed MLST analysis. SK, JT and SD performed the contig assembly. SK created the sequence analysis pipeline, performed sequence analysis and completed comparative genome analysis. SK and AMC wrote the manuscript. All authors have read and approved the final manuscript.

## Supplementary Material

Additional file 1 **The file contains information of genes analyzed in this study.** This file also shows the indexing of spots shown in figures 2 and 3A in the text. The color codes used in this file are similar to those used in the figure legends. Blue represents the common minimal genome, red the shared variable genes, light blue the unique genes and black represents ORFs that are absent. The file also contains NCBI accession for each of the 6122 ORFs and NRID used specifically in this study to identify ORFs. Click here for file

Additional file 2 **The file lists unique genes as assessed by this study in both D30(Table **1**in Additional file) and 930918-3(Table**2**in Additional file)**. These genes are cross referenced by a) their NCBI accession numbers and b) project specific NRID and annotations where applicable are provided. Genes marked in turquoise blue belong to UNF set. Click here for file

Additional file 3 The file lists the primers used in this study for analyzing the distribution of UNF genes in strains D30 and 930918-3. Also provided are conditions of PCR including the annealing temperature for each primer pair. Click here for file
