# Supplementary material for: Genome sequencing and analysis reveals possible determinants of Staphylococcus aureus nasal carriage
Source: BMC Genomics. 2008 Sep 22;9:433. doi: 10.1186/1471-2164-9-433 (PMC2566312; doi:10.1186/1471-2164-9-433)
Supplement: Additional file 1 — The file contains information of genes analyzed in this study. This file also shows the indexing of spots shown in figures 2 and 3A in the text. The color codes used in this file are similar to those used in the figure legends. Blue represents the common minimal genome, red the shared variable genes, light blue the unique genes and black represents ORFs that are absent. The file also contains NCBI accession for each of the 6122 ORFs and NRID used specifically in this study to identify ORFs. [file 1471-2164-9-433-S1.pdf]

***Supplementary information 1**: A non-redundant list of orthologous genes in*

Staphylococcus aureus genomes. The row and column denote the row and column i

figure 2 and figure 3A in main text. There are 72 rows and 86 columns representinç

a total of 6122 open reading frames in S. aureus pan-genome.

The NRID was assigned in this study as method of referring to the genes

The accession numbers are those assigned by genbank to the respective gene

The color codes for ORFs match those used in the figures. Blue represents the

common minimal genome, red the shared variable genes, light blue the unique gene

and black represents genes that are absent.

Row	Column	NRID	Accession Information	D30	930918-3
1	1	nr0001	ref|NC_002745.2|:517-1878		
1	2	nr0002	ref|NC_002745.2|:2156-3289		
1	3	nr0003	ref|NC_002745.2|:3670-3915		
1	4	nr0004	ref|NC_002745.2|:3912-5024		
1	5	nr0005	ref|NC_002745.2|:5034-6968		
1	6	nr0006	ref|NC_002745.2|:7005-9674		
1	7	nr0007	ref|NC_002745.2|:c10592-9762		
1	8	nr0008	ref|NC_002745.2|:10879-12414		
1	9	nr0009	ref|NC_002745.2|:12793-14079		
1	10	nr0010	ref|NC_002745.2|:14730-15425		
1	11	nr0011	ref|NC_002745.2|:15422-15751		
1	12	nr0012	ref|NC_002745.2|:16093-17082		
1	13	nr0013	ref|NC_002745.2|:17318-18313		
1	14	nr0014	ref|NC_002745.2|:18328-20295		
1	15	nr0015	ref|NC_002745.2|:20292-20738		
1	16	nr0016	ref|NC_002745.2|:20770-22170		
1	17	nr0017	ref|NC_002745.2|:22448-23731		
1	18	nr0018	ref|NC_002745.2|:24928-25635		
1	19	nr0019	ref|NC_002745.2|:25648-27474		
1	20	nr0020	ref|NC_002745.2|:27440-28801		
1	21	nr0021	ref|NC_002745.2|:28802-29590		
1	22	nr0022	ref|NC_002745.2|:29979-30779		
1	23	nr0023	ref|NC_002745.2|:31006-33324		
1	24	nr0024	ref|NC_002745.2|:33692-34171		
1	25	nr0025	ref|NC_002745.2|:34454-35749		
1	26	nr0026	ref|NC_002745.2|:36164-36403		

1	27	nr0027	ref|NC_002745.2|:c37109-36435
1	28	nr0028	ref|NC_002745.2|:c37406-37200
1	29	nr0029	ref|NC_002745.2|:c38204-37410
1	30	nr0030	ref|NC_002745.2|:c38309-38229
1	31	nr0031	ref|NC_002745.2|:c39690-38428
1	32	nr0032	ref|NC_002745.2|:39799-39939
1	33	nr0033	ref|NC_002745.2|:39987-40178
1	34	nr0034	ref|NC_002745.2|:c40601-40197
1	35	nr0035	ref|NC_002745.2|:c41588-40818
1	36	nr0036	ref|NC_002745.2|:c42455-41781
1	37	nr0037	ref|NC_002745.2|:42539-42880
1	38	nr0038	ref|NC_002745.2|:43717-44460
1	39	nr0039	ref|NC_002745.2|:44557-44985
1	40	nr0040	ref|NC_002745.2|:c47037-45031
1	41	nr0041	ref|NC_002745.2|:47137-48894
1	42	nr0042	ref|NC_002745.2|:48894-49265
1	43	nr0043	ref|NC_002745.2|:49752-50882
1	44	nr0044	ref|NC_002745.2|:c51388-50996
1	45	nr0045	ref|NC_002745.2|:c52330-51392
1	46	nr0046	ref|NC_002745.2|:c53427-52363
1	47	nr0047	ref|NC_002745.2|:53563-53823
1	48	nr0048	ref|NC_002745.2|:53823-54467
1	49	nr0049	ref|NC_002745.2|:c55474-54806
1	50	nr0050	ref|NC_002745.2|:56002-56733
1	51	nr0051	ref|NC_002745.2|:c57641-56859
1	52	nr0052	ref|NC_002745.2|:c58169-57792
1	53	nr0053	ref|NC_002745.2|:c60068-58176
1	54	nr0054	ref|NC_002745.2|:c61150-60065
1	55	nr0055	ref|NC_002745.2|:c61586-61269
1	56	nr0056	ref|NC_002745.2|:c62125-61607
1	57	nr0057	ref|NC_002745.2|:c62442-62131
1	58	nr0058	ref|NC_002745.2|:c62879-62529
1	59	nr0059	ref|NC_002745.2|:c65025-63397
1	60	nr0060	ref|NC_002745.2|:c66396-65047
1	61	nr0061	ref|NC_002745.2|:c68423-66630
1	62	nr0062	ref|NC_002745.2|:c68719-68423
1	63	nr0063	ref|NC_002745.2|:c69958-68912
1	64	nr0064	ref|NC_002745.2|:c70586-70209
1	65	nr0065	ref|NC_002745.2|:c71051-70698
1	66	nr0066	ref|NC_002745.2|:c71350-71036
1	67	nr0067	ref|NC_002745.2|:c72445-71861
1	68	nr0068	ref|NC_002745.2|:c74204-73509
1	69	nr0069	ref|NC_002745.2|:c76812-74179
1	70	nr0070	ref|NC_002745.2|:77116-78792
1	71	nr0071	ref|NC_002745.2|:78811-80832
1	72	nr0072	ref|NC_002745.2|:80880-81251
1	73	nr0073	ref|NC_002745.2|:c82819-81950
1	74	nr0074	ref|NC_002745.2|:c83731-82829
1	75	nr0075	ref|NC_002745.2|:83767-83892

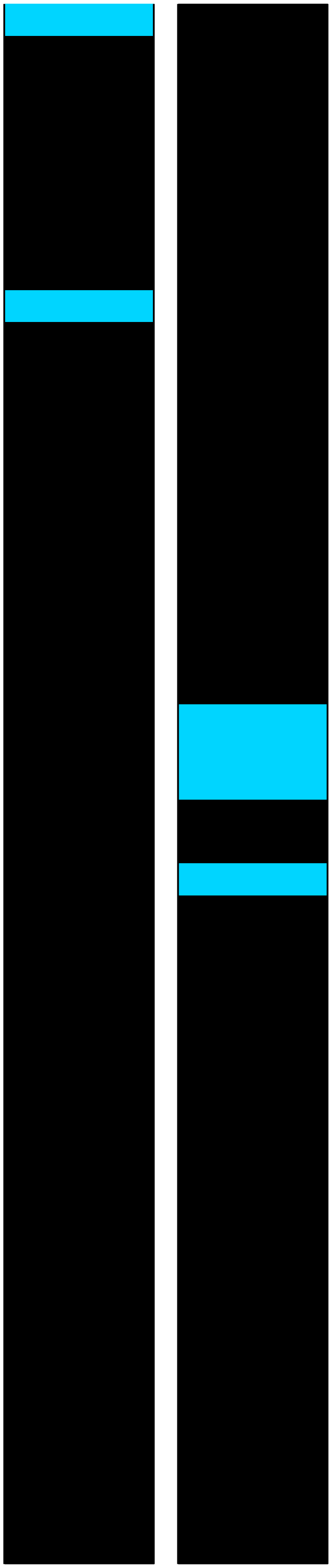

1	76	nr0076	ref|NC_002745.2|:c84404-83925
1	77	nr0077	ref|NC_002745.2|:c84799-84467
1	78	nr0078	ref|NC_002745.2|:c85470-84844
1	79	nr0079	ref|NC_002745.2|:85585-87093
1	80	nr0080	ref|NC_002745.2|:87692-89425
1	81	nr0081	ref|NC_002745.2|:c89947-89609
1	82	nr0082	ref|NC_002745.2|:c91124-90369
1	83	nr0083	ref|NC_002745.2|:c91384-91124
1	84	nr0084	ref|NC_002745.2|:91521-92585
1	85	nr0085	ref|NC_002745.2|:92616-93950
1	86	nr0086	ref|NC_002745.2|:93969-95162
2	1	nr0087	ref|NC_002745.2|:c96815-95829
2	2	nr0088	ref|NC_002745.2|:97124-97327
2	3	nr0089	ref|NC_002745.2|:97376-97672
2	4	nr0090	ref|NC_002745.2|:97871-98482
2	5	nr0091	ref|NC_002745.2|:98749-101901
2	6	nr0092	ref|NC_002745.2|:101958-102440
2	7	nr0093	ref|NC_002745.2|:102646-103632
2	8	nr0094	ref|NC_002745.2|:103853-104623
2	9	nr0095	ref|NC_002745.2|:104675-105445
2	10	nr0096	ref|NC_002745.2|:105513-106280
2	11	nr0097	ref|NC_002745.2|:106345-107115
2	12	nr0098	ref|NC_002745.2|:107182-107949
2	13	nr0099	ref|NC_002745.2|:108046-110283
2	14	nr0100	ref|NC_002745.2|:110434-111612
2	15	nr0101	ref|NC_002745.2|:111614-113002
2	16	nr0102	ref|NC_002745.2|:c115153-113486
2	17	nr0103	ref|NC_002745.2|:c115415-115245
2	18	nr0104	ref|NC_002745.2|:c117247-115472
2	19	nr0105	ref|NC_002745.2|:c118293-117421
2	20	nr0106	ref|NC_002745.2|:118475-119803
2	21	nr0107	ref|NC_002745.2|:119993-120466
2	22	nr0108	ref|NC_002745.2|:120728-122320
2	23	nr0109	ref|NC_002745.2|:c124001-122649
2	24	nr0110	ref|NC_002745.2|:c125174-124422
2	25	nr0111	ref|NC_002745.2|:c126460-125543
2	26	nr0112	ref|NC_002745.2|:c127533-126538
2	27	nr0113	ref|NC_002745.2|:c128541-127549
2	28	nr0114	ref|NC_002745.2|:128772-129752
2	29	nr0115	ref|NC_002745.2|:129749-130759
2	30	nr0116	ref|NC_002745.2|:130780-132534
2	31	nr0117	ref|NC_002745.2|:132527-133783
2	32	nr0118	ref|NC_002745.2|:133773-135509
2	33	nr0119	ref|NC_002745.2|:135490-137268
2	34	nr0120	ref|NC_002745.2|:137240-138019
2	35	nr0121	ref|NC_002745.2|:138019-139221
2	36	nr0122	ref|NC_002745.2|:139225-139989
2	37	nr0123	ref|NC_002745.2|:140185-140811
2	38	nr0124	ref|NC_002745.2|:141023-141799

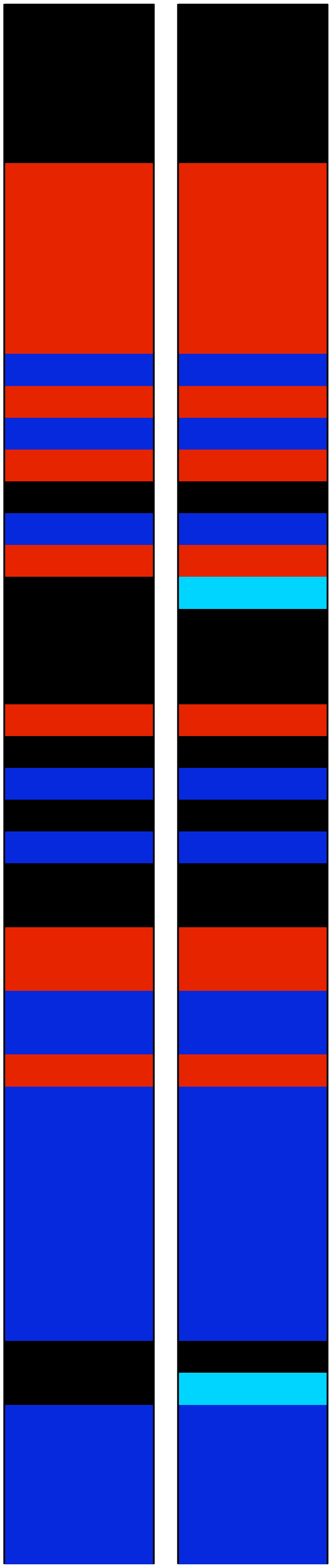

2	39	nr0125	ref|NC_002745.2|:c142000-141848
2	40	nr0126	ref|NC_002745.2|:142144-143115
2	41	nr0127	ref|NC_002745.2|:143078-143770
2	42	nr0128	ref|NC_002745.2|:143980-145146
2	43	nr0129	ref|NC_002745.2|:145127-146365
2	44	nr0130	ref|NC_002745.2|:146355-147785
2	45	nr0131	ref|NC_002745.2|:148053-148652
2	46	nr0132	ref|NC_002745.2|:149019-149744
2	47	nr0133	ref|NC_002745.2|:c150675-149935
2	48	nr0134	ref|NC_002745.2|:150926-151633
2	49	nr0135	ref|NC_002745.2|:151640-152992
2	50	nr0136	ref|NC_002745.2|:153073-153735
2	51	nr0137	ref|NC_002745.2|:153763-154941
2	52	nr0138	ref|NC_002745.2|:c155885-155070
2	53	nr0139	ref|NC_002745.2|:c156682-155882
2	54	nr0140	ref|NC_002745.2|:c157457-156684
2	55	nr0141	ref|NC_002745.2|:c158608-157652
2	56	nr0142	ref|NC_002745.2|:158837-160381
2	57	nr0143	ref|NC_002745.2|:160432-161967
2	58	nr0144	ref|NC_002745.2|:162124-162891
2	59	nr0145	ref|NC_002745.2|:162897-164072
2	60	nr0146	ref|NC_002745.2|:164457-167066
2	61	nr0147	ref|NC_002745.2|:167409-168077
2	62	nr0148	ref|NC_002745.2|:168093-168779
2	63	nr0149	ref|NC_002745.2|:168782-169546
2	64	nr0150	ref|NC_002745.2|:169566-171389
2	65	nr0151	ref|NC_002745.2|:171379-172407
2	66	nr0152	ref|NC_002745.2|:172420-173529
2	67	nr0153	ref|NC_002745.2|:173533-174657
2	68	nr0154	ref|NC_002745.2|:174660-175286
2	69	nr0155	ref|NC_002745.2|:175291-176400
2	70	nr0156	ref|NC_002745.2|:176414-177580
2	71	nr0157	ref|NC_002745.2|:177573-178778
2	72	nr0158	ref|NC_002745.2|:178779-179984
2	73	nr0159	ref|NC_002745.2|:179995-180552
2	74	nr0160	ref|NC_002745.2|:180552-181439
2	75	nr0161	ref|NC_002745.2|:181493-182755
2	76	nr0162	ref|NC_002745.2|:182802-183977
2	77	nr0163	ref|NC_002745.2|:c184368-184042
2	78	nr0164	ref|NC_002745.2|:c184758-184375
2	79	nr0165	ref|NC_002745.2|:185185-186672
2	80	nr0166	ref|NC_002745.2|:187319-188278
2	81	nr0167	ref|NC_002745.2|:c188543-188388
2	82	nr0168	ref|NC_002745.2|:c188776-188573
2	83	nr0169	ref|NC_002745.2|:188955-189467
2	84	nr0170	ref|NC_002745.2|:189809-190549
2	85	nr0171	ref|NC_002745.2|:190563-191534
2	86	nr0172	ref|NC_002745.2|:191531-192292
3	1	nr0173	ref|NC_002745.2|:192305-193336

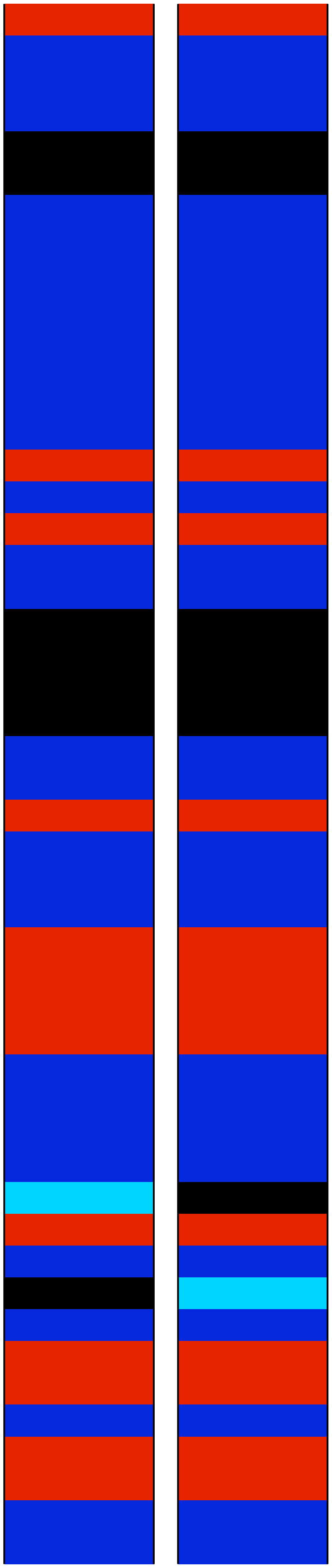

3	2	nr0174	ref|NC_002745.2|:193562-193912
3	3	nr0175	ref|NC_002745.2|:193987-195111
3	4	nr0176	ref|NC_002745.2|:195497-196747
3	5	nr0177	ref|NC_002745.2|:197194-204369
3	6	nr0178	ref|NC_002745.2|:204382-205026
3	7	nr0179	ref|NC_002745.2|:c205848-205354
3	8	nr0180	ref|NC_002745.2|:c206867-206103
3	9	nr0181	ref|NC_002745.2|:c208124-206883
3	10	nr0182	ref|NC_002745.2|:c209167-208136
3	11	nr0183	ref|NC_002745.2|:c210390-209206
3	12	nr0184	ref|NC_002745.2|:c211998-210649
3	13	nr0185	ref|NC_002745.2|:c212774-212277
3	14	nr0186	ref|NC_002745.2|:c214541-212901
3	15	nr0187	ref|NC_002745.2|:c216859-214814
3	16	nr0188	ref|NC_002745.2|:217444-218499
3	17	nr0189	ref|NC_002745.2|:218496-219395
3	18	nr0190	ref|NC_002745.2|:219407-220861
3	19	nr0191	ref|NC_002745.2|:220864-221739
3	20	nr0192	ref|NC_002745.2|:c222214-221933
3	21	nr0193	ref|NC_002745.2|:222427-225216
3	22	nr0194	ref|NC_002745.2|:c225962-225363
3	23	nr0195	ref|NC_002745.2|:226088-227200
3	24	nr0196	ref|NC_002745.2|:227197-227877
3	25	nr0197	ref|NC_002745.2|:227874-229052
3	26	nr0198	ref|NC_002745.2|:229259-229480
3	27	nr0199	ref|NC_002745.2|:229518-231236
3	28	nr0200	ref|NC_002745.2|:231295-231738
3	29	nr0201	ref|NC_002745.2|:231750-232472
3	30	nr0202	ref|NC_002745.2|:232459-232635
3	31	nr0203	ref|NC_002745.2|:c234937-233345
3	32	nr0204	ref|NC_002745.2|:235035-236372
3	33	nr0205	ref|NC_002745.2|:236378-237541
3	34	nr0206	ref|NC_002745.2|:237558-239333
3	35	nr0207	ref|NC_002745.2|:239371-241377
3	36	nr0208	ref|NC_002745.2|:c242463-241690
3	37	nr0209	ref|NC_002745.2|:c243278-242652
3	38	nr0210	ref|NC_002745.2|:243487-244065
3	39	nr0211	ref|NC_002745.2|:244449-245546
3	40	nr0212	ref|NC_002745.2|:245559-246830
3	41	nr0213	ref|NC_002745.2|:246833-248101
3	42	nr0214	ref|NC_002745.2|:248103-248942
3	43	nr0215	ref|NC_002745.2|:249013-250092
3	44	nr0216	ref|NC_002745.2|:250117-251157
3	45	nr0217	ref|NC_002745.2|:251212-252180
3	46	nr0218	ref|NC_002745.2|:c253035-252541
3	47	nr0219	ref|NC_002745.2|:253268-254647
3	48	nr0220	ref|NC_002745.2|:c255764-255006
3	49	nr0221	ref|NC_002745.2|:c257313-255757
3	50	nr0222	ref|NC_002745.2|:c258278-257310

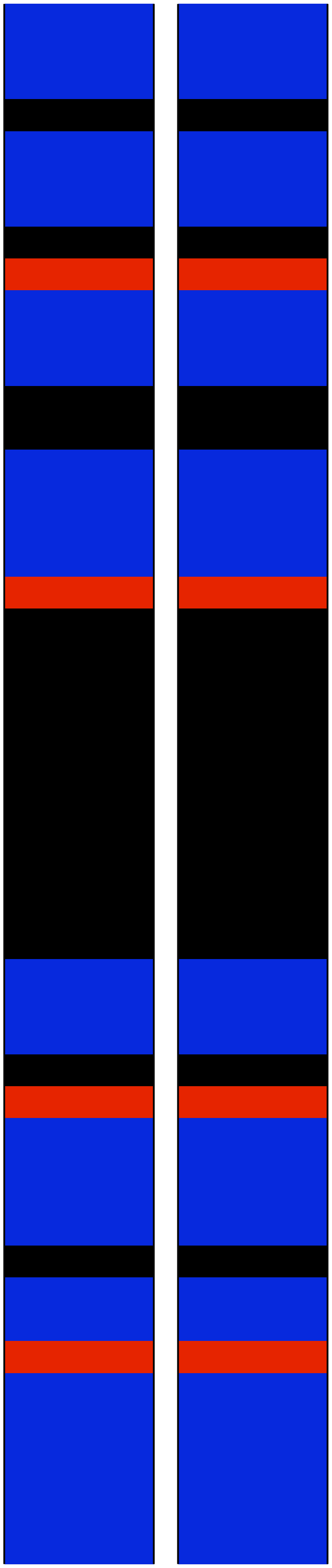

3	51	nr0223	ref|NC_002745.2|:258866-261115
3	52	nr0224	ref|NC_002745.2|:261138-261893
3	53	nr0225	ref|NC_002745.2|:262215-263978
3	54	nr0226	ref|NC_002745.2|:c264486-264142
3	55	nr0227	ref|NC_002745.2|:264676-266652
3	56	nr0228	ref|NC_002745.2|:c268423-267239
3	57	nr0229	ref|NC_002745.2|:c270714-268453
3	58	nr0230	ref|NC_002745.2|:c272111-270900
3	59	nr0231	ref|NC_002745.2|:c273728-272223
3	60	nr0232	ref|NC_002745.2|:c275316-273754
3	61	nr0233	ref|NC_002745.2|:275772-276914
3	62	nr0234	ref|NC_002745.2|:c278703-277228
3	63	nr0235	ref|NC_002745.2|:c279257-278901
3	64	nr0236	ref|NC_002745.2|:c279587-279414
3	65	nr0237	ref|NC_002745.2|:c280758-279613
3	66	nr0238	ref|NC_002745.2|:281331-282284
3	67	nr0239	ref|NC_002745.2|:c284133-282604
3	68	nr0240	ref|NC_002745.2|:284495-285430
3	69	nr0241	ref|NC_002745.2|:285767-287863
3	70	nr0242	ref|NC_002745.2|:287848-288315
3	71	nr0243	ref|NC_002745.2|:288338-288616
3	72	nr0244	ref|NC_002745.2|:288843-290102
3	73	nr0245	ref|NC_002745.2|:290120-291175
3	74	nr0246	ref|NC_002745.2|:291177-291323
3	75	nr0247	ref|NC_002745.2|:291347-292390
3	76	nr0248	ref|NC_002745.2|:292918-293634
3	77	nr0249	ref|NC_002745.2|:293627-294652
3	78	nr0250	ref|NC_002745.2|:294674-296368
3	79	nr0251	ref|NC_002745.2|:296796-297965
3	80	nr0252	ref|NC_002745.2|:298241-298957
3	81	nr0253	ref|NC_002745.2|:298950-299975
3	82	nr0254	ref|NC_002745.2|:299997-301685
3	83	nr0255	ref|NC_002745.2|:301718-303439
3	84	nr0256	ref|NC_002745.2|:303583-304257
3	85	nr0257	ref|NC_002745.2|:304502-306256
3	86	nr0258	ref|NC_002745.2|:306259-306999
4	1	nr0259	ref|NC_002745.2|:307118-307555
4	2	nr0260	ref|NC_002745.2|:307548-308249
4	3	nr0261	ref|NC_002745.2|:c309061-308357
4	4	nr0262	ref|NC_002745.2|:309210-310001
4	5	nr0263	ref|NC_002745.2|:310017-311453
4	6	nr0264	ref|NC_002745.2|:c312701-311940
4	7	nr0265	ref|NC_002745.2|:c313866-312952
4	8	nr0266	ref|NC_002745.2|:c314298-313894
4	9	nr0267	ref|NC_002745.2|:c315194-314313
4	10	nr0268	ref|NC_002745.2|:c316424-315426
4	11	nr0269	ref|NC_002745.2|:316804-317196
4	12	nr0270	ref|NC_002745.2|:c318700-317324
4	13	nr0271	ref|NC_002745.2|:318934-319926

4	14	nr0272	ref|NC_002745.2|:320252-321202
4	15	nr0273	ref|NC_002745.2|:c321913-321254
4	16	nr0274	ref|NC_002745.2|:c322847-321927
4	17	nr0275	ref|NC_002745.2|:c324010-322844
4	18	nr0276	ref|NC_002745.2|:c325601-324078
4	19	nr0277	ref|NC_002745.2|:c326825-325932
4	20	nr0278	ref|NC_002745.2|:327073-327366
4	21	nr0279	ref|NC_002745.2|:327449-330478
4	22	nr0280	ref|NC_002745.2|:330478-330936
4	23	nr0281	ref|NC_002745.2|:330908-331150
4	24	nr0282	ref|NC_002745.2|:331163-332497
4	25	nr0283	ref|NC_002745.2|:332519-336958
4	26	nr0284	ref|NC_002745.2|:336988-337380
4	27	nr0285	ref|NC_002745.2|:337396-337710
4	28	nr0286	ref|NC_002745.2|:337707-338384
4	29	nr0287	ref|NC_002745.2|:338384-338701
4	30	nr0288	ref|NC_002745.2|:338711-340555
4	31	nr0289	ref|NC_002745.2|:340566-341057
4	32	nr0290	ref|NC_002745.2|:341260-341943
4	33	nr0291	ref|NC_002745.2|:342089-342700
4	34	nr0292	ref|NC_002745.2|:342835-343053
4	35	nr0293	ref|NC_002745.2|:343185-343685
4	36	nr0294	ref|NC_002745.2|:343696-344196
4	37	nr0295	ref|NC_002745.2|:344207-344668
4	38	nr0296	ref|NC_002745.2|:344717-345217
4	39	nr0297	ref|NC_002745.2|:345228-345713
4	40	nr0298	ref|NC_002745.2|:346338-346712
4	41	nr0299	ref|NC_002745.2|:346862-347260
4	42	nr0300	ref|NC_002745.2|:c348300-347476
4	43	nr0301	ref|NC_002745.2|:c349844-348537
4	44	nr0302	ref|NC_002745.2|:350428-351318
4	45	nr0303	ref|NC_002745.2|:351567-352616
4	46	nr0304	ref|NC_002745.2|:352629-353306
4	47	nr0305	ref|NC_002745.2|:353514-354545
4	48	nr0306	ref|NC_002745.2|:354888-355649
4	49	nr0307	ref|NC_002745.2|:355764-356006
4	50	nr0308	ref|NC_002745.2|:355981-356904
4	51	nr0309	ref|NC_002745.2|:356915-358135
4	52	nr0310	ref|NC_002745.2|:c359772-358240
4	53	nr0311	ref|NC_002745.2|:c360693-359812
4	54	nr0312	ref|NC_002745.2|:360851-361711
4	55	nr0313	ref|NC_002745.2|:c362788-361988
4	56	nr0314	ref|NC_002745.2|:362928-363596
4	57	nr0315	ref|NC_002745.2|:c365040-363727
4	58	nr0316	ref|NC_002745.2|:365458-367533
4	59	nr0317	ref|NC_002745.2|:c368602-367775
4	60	nr0318	ref|NC_002745.2|:c369874-368675
4	61	nr0319	ref|NC_002745.2|:370176-371177
4	62	nr0320	ref|NC_002745.2|:371211-371543

4	63	nr0321	ref|NC_002745.2|:371544-372344
4	64	nr0322	ref|NC_002745.2|:372331-373278
4	65	nr0323	ref|NC_002745.2|:373256-374278
4	66	nr0324	ref|NC_002745.2|:374594-375619
4	67	nr0325	ref|NC_002745.2|:c377059-375713
4	68	nr0326	ref|NC_002745.2|:c377358-377074
4	69	nr0327	ref|NC_002745.2|:c377803-377360
4	70	nr0328	ref|NC_002745.2|:c379763-377808
4	71	nr0329	ref|NC_002745.2|:379975-380394
4	72	nr0330	ref|NC_002745.2|:380501-381856
4	73	nr0331	ref|NC_002745.2|:381960-382418
4	74	nr0332	ref|NC_002745.2|:c383840-382482
4	75	nr0333	ref|NC_002745.2|:384152-385078
4	76	nr0334	ref|NC_002745.2|:385092-386153
4	77	nr0335	ref|NC_002745.2|:386167-386733
4	78	nr0336	ref|NC_002745.2|:c387787-386792
4	79	nr0337	ref|NC_002745.2|:388152-388697
4	80	nr0338	ref|NC_002745.2|:388964-389818
4	81	nr0339	ref|NC_002745.2|:389929-391044
4	82	nr0340	ref|NC_002745.2|:391022-392737
4	83	nr0341	ref|NC_002745.2|:c393640-392978
4	84	nr0342	ref|NC_002745.2|:c393911-393696
4	85	nr0343	ref|NC_002745.2|:c394408-394019
4	86	nr0344	ref|NC_002745.2|:394648-394851
5	1	nr0345	ref|NC_002745.2|:394848-395561
5	2	nr0346	ref|NC_002745.2|:395586-396428
5	3	nr0347	ref|NC_002745.2|:396428-397057
5	4	nr0348	ref|NC_002745.2|:c398577-397444
5	5	nr0349	ref|NC_002745.2|:399116-400297
5	6	nr0350	ref|NC_002745.2|:c401132-400380
5	7	nr0351	ref|NC_002745.2|:c403403-401175
5	8	nr0352	ref|NC_002745.2|:c405241-403400
5	9	nr0353	ref|NC_002745.2|:c406370-405210
5	10	nr0354	ref|NC_002745.2|:c407470-406367
5	11	nr0355	ref|NC_002745.2|:408141-408977
5	12	nr0356	ref|NC_002745.2|:409131-410012
5	13	nr0357	ref|NC_002745.2|:410042-410245
5	14	nr0358	ref|NC_002745.2|:410257-411354
5	15	nr0359	ref|NC_002745.2|:c411631-411440
5	16	nr0360	ref|NC_002745.2|:411875-412171
5	17	nr0361	ref|NC_002745.2|:412192-412695
5	18	nr0362	ref|NC_002745.2|:412747-412989
5	19	nr0363	ref|NC_002745.2|:c414240-413266
5	20	nr0364	ref|NC_002745.2|:c414546-414292
5	21	nr0365	ref|NC_002745.2|:415162-415773
5	22	nr0366	ref|NC_002745.2|:416142-416510
5	23	nr0367	ref|NC_002745.2|:416691-417263
5	24	nr0368	ref|NC_002745.2|:c417663-417400
5	25	nr0369	ref|NC_002745.2|:417963-418214

5	26	nr0370	ref|NC_002745.2|:c418462-418253
5	27	nr0371	ref|NC_002745.2|:418616-419221
5	28	nr0372	ref|NC_002745.2|:c419670-419287
5	29	nr0373	ref|NC_002745.2|:c420566-419940
5	30	nr0374	ref|NC_002745.2|:c420830-420639
5	31	nr0375	ref|NC_002745.2|:c422533-421010
5	32	nr0376	ref|NC_002745.2|:c423118-422549
5	33	nr0377	ref|NC_002745.2|:423610-424365
5	34	nr0378	ref|NC_002745.2|:c425833-424445
5	35	nr0379	ref|NC_002745.2|:426680-427999
5	36	nr0380	ref|NC_002745.2|:c429287-428331
5	37	nr0381	ref|NC_002745.2|:c430067-429405
5	38	nr0382	ref|NC_002745.2|:c430617-430210
5	39	nr0383	ref|NC_002745.2|:431130-431708
5	40	nr0384	ref|NC_002745.2|:431708-432976
5	41	nr0385	ref|NC_002745.2|:433014-434480
5	42	nr0386	ref|NC_002745.2|:434505-436046
5	43	nr0387	ref|NC_002745.2|:c436695-436159
5	44	nr0388	ref|NC_002745.2|:c437791-437087
5	45	nr0389	ref|NC_002745.2|:c438486-438190
5	46	nr0390	ref|NC_002745.2|:c438875-438723
5	47	nr0391	ref|NC_002745.2|:c439766-439407
5	48	nr0392	ref|NC_002745.2|:c440630-439785
5	49	nr0393	ref|NC_002745.2|:441102-441782
5	50	nr0394	ref|NC_002745.2|:442068-442763
5	51	nr0395	ref|NC_002745.2|:443054-444124
5	52	nr0396	ref|NC_002745.2|:444488-445366
5	53	nr0397	ref|NC_002745.2|:445730-446434
5	54	nr0398	ref|NC_002745.2|:446880-447575
5	55	nr0399	ref|NC_002745.2|:447914-448612
5	56	nr0400	ref|NC_002745.2|:448989-449687
5	57	nr0401	ref|NC_002745.2|:450053-450736
5	58	nr0402	ref|NC_002745.2|:450789-450947
5	59	nr0403	ref|NC_002745.2|:451000-452556
5	60	nr0404	ref|NC_002745.2|:452549-453760
5	61	nr0405	ref|NC_002745.2|:454143-454826
5	62	nr0406	ref|NC_002745.2|:454848-456335
5	63	nr0407	ref|NC_002745.2|:c456752-456444
5	64	nr0408	ref|NC_002745.2|:457089-457892
5	65	nr0409	ref|NC_002745.2|:457923-458717
5	66	nr0410	ref|NC_002745.2|:458762-459550
5	67	nr0411	ref|NC_002745.2|:459874-460356
5	68	nr0412	ref|NC_002745.2|:460417-461202
5	69	nr0413	ref|NC_002745.2|:461221-462003
5	70	nr0414	ref|NC_002745.2|:462060-462854
5	71	nr0415	ref|NC_002745.2|:462906-463706
5	72	nr0416	ref|NC_002745.2|:463725-464507
5	73	nr0417	ref|NC_002745.2|:464686-465501
5	74	nr0418	ref|NC_002745.2|:465491-466816

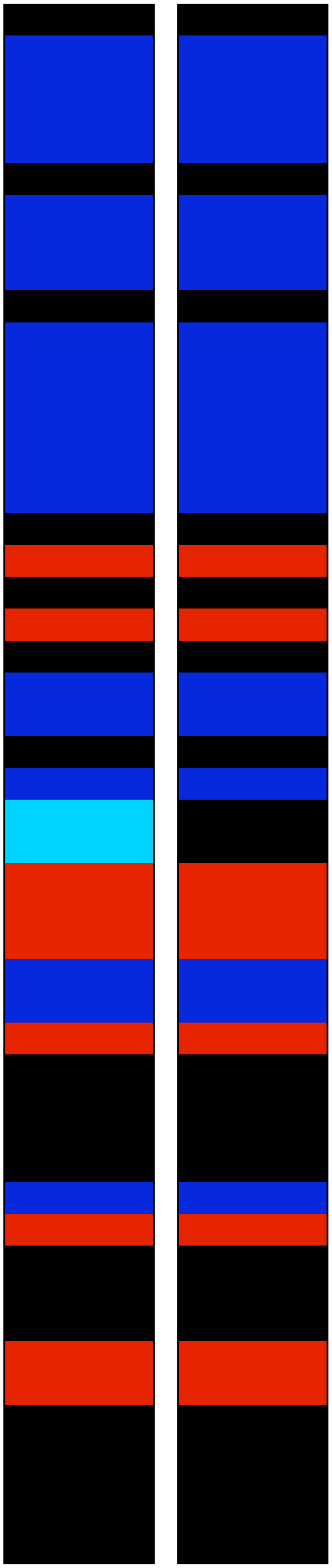

5	75	nr0419	ref|NC_002745.2|:466813-467136
5	76	nr0420	ref|NC_002745.2|:467155-467469
5	77	nr0421	ref|NC_002745.2|:c467958-467758
5	78	nr0422	ref|NC_002745.2|:468137-469339
5	79	nr0423	ref|NC_002745.2|:c469896-469753
5	80	nr0424	ref|NC_002745.2|:470927-472411
5	81	nr0425	ref|NC_002745.2|:472424-475129
5	82	nr0426	ref|NC_002745.2|:475291-475653
5	83	nr0427	ref|NC_002745.2|:c476124-475882
5	84	nr0428	ref|NC_002745.2|:476336-477010
5	85	nr0429	ref|NC_002745.2|:477047-477781
5	86	nr0430	ref|NC_002745.2|:478150-479490
6	1	nr0431	ref|NC_002745.2|:479707-480612
6	2	nr0432	ref|NC_002745.2|:480605-481747
6	3	nr0433	ref|NC_002745.2|:482042-483067
6	4	nr0434	ref|NC_002745.2|:483071-483730
6	5	nr0435	ref|NC_002745.2|:483767-484609
6	6	nr0436	ref|NC_002745.2|:484941-485945
6	7	nr0437	ref|NC_002745.2|:c486408-486130
6	8	nr0438	ref|NC_002745.2|:486548-486943
6	9	nr0439	ref|NC_002745.2|:486933-487418
6	10	nr0440	ref|NC_002745.2|:c488369-487587
6	11	nr0441	ref|NC_002745.2|:c489478-488366
6	12	nr0442	ref|NC_002745.2|:c490483-489599
6	13	nr0443	ref|NC_002745.2|:490664-495163
6	14	nr0444	ref|NC_002745.2|:495181-496644
6	15	nr0445	ref|NC_002745.2|:497474-498901
6	16	nr0446	ref|NC_002745.2|:498965-500605
6	17	nr0447	ref|NC_002745.2|:500630-501358
6	18	nr0448	ref|NC_002745.2|:502001-502525
6	19	nr0449	ref|NC_002745.2|:502594-504291
6	20	nr0450	ref|NC_002745.2|:504381-504698
6	21	nr0451	ref|NC_002745.2|:504705-505301
6	22	nr0452	ref|NC_002745.2|:512201-513538
6	23	nr0453	ref|NC_002745.2|:513540-514157
6	24	nr0454	ref|NC_002745.2|:514185-514514
6	25	nr0455	ref|NC_002745.2|:514728-515654
6	26	nr0456	ref|NC_002745.2|:515823-516458
6	27	nr0457	ref|NC_002745.2|:516454-516822
6	28	nr0458	ref|NC_002745.2|:517096-517821
6	29	nr0459	ref|NC_002745.2|:517814-518062
6	30	nr0460	ref|NC_002745.2|:518064-518903
6	31	nr0461	ref|NC_002745.2|:519188-521161
6	32	nr0462	ref|NC_002745.2|:521192-521965
6	33	nr0463	ref|NC_002745.2|:522132-522668
6	34	nr0464	ref|NC_002745.2|:522679-523572
6	35	nr0465	ref|NC_002745.2|:523672-523935
6	36	nr0466	ref|NC_002745.2|:524245-525093
6	37	nr0467	ref|NC_002745.2|:525107-525931

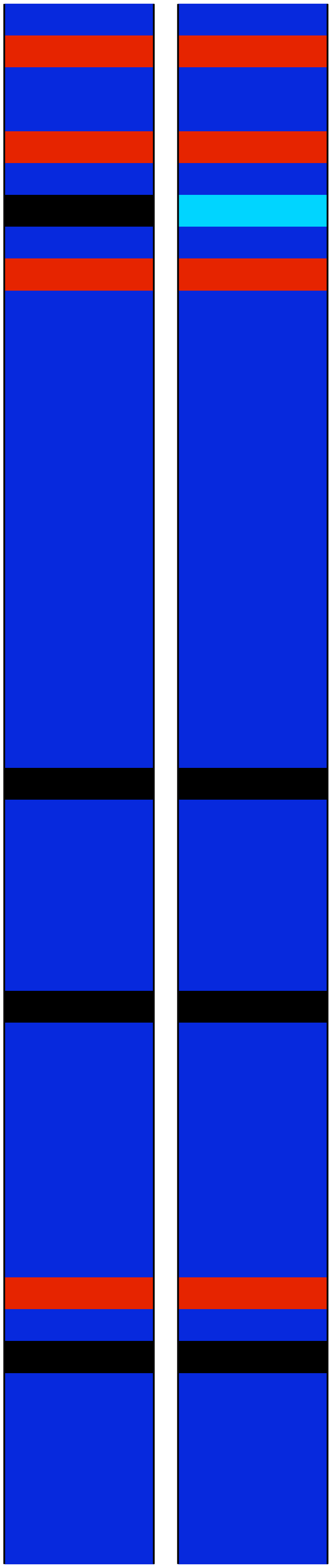

6	38	nr0468	ref|NC_002745.2|:525948-526328
6	39	nr0469	ref|NC_002745.2|:526376-526702
6	40	nr0470	ref|NC_002745.2|:527067-528419
6	41	nr0471	ref|NC_002745.2|:528566-529531
6	42	nr0472	ref|NC_002745.2|:529681-530334
6	43	nr0473	ref|NC_002745.2|:530645-531217
6	44	nr0474	ref|NC_002745.2|:531217-534723
6	45	nr0475	ref|NC_002745.2|:534713-536239
6	46	nr0476	ref|NC_002745.2|:536239-537432
6	47	nr0477	ref|NC_002745.2|:537429-537692
6	48	nr0478	ref|NC_002745.2|:537710-538102
6	49	nr0479	ref|NC_002745.2|:538207-538608
6	50	nr0480	ref|NC_002745.2|:538788-540083
6	51	nr0481	ref|NC_002745.2|:540088-540627
6	52	nr0482	ref|NC_002745.2|:540884-542977
6	53	nr0483	ref|NC_002745.2|:543205-544086
6	54	nr0484	ref|NC_002745.2|:544265-545197
6	55	nr0485	ref|NC_002745.2|:545413-546216
6	56	nr0486	ref|NC_002745.2|:546194-546559
6	57	nr0487	ref|NC_002745.2|:546556-547032
6	58	nr0488	ref|NC_002745.2|:547571-549058
6	59	nr0489	ref|NC_002745.2|:c557494-556112
6	60	nr0490	ref|NC_002745.2|:557598-558485
6	61	nr0491	ref|NC_002745.2|:558489-559049
6	62	nr0492	ref|NC_002745.2|:c560471-559257
6	63	nr0493	ref|NC_002745.2|:560629-561090
6	64	nr0494	ref|NC_002745.2|:561109-561675
6	65	nr0495	ref|NC_002745.2|:561665-562672
6	66	nr0496	ref|NC_002745.2|:562686-565142
6	67	nr0497	ref|NC_002745.2|:565627-566991
6	68	nr0498	ref|NC_002745.2|:567016-568089
6	69	nr0499	ref|NC_002745.2|:568646-570100
6	70	nr0500	ref|NC_002745.2|:570529-571170
6	71	nr0501	ref|NC_002745.2|:571154-572554
6	72	nr0502	ref|NC_002745.2|:572547-572951
6	73	nr0503	ref|NC_002745.2|:572959-573705
6	74	nr0504	ref|NC_002745.2|:573705-574229
6	75	nr0505	ref|NC_002745.2|:574310-574879
6	76	nr0506	ref|NC_002745.2|:575193-575375
6	77	nr0507	ref|NC_002745.2|:575388-575936
6	78	nr0508	ref|NC_002745.2|:576074-576124
6	79	nr0509	ref|NC_002745.2|:576117-576539
6	80	nr0510	ref|NC_002745.2|:576747-577439
6	81	nr0511	ref|NC_002745.2|:577711-578211
6	82	nr0512	ref|NC_002745.2|:578254-578622
6	83	nr0513	ref|NC_002745.2|:578797-579405
6	84	nr0514	ref|NC_002745.2|:579620-583171
6	85	nr0515	ref|NC_002745.2|:583308-586931
6	86	nr0516	ref|NC_002745.2|:587068-587322

7	1	nr0517	ref|NC_002745.2|:587420-587833
7	2	nr0518	ref|NC_002745.2|:587899-588369
7	3	nr0519	ref|NC_002745.2|:588492-590573
7	4	nr0520	ref|NC_002745.2|:590790-591974
7	5	nr0521	ref|NC_002745.2|:c593431-592256
7	6	nr0522	ref|NC_002745.2|:593638-594789
7	7	nr0523	ref|NC_002745.2|:595059-595937
7	8	nr0524	ref|NC_002745.2|:596096-597733
7	9	nr0525	ref|NC_002745.2|:597947-598912
7	10	nr0526	ref|NC_002745.2|:599248-600324
7	11	nr0527	ref|NC_002745.2|:600574-601257
7	12	nr0528	ref|NC_002745.2|:c602035-601373
7	13	nr0529	ref|NC_002745.2|:c602645-602028
7	14	nr0530	ref|NC_002745.2|:602712-603182
7	15	nr0531	ref|NC_002745.2|:603329-604198
7	16	nr0532	ref|NC_002745.2|:604220-604786
7	17	nr0533	ref|NC_002745.2|:605215-608076
7	18	nr0534	ref|NC_002745.2|:608443-612600
7	19	nr0535	ref|NC_002745.2|:612994-616419
7	20	nr0536	ref|NC_002745.2|:616540-618012
7	21	nr0537	ref|NC_002745.2|:c619627-618137
7	22	nr0538	ref|NC_002745.2|:c620787-619909
7	23	nr0539	ref|NC_002745.2|:c621465-620800
7	24	nr0540	ref|NC_002745.2|:c621838-621479
7	25	nr0541	ref|NC_002745.2|:622117-622875
7	26	nr0542	ref|NC_002745.2|:622952-623584
7	27	nr0543	ref|NC_002745.2|:623586-624134
7	28	nr0544	ref|NC_002745.2|:624247-624894
7	29	nr0545	ref|NC_002745.2|:625396-626796
7	30	nr0546	ref|NC_002745.2|:627131-627340
7	31	nr0547	ref|NC_002745.2|:627340-628716
7	32	nr0548	ref|NC_002745.2|:628718-629857
7	33	nr0549	ref|NC_002745.2|:629832-630197
7	34	nr0550	ref|NC_002745.2|:630200-630469
7	35	nr0551	ref|NC_002745.2|:c630837-630670
7	36	nr0552	ref|NC_002745.2|:c632174-631344
7	37	nr0553	ref|NC_002745.2|:632358-633014
7	38	nr0554	ref|NC_002745.2|:633015-633395
7	39	nr0555	ref|NC_002745.2|:633528-633896
7	40	nr0556	ref|NC_002745.2|:634017-635501
7	41	nr0557	ref|NC_002745.2|:c636094-635642
7	42	nr0558	ref|NC_002745.2|:c636757-636107
7	43	nr0559	ref|NC_002745.2|:c638172-637420
7	44	nr0560	ref|NC_002745.2|:638340-639326
7	45	nr0561	ref|NC_002745.2|:639329-640165
7	46	nr0562	ref|NC_002745.2|:640833-641672
7	47	nr0563	ref|NC_002745.2|:641677-642660
7	48	nr0564	ref|NC_002745.2|:642673-643749
7	49	nr0565	ref|NC_002745.2|:643926-644267

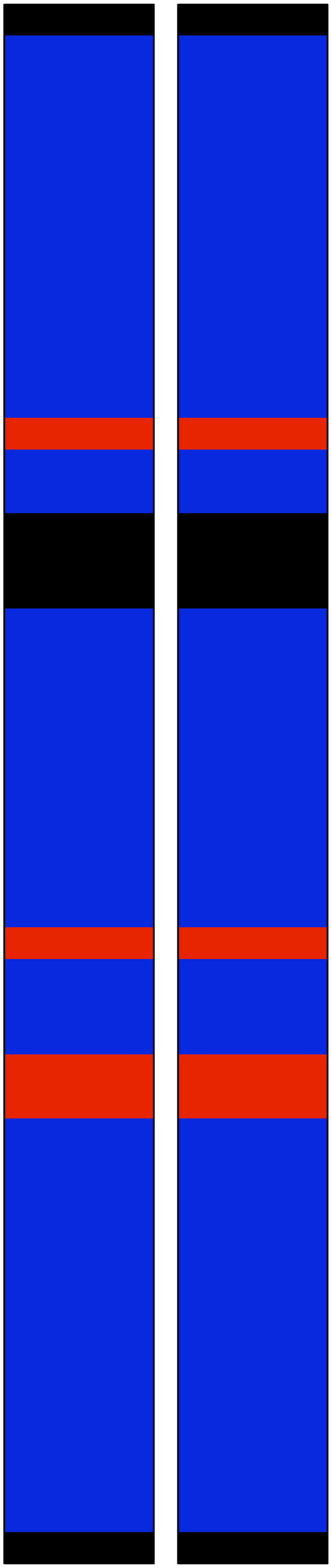

7	50	nr0566	ref|NC_002745.2|:c645767-644319
7	51	nr0567	ref|NC_002745.2|:c646068-645628
7	52	nr0568	ref|NC_002745.2|:646693-648087
7	53	nr0569	ref|NC_002745.2|:648091-648723
7	54	nr0570	ref|NC_002745.2|:648770-649390
7	55	nr0571	ref|NC_002745.2|:649530-650162
7	56	nr0572	ref|NC_002745.2|:650610-651548
7	57	nr0573	ref|NC_002745.2|:651994-652530
7	58	nr0574	ref|NC_002745.2|:652698-653174
7	59	nr0575	ref|NC_002745.2|:653214-654509
7	60	nr0576	ref|NC_002745.2|:654552-655058
7	61	nr0577	ref|NC_002745.2|:655557-656567
7	62	nr0578	ref|NC_002745.2|:656831-657259
7	63	nr0579	ref|NC_002745.2|:657256-658917
7	64	nr0580	ref|NC_002745.2|:659187-659822
7	65	nr0581	ref|NC_002745.2|:660074-660961
7	66	nr0582	ref|NC_002745.2|:661110-662060
7	67	nr0583	ref|NC_002745.2|:662115-662834
7	68	nr0584	ref|NC_002745.2|:662818-663618
7	69	nr0585	ref|NC_002745.2|:663754-664260
7	70	nr0586	ref|NC_002745.2|:664422-665138
7	71	nr0587	ref|NC_002745.2|:665307-666095
7	72	nr0588	ref|NC_002745.2|:c666721-666347
7	73	nr0589	ref|NC_002745.2|:c668598-667669
7	74	nr0590	ref|NC_002745.2|:c669017-668793
7	75	nr0591	ref|NC_002745.2|:c669237-669034
7	76	nr0592	ref|NC_002745.2|:669396-669956
7	77	nr0593	ref|NC_002745.2|:669975-672377
7	78	nr0594	ref|NC_002745.2|:672364-672789
7	79	nr0595	ref|NC_002745.2|:672786-673130
7	80	nr0596	ref|NC_002745.2|:673120-674616
7	81	nr0597	ref|NC_002745.2|:674617-675099
7	82	nr0598	ref|NC_002745.2|:675096-675398
7	83	nr0599	ref|NC_002745.2|:675373-675810
7	84	nr0600	ref|NC_002745.2|:676150-678192
7	85	nr0601	ref|NC_002745.2|:c679619-678300
7	86	nr0602	ref|NC_002745.2|:c681668-680739
8	1	nr0603	ref|NC_002745.2|:c682501-681665
8	2	nr0604	ref|NC_002745.2|:c683238-682495
8	3	nr0605	ref|NC_002745.2|:683360-684004
8	4	nr0606	ref|NC_002745.2|:c684836-684084
8	5	nr0607	ref|NC_002745.2|:685072-685836
8	6	nr0608	ref|NC_002745.2|:c686691-685897
8	7	nr0609	ref|NC_002745.2|:687017-687850
8	8	nr0610	ref|NC_002745.2|:688582-689052
8	9	nr0611	ref|NC_002745.2|:689103-690110
8	10	nr0612	ref|NC_002745.2|:690173-690571
8	11	nr0613	ref|NC_002745.2|:c691983-690688
8	12	nr0614	ref|NC_002745.2|:692404-694131

8	13	nr0615	ref|NC_002745.2|:694200-694280		
8	14	nr0616	ref|NC_002745.2|:694488-695717		
8	15	nr0617	ref|NC_002745.2|:696464-697075		
8	16	nr0618	ref|NC_002745.2|:697357-698154		
8	17	nr0619	ref|NC_002745.2|:698190-699194		
8	18	nr0620	ref|NC_002745.2|:699191-700207		
8	19	nr0621	ref|NC_002745.2|:700444-701409		
8	20	nr0622	ref|NC_002745.2|:701451-702035		
8	21	nr0623	ref|NC_002745.2|:702028-702390		
8	22	nr0624	ref|NC_002745.2|:702506-703003		
8	23	nr0625	ref|NC_002745.2|:703547-704527		
8	24	nr0626	ref|NC_002745.2|:704678-705721		
8	25	nr0627	ref|NC_002745.2|:706050-706478		
8	26	nr0628	ref|NC_002745.2|:c707193-706687		
8	27	nr0629	ref|NC_002745.2|:707306-708229		
8	28	nr0630	ref|NC_002745.2|:708245-708919		
8	29	nr0631	ref|NC_002745.2|:708912-709952		
8	30	nr0632	ref|NC_002745.2|:710096-710857		
8	31	nr0633	ref|NC_002745.2|:710835-712736		
8	32	nr0634	ref|NC_002745.2|:713459-714076		
8	33	nr0635	ref|NC_002745.2|:714092-715099		
8	34	nr0636	ref|NC_002745.2|:c716484-715687		
8	35	nr0637	ref|NC_002745.2|:716845-717489		
8	36	nr0638	ref|NC_002745.2|:718186-720336		
8	37	nr0639	ref|NC_002745.2|:720416-720841		
8	38	nr0640	ref|NC_002745.2|:721307-721747		
8	39	nr0641	ref|NC_002745.2|:722032-722220		
8	40	nr0642	ref|NC_002745.2|:722573-723217		
8	41	nr0643	ref|NC_002745.2|:723519-724199		
8	42	nr0644	ref|NC_002745.2|:724337-725551		
8	43	nr0645	ref|NC_002745.2|:725548-726036		
8	44	nr0646	ref|NC_002745.2|:c726824-726135		
8	45	nr0647	ref|NC_002745.2|:726951-727394		
8	46	nr0648	ref|NC_002745.2|:727461-727856		
8	47	nr0649	ref|NC_002745.2|:727994-728293		
8	48	nr0650	ref|NC_002745.2|:728373-728915		
8	49	nr0651	ref|NC_002745.2|:c729577-729011		
8	50	nr0652	ref|NC_002745.2|:c730037-729579		
8	51	nr0653	ref|NC_002745.2|:c730723-730040		
8	52	nr0654	ref|NC_002745.2|:c731770-730895		
8	53	nr0655	ref|NC_002745.2|:731989-733620		
8	54	nr0656	ref|NC_002745.2|:733617-735290		
8	55	nr0657	ref|NC_002745.2|:c735860-735417		
8	56	nr0658	ref|NC_002745.2|:736087-737013		
8	57	nr0659	ref|NC_002745.2|:737116-738024		
8	58	nr0660	ref|NC_002745.2|:738059-738346		
8	59	nr0661	ref|NC_002745.2|:738629-740182		
8	60	nr0662	ref|NC_002745.2|:740268-741641		
8	61	nr0663	ref|NC_002745.2|:c742080-741796		

8	62	nr0664	ref|NC_002745.2|:c742697-742077
8	63	nr0665	ref|NC_002745.2|:c743302-742880
8	64	nr0666	ref|NC_002745.2|:743511-744677
8	65	nr0667	ref|NC_002745.2|:744970-745422
8	66	nr0668	ref|NC_002745.2|:745596-746078
8	67	nr0669	ref|NC_002745.2|:746331-747092
8	68	nr0670	ref|NC_002745.2|:747089-748009
8	69	nr0671	ref|NC_002745.2|:748015-749973
8	70	nr0672	ref|NC_002745.2|:750281-751462
8	71	nr0673	ref|NC_002745.2|:751683-753032
8	72	nr0674	ref|NC_002745.2|:753254-754093
8	73	nr0675	ref|NC_002745.2|:754225-755208
8	74	nr0676	ref|NC_002745.2|:c756335-755280
8	75	nr0677	ref|NC_002745.2|:c757021-756335
8	76	nr0678	ref|NC_002745.2|:c757178-756996
8	77	nr0679	ref|NC_002745.2|:c758252-757812
8	78	nr0680	ref|NC_002745.2|:c759116-758532
8	79	nr0681	ref|NC_002745.2|:c759928-759215
8	80	nr0682	ref|NC_002745.2|:c760351-759932
8	81	nr0683	ref|NC_002745.2|:c761021-760353
8	82	nr0684	ref|NC_002745.2|:761372-761965
8	83	nr0685	ref|NC_002745.2|:761949-763100
8	84	nr0686	ref|NC_002745.2|:763100-763708
8	85	nr0687	ref|NC_002745.2|:763774-763980
8	86	nr0688	ref|NC_002745.2|:764068-764778
9	1	nr0689	ref|NC_002745.2|:764762-765766
9	2	nr0690	ref|NC_002745.2|:766240-768180
9	3	nr0691	ref|NC_002745.2|:768456-770333
9	4	nr0692	ref|NC_002745.2|:770345-772126
9	5	nr0693	ref|NC_002745.2|:772348-773325
9	6	nr0694	ref|NC_002745.2|:773318-774832
9	7	nr0695	ref|NC_002745.2|:775072-776130
9	8	nr0696	ref|NC_002745.2|:776469-777011
9	9	nr0697	ref|NC_002745.2|:c778807-777890
9	10	nr0698	ref|NC_002745.2|:c780582-779077
9	11	nr0699	ref|NC_002745.2|:c781423-780923
9	12	nr0700	ref|NC_002745.2|:c782309-781443
9	13	nr0701	ref|NC_002745.2|:783110-783508
9	14	nr0702	ref|NC_002745.2|:783420-785576
9	15	nr0703	ref|NC_002745.2|:785694-786665
9	16	nr0704	ref|NC_002745.2|:787040-788011
9	17	nr0705	ref|NC_002745.2|:787998-788954
9	18	nr0706	ref|NC_002745.2|:788951-789712
9	19	nr0707	ref|NC_002745.2|:789831-790859
9	20	nr0708	ref|NC_002745.2|:c791490-791176
9	21	nr0709	ref|NC_002745.2|:c792431-791508
9	22	nr0710	ref|NC_002745.2|:c793076-792558
9	23	nr0711	ref|NC_002745.2|:793196-794074
9	24	nr0712	ref|NC_002745.2|:794228-794548

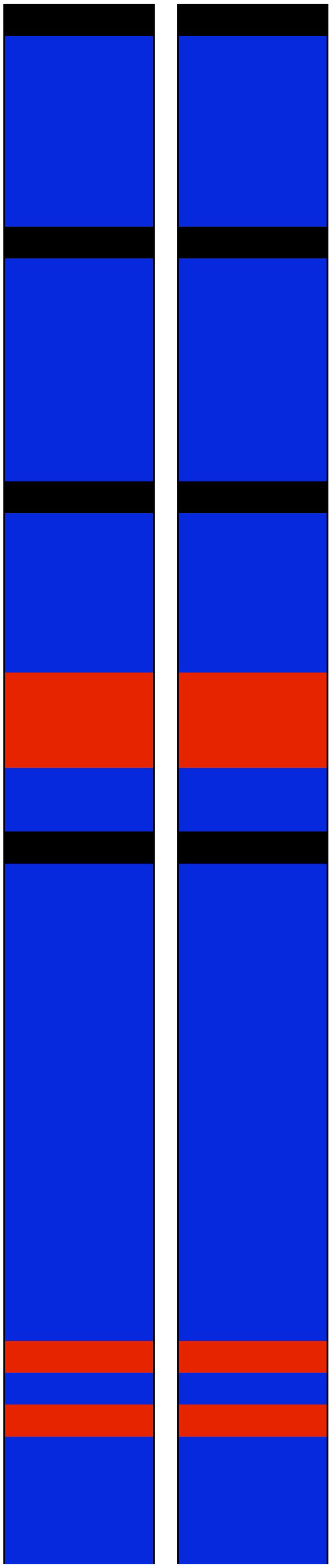

9	25	nr0713	ref|NC_002745.2|:794972-796096
9	26	nr0714	ref|NC_002745.2|:c797509-796283
9	27	nr0715	ref|NC_002745.2|:c798017-797523
9	28	nr0716	ref|NC_002745.2|:c798796-798035
9	29	nr0717	ref|NC_002745.2|:c800062-798992
9	30	nr0718	ref|NC_002745.2|:800380-801435
9	31	nr0719	ref|NC_002745.2|:c802241-801600
9	32	nr0720	ref|NC_002745.2|:802385-803251
9	33	nr0721	ref|NC_002745.2|:803795-804697
9	34	nr0722	ref|NC_002745.2|:804690-805364
9	35	nr0723	ref|NC_002745.2|:805425-805997
9	36	nr0724	ref|NC_002745.2|:806411-808942
9	37	nr0725	ref|NC_002745.2|:809375-810367
9	38	nr0726	ref|NC_002745.2|:810776-811615
9	39	nr0727	ref|NC_002745.2|:811789-812439
9	40	nr0728	ref|NC_002745.2|:812436-812672
9	41	nr0729	ref|NC_002745.2|:812935-814926
9	42	nr0730	ref|NC_002745.2|:814934-817780
9	43	nr0731	ref|NC_002745.2|:818444-819376
9	44	nr0732	ref|NC_002745.2|:819382-820221
9	45	nr0733	ref|NC_002745.2|:820229-820714
9	46	nr0734	ref|NC_002745.2|:820722-822161
9	47	nr0735	ref|NC_002745.2|:822228-823163
9	48	nr0736	ref|NC_002745.2|:824108-825019
9	49	nr0737	ref|NC_002745.2|:825016-826011
9	50	nr0738	ref|NC_002745.2|:826122-827066
9	51	nr0739	ref|NC_002745.2|:827630-828217
9	52	nr0740	ref|NC_002745.2|:c829362-828460
9	53	nr0741	ref|NC_002745.2|:830065-830694
9	54	nr0742	ref|NC_002745.2|:831619-832632
9	55	nr0743	ref|NC_002745.2|:832685-833695
9	56	nr0744	ref|NC_002745.2|:833834-835024
9	57	nr0745	ref|NC_002745.2|:835146-835907
9	58	nr0746	ref|NC_002745.2|:835910-837427
9	59	nr0747	ref|NC_002745.2|:837557-838861
9	60	nr0748	ref|NC_002745.2|:839200-839658
9	61	nr0749	ref|NC_002745.2|:839725-839958
9	62	nr0750	ref|NC_002745.2|:840074-840814
9	63	nr0751	ref|NC_002745.2|:840848-843220
9	64	nr0752	ref|NC_002745.2|:843242-843706
9	65	nr0753	ref|NC_002745.2|:844543-844761
9	66	nr0754	ref|NC_002745.2|:c845330-845028
9	67	nr0755	ref|NC_002745.2|:c846433-845705
9	68	nr0756	ref|NC_002745.2|:847031-847552
9	69	nr0757	ref|NC_002745.2|:847691-848221
9	70	nr0758	ref|NC_002745.2|:848484-851453
9	71	nr0759	ref|NC_002745.2|:851674-853176
9	72	nr0760	ref|NC_002745.2|:853529-854551
9	73	nr0761	ref|NC_002745.2|:854886-855407

9	74	nr0762	ref|NC_002745.2|:855748-856434
9	75	nr0763	ref|NC_002745.2|:856791-856991
9	76	nr0764	ref|NC_002745.2|:c857701-857483
9	77	nr0765	ref|NC_002745.2|:c858047-857763
9	78	nr0766	ref|NC_002745.2|:c858704-858135
9	79	nr0767	ref|NC_002745.2|:c859080-858892
9	80	nr0768	ref|NC_002745.2|:c859369-859109
9	81	nr0769	ref|NC_002745.2|:859710-859913
9	82	nr0770	ref|NC_002745.2|:c860146-859910
9	83	nr0771	ref|NC_002745.2|:860371-860952
9	84	nr0772	ref|NC_002745.2|:c861642-861025
9	85	nr0773	ref|NC_002745.2|:c862291-861797
9	86	nr0774	ref|NC_002745.2|:862499-862549
10	1	nr0775	ref|NC_002745.2|:c863112-862690
10	2	nr0776	ref|NC_002745.2|:863260-863976
10	3	nr0777	ref|NC_002745.2|:864059-864598
10	4	nr0778	ref|NC_002745.2|:c865069-864749
10	5	nr0779	ref|NC_002745.2|:865213-865569
10	6	nr0780	ref|NC_002745.2|:865727-866107
10	7	nr0781	ref|NC_002745.2|:866286-866906
10	8	nr0782	ref|NC_002745.2|:867008-868093
10	9	nr0783	ref|NC_002745.2|:868090-869982
10	10	nr0784	ref|NC_002745.2|:869989-870366
10	11	nr0785	ref|NC_002745.2|:870517-871299
10	12	nr0786	ref|NC_002745.2|:c872156-871425
10	13	nr0787	ref|NC_002745.2|:872690-873352
10	14	nr0788	ref|NC_002745.2|:873613-873876
10	15	nr0789	ref|NC_002745.2|:874673-875059
10	16	nr0790	ref|NC_002745.2|:875052-875348
10	17	nr0791	ref|NC_002745.2|:875598-876623
10	18	nr0792	ref|NC_002745.2|:876616-877311
10	19	nr0793	ref|NC_002745.2|:877329-878150
10	20	nr0794	ref|NC_002745.2|:878504-878698
10	21	nr0795	ref|NC_002745.2|:c879783-878932
10	22	nr0796	ref|NC_002745.2|:880111-880872
10	23	nr0797	ref|NC_002745.2|:880970-882277
10	24	nr0798	ref|NC_002745.2|:882392-883633
10	25	nr0799	ref|NC_002745.2|:883623-884087
10	26	nr0800	ref|NC_002745.2|:884238-885635
10	27	nr0801	ref|NC_002745.2|:c886281-885967
10	28	nr0802	ref|NC_002745.2|:886646-887686
10	29	nr0803	ref|NC_002745.2|:887700-888767
10	30	nr0804	ref|NC_002745.2|:889152-890000
10	31	nr0805	ref|NC_002745.2|:890013-890840
10	32	nr0806	ref|NC_002745.2|:890867-892186
10	33	nr0807	ref|NC_002745.2|:892270-893187
10	34	nr0808	ref|NC_002745.2|:893317-893700
10	35	nr0809	ref|NC_002745.2|:893906-895225
10	36	nr0810	ref|NC_002745.2|:c895565-895299

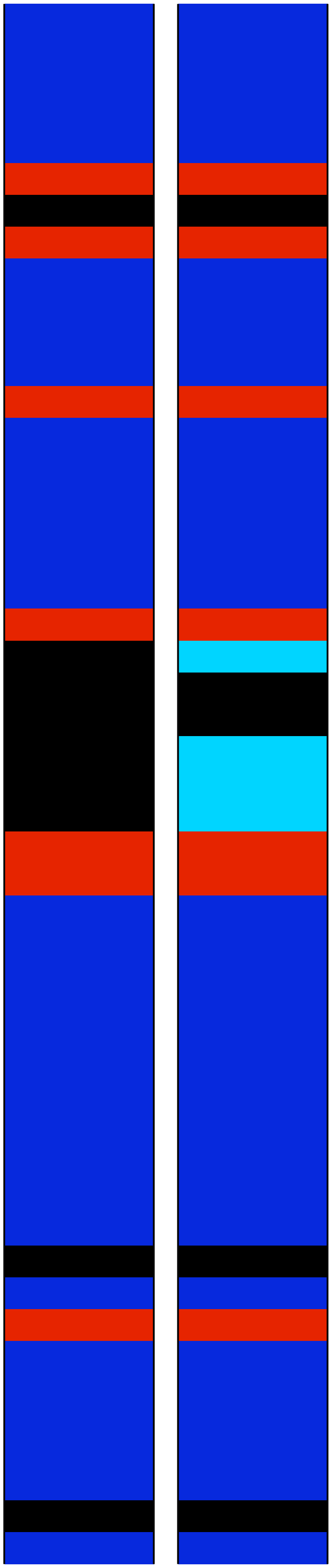

10	37	nr0811	ref|NC_002745.2|:895663-896097
10	38	nr0812	ref|NC_002745.2|:896097-896876
10	39	nr0813	ref|NC_002745.2|:896909-897868
10	40	nr0814	ref|NC_002745.2|:898280-898432
10	41	nr0815	ref|NC_002745.2|:898448-899905
10	42	nr0816	ref|NC_002745.2|:899902-901116
10	43	nr0817	ref|NC_002745.2|:901134-901370
10	44	nr0818	ref|NC_002745.2|:901367-902542
10	45	nr0819	ref|NC_002745.2|:c903047-902805
10	46	nr0820	ref|NC_002745.2|:903163-903471
10	47	nr0821	ref|NC_002745.2|:c904594-903530
10	48	nr0822	ref|NC_002745.2|:904911-905147
10	49	nr0823	ref|NC_002745.2|:905160-905519
10	50	nr0824	ref|NC_002745.2|:905973-907181
10	51	nr0825	ref|NC_002745.2|:907312-908787
10	52	nr0826	ref|NC_002745.2|:909198-910514
10	53	nr0827	ref|NC_002745.2|:910533-910907
10	54	nr0828	ref|NC_002745.2|:c912117-910963
10	55	nr0829	ref|NC_002745.2|:c912722-912366
10	56	nr0830	ref|NC_002745.2|:c912993-912700
10	57	nr0831	ref|NC_002745.2|:c913472-912993
10	58	nr0832	ref|NC_002745.2|:c914970-913474
10	59	nr0833	ref|NC_002745.2|:c915304-914963
10	60	nr0834	ref|NC_002745.2|:c915732-915304
10	61	nr0835	ref|NC_002745.2|:c918130-915725
10	62	nr0836	ref|NC_002745.2|:c918644-918261
10	63	nr0837	ref|NC_002745.2|:918708-919301
10	64	nr0838	ref|NC_002745.2|:919833-920093
10	65	nr0839	ref|NC_002745.2|:920455-921582
10	66	nr0840	ref|NC_002745.2|:921890-923080
10	67	nr0841	ref|NC_002745.2|:923189-924433
10	68	nr0842	ref|NC_002745.2|:c925699-924770
10	69	nr0843	ref|NC_002745.2|:c927320-925941
10	70	nr0844	ref|NC_002745.2|:c928515-927310
10	71	nr0845	ref|NC_002745.2|:928866-930197
10	72	nr0846	ref|NC_002745.2|:930522-931097
10	73	nr0847	ref|NC_002745.2|:931102-931626
10	74	nr0848	ref|NC_002745.2|:931642-932217
10	75	nr0849	ref|NC_002745.2|:932377-935850
10	76	nr0850	ref|NC_002745.2|:935854-939507
10	77	nr0851	ref|NC_002745.2|:939673-940575
10	78	nr0852	ref|NC_002745.2|:940904-941293
10	79	nr0853	ref|NC_002745.2|:c942781-941465
10	80	nr0854	ref|NC_002745.2|:c943657-942833
10	81	nr0855	ref|NC_002745.2|:943771-944079
10	82	nr0856	ref|NC_002745.2|:c944168-944076
10	83	nr0857	ref|NC_002745.2|:944627-946441
10	84	nr0858	ref|NC_002745.2|:946644-949253
10	85	nr0859	ref|NC_002745.2|:c950181-949312

10	86	nr0860	ref|NC_002745.2|:950291-951436
11	1	nr0861	ref|NC_002745.2|:951420-952061
11	2	nr0862	ref|NC_002745.2|:952054-953175
11	3	nr0863	ref|NC_002745.2|:953153-953668
11	4	nr0864	ref|NC_002745.2|:953979-954413
11	5	nr0865	ref|NC_002745.2|:c954855-954670
11	6	nr0866	ref|NC_002745.2|:955150-956091
11	7	nr0867	ref|NC_002745.2|:956103-957347
11	8	nr0868	ref|NC_002745.2|:c957773-957402
11	9	nr0869	ref|NC_002745.2|:958016-958942
11	10	nr0870	ref|NC_002745.2|:958942-960012
11	11	nr0871	ref|NC_002745.2|:960028-961110
11	12	nr0872	ref|NC_002745.2|:961100-962041
11	13	nr0873	ref|NC_002745.2|:962060-963715
11	14	nr0874	ref|NC_002745.2|:963927-965642
11	15	nr0875	ref|NC_002745.2|:965693-966679
11	16	nr0876	ref|NC_002745.2|:966682-967662
11	17	nr0877	ref|NC_002745.2|:967655-968617
11	18	nr0878	ref|NC_002745.2|:968629-969510
11	19	nr0879	ref|NC_002745.2|:c970541-969552
11	20	nr0880	ref|NC_002745.2|:970835-971230
11	21	nr0881	ref|NC_002745.2|:971601-972320
11	22	nr0882	ref|NC_002745.2|:972441-973427
11	23	nr0883	ref|NC_002745.2|:973475-975283
11	24	nr0884	ref|NC_002745.2|:c976528-975743
11	25	nr0885	ref|NC_002745.2|:c976937-976572
11	26	nr0886	ref|NC_002745.2|:c977634-977041
11	27	nr0887	ref|NC_002745.2|:977820-978167
11	28	nr0888	ref|NC_002745.2|:978184-978819
11	29	nr0889	ref|NC_002745.2|:978836-979645
11	30	nr0890	ref|NC_002745.2|:979642-980496
11	31	nr0891	ref|NC_002745.2|:980517-981902
11	32	nr0892	ref|NC_002745.2|:981912-983756
11	33	nr0893	ref|NC_002745.2|:984034-984804
11	34	nr0894	ref|NC_002745.2|:c986086-985001
11	35	nr0895	ref|NC_002745.2|:986491-987996
11	36	nr0896	ref|NC_002745.2|:988141-988899
11	37	nr0897	ref|NC_002745.2|:989093-989602
11	38	nr0898	ref|NC_002745.2|:c990905-989715
11	39	nr0899	ref|NC_002745.2|:c992058-990883
11	40	nr0900	ref|NC_002745.2|:992491-993975
11	41	nr0901	ref|NC_002745.2|:993965-994216
11	42	nr0902	ref|NC_002745.2|:994216-995778
11	43	nr0903	ref|NC_002745.2|:996080-996883
11	44	nr0904	ref|NC_002745.2|:997117-999426
11	45	nr0905	ref|NC_002745.2|:999443-1000801
11	46	nr0906	ref|NC_002745.2|:1000937-1002451
11	47	nr0907	ref|NC_002745.2|:c1003507-1002938
11	48	nr0908	ref|NC_002745.2|:1003717-1003935

11	49	nr0909	ref|NC_002745.2|:c1005002-1004016
11	50	nr0910	ref|NC_002745.2|:1005201-1005377
11	51	nr0911	ref|NC_002745.2|:1005392-1005994
11	52	nr0912	ref|NC_002745.2|:1006203-1006364
11	53	nr0913	ref|NC_002745.2|:1007021-1007293
11	54	nr0914	ref|NC_002745.2|:1007337-1009301
11	55	nr0915	ref|NC_002745.2|:1009304-1009624
11	56	nr0916	ref|NC_002745.2|:1009621-1010262
11	57	nr0917	ref|NC_002745.2|:c1010595-1010350
11	58	nr0918	ref|NC_002745.2|:c1011363-1011007
11	59	nr0919	ref|NC_002745.2|:1011851-1012810
11	60	nr0920	ref|NC_002745.2|:c1013266-1013051
11	61	nr0921	ref|NC_002745.2|:1013431-1013982
11	62	nr0922	ref|NC_002745.2|:c1014972-1014034
11	63	nr0923	ref|NC_002745.2|:1015154-1016515
11	64	nr0924	ref|NC_002745.2|:1016502-1018175
11	65	nr0925	ref|NC_002745.2|:1018162-1018965
11	66	nr0926	ref|NC_002745.2|:1018958-1019779
11	67	nr0927	ref|NC_002745.2|:c1020346-1020017
11	68	nr0928	ref|NC_002745.2|:c1021565-1020384
11	69	nr0929	ref|NC_002745.2|:c1022675-1021647
11	70	nr0930	ref|NC_002745.2|:c1024357-1023203
11	71	nr0931	ref|NC_002745.2|:c1025563-1024553
11	72	nr0932	ref|NC_002745.2|:1025716-1026135
11	73	nr0933	ref|NC_002745.2|:c1030088-1026342
11	74	nr0934	ref|NC_002745.2|:c1030750-1030316
11	75	nr0935	ref|NC_002745.2|:c1031375-1030905
11	76	nr0936	ref|NC_002745.2|:c1032640-1031423
11	77	nr0937	ref|NC_002745.2|:1033091-1034284
11	78	nr0938	ref|NC_002745.2|:c1035111-1034821
11	79	nr0939	ref|NC_002745.2|:c1035713-1035108
11	80	nr0940	ref|NC_002745.2|:c1037691-1035703
11	81	nr0941	ref|NC_002745.2|:c1038791-1037691
11	82	nr0942	ref|NC_002745.2|:c1039679-1039362
11	83	nr0943	ref|NC_002745.2|:c1041403-1040543
11	84	nr0944	ref|NC_002745.2|:1041604-1042086
11	85	nr0945	ref|NC_002745.2|:1042073-1043197
11	86	nr0946	ref|NC_002745.2|:1043201-1043905
12	1	nr0947	ref|NC_002745.2|:1043905-1044168
12	2	nr0948	ref|NC_002745.2|:1044170-1044841
12	3	nr0949	ref|NC_002745.2|:1044834-1047023
12	4	nr0950	ref|NC_002745.2|:1047002-1048486
12	5	nr0951	ref|NC_002745.2|:1048479-1049507
12	6	nr0952	ref|NC_002745.2|:1049510-1050076
12	7	nr0953	ref|NC_002745.2|:1050088-1051569
12	8	nr0954	ref|NC_002745.2|:1051699-1052838
12	9	nr0955	ref|NC_002745.2|:c1053912-1053106
12	10	nr0956	ref|NC_002745.2|:c1055305-1053905
12	11	nr0957	ref|NC_002745.2|:c1055895-1055320

12	12	nr0958	ref|NC_002745.2|:1056358-1056567
12	13	nr0959	ref|NC_002745.2|:c1056865-1056731
12	14	nr0960	ref|NC_002745.2|:1057017-1058312
12	15	nr0961	ref|NC_002745.2|:1058737-1059909
12	16	nr0962	ref|NC_002745.2|:1059963-1060505
12	17	nr0963	ref|NC_002745.2|:1060659-1060925
12	18	nr0964	ref|NC_002745.2|:1060928-1062646
12	19	nr0965	ref|NC_002745.2|:c1063116-1062883
12	20	nr0966	ref|NC_002745.2|:1063314-1064675
12	21	nr0967	ref|NC_002745.2|:1064672-1065691
12	22	nr0968	ref|NC_002745.2|:1065824-1066486
12	23	nr0969	ref|NC_002745.2|:c1068429-1066732
12	24	nr0970	ref|NC_002745.2|:c1068599-1068429
12	25	nr0971	ref|NC_002745.2|:c1069680-1069129
12	26	nr0972	ref|NC_002745.2|:1070045-1070671
12	27	nr0973	ref|NC_002745.2|:1070842-1071954
12	28	nr0974	ref|NC_002745.2|:1071958-1072935
12	29	nr0975	ref|NC_002745.2|:1073026-1074318
12	30	nr0976	ref|NC_002745.2|:1074322-1075728
12	31	nr0977	ref|NC_002745.2|:1075896-1076171
12	32	nr0978	ref|NC_002745.2|:1076315-1076854
12	33	nr0979	ref|NC_002745.2|:1076867-1077961
12	34	nr0980	ref|NC_002745.2|:1077954-1078751
12	35	nr0981	ref|NC_002745.2|:1078757-1079566
12	36	nr0982	ref|NC_002745.2|:1079566-1080639
12	37	nr0983	ref|NC_002745.2|:1080713-1081732
12	38	nr0984	ref|NC_002745.2|:1082015-1082434
12	39	nr0985	ref|NC_002745.2|:c1083865-1082513
12	40	nr0986	ref|NC_002745.2|:c1084663-1084049
12	41	nr0987	ref|NC_002745.2|:1084820-1085647
12	42	nr0988	ref|NC_002745.2|:c1085992-1085801
12	43	nr0989	ref|NC_002745.2|:1086094-1087941
12	44	nr0990	ref|NC_002745.2|:c1088248-1088081
12	45	nr0991	ref|NC_002745.2|:c1088732-1088250
12	46	nr0992	ref|NC_002745.2|:1088873-1089148
12	47	nr0993	ref|NC_002745.2|:1089567-1090688
12	48	nr0994	ref|NC_002745.2|:1091242-1094694
12	49	nr0995	ref|NC_002745.2|:c1095745-1094834
12	50	nr0996	ref|NC_002745.2|:1096182-1097096
12	51	nr0997	ref|NC_002745.2|:1097121-1097582
12	52	nr0998	ref|NC_002745.2|:1097909-1098946
12	53	nr0999	ref|NC_002745.2|:1098962-1099396
12	54	nr1000	ref|NC_002745.2|:c1100385-1099459
12	55	nr1001	ref|NC_002745.2|:1100624-1100878
12	56	nr1002	ref|NC_002745.2|:c1101270-1100881
12	57	nr1003	ref|NC_002745.2|:1101340-1101882
12	58	nr1004	ref|NC_002745.2|:1101884-1102366
12	59	nr1005	ref|NC_002745.2|:c1103567-1102428
12	60	nr1006	ref|NC_002745.2|:1103694-1104251

12	61	nr1007	ref|NC_002745.2|:1104331-1104504
12	62	nr1008	ref|NC_002745.2|:c1106595-1104658
12	63	nr1009	ref|NC_002745.2|:c1107850-1106798
12	64	nr1010	ref|NC_002745.2|:1108059-1108742
12	65	nr1011	ref|NC_002745.2|:1108742-1109818
12	66	nr1012	ref|NC_002745.2|:1109815-1110693
12	67	nr1013	ref|NC_002745.2|:1110850-1111671
12	68	nr1014	ref|NC_002745.2|:1111733-1112467
12	69	nr1015	ref|NC_002745.2|:1112495-1112809
12	70	nr1016	ref|NC_002745.2|:1113193-1113933
12	71	nr1017	ref|NC_002745.2|:1114313-1115371
12	72	nr1018	ref|NC_002745.2|:1115371-1117773
12	73	nr1019	ref|NC_002745.2|:c1119143-1118205
12	74	nr1020	ref|NC_002745.2|:1119513-1119779
12	75	nr1021	ref|NC_002745.2|:1119780-1120301
12	76	nr1022	ref|NC_002745.2|:1120374-1122086
12	77	nr1023	ref|NC_002745.2|:1122096-1124444
12	78	nr1024	ref|NC_002745.2|:1124617-1124931
12	79	nr1025	ref|NC_002745.2|:1125255-1127036
12	80	nr1026	ref|NC_002745.2|:1127360-1127974
12	81	nr1027	ref|NC_002745.2|:1128026-1129792
12	82	nr1028	ref|NC_002745.2|:1129792-1130607
12	83	nr1029	ref|NC_002745.2|:1130843-1131643
12	84	nr1030	ref|NC_002745.2|:1131655-1132242
12	85	nr1031	ref|NC_002745.2|:1132235-1132738
12	86	nr1032	ref|NC_002745.2|:1132896-1133033
13	1	nr1033	ref|NC_002745.2|:1133227-1133556
13	2	nr1034	ref|NC_002745.2|:c1134326-1133925
13	3	nr1035	ref|NC_002745.2|:1134996-1135502
13	4	nr1036	ref|NC_002745.2|:1135762-1136259
13	5	nr1037	ref|NC_002745.2|:1136409-1136759
13	6	nr1038	ref|NC_002745.2|:c1137710-1137525
13	7	nr1039	ref|NC_002745.2|:c1138565-1138332
13	8	nr1040	ref|NC_002745.2|:1139084-1140403
13	9	nr1041	ref|NC_002745.2|:c1141521-1140562
13	10	nr1042	ref|NC_002745.2|:1142324-1142521
13	11	nr1043	ref|NC_002745.2|:c1142879-1142748
13	12	nr1044	ref|NC_002745.2|:c1143727-1143011
13	13	nr1045	ref|NC_002745.2|:c1144560-1143835
13	14	nr1046	ref|NC_002745.2|:c1145380-1144655
13	15	nr1047	ref|NC_002745.2|:1145818-1146819
13	16	nr1048	ref|NC_002745.2|:1146842-1147774
13	17	nr1049	ref|NC_002745.2|:1147946-1149502
13	18	nr1050	ref|NC_002745.2|:1149809-1150036
13	19	nr1051	ref|NC_002745.2|:c1151289-1150342
13	20	nr1052	ref|NC_002745.2|:1151538-1151726
13	21	nr1053	ref|NC_002745.2|:1153089-1153775
13	22	nr1054	ref|NC_002745.2|:c1154324-1153884
13	23	nr1055	ref|NC_002745.2|:1154519-1156132

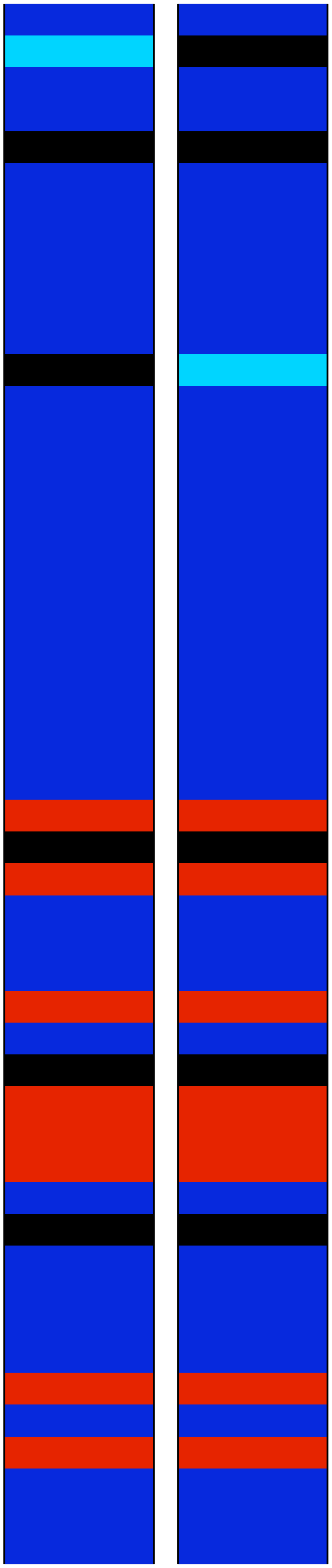

13	24	nr1056	ref|NC_002745.2|:1156276-1156707
13	25	nr1057	ref|NC_002745.2|:1156723-1157658
13	26	nr1058	ref|NC_002745.2|:1157672-1158073
13	27	nr1059	ref|NC_002745.2|:1158054-1160288
13	28	nr1060	ref|NC_002745.2|:1160580-1161545
13	29	nr1061	ref|NC_002745.2|:1161547-1162896
13	30	nr1062	ref|NC_002745.2|:1162912-1164231
13	31	nr1063	ref|NC_002745.2|:1164337-1165749
13	32	nr1064	ref|NC_002745.2|:1165782-1166954
13	33	nr1065	ref|NC_002745.2|:1167214-1168005
13	34	nr1066	ref|NC_002745.2|:1168023-1168697
13	35	nr1067	ref|NC_002745.2|:1168694-1169257
13	36	nr1068	ref|NC_002745.2|:1169269-1169559
13	37	nr1069	ref|NC_002745.2|:1169810-1170448
13	38	nr1070	ref|NC_002745.2|:1170472-1171089
13	39	nr1071	ref|NC_002745.2|:1171310-1174063
13	40	nr1072	ref|NC_002745.2|:1174354-1175151
13	41	nr1073	ref|NC_002745.2|:c1175424-1175212
13	42	nr1074	ref|NC_002745.2|:1175975-1176466
13	43	nr1075	ref|NC_002745.2|:1176466-1177383
13	44	nr1076	ref|NC_002745.2|:1177783-1178310
13	45	nr1077	ref|NC_002745.2|:1178528-1179835
13	46	nr1078	ref|NC_002745.2|:1179863-1180744
13	47	nr1079	ref|NC_002745.2|:1180762-1182036
13	48	nr1080	ref|NC_002745.2|:1182038-1183138
13	49	nr1081	ref|NC_002745.2|:1183131-1186304
13	50	nr1082	ref|NC_002745.2|:1186414-1187106
13	51	nr1083	ref|NC_002745.2|:1187106-1187717
13	52	nr1084	ref|NC_002745.2|:1187747-1187959
13	53	nr1085	ref|NC_002745.2|:1188396-1188797
13	54	nr1086	ref|NC_002745.2|:c1190757-1189060
13	55	nr1087	ref|NC_002745.2|:1191032-1191655
13	56	nr1088	ref|NC_002745.2|:1191655-1191873
13	57	nr1089	ref|NC_002745.2|:1192089-1193288
13	58	nr1090	ref|NC_002745.2|:1193288-1195696
13	59	nr1091	ref|NC_002745.2|:1196202-1197155
13	60	nr1092	ref|NC_002745.2|:c1197521-1197318
13	61	nr1093	ref|NC_002745.2|:1197820-1198308
13	62	nr1094	ref|NC_002745.2|:1198301-1199236
13	63	nr1095	ref|NC_002745.2|:1199155-1200540
13	64	nr1096	ref|NC_002745.2|:1200543-1201637
13	65	nr1097	ref|NC_002745.2|:1201644-1202387
13	66	nr1098	ref|NC_002745.2|:1202384-1204378
13	67	nr1099	ref|NC_002745.2|:1204606-1205481
13	68	nr1100	ref|NC_002745.2|:1205482-1206126
13	69	nr1101	ref|NC_002745.2|:1206133-1206774
13	70	nr1102	ref|NC_002745.2|:c1207343-1207155
13	71	nr1103	ref|NC_002745.2|:1207786-1208160
13	72	nr1104	ref|NC_002745.2|:1208175-1209821

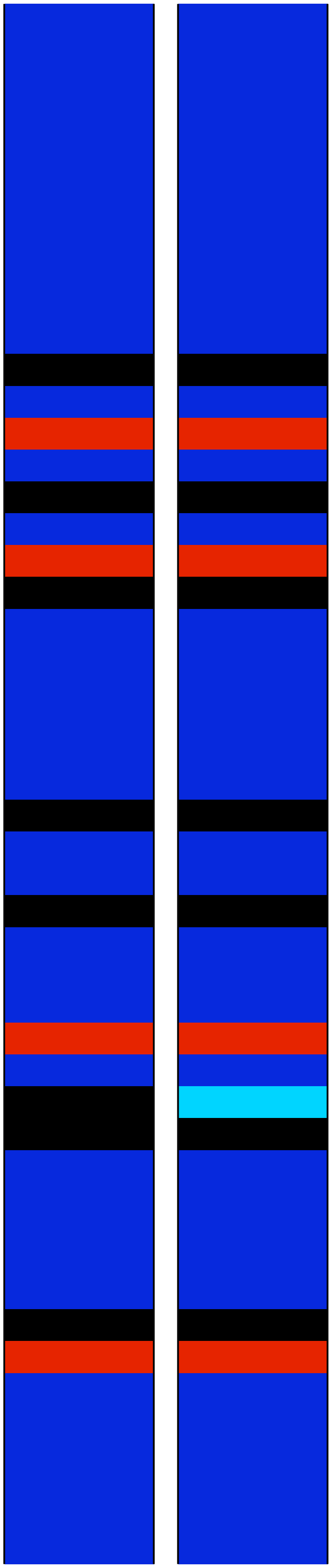

13	73	nr1105	ref|NC_002745.2|:1210011-1212071
13	74	nr1106	ref|NC_002745.2|:1212289-1212846
13	75	nr1107	ref|NC_002745.2|:1212851-1213837
13	76	nr1108	ref|NC_002745.2|:1213830-1214756
13	77	nr1109	ref|NC_002745.2|:1214743-1215483
13	78	nr1110	ref|NC_002745.2|:1215918-1216151
13	79	nr1111	ref|NC_002745.2|:1216267-1216998
13	80	nr1112	ref|NC_002745.2|:1217145-1220711
13	81	nr1113	ref|NC_002745.2|:1220711-1221961
13	82	nr1114	ref|NC_002745.2|:1221948-1222280
13	83	nr1115	ref|NC_002745.2|:1222306-1223673
13	84	nr1116	ref|NC_002745.2|:1224108-1224383
13	85	nr1117	ref|NC_002745.2|:1224571-1225074
13	86	nr1118	ref|NC_002745.2|:1225074-1225811
14	1	nr1119	ref|NC_002745.2|:1225914-1226264
14	2	nr1120	ref|NC_002745.2|:c1229114-1226508
14	3	nr1121	ref|NC_002745.2|:1229515-1230399
14	4	nr1122	ref|NC_002745.2|:1230383-1231150
14	5	nr1123	ref|NC_002745.2|:1231259-1232425
14	6	nr1124	ref|NC_002745.2|:1232447-1233355
14	7	nr1125	ref|NC_002745.2|:1233582-1234700
14	8	nr1126	ref|NC_002745.2|:1234728-1235972
14	9	nr1127	ref|NC_002745.2|:1236145-1237017
14	10	nr1128	ref|NC_002745.2|:1237197-1239266
14	11	nr1129	ref|NC_002745.2|:1239422-1240729
14	12	nr1130	ref|NC_002745.2|:1241147-1242043
14	13	nr1131	ref|NC_002745.2|:1242040-1242585
14	14	nr1132	ref|NC_002745.2|:1242651-1244054
14	15	nr1133	ref|NC_002745.2|:1244079-1244852
14	16	nr1134	ref|NC_002745.2|:1245199-1245966
14	17	nr1135	ref|NC_002745.2|:1246148-1247029
14	18	nr1136	ref|NC_002745.2|:1247166-1247888
14	19	nr1137	ref|NC_002745.2|:1247907-1248461
14	20	nr1138	ref|NC_002745.2|:1248834-1249604
14	21	nr1139	ref|NC_002745.2|:1249611-1250393
14	22	nr1140	ref|NC_002745.2|:1250605-1251891
14	23	nr1141	ref|NC_002745.2|:1251911-1253614
14	24	nr1142	ref|NC_002745.2|:1253872-1258188
14	25	nr1143	ref|NC_002745.2|:1258478-1258945
14	26	nr1144	ref|NC_002745.2|:1258966-1260141
14	27	nr1145	ref|NC_002745.2|:1260162-1260446
14	28	nr1146	ref|NC_002745.2|:1260443-1260760
14	29	nr1147	ref|NC_002745.2|:1260765-1262882
14	30	nr1148	ref|NC_002745.2|:1263268-1263618
14	31	nr1149	ref|NC_002745.2|:1263787-1264704
14	32	nr1150	ref|NC_002745.2|:1264719-1265690
14	33	nr1151	ref|NC_002745.2|:1265805-1266074
14	34	nr1152	ref|NC_002745.2|:1266443-1268539
14	35	nr1153	ref|NC_002745.2|:1268775-1270448

14	36	nr1154	ref|NC_002745.2|:1270705-1273071
14	37	nr1155	ref|NC_002745.2|:1273076-1273789
14	38	nr1156	ref|NC_002745.2|:1273820-1275085
14	39	nr1157	ref|NC_002745.2|:1275085-1276371
14	40	nr1158	ref|NC_002745.2|:1276371-1277075
14	41	nr1159	ref|NC_002745.2|:1277180-1278007
14	42	nr1160	ref|NC_002745.2|:1278026-1278418
14	43	nr1161	ref|NC_002745.2|:1278452-1279030
14	44	nr1162	ref|NC_002745.2|:1279254-1280405
14	45	nr1163	ref|NC_002745.2|:1280570-1281613
14	46	nr1164	ref|NC_002745.2|:1281967-1283526
14	47	nr1165	ref|NC_002745.2|:c1283993-1283823
14	48	nr1166	ref|NC_002745.2|:1284212-1285009
14	49	nr1167	ref|NC_002745.2|:1285149-1286909
14	50	nr1168	ref|NC_002745.2|:1286910-1287776
14	51	nr1169	ref|NC_002745.2|:1287870-1288163
14	52	nr1170	ref|NC_002745.2|:1288297-1289841
14	53	nr1171	ref|NC_002745.2|:1289842-1290207
14	54	nr1172	ref|NC_002745.2|:1290234-1290725
14	55	nr1173	ref|NC_002745.2|:1291028-1293646
14	56	nr1174	ref|NC_002745.2|:1293659-1295668
14	57	nr1175	ref|NC_002745.2|:1295683-1296216
14	58	nr1176	ref|NC_002745.2|:c1296342-1296253
14	59	nr1177	ref|NC_002745.2|:1296691-1297509
14	60	nr1178	ref|NC_002745.2|:1297638-1299134
14	61	nr1179	ref|NC_002745.2|:1299244-1300965
14	62	nr1180	ref|NC_002745.2|:1301115-1302029
14	63	nr1181	ref|NC_002745.2|:1302047-1302982
14	64	nr1182	ref|NC_002745.2|:1302997-1303230
14	65	nr1183	ref|NC_002745.2|:c1303928-1303452
14	66	nr1184	ref|NC_002745.2|:1304040-1305278
14	67	nr1185	ref|NC_002745.2|:1305297-1306535
14	68	nr1186	ref|NC_002745.2|:1306778-1307146
14	69	nr1187	ref|NC_002745.2|:1307165-1308505
14	70	nr1188	ref|NC_002745.2|:1310400-1310606
14	71	nr1189	ref|NC_002745.2|:1311840-1312025
14	72	nr1190	ref|NC_002745.2|:1312768-1313019
14	73	nr1191	ref|NC_002745.2|:1313742-1313936
14	74	nr1192	ref|NC_002745.2|:c1315505-1314480
14	75	nr1193	ref|NC_002745.2|:1315775-1315972
14	76	nr1194	ref|NC_002745.2|:1315993-1317510
14	77	nr1195	ref|NC_002745.2|:1317694-1318593
14	78	nr1196	ref|NC_002745.2|:1318562-1319293
14	79	nr1197	ref|NC_002745.2|:1319297-1320388
14	80	nr1198	ref|NC_002745.2|:1320385-1320987
14	81	nr1199	ref|NC_002745.2|:c1321288-1321100
14	82	nr1200	ref|NC_002745.2|:1321427-1321960
14	83	nr1201	ref|NC_002745.2|:c1322959-1322105
14	84	nr1202	ref|NC_002745.2|:1323275-1323865

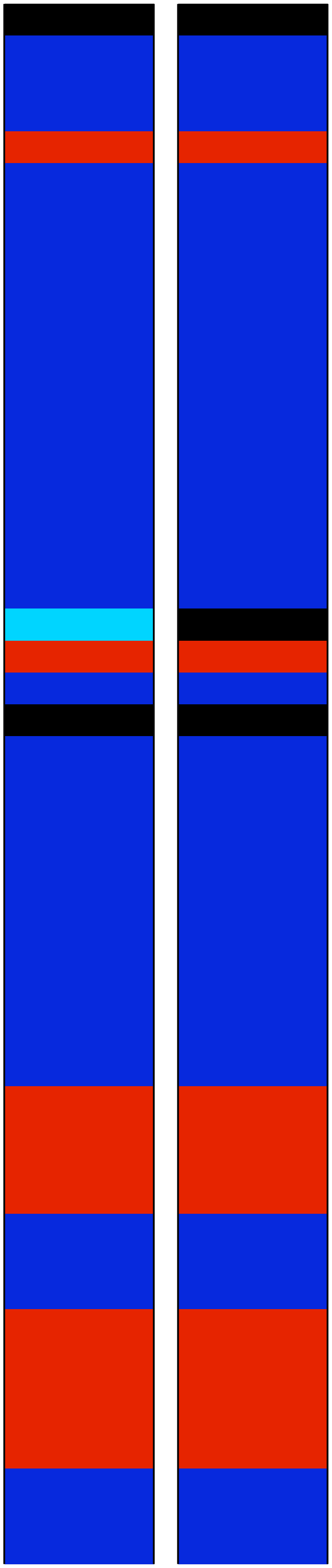

14	85	nr1203	ref|NC_002745.2|:c1325301-1323919
14	86	nr1204	ref|NC_002745.2|:1325492-1326772
15	1	nr1205	ref|NC_002745.2|:1326778-1327839
15	2	nr1206	ref|NC_002745.2|:1327841-1328755
15	3	nr1207	ref|NC_002745.2|:1328813-1329616
15	4	nr1208	ref|NC_002745.2|:c1330223-1329909
15	5	nr1209	ref|NC_002745.2|:c1331895-1330441
15	6	nr1210	ref|NC_002745.2|:1332093-1333616
15	7	nr1211	ref|NC_002745.2|:1333707-1333856
15	8	nr1212	ref|NC_002745.2|:1334310-1334579
15	9	nr1213	ref|NC_002745.2|:1334736-1335713
15	10	nr1214	ref|NC_002745.2|:1335735-1336751
15	11	nr1215	ref|NC_002745.2|:c1337754-1337131
15	12	nr1216	ref|NC_002745.2|:1337897-1338130
15	13	nr1217	ref|NC_002745.2|:1338267-1338506
15	14	nr1218	ref|NC_002745.2|:1338627-1340615
15	15	nr1219	ref|NC_002745.2|:1340893-1341135
15	16	nr1220	ref|NC_002745.2|:1341314-1341781
15	17	nr1221	ref|NC_002745.2|:1341905-1343026
15	18	nr1222	ref|NC_002745.2|:1343030-1346059
15	19	nr1223	ref|NC_002745.2|:c1346492-1346130
15	20	nr1224	ref|NC_002745.2|:1346696-1348342
15	21	nr1225	ref|NC_002745.2|:1349289-1351994
15	22	nr1226	ref|NC_002745.2|:1352174-1352641
15	23	nr1227	ref|NC_002745.2|:c1353144-1352848
15	24	nr1228	ref|NC_002745.2|:c1354128-1353520
15	25	nr1229	ref|NC_002745.2|:1354328-1356325
15	26	nr1230	ref|NC_002745.2|:1356325-1358727
15	27	nr1231	ref|NC_002745.2|:1358977-1360437
15	28	nr1232	ref|NC_002745.2|:1360937-1361788
15	29	nr1233	ref|NC_002745.2|:1361835-1361930
15	30	nr1234	ref|NC_002745.2|:1361923-1363131
15	31	nr1235	ref|NC_002745.2|:1363612-1366134
15	32	nr1236	ref|NC_002745.2|:c1366846-1366337
15	33	nr1237	ref|NC_002745.2|:1366983-1367966
15	34	nr1238	ref|NC_002745.2|:c1368242-1368054
15	35	nr1239	ref|NC_002745.2|:1368387-1369649
15	36	nr1240	ref|NC_002745.2|:c1370877-1369786
15	37	nr1241	ref|NC_002745.2|:1371042-1372073
15	38	nr1242	ref|NC_002745.2|:1372565-1373971
15	39	nr1243	ref|NC_002745.2|:1373968-1374534
15	40	nr1244	ref|NC_002745.2|:1374540-1375538
15	41	nr1245	ref|NC_002745.2|:1375540-1376322
15	42	nr1246	ref|NC_002745.2|:1376322-1376954
15	43	nr1247	ref|NC_002745.2|:1376947-1378161
15	44	nr1248	ref|NC_002745.2|:1378154-1378882
15	45	nr1249	ref|NC_002745.2|:1379204-1380466
15	46	nr1250	ref|NC_002745.2|:1380485-1381744
15	47	nr1251	ref|NC_002745.2|:1382033-1382779

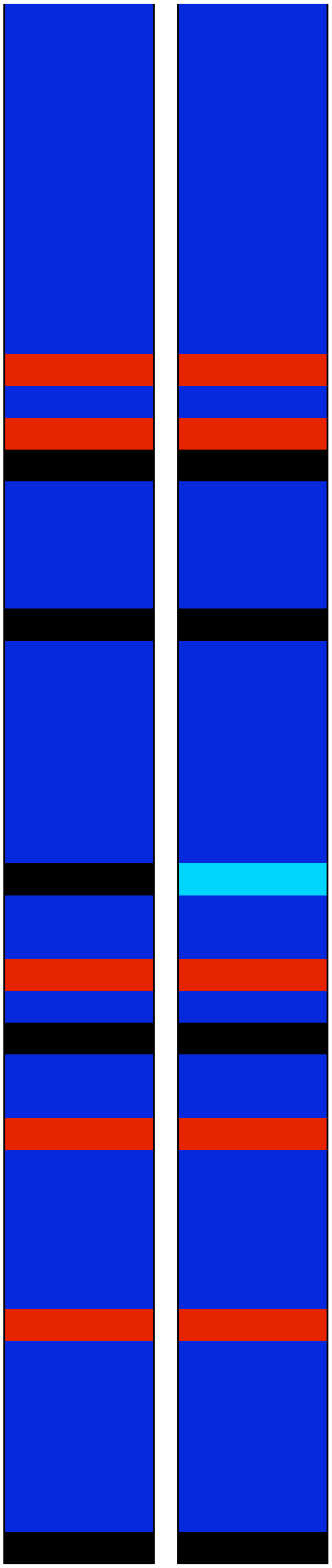

15	48	nr1252	ref|NC_002745.2|:1383009-1383776
15	49	nr1253	ref|NC_002745.2|:c1384297-1383824
15	50	nr1254	ref|NC_002745.2|:c1385122-1384421
15	51	nr1255	ref|NC_002745.2|:c1385888-1385115
15	52	nr1256	ref|NC_002745.2|:c1386705-1385875
15	53	nr1257	ref|NC_002745.2|:c1387684-1386698
15	54	nr1258	ref|NC_002745.2|:c1388332-1387988
15	55	nr1259	ref|NC_002745.2|:1388537-1390351
15	56	nr1260	ref|NC_002745.2|:c1391132-1390491
15	57	nr1261	ref|NC_002745.2|:c1391990-1391139
15	58	nr1262	ref|NC_002745.2|:c1392954-1392037
15	59	nr1263	ref|NC_002745.2|:c1393882-1392956
15	60	nr1264	ref|NC_002745.2|:c1395056-1394073
15	61	nr1265	ref|NC_002745.2|:1395599-1395895
15	62	nr1266	ref|NC_002745.2|:c1396933-1396031
15	63	nr1267	ref|NC_002745.2|:1397080-1398681
15	64	nr1268	ref|NC_002745.2|:1399743-1400948
15	65	nr1269	ref|NC_002745.2|:1401012-1402001
15	66	nr1270	ref|NC_002745.2|:1402003-1402890
15	67	nr1271	ref|NC_002745.2|:1402887-1403609
15	68	nr1272	ref|NC_002745.2|:1403636-1404355
15	69	nr1273	ref|NC_002745.2|:1404498-1405649
15	70	nr1274	ref|NC_002745.2|:1405654-1406739
15	71	nr1275	ref|NC_002745.2|:1406729-1407994
15	72	nr1276	ref|NC_002745.2|:c1408635-1408234
15	73	nr1277	ref|NC_002745.2|:c1409032-1408832
15	74	nr1278	ref|NC_002745.2|:c1409511-1409203
15	75	nr1279	ref|NC_002745.2|:1409674-1409943
15	76	nr1280	ref|NC_002745.2|:1409968-1410591
15	77	nr1281	ref|NC_002745.2|:1410623-1411759
15	78	nr1282	ref|NC_002745.2|:c1413175-1411832
15	79	nr1283	ref|NC_002745.2|:c1415287-1413401
15	80	nr1284	ref|NC_002745.2|:c1416092-1415301
15	81	nr1285	ref|NC_002745.2|:c1416476-1416273
15	82	nr1286	ref|NC_002745.2|:c1417314-1416505
15	83	nr1287	ref|NC_002745.2|:c1419162-1417894
15	84	nr1288	ref|NC_002745.2|:c1421908-1419176
15	85	nr1289	ref|NC_002745.2|:c1423547-1422192
15	86	nr1290	ref|NC_002745.2|:c1423789-1423544
16	1	nr1291	ref|NC_002745.2|:c1424187-1423786
16	2	nr1292	ref|NC_002745.2|:c1424858-1424658
16	3	nr1293	ref|NC_002745.2|:c1425589-1424975
16	4	nr1294	ref|NC_002745.2|:c1426676-1425606
16	5	nr1295	ref|NC_002745.2|:c1427197-1426688
16	6	nr1296	ref|NC_002745.2|:c1429087-1427597
16	7	nr1297	ref|NC_002745.2|:c1429500-1429279
16	8	nr1298	ref|NC_002745.2|:c1430000-1429500
16	9	nr1299	ref|NC_002745.2|:c1430440-1430012
16	10	nr1300	ref|NC_002745.2|:c1430966-1430433

16	11	nr1301	ref|NC_002745.2|:c1431891-1431052
16	12	nr1302	ref|NC_002745.2|:c1432385-1431906
16	13	nr1303	ref|NC_002745.2|:c1433541-1432585
16	14	nr1304	ref|NC_002745.2|:c1434402-1433965
16	15	nr1305	ref|NC_002745.2|:c1435542-1434418
16	16	nr1306	ref|NC_002745.2|:c1435831-1435580
16	17	nr1307	ref|NC_002745.2|:c1436028-1435843
16	18	nr1308	ref|NC_002745.2|:1436282-1436986
16	19	nr1309	ref|NC_002745.2|:1437468-1437869
16	20	nr1310	ref|NC_002745.2|:c1458069-1437928
16	21	nr1311	ref|NC_002745.2|:c1469802-1458130
16	22	nr1312	ref|NC_002745.2|:c1471591-1470200
16	23	nr1313	ref|NC_002745.2|:c1473069-1471747
16	24	nr1314	ref|NC_002745.2|:c1474140-1473100
16	25	nr1315	ref|NC_002745.2|:c1475353-1474235
16	26	nr1316	ref|NC_002745.2|:c1476707-1475829
16	27	nr1317	ref|NC_002745.2|:c1480167-1476727
16	28	nr1318	ref|NC_002745.2|:1480471-1481811
16	29	nr1319	ref|NC_002745.2|:c1482349-1482017
16	30	nr1320	ref|NC_002745.2|:c1483578-1482433
16	31	nr1321	ref|NC_002745.2|:c1484128-1484012
16	32	nr1322	ref|NC_002745.2|:c1484573-1484229
16	33	nr1323	ref|NC_002745.2|:c1485150-1484587
16	34	nr1324	ref|NC_002745.2|:c1485493-1485143
16	35	nr1325	ref|NC_002745.2|:1486033-1486659
16	36	nr1326	ref|NC_002745.2|:1486656-1488839
16	37	nr1327	ref|NC_002745.2|:c1489746-1489405
16	38	nr1328	ref|NC_002745.2|:c1490410-1489751
16	39	nr1329	ref|NC_002745.2|:c1491086-1490400
16	40	nr1330	ref|NC_002745.2|:c1492706-1491414
16	41	nr1331	ref|NC_002745.2|:c1495721-1493028
16	42	nr1332	ref|NC_002745.2|:c1496716-1495745
16	43	nr1333	ref|NC_002745.2|:c1497905-1496703
16	44	nr1334	ref|NC_002745.2|:c1499052-1497910
16	45	nr1335	ref|NC_002745.2|:c1499613-1499296
16	46	nr1336	ref|NC_002745.2|:c1500647-1499949
16	47	nr1337	ref|NC_002745.2|:c1501288-1500701
16	48	nr1338	ref|NC_002745.2|:c1501853-1501278
16	49	nr1339	ref|NC_002745.2|:c1503111-1501867
16	50	nr1340	ref|NC_002745.2|:c1504416-1503118
16	51	nr1341	ref|NC_002745.2|:c1505490-1504426
16	52	nr1342	ref|NC_002745.2|:c1506682-1505516
16	53	nr1343	ref|NC_002745.2|:c1507268-1507068
16	54	nr1344	ref|NC_002745.2|:c1507422-1507297
16	55	nr1345	ref|NC_002745.2|:c1507926-1507477
16	56	nr1346	ref|NC_002745.2|:c1508977-1508018
16	57	nr1347	ref|NC_002745.2|:c1509704-1508979
16	58	nr1348	ref|NC_002745.2|:c1510279-1509707
16	59	nr1349	ref|NC_002745.2|:c1510982-1510710

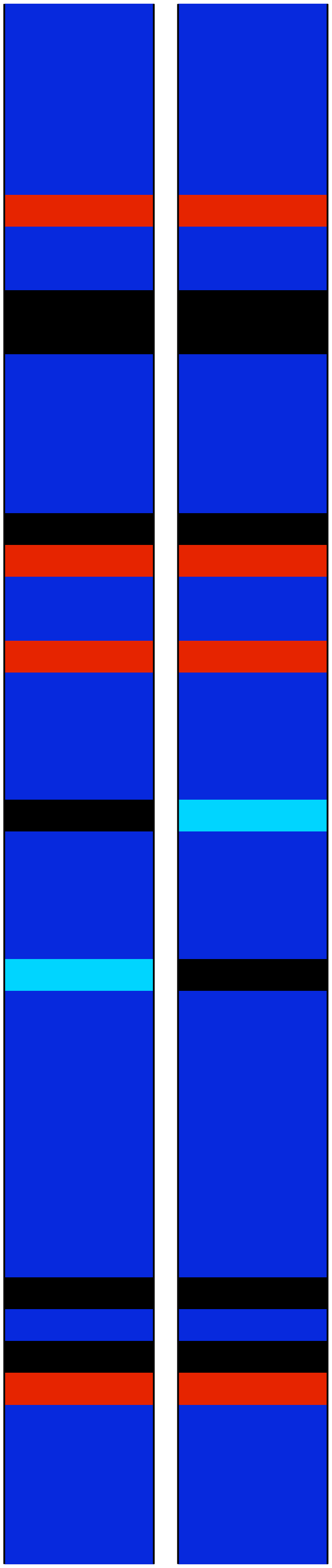

16	60	nr1350	ref|NC_002745.2|:c1512151-1511153
16	61	nr1351	ref|NC_002745.2|:c1513478-1512168
16	62	nr1352	ref|NC_002745.2|:c1514875-1513700
16	63	nr1353	ref|NC_002745.2|:c1515481-1515404
16	64	nr1354	ref|NC_002745.2|:c1516246-1515587
16	65	nr1355	ref|NC_002745.2|:1516323-1517291
16	66	nr1356	ref|NC_002745.2|:c1518392-1517406
16	67	nr1357	ref|NC_002745.2|:c1520254-1518794
16	68	nr1358	ref|NC_002745.2|:c1521786-1520407
16	69	nr1359	ref|NC_002745.2|:c1522729-1521776
16	70	nr1360	ref|NC_002745.2|:1522837-1523085
16	71	nr1361	ref|NC_002745.2|:c1523736-1523191
16	72	nr1362	ref|NC_002745.2|:c1525094-1524138
16	73	nr1363	ref|NC_002745.2|:c1526461-1525565
16	74	nr1364	ref|NC_002745.2|:c1527424-1526519
16	75	nr1365	ref|NC_002745.2|:c1529676-1527514
16	76	nr1366	ref|NC_002745.2|:c1530535-1529669
16	77	nr1367	ref|NC_002745.2|:c1532605-1530839
16	78	nr1368	ref|NC_002745.2|:c1533296-1532571
16	79	nr1369	ref|NC_002745.2|:c1534166-1533429
16	80	nr1370	ref|NC_002745.2|:c1534701-1534159
16	81	nr1371	ref|NC_002745.2|:c1535449-1534694
16	82	nr1372	ref|NC_002745.2|:1535517-1536023
16	83	nr1373	ref|NC_002745.2|:c1536988-1536101
16	84	nr1374	ref|NC_002745.2|:c1537486-1537037
16	85	nr1375	ref|NC_002745.2|:c1538133-1537591
16	86	nr1376	ref|NC_002745.2|:1538215-1539123
17	1	nr1377	ref|NC_002745.2|:c1539587-1539339
17	2	nr1378	ref|NC_002745.2|:c1540358-1539603
17	3	nr1379	ref|NC_002745.2|:1540500-1541315
17	4	nr1380	ref|NC_002745.2|:c1542338-1541418
17	5	nr1381	ref|NC_002745.2|:1542651-1544135
17	6	nr1382	ref|NC_002745.2|:1544365-1545231
17	7	nr1383	ref|NC_002745.2|:c1546962-1545313
17	8	nr1384	ref|NC_002745.2|:c1547997-1546978
17	9	nr1385	ref|NC_002745.2|:c1548562-1548191
17	10	nr1386	ref|NC_002745.2|:c1550246-1549029
17	11	nr1387	ref|NC_002745.2|:c1551968-1550562
17	12	nr1388	ref|NC_002745.2|:c1553169-1552036
17	13	nr1389	ref|NC_002745.2|:c1554718-1553738
17	14	nr1390	ref|NC_002745.2|:c1555169-1554732
17	15	nr1391	ref|NC_002745.2|:c1556794-1555520
17	16	nr1392	ref|NC_002745.2|:c1557790-1556807
17	17	nr1393	ref|NC_002745.2|:c1558782-1557790
17	18	nr1394	ref|NC_002745.2|:c1560219-1558798
17	19	nr1395	ref|NC_002745.2|:c1562049-1560370
17	20	nr1396	ref|NC_002745.2|:c1562517-1562065
17	21	nr1397	ref|NC_002745.2|:c1563830-1562949
17	22	nr1398	ref|NC_002745.2|:c1564038-1563808

17	23	nr1399	ref|NC_002745.2|:c1565368-1564031
17	24	nr1400	ref|NC_002745.2|:c1565774-1565385
17	25	nr1401	ref|NC_002745.2|:c1566196-1565834
17	26	nr1402	ref|NC_002745.2|:c1567566-1566211
17	27	nr1403	ref|NC_002745.2|:c1568030-1567566
17	28	nr1404	ref|NC_002745.2|:c1569061-1568504
17	29	nr1405	ref|NC_002745.2|:c1570148-1569087
17	30	nr1406	ref|NC_002745.2|:1570253-1570834
17	31	nr1407	ref|NC_002745.2|:1570848-1571066
17	32	nr1408	ref|NC_002745.2|:c1571960-1571130
17	33	nr1409	ref|NC_002745.2|:1572118-1572504
17	34	nr1410	ref|NC_002745.2|:c1574341-1572869
17	35	nr1411	ref|NC_002745.2|:c1575680-1574334
17	36	nr1412	ref|NC_002745.2|:c1576791-1575700
17	37	nr1413	ref|NC_002745.2|:c1577474-1576950
17	38	nr1414	ref|NC_002745.2|:c1578204-1577707
17	39	nr1415	ref|NC_002745.2|:c1578421-1578122
17	40	nr1416	ref|NC_002745.2|:c1578842-1578408
17	41	nr1417	ref|NC_002745.2|:c1579143-1578832
17	42	nr1418	ref|NC_002745.2|:c1580227-1579157
17	43	nr1419	ref|NC_002745.2|:c1581173-1580199
17	44	nr1420	ref|NC_002745.2|:c1581848-1581225
17	45	nr1421	ref|NC_002745.2|:c1582174-1581845
17	46	nr1422	ref|NC_002745.2|:c1583160-1582174
17	47	nr1423	ref|NC_002745.2|:c1583360-1583157
17	48	nr1424	ref|NC_002745.2|:c1584804-1583341
17	49	nr1425	ref|NC_002745.2|:c1585355-1584816
17	50	nr1426	ref|NC_002745.2|:c1585713-1585564
17	51	nr1427	ref|NC_002745.2|:c1587901-1585826
17	52	nr1428	ref|NC_002745.2|:c1588621-1588022
17	53	nr1429	ref|NC_002745.2|:c1589307-1588897
17	54	nr1430	ref|NC_002745.2|:c1590157-1589294
17	55	nr1431	ref|NC_002745.2|:c1590966-1590199
17	56	nr1432	ref|NC_002745.2|:c1592000-1591110
17	57	nr1433	ref|NC_002745.2|:c1593356-1592010
17	58	nr1434	ref|NC_002745.2|:c1594570-1593470
17	59	nr1435	ref|NC_002745.2|:c1595250-1594573
17	60	nr1436	ref|NC_002745.2|:c1596487-1595381
17	61	nr1437	ref|NC_002745.2|:c1598528-1596711
17	62	nr1438	ref|NC_002745.2|:c1599407-1598589
17	63	nr1439	ref|NC_002745.2|:c1600041-1599418
17	64	nr1440	ref|NC_002745.2|:1600376-1601767
17	65	nr1441	ref|NC_002745.2|:c1602669-1601917
17	66	nr1442	ref|NC_002745.2|:c1603590-1602691
17	67	nr1443	ref|NC_002745.2|:c1603995-1603591
17	68	nr1444	ref|NC_002745.2|:c1604350-1604006
17	69	nr1445	ref|NC_002745.2|:c1604820-1604353
17	70	nr1446	ref|NC_002745.2|:c1605768-1604821
17	71	nr1447	ref|NC_002745.2|:c1606770-1606072

17	72	nr1448	ref|NC_002745.2|:c1607776-1606787
17	73	nr1449	ref|NC_002745.2|:c1608501-1607794
17	74	nr1450	ref|NC_002745.2|:c1608897-1608721
17	75	nr1451	ref|NC_002745.2|:c1610536-1609190
17	76	nr1452	ref|NC_002745.2|:c1611295-1610543
17	77	nr1453	ref|NC_002745.2|:c1612235-1611297
17	78	nr1454	ref|NC_002745.2|:c1613378-1612239
17	79	nr1455	ref|NC_002745.2|:c1615346-1613514
17	80	nr1456	ref|NC_002745.2|:c1616041-1615415
17	81	nr1457	ref|NC_002745.2|:c1617050-1616073
17	82	nr1458	ref|NC_002745.2|:c1618275-1617151
17	83	nr1459	ref|NC_002745.2|:1618552-1618710
17	84	nr1460	ref|NC_002745.2|:c1620664-1618841
17	85	nr1461	ref|NC_002745.2|:1621010-1621261
17	86	nr1462	ref|NC_002745.2|:c1622280-1621306
18	1	nr1463	ref|NC_002745.2|:c1624538-1622337
18	2	nr1464	ref|NC_002745.2|:c1625004-1624543
18	3	nr1465	ref|NC_002745.2|:c1625773-1625096
18	4	nr1466	ref|NC_002745.2|:c1626538-1625822
18	5	nr1467	ref|NC_002745.2|:c1626894-1626541
18	6	nr1468	ref|NC_002745.2|:c1627479-1626895
18	7	nr1469	ref|NC_002745.2|:c1628038-1627469
18	8	nr1470	ref|NC_002745.2|:c1628331-1628041
18	9	nr1471	ref|NC_002745.2|:c1629141-1628335
18	10	nr1472	ref|NC_002745.2|:c1630255-1629155
18	11	nr1473	ref|NC_002745.2|:c1630783-1630256
18	12	nr1474	ref|NC_002745.2|:c1631489-1630803
18	13	nr1475	ref|NC_002745.2|:1631803-1632072
18	14	nr1476	ref|NC_002745.2|:c1632462-1632274
18	15	nr1477	ref|NC_002745.2|:c1633084-1632611
18	16	nr1478	ref|NC_002745.2|:c1635011-1633791
18	17	nr1479	ref|NC_002745.2|:c1636529-1635300
18	18	nr1480	ref|NC_002745.2|:c1637294-1636542
18	19	nr1481	ref|NC_002745.2|:c1638655-1637294
18	20	nr1482	ref|NC_002745.2|:c1639118-1638669
18	21	nr1483	ref|NC_002745.2|:c1640130-1639120
18	22	nr1484	ref|NC_002745.2|:c1640854-1640120
18	23	nr1485	ref|NC_002745.2|:c1641656-1641180
18	24	nr1486	ref|NC_002745.2|:c1642307-1641684
18	25	nr1487	ref|NC_002745.2|:c1643575-1642307
18	26	nr1488	ref|NC_002745.2|:c1644510-1643587
18	27	nr1489	ref|NC_002745.2|:c1645151-1644513
18	28	nr1490	ref|NC_002745.2|:c1645744-1645436
18	29	nr1491	ref|NC_002745.2|:c1646130-1645759
18	30	nr1492	ref|NC_002745.2|:c1646451-1646191
18	31	nr1493	ref|NC_002745.2|:c1649144-1646514
18	32	nr1494	ref|NC_002745.2|:c1651964-1649487
18	33	nr1495	ref|NC_002745.2|:c1652634-1651966
18	34	nr1496	ref|NC_002745.2|:c1654447-1653329

18	35	nr1497	ref|NC_002745.2|:c1655590-1654448
18	36	nr1498	ref|NC_002745.2|:1655902-1656915
18	37	nr1499	ref|NC_002745.2|:c1657298-1657152
18	38	nr1500	ref|NC_002745.2|:c1657520-1657338
18	39	nr1501	ref|NC_002745.2|:c1658042-1657620
18	40	nr1502	ref|NC_002745.2|:1658127-1659401
18	41	nr1503	ref|NC_002745.2|:c1660335-1659562
18	42	nr1504	ref|NC_002745.2|:c1660650-1660549
18	43	nr1505	ref|NC_002745.2|:c1662562-1660796
18	44	nr1506	ref|NC_002745.2|:c1663840-1662578
18	45	nr1507	ref|NC_002745.2|:c1665176-1664301
18	46	nr1508	ref|NC_002745.2|:c1665625-1665173
18	47	nr1509	ref|NC_002745.2|:c1667826-1665637
18	48	nr1510	ref|NC_002745.2|:c1668772-1668254
18	49	nr1511	ref|NC_002745.2|:c1671067-1668794
18	50	nr1512	ref|NC_002745.2|:c1673549-1671270
18	51	nr1513	ref|NC_002745.2|:c1674084-1673824
18	52	nr1514	ref|NC_002745.2|:c1675242-1674103
18	53	nr1515	ref|NC_002745.2|:c1676290-1675265
18	54	nr1516	ref|NC_002745.2|:c1677296-1676292
18	55	nr1517	ref|NC_002745.2|:c1677930-1677328
18	56	nr1518	ref|NC_002745.2|:c1678402-1677944
18	57	nr1519	ref|NC_002745.2|:c1679704-1678412
18	58	nr1520	ref|NC_002745.2|:c1680342-1680058
18	59	nr1521	ref|NC_002745.2|:c1680674-1680354
18	60	nr1522	ref|NC_002745.2|:c1680988-1680680
18	61	nr1523	ref|NC_002745.2|:c1681754-1681224
18	62	nr1524	ref|NC_002745.2|:c1682596-1681754
18	63	nr1525	ref|NC_002745.2|:c1683461-1682988
18	64	nr1526	ref|NC_002745.2|:1683621-1683818
18	65	nr1527	ref|NC_002745.2|:c1684219-1683935
18	66	nr1528	ref|NC_002745.2|:c1685330-1684668
18	67	nr1529	ref|NC_002745.2|:1685864-1686595
18	68	nr1530	ref|NC_002745.2|:c1687503-1686721
18	69	nr1531	ref|NC_002745.2|:c1688031-1687654
18	70	nr1532	ref|NC_002745.2|:c1689930-1688038
18	71	nr1533	ref|NC_002745.2|:c1691012-1689927
18	72	nr1534	ref|NC_002745.2|:c1691685-1691131
18	73	nr1535	ref|NC_002745.2|:c1692389-1691682
18	74	nr1536	ref|NC_002745.2|:c1693930-1692659
18	75	nr1537	ref|NC_002745.2|:c1696573-1693943
18	76	nr1538	ref|NC_002745.2|:1696976-1697536
18	77	nr1539	ref|NC_002745.2|:c1698832-1697759
18	78	nr1540	ref|NC_002745.2|:c1698955-1698866
18	79	nr1541	ref|NC_002745.2|:c1700314-1699028
18	80	nr1542	ref|NC_002745.2|:c1701336-1700362
18	81	nr1543	ref|NC_002745.2|:c1702007-1701339
18	82	nr1544	ref|NC_002745.2|:c1702955-1702029
18	83	nr1545	ref|NC_002745.2|:c1703812-1702997

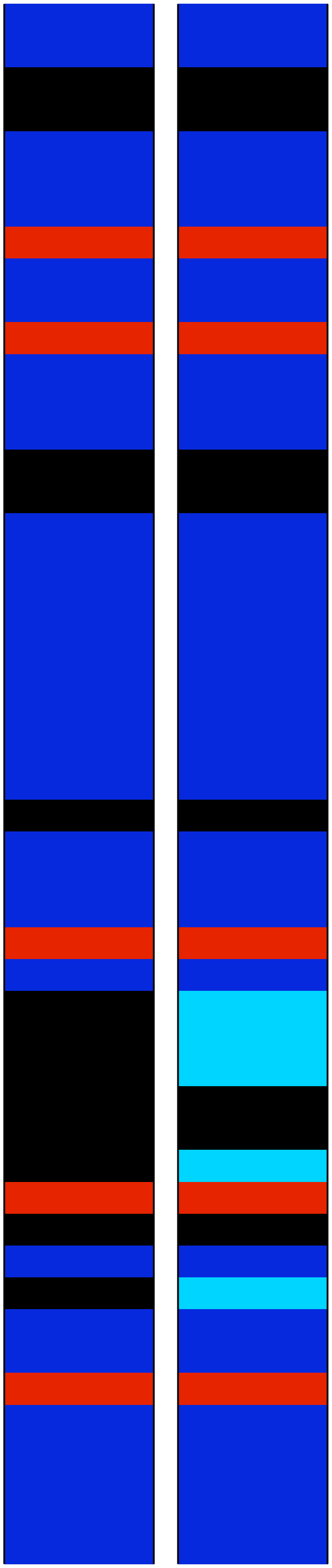

18	84	nr1546	ref|NC_002745.2|:c1705180-1703834
18	85	nr1547	ref|NC_002745.2|:c1705987-1705397
18	86	nr1548	ref|NC_002745.2|:c1707403-1706141
19	1	nr1549	ref|NC_002745.2|:c1708855-1707554
19	2	nr1550	ref|NC_002745.2|:c1709947-1709018
19	3	nr1551	ref|NC_002745.2|:c1710574-1709966
19	4	nr1552	ref|NC_002745.2|:c1711072-1710716
19	5	nr1553	ref|NC_002745.2|:c1711319-1711119
19	6	nr1554	ref|NC_002745.2|:c1711866-1711348
19	7	nr1555	ref|NC_002745.2|:c1713597-1712104
19	8	nr1556	ref|NC_002745.2|:c1715960-1714023
19	9	nr1557	ref|NC_002745.2|:c1717293-1716373
19	10	nr1558	ref|NC_002745.2|:c1718693-1717293
19	11	nr1559	ref|NC_002745.2|:c1719164-1718694
19	12	nr1560	ref|NC_002745.2|:c1720400-1719375
19	13	nr1561	ref|NC_002745.2|:c1721193-1720570
19	14	nr1562	ref|NC_002745.2|:c1722081-1721209
19	15	nr1563	ref|NC_002745.2|:c1724727-1722097
19	16	nr1564	ref|NC_002745.2|:c1726508-1725021
19	17	nr1565	ref|NC_002745.2|:c1728671-1727007
19	18	nr1566	ref|NC_002745.2|:c1729372-1728668
19	19	nr1567	ref|NC_002745.2|:c1731030-1729762
19	20	nr1568	ref|NC_002745.2|:c1732200-1731079
19	21	nr1569	ref|NC_002745.2|:1732548-1733909
19	22	nr1570	ref|NC_002745.2|:c1736014-1734257
19	23	nr1571	ref|NC_002745.2|:c1736959-1736036
19	24	nr1572	ref|NC_002745.2|:c1738217-1737273
19	25	nr1573	ref|NC_002745.2|:c1739074-1738217
19	26	nr1574	ref|NC_002745.2|:c1740498-1739269
19	27	nr1575	ref|NC_002745.2|:c1744145-1740948
19	28	nr1576	ref|NC_002745.2|:c1745107-1744166
19	29	nr1577	ref|NC_002745.2|:c1746717-1745419
19	30	nr1578	ref|NC_002745.2|:1746922-1747335
19	31	nr1579	ref|NC_002745.2|:c1748329-1747640
19	32	nr1580	ref|NC_002745.2|:1748483-1749559
19	33	nr1581	ref|NC_002745.2|:c1750993-1749875
19	34	nr1582	ref|NC_002745.2|:1751134-1751634
19	35	nr1583	ref|NC_002745.2|:c1753082-1751880
19	36	nr1584	ref|NC_002745.2|:c1754117-1753170
19	37	nr1585	ref|NC_002745.2|:c1754734-1754240
19	38	nr1586	ref|NC_002745.2|:c1755602-1754832
19	39	nr1587	ref|NC_002745.2|:c1756869-1755646
19	40	nr1588	ref|NC_002745.2|:c1758008-1756869
19	41	nr1589	ref|NC_002745.2|:c1760075-1758381
19	42	nr1590	ref|NC_002745.2|:1760212-1760676
19	43	nr1591	ref|NC_002745.2|:1760920-1761522
19	44	nr1592	ref|NC_002745.2|:1761714-1763033
19	45	nr1593	ref|NC_002745.2|:c1764001-1763258
19	46	nr1594	ref|NC_002745.2|:1764081-1764527

19	47	nr1595	ref|NC_002745.2|:1764642-1765799
19	48	nr1596	ref|NC_002745.2|:1765786-1767390
19	49	nr1597	ref|NC_002745.2|:c1768670-1767540
19	50	nr1598	ref|NC_002745.2|:c1770243-1768777
19	51	nr1599	ref|NC_002745.2|:c1770988-1770371
19	52	nr1600	ref|NC_002745.2|:1771160-1772434
19	53	nr1601	ref|NC_002745.2|:c1773790-1772528
19	54	nr1602	ref|NC_002745.2|:1774172-1775077
19	55	nr1603	ref|NC_002745.2|:c1777955-1775280
19	56	nr1604	ref|NC_002745.2|:c1779955-1778288
19	57	nr1605	ref|NC_002745.2|:c1782101-1780395
19	58	nr1606	ref|NC_002745.2|:1782270-1782902
19	59	nr1607	ref|NC_002745.2|:1782927-1784096
19	60	nr1608	ref|NC_002745.2|:c1785138-1784194
19	61	nr1609	ref|NC_002745.2|:c1786814-1785723
19	62	nr1610	ref|NC_002745.2|:c1788866-1787508
19	63	nr1611	ref|NC_002745.2|:c1789431-1788940
19	64	nr1612	ref|NC_002745.2|:c1790818-1789505
19	65	nr1613	ref|NC_002745.2|:c1794666-1790842
19	66	nr1614	ref|NC_002745.2|:c1795283-1794687
19	67	nr1615	ref|NC_002745.2|:c1796169-1795312
19	68	nr1616	ref|NC_002745.2|:c1796580-1796269
19	69	nr1617	ref|NC_002745.2|:c1797721-1796645
19	70	nr1618	ref|NC_002745.2|:1797808-1798119
19	71	nr1619	ref|NC_002745.2|:c1799086-1798244
19	72	nr1620	ref|NC_002745.2|:c1800201-1799557
19	73	nr1621	ref|NC_002745.2|:c1801007-1800216
19	74	nr1622	ref|NC_002745.2|:c1802405-1801557
19	75	nr1623	ref|NC_002745.2|:c1803818-1802409
19	76	nr1624	ref|NC_002745.2|:c1804798-1804376
19	77	nr1625	ref|NC_002745.2|:c1805510-1804815
19	78	nr1626	ref|NC_002745.2|:c1807168-1805507
19	79	nr1627	ref|NC_002745.2|:1807575-1808843
19	80	nr1628	ref|NC_002745.2|:c1815520-1808960
19	81	nr1629	ref|NC_002745.2|:c1816157-1815846
19	82	nr1630	ref|NC_002745.2|:c1818593-1816179
19	83	nr1631	ref|NC_002745.2|:c1820065-1818884
19	84	nr1632	ref|NC_002745.2|:1820175-1821128
19	85	nr1633	ref|NC_002745.2|:1821125-1821688
19	86	nr1634	ref|NC_002745.2|:c1822272-1821811
20	1	nr1635	ref|NC_002745.2|:c1823610-1822783
20	2	nr1636	ref|NC_002745.2|:1823844-1824845
20	3	nr1637	ref|NC_002745.2|:c1825431-1824967
20	4	nr1638	ref|NC_002745.2|:c1826625-1825444
20	5	nr1639	ref|NC_002745.2|:c1827268-1826636
20	6	nr1640	ref|NC_002745.2|:c1828318-1827275
20	7	nr1641	ref|NC_002745.2|:c1830301-1828799
20	8	nr1642	ref|NC_002745.2|:1830824-1831138
20	9	nr1643	ref|NC_002745.2|:1831138-1832430

20	10	nr1644	ref|NC_002745.2|:c1833371-1832517
20	11	nr1645	ref|NC_002745.2|:c1833871-1833647
20	12	nr1646	ref|NC_002745.2|:1834070-1834540
20	13	nr1647	ref|NC_002745.2|:1834653-1835096
20	14	nr1648	ref|NC_002745.2|:c1835526-1835083
20	15	nr1649	ref|NC_002745.2|:1835822-1836457
20	16	nr1650	ref|NC_002745.2|:c1837610-1836897
20	17	nr1651	ref|NC_002745.2|:1837870-1838172
20	18	nr1652	ref|NC_002745.2|:1838350-1838793
20	19	nr1653	ref|NC_002745.2|:1838790-1839143
20	20	nr1654	ref|NC_002745.2|:c1841973-1841140
20	21	nr1655	ref|NC_002745.2|:c1843096-1842185
20	22	nr1656	ref|NC_002745.2|:c1844411-1843215
20	23	nr1657	ref|NC_002745.2|:1844783-1846375
20	24	nr1658	ref|NC_002745.2|:1846499-1846675
20	25	nr1659	ref|NC_002745.2|:c1847523-1846753
20	26	nr1660	ref|NC_002745.2|:c1847983-1847504
20	27	nr1661	ref|NC_002745.2|:1848043-1848300
20	28	nr1662	ref|NC_002745.2|:c1849298-1848297
20	29	nr1663	ref|NC_002745.2|:c1850781-1849303
20	30	nr1664	ref|NC_002745.2|:c1851422-1850940
20	31	nr1665	ref|NC_002745.2|:1851734-1852348
20	32	nr1666	ref|NC_002745.2|:1852429-1853424
20	33	nr1667	ref|NC_002745.2|:1853499-1854125
20	34	nr1668	ref|NC_002745.2|:1854166-1854507
20	35	nr1669	ref|NC_002745.2|:1854608-1855180
20	36	nr1670	ref|NC_002745.2|:c1857478-1856978
20	37	nr1671	ref|NC_002745.2|:c1859159-1857930
20	38	nr1672	ref|NC_002745.2|:c1860708-1859152
20	39	nr1673	ref|NC_002745.2|:c1861788-1861069
20	40	nr1674	ref|NC_002745.2|:c1862665-1861946
20	41	nr1675	ref|NC_002745.2|:c1863505-1862786
20	42	nr1676	ref|NC_002745.2|:c1864285-1863563
20	43	nr1677	ref|NC_002745.2|:c1865117-1864410
20	44	nr1678	ref|NC_002745.2|:c1865771-1865553
20	45	nr1679	ref|NC_002745.2|:1866080-1866661
20	46	nr1680	ref|NC_002745.2|:c1867412-1867134
20	47	nr1681	ref|NC_002745.2|:1867608-1868081
20	48	nr1682	ref|NC_002745.2|:1868086-1868877
20	49	nr1683	ref|NC_002745.2|:c1870230-1869247
20	50	nr1684	ref|NC_002745.2|:c1871167-1870232
20	51	nr1685	ref|NC_002745.2|:1872531-1873319
20	52	nr1686	ref|NC_002745.2|:1874153-1874647
20	53	nr1687	ref|NC_002745.2|:1874982-1875758
20	54	nr1688	ref|NC_002745.2|:c1877086-1876310
20	55	nr1689	ref|NC_002745.2|:c1878145-1877369
20	56	nr1690	ref|NC_002745.2|:c1878573-1878163
20	57	nr1691	ref|NC_002745.2|:c1878934-1878533
20	58	nr1692	ref|NC_002745.2|:c1879816-1879088

20	59	nr1693	ref|NC_002745.2|:c1880570-1879851
20	60	nr1694	ref|NC_002745.2|:c1881633-1880851
20	61	nr1695	ref|NC_002745.2|:c1883547-1882993
20	62	nr1696	ref|NC_002745.2|:c1885270-1883870
20	63	nr1697	ref|NC_002745.2|:c1886217-1885294
20	64	nr1698	ref|NC_002745.2|:c1887312-1886275
20	65	nr1699	ref|NC_002745.2|:1887575-1888078
20	66	nr1700	ref|NC_002745.2|:c1889425-1888202
20	67	nr1701	ref|NC_002745.2|:c1890158-1889418
20	68	nr1702	ref|NC_002745.2|:1890292-1890714
20	69	nr1703	ref|NC_002745.2|:1890856-1891221
20	70	nr1704	ref|NC_002745.2|:1891956-1892513
20	71	nr1705	ref|NC_002745.2|:1892718-1893680
20	72	nr1706	ref|NC_002745.2|:c1894742-1893801
20	73	nr1707	ref|NC_002745.2|:c1897675-1894739
20	74	nr1708	ref|NC_002745.2|:c1898861-1897665
20	75	nr1709	ref|NC_002745.2|:c1900133-1899789
20	76	nr1710	ref|NC_002745.2|:c1901326-1900202
20	77	nr1711	ref|NC_002745.2|:c1901969-1901505
20	78	nr1712	ref|NC_002745.2|:c1902948-1902325
20	79	nr1713	ref|NC_002745.2|:c1904082-1902970
20	80	nr1714	ref|NC_002745.2|:1904245-1905066
20	81	nr1715	ref|NC_002745.2|:c1906904-1905519
20	82	nr1716	ref|NC_002745.2|:c1907495-1907100
20	83	nr1717	ref|NC_002745.2|:c1908195-1908043
20	84	nr1718	ref|NC_002745.2|:c1908819-1908220
20	85	nr1719	ref|NC_002745.2|:c1909448-1908978
20	86	nr1720	ref|NC_002745.2|:c1910580-1909453
21	1	nr1721	ref|NC_002745.2|:c1911459-1910731
21	2	nr1722	ref|NC_002745.2|:c1912771-1911446
21	3	nr1723	ref|NC_002745.2|:c1914222-1913161
21	4	nr1724	ref|NC_002745.2|:1914806-1916125
21	5	nr1725	ref|NC_002745.2|:c1925070-1924624
21	6	nr1726	ref|NC_002745.2|:c1926117-1925167
21	7	nr1727	ref|NC_002745.2|:c1926578-1926123
21	8	nr1728	ref|NC_002745.2|:1926659-1927948
21	9	nr1729	ref|NC_002745.2|:1928241-1929335
21	10	nr1730	ref|NC_002745.2|:c1931261-1929525
21	11	nr1731	ref|NC_002745.2|:c1932094-1931552
21	12	nr1732	ref|NC_002745.2|:c1933434-1932397
21	13	nr1733	ref|NC_002745.2|:1933586-1934563
21	14	nr1734	ref|NC_002745.2|:c1935660-1934824
21	15	nr1735	ref|NC_002745.2|:c1937186-1935669
21	16	nr1736	ref|NC_002745.2|:c1937515-1937201
21	17	nr1737	ref|NC_002745.2|:c1938311-1937493
21	18	nr1738	ref|NC_002745.2|:c1939380-1938571
21	19	nr1739	ref|NC_002745.2|:c1940235-1939720
21	20	nr1740	ref|NC_002745.2|:c1940527-1940366
21	21	nr1741	ref|NC_002745.2|:1940751-1941902

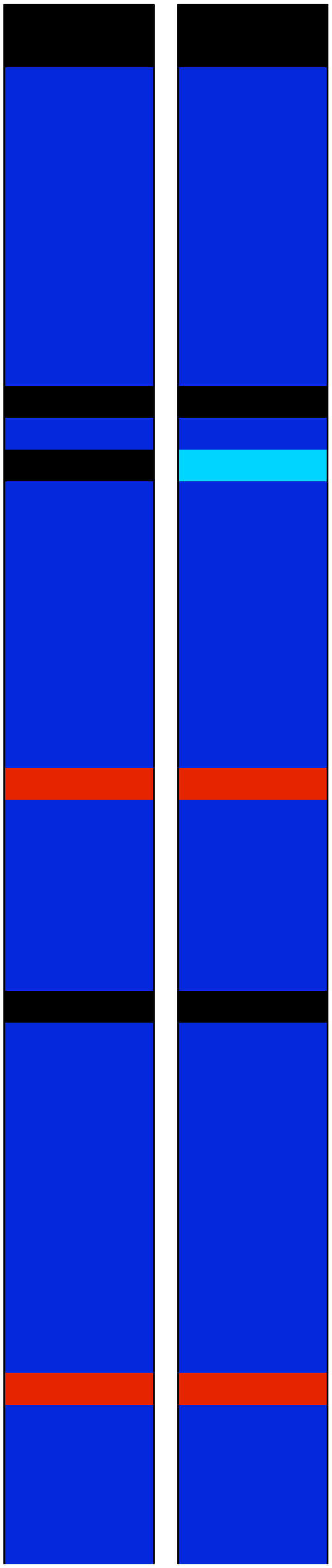

21	22	nr1742	ref|NC_002745.2|:c1942692-1942162
21	23	nr1743	ref|NC_002745.2|:c1944029-1942782
21	24	nr1744	ref|NC_002745.2|:c1944247-1944041
21	25	nr1745	ref|NC_002745.2|:1944388-1944852
21	26	nr1746	ref|NC_002745.2|:1944859-1945134
21	27	nr1747	ref|NC_002745.2|:1945412-1946629
21	28	nr1748	ref|NC_002745.2|:c1947371-1946742
21	29	nr1749	ref|NC_002745.2|:c1948404-1947361
21	30	nr1750	ref|NC_002745.2|:c1949102-1948401
21	31	nr1751	ref|NC_002745.2|:c1949503-1949117
21	32	nr1752	ref|NC_002745.2|:c1950478-1949720
21	33	nr1753	ref|NC_002745.2|:1951009-1951995
21	34	nr1754	ref|NC_002745.2|:c1952878-1952426
21	35	nr1755	ref|NC_002745.2|:c1953795-1953064
21	36	nr1756	ref|NC_002745.2|:c1955110-1953797
21	37	nr1757	ref|NC_002745.2|:1955402-1955902
21	38	nr1758	ref|NC_002745.2|:1956292-1956846
21	39	nr1759	ref|NC_002745.2|:c1957984-1956914
21	40	nr1760	ref|NC_002745.2|:c1958761-1958231
21	41	nr1761	ref|NC_002745.2|:c1960292-1958931
21	42	nr1762	ref|NC_002745.2|:c1961320-1960373
21	43	nr1763	ref|NC_002745.2|:c1963515-1962088
21	44	nr1764	ref|NC_002745.2|:c1964985-1963528
21	45	nr1765	ref|NC_002745.2|:c1965289-1964987
21	46	nr1766	ref|NC_002745.2|:1965656-1967194
21	47	nr1767	ref|NC_002745.2|:c1968482-1967283
21	48	nr1768	ref|NC_002745.2|:c1970498-1968495
21	49	nr1769	ref|NC_002745.2|:c1972694-1970502
21	50	nr1770	ref|NC_002745.2|:c1973383-1972691
21	51	nr1771	ref|NC_002745.2|:c1973857-1973555
21	52	nr1772	ref|NC_002745.2|:c1975261-1973966
21	53	nr1773	ref|NC_002745.2|:1976121-1977287
21	54	nr1774	ref|NC_002745.2|:1977318-1977644
21	55	nr1775	ref|NC_002745.2|:c1978692-1978090
21	56	nr1776	ref|NC_002745.2|:c1979784-1978963
21	57	nr1777	ref|NC_002745.2|:c1981246-1979777
21	58	nr1778	ref|NC_002745.2|:1981430-1982506
21	59	nr1779	ref|NC_002745.2|:1982526-1983320
21	60	nr1780	ref|NC_002745.2|:c1985072-1983510
21	61	nr1781	ref|NC_002745.2|:c1986374-1985277
21	62	nr1782	ref|NC_002745.2|:1986743-1987303
21	63	nr1783	ref|NC_002745.2|:1987356-1988285
21	64	nr1784	ref|NC_002745.2|:1988703-1990082
21	65	nr1785	ref|NC_002745.2|:c1991230-1990202
21	66	nr1786	ref|NC_002745.2|:1991500-1991901
21	67	nr1787	ref|NC_002745.2|:c1992089-1991916
21	68	nr1788	ref|NC_002745.2|:c1992647-1992474
21	69	nr1789	ref|NC_002745.2|:1993098-1994138
21	70	nr1790	ref|NC_002745.2|:c1995037-1994195

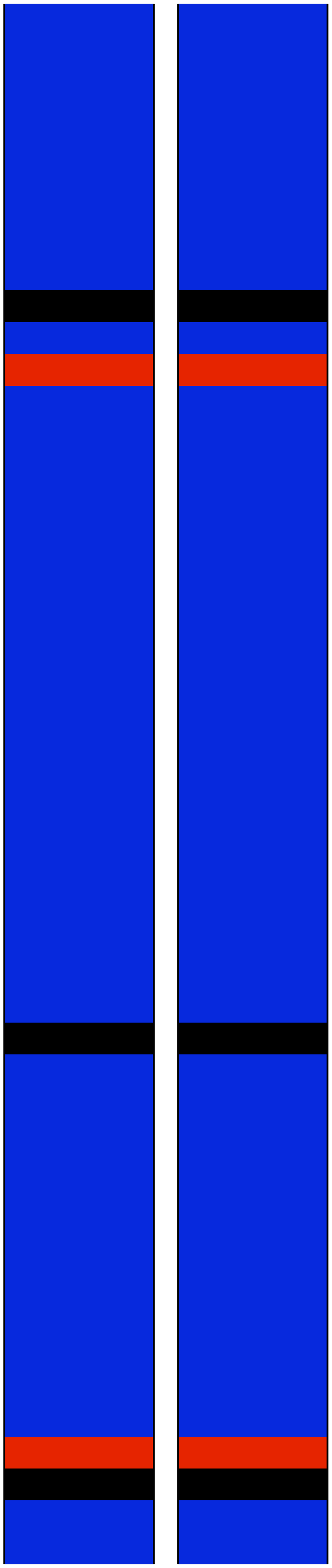

21	71	nr1791	ref|NC_002745.2|:c1995591-1995034
21	72	nr1792	ref|NC_002745.2|:c1996175-1995651
21	73	nr1793	ref|NC_002745.2|:1996296-1996859
21	74	nr1794	ref|NC_002745.2|:c1997665-1996925
21	75	nr1795	ref|NC_002745.2|:c1998537-1997665
21	76	nr1796	ref|NC_002745.2|:c1999214-1998534
21	77	nr1797	ref|NC_002745.2|:c2000111-1999215
21	78	nr1798	ref|NC_002745.2|:c2000488-2000108
21	79	nr1799	ref|NC_002745.2|:c2000922-2000746
21	80	nr1800	ref|NC_002745.2|:2001463-2002749
21	81	nr1801	ref|NC_002745.2|:c2003348-2003028
21	82	nr1802	ref|NC_002745.2|:c2004763-2003333
21	83	nr1803	ref|NC_002745.2|:2005149-2005349
21	84	nr1804	ref|NC_002745.2|:2005721-2005900
21	85	nr1805	ref|NC_002745.2|:2005924-2006211
21	86	nr1806	ref|NC_002745.2|:c2006878-2006528
22	1	nr1807	ref|NC_002745.2|:2007561-2008010
22	2	nr1808	ref|NC_002745.2|:c2008311-2008105
22	3	nr1809	ref|NC_002745.2|:c2008571-2008308
22	4	nr1810	ref|NC_002745.2|:c2009577-2009086
22	5	nr1811	ref|NC_002745.2|:c2010523-2009768
22	6	nr1812	ref|NC_002745.2|:c2010789-2010535
22	7	nr1813	ref|NC_002745.2|:c2011135-2011001
22	8	nr1814	ref|NC_002745.2|:c2012162-2011380
22	9	nr1815	ref|NC_002745.2|:c2012447-2012271
22	10	nr1816	ref|NC_002745.2|:c2012948-2012574
22	11	nr1817	ref|NC_002745.2|:c2013333-2013004
22	12	nr1818	ref|NC_002745.2|:c2013489-2013337
22	13	nr1819	ref|NC_002745.2|:c2017264-2013482
22	14	nr1820	ref|NC_002745.2|:c2018764-2017280
22	15	nr1821	ref|NC_002745.2|:c2023290-2018761
22	16	nr1822	ref|NC_002745.2|:c2023885-2023535
22	17	nr1823	ref|NC_002745.2|:c2024845-2024201
22	18	nr1824	ref|NC_002745.2|:c2025253-2024846
22	19	nr1825	ref|NC_002745.2|:c2025654-2025250
22	20	nr1826	ref|NC_002745.2|:c2026013-2025651
22	21	nr1827	ref|NC_002745.2|:c2026281-2025997
22	22	nr1828	ref|NC_002745.2|:c2026555-2026271
22	23	nr1829	ref|NC_002745.2|:c2027720-2026575
22	24	nr1830	ref|NC_002745.2|:c2028481-2027744
22	25	nr1831	ref|NC_002745.2|:c2029652-2028465
22	26	nr1832	ref|NC_002745.2|:c2031329-2029668
22	27	nr1833	ref|NC_002745.2|:c2031670-2031326
22	28	nr1834	ref|NC_002745.2|:c2032101-2031802
22	29	nr1835	ref|NC_002745.2|:c2032749-2032333
22	30	nr1836	ref|NC_002745.2|:c2032980-2032777
22	31	nr1837	ref|NC_002745.2|:c2033126-2032977
22	32	nr1838	ref|NC_002745.2|:c2033329-2033123
22	33	nr1839	ref|NC_002745.2|:c2033562-2033326

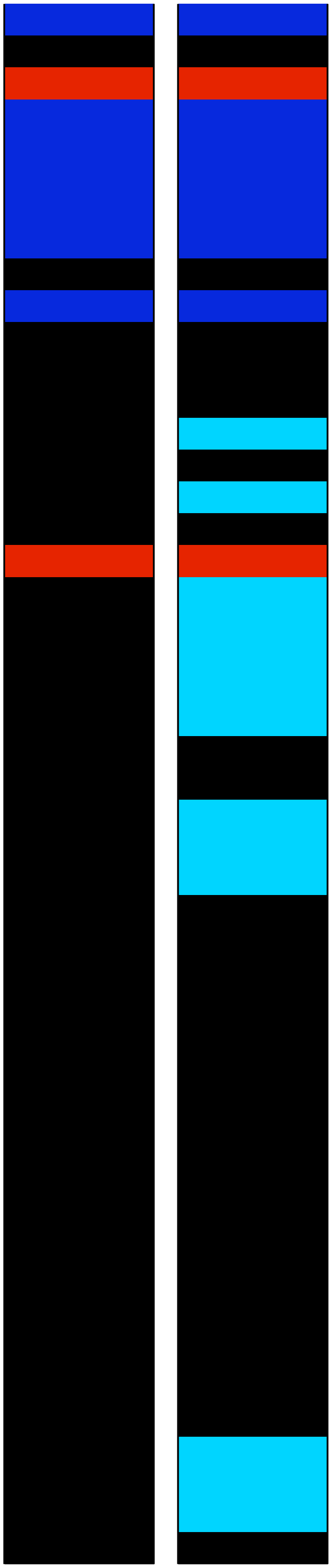

22	34	nr1840	ref|NC_002745.2|:c2034144-2033608
22	35	nr1841	ref|NC_002745.2|:c2034389-2034141
22	36	nr1842	ref|NC_002745.2|:c2034649-2034401
22	37	nr1843	ref|NC_002745.2|:c2034903-2034646
22	38	nr1844	ref|NC_002745.2|:c2035274-2034903
22	39	nr1845	ref|NC_002745.2|:c2035691-2035287
22	40	nr1846	ref|NC_002745.2|:c2035918-2035700
22	41	nr1847	ref|NC_002745.2|:c2036818-2035925
22	42	nr1848	ref|NC_002745.2|:c2037318-2036848
22	43	nr1849	ref|NC_002745.2|:c2037804-2037319
22	44	nr1850	ref|NC_002745.2|:c2038937-2038017
22	45	nr1851	ref|NC_002745.2|:c2040882-2038939
22	46	nr1852	ref|NC_002745.2|:c2041154-2040891
22	47	nr1853	ref|NC_002745.2|:c2041423-2041163
22	48	nr1854	ref|NC_002745.2|:c2041730-2041428
22	49	nr1855	ref|NC_002745.2|:c2041986-2041825
22	50	nr1856	ref|NC_002745.2|:c2042306-2041983
22	51	nr1857	ref|NC_002745.2|:2042361-2042741
22	52	nr1858	ref|NC_002745.2|:c2042925-2042728
22	53	nr1859	ref|NC_002745.2|:c2043693-2042941
22	54	nr1860	ref|NC_002745.2|:2043744-2044073
22	55	nr1861	ref|NC_002745.2|:c2044277-2044062
22	56	nr1862	ref|NC_002745.2|:c2044556-2044293
22	57	nr1863	ref|NC_002745.2|:2044690-2045403
22	58	nr1864	ref|NC_002745.2|:2045419-2046351
22	59	nr1865	ref|NC_002745.2|:2046902-2047084
22	60	nr1866	ref|NC_002745.2|:2047184-2048167
22	61	nr1867	ref|NC_002745.2|:2048178-2048420
22	62	nr1868	ref|NC_002745.2|:2048483-2049520
22	63	nr1869	ref|NC_002745.2|:2049532-2050401
22	64	nr1870	ref|NC_002745.2|:c2051655-2050639
22	65	nr1871	ref|NC_002745.2|:c2052732-2051677
22	66	nr1872	ref|NC_002745.2|:2053168-2054391
22	67	nr1873	ref|NC_002745.2|:2054835-2056142
22	68	nr1874	ref|NC_002745.2|:2057159-2057881
22	69	nr1875	ref|NC_002745.2|:c2058850-2058050
22	70	nr1876	ref|NC_002745.2|:2059637-2060197
22	71	nr1877	ref|NC_002745.2|:2061076-2061780
22	72	nr1878	ref|NC_002745.2|:c2062504-2061935
22	73	nr1879	ref|NC_002745.2|:c2062842-2062501
22	74	nr1880	ref|NC_002745.2|:c2063372-2062845
22	75	nr1881	ref|NC_002745.2|:c2063641-2063423
22	76	nr1882	ref|NC_002745.2|:c2064237-2063659
22	77	nr1883	ref|NC_002745.2|:c2064590-2064249
22	78	nr1884	ref|NC_002745.2|:c2065681-2065040
22	79	nr1885	ref|NC_002745.2|:c2066058-2065678
22	80	nr1886	ref|NC_002745.2|:c2068077-2066368
22	81	nr1887	ref|NC_002745.2|:c2068960-2068091
22	82	nr1888	ref|NC_002745.2|:c2069351-2069025

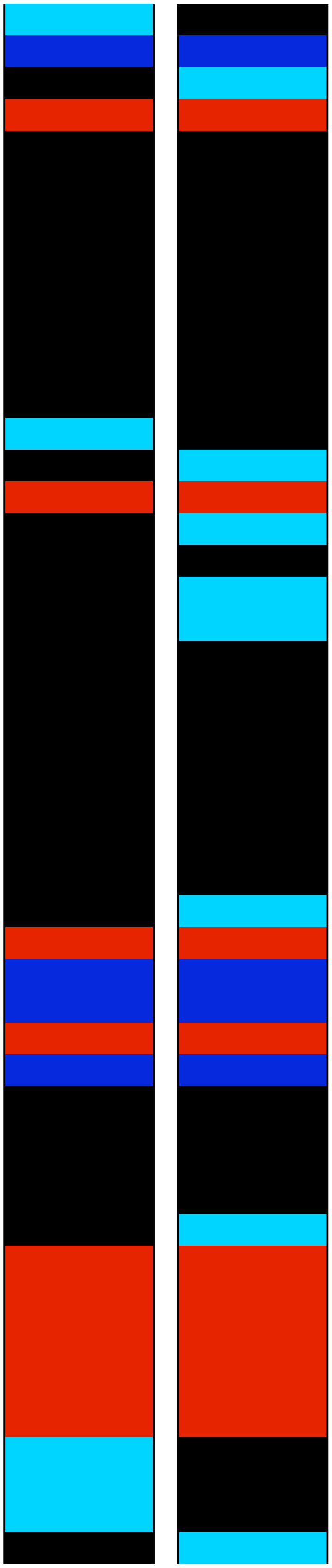

22	83	nr1889	ref|NC_002745.2|:c2069563-2069354		
22	84	nr1890	ref|NC_002745.2|:c2070016-2069699		
22	85	nr1891	ref|NC_002745.2|:c2070239-2070021		
22	86	nr1892	ref|NC_002745.2|:2070412-2071086		
23	1	nr1893	ref|NC_002745.2|:2071100-2072272		
23	2	nr1894	ref|NC_002745.2|:c2073957-2072341		
23	3	nr1895	ref|NC_002745.2|:c2074317-2074033		
23	4	nr1896	ref|NC_002745.2|:2074492-2075235		
23	5	nr1897	ref|NC_002745.2|:c2076507-2075260		
23	6	nr1898	ref|NC_002745.2|:2076704-2077330		
23	7	nr1899	ref|NC_002745.2|:2077691-2078476		
23	8	nr1900	ref|NC_002745.2|:c2079210-2079076		
23	9	nr1901	ref|NC_002745.2|:2079446-2080009		
23	10	nr1902	ref|NC_002745.2|:2080012-2080155		
23	11	nr1903	ref|NC_002745.2|:2080353-2081468		
23	12	nr1904	ref|NC_002745.2|:2081487-2082203		
23	13	nr1905	ref|NC_002745.2|:c2083531-2082572		
23	14	nr1906	ref|NC_002745.2|:c2085012-2083528		
23	15	nr1907	ref|NC_002745.2|:c2086111-2085161		
23	16	nr1908	ref|NC_002745.2|:c2087544-2086294		
23	17	nr1909	ref|NC_002745.2|:c2087976-2087752		
23	18	nr1910	ref|NC_002745.2|:c2089115-2088036		
23	19	nr1911	ref|NC_002745.2|:c2090054-2089419		
23	20	nr1912	ref|NC_002745.2|:2090307-2092235		
23	21	nr1913	ref|NC_002745.2|:2092597-2094207		
23	22	nr1914	ref|NC_002745.2|:c2095547-2094522		
23	23	nr1915	ref|NC_002745.2|:c2096004-2095540		
23	24	nr1916	ref|NC_002745.2|:c2096666-2095977		
23	25	nr1917	ref|NC_002745.2|:c2097081-2096620		
23	26	nr1918	ref|NC_002745.2|:2097592-2099280		
23	27	nr1919	ref|NC_002745.2|:2099308-2101077		
23	28	nr1920	ref|NC_002745.2|:2101077-2101331		
23	29	nr1921	ref|NC_002745.2|:2101468-2102472		
23	30	nr1922	ref|NC_002745.2|:2102502-2104031		
23	31	nr1923	ref|NC_002745.2|:2104034-2105080		
23	32	nr1924	ref|NC_002745.2|:2105094-2106464		
23	33	nr1925	ref|NC_002745.2|:2106465-2107037		
23	34	nr1926	ref|NC_002745.2|:2107052-2108320		
23	35	nr1927	ref|NC_002745.2|:c2115573-2115118		
23	36	nr1928	ref|NC_002745.2|:c2117716-2115566		
23	37	nr1929	ref|NC_002745.2|:c2118920-2118150		
23	38	nr1930	ref|NC_002745.2|:c2119374-2118895		
23	39	nr1931	ref|NC_002745.2|:c2119702-2119376		
23	40	nr1932	ref|NC_002745.2|:c2120822-2119821		
23	41	nr1933	ref|NC_002745.2|:c2121534-2121172		
23	42	nr1934	ref|NC_002745.2|:c2121701-2121531		
23	43	nr1935	ref|NC_002745.2|:c2122934-2121786		
23	44	nr1936	ref|NC_002745.2|:c2123359-2123000		
23	45	nr1937	ref|NC_002745.2|:c2123854-2123363		

23	46	nr1938	ref|NC_002745.2|:c2125424-2123841
23	47	nr1939	ref|NC_002745.2|:c2125896-2125417
23	48	nr1940	ref|NC_002745.2|:c2126664-2126104
23	49	nr1941	ref|NC_002745.2|:c2128711-2126684
23	50	nr1942	ref|NC_002745.2|:c2130406-2128730
23	51	nr1943	ref|NC_002745.2|:2130678-2133335
23	52	nr1944	ref|NC_002745.2|:2133335-2134030
23	53	nr1945	ref|NC_002745.2|:c2135733-2134414
23	54	nr1946	ref|NC_002745.2|:c2137422-2135902
23	55	nr1947	ref|NC_002745.2|:c2139297-2137939
23	56	nr1948	ref|NC_002745.2|:c2140382-2139312
23	57	nr1949	ref|NC_002745.2|:2140700-2141902
23	58	nr1950	ref|NC_002745.2|:c2142303-2142166
23	59	nr1951	ref|NC_002745.2|:c2142640-2142431
23	60	nr1952	ref|NC_002745.2|:c2142945-2142652
23	61	nr1953	ref|NC_002745.2|:2143111-2144595
23	62	nr1954	ref|NC_002745.2|:2144623-2145270
23	63	nr1955	ref|NC_002745.2|:c2146955-2146083
23	64	nr1956	ref|NC_002745.2|:c2147682-2147041
23	65	nr1957	ref|NC_002745.2|:c2148475-2147684
23	66	nr1958	ref|NC_002745.2|:c2149289-2148459
23	67	nr1959	ref|NC_002745.2|:c2149971-2149282
23	68	nr1960	ref|NC_002745.2|:c2151053-2150358
23	69	nr1961	ref|NC_002745.2|:c2151837-2151442
23	70	nr1962	ref|NC_002745.2|:2152031-2152471
23	71	nr1963	ref|NC_002745.2|:c2152970-2152530
23	72	nr1964	ref|NC_002745.2|:c2154269-2153004
23	73	nr1965	ref|NC_002745.2|:c2154613-2154380
23	74	nr1966	ref|NC_002745.2|:c2155579-2155175
23	75	nr1967	ref|NC_002745.2|:c2157011-2155599
23	76	nr1968	ref|NC_002745.2|:c2157899-2157033
23	77	nr1969	ref|NC_002745.2|:c2159438-2157930
23	78	nr1970	ref|NC_002745.2|:c2159999-2159460
23	79	nr1971	ref|NC_002745.2|:c2160520-2159999
23	80	nr1972	ref|NC_002745.2|:c2160930-2160718
23	81	nr1973	ref|NC_002745.2|:c2161701-2160973
23	82	nr1974	ref|NC_002745.2|:c2162075-2161722
23	83	nr1975	ref|NC_002745.2|:c2163372-2162242
23	84	nr1976	ref|NC_002745.2|:c2164022-2163393
23	85	nr1977	ref|NC_002745.2|:c2165288-2164050
23	86	nr1978	ref|NC_002745.2|:c2165839-2165315
24	1	nr1979	ref|NC_002745.2|:c2166365-2165946
24	2	nr1980	ref|NC_002745.2|:c2167408-2166362
24	3	nr1981	ref|NC_002745.2|:c2168328-2167492
24	4	nr1982	ref|NC_002745.2|:c2169391-2168315
24	5	nr1983	ref|NC_002745.2|:c2169991-2169392
24	6	nr1984	ref|NC_002745.2|:c2170592-2170338
24	7	nr1985	ref|NC_002745.2|:c2172026-2170710
24	8	nr1986	ref|NC_002745.2|:c2173700-2172273

24	9	nr1987	ref|NC_002745.2|:c2174276-2173938
24	10	nr1988	ref|NC_002745.2|:c2175621-2174362
24	11	nr1989	ref|NC_002745.2|:c2176947-2176087
24	12	nr1990	ref|NC_002745.2|:2177165-2177686
24	13	nr1991	ref|NC_002745.2|:c2179405-2177795
24	14	nr1992	ref|NC_002745.2|:c2180271-2179741
24	15	nr1993	ref|NC_002745.2|:c2181243-2180383
24	16	nr1994	ref|NC_002745.2|:2181668-2182471
24	17	nr1995	ref|NC_002745.2|:c2183273-2182602
24	18	nr1996	ref|NC_002745.2|:c2184865-2183672
24	19	nr1997	ref|NC_002745.2|:c2186049-2184865
24	20	nr1998	ref|NC_002745.2|:2186360-2186830
24	21	nr1999	ref|NC_002745.2|:c2187332-2186982
24	22	nr2000	ref|NC_002745.2|:c2188686-2187346
24	23	nr2001	ref|NC_002745.2|:c2189588-2188926
24	24	nr2002	ref|NC_002745.2|:2189902-2190612
24	25	nr2003	ref|NC_002745.2|:c2191176-2190733
24	26	nr2004	ref|NC_002745.2|:c2191789-2191376
24	27	nr2005	ref|NC_002745.2|:c2193619-2192246
24	28	nr2006	ref|NC_002745.2|:c2194521-2194291
24	29	nr2007	ref|NC_002745.2|:c2195490-2194552
24	30	nr2008	ref|NC_002745.2|:c2196591-2195926
24	31	nr2009	ref|NC_002745.2|:2196743-2197063
24	32	nr2010	ref|NC_002745.2|:2197065-2198042
24	33	nr2011	ref|NC_002745.2|:2198308-2198781
24	34	nr2012	ref|NC_002745.2|:c2199659-2198997
24	35	nr2013	ref|NC_002745.2|:2200193-2200924
24	36	nr2014	ref|NC_002745.2|:c2201832-2201050
24	37	nr2015	ref|NC_002745.2|:c2202360-2201983
24	38	nr2016	ref|NC_002745.2|:c2204259-2202367
24	39	nr2017	ref|NC_002745.2|:c2205341-2204256
24	40	nr2018	ref|NC_002745.2|:2205452-2206111
24	41	nr2019	ref|NC_002745.2|:2206973-2207164
24	42	nr2020	ref|NC_002745.2|:c2207263-2207081
24	43	nr2021	ref|NC_002745.2|:2207165-2207335
24	44	nr2022	ref|NC_002745.2|:2207322-2207486
24	45	nr2023	ref|NC_002745.2|:c2209001-2208144
24	46	nr2024	ref|NC_002745.2|:c2209851-2209069
24	47	nr2025	ref|NC_002745.2|:c2211888-2210083
24	48	nr2026	ref|NC_002745.2|:2212356-2212511
24	49	nr2027	ref|NC_002745.2|:2212569-2214107
24	50	nr2028	ref|NC_002745.2|:2214142-2216274
24	51	nr2029	ref|NC_002745.2|:2216286-2216720
24	52	nr2030	ref|NC_002745.2|:2216720-2217826
24	53	nr2031	ref|NC_002745.2|:c2225590-2218145
24	54	nr2032	ref|NC_002745.2|:c2227223-2225868
24	55	nr2033	ref|NC_002745.2|:c2228182-2227250
24	56	nr2034	ref|NC_002745.2|:c2228993-2228184
24	57	nr2035	ref|NC_002745.2|:c2230090-2229182

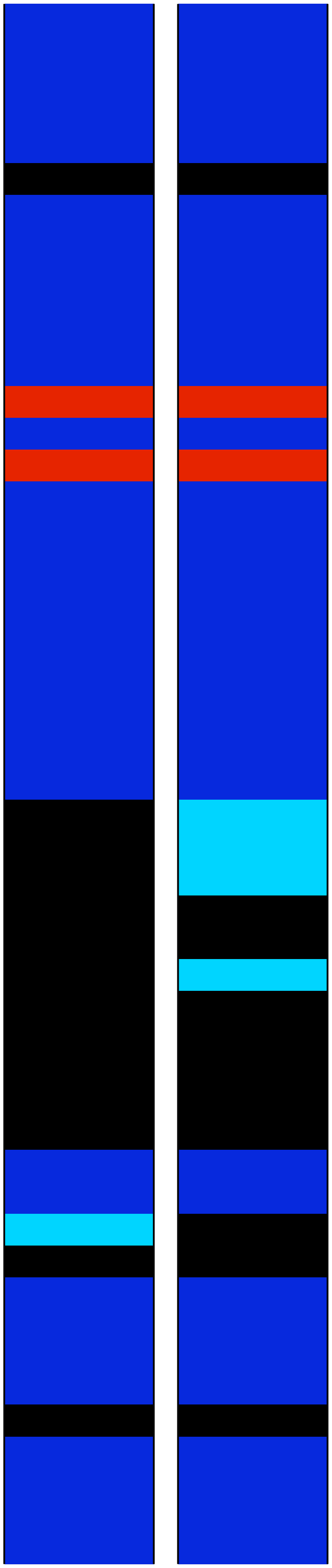

24	58	nr2036	ref|NC_002745.2|:c2237395-2236331
24	59	nr2037	ref|NC_002745.2|:c2238985-2237543
24	60	nr2038	ref|NC_002745.2|:c2239781-2239308
24	61	nr2039	ref|NC_002745.2|:c2241223-2239880
24	62	nr2040	ref|NC_002745.2|:c2242086-2241400
24	63	nr2041	ref|NC_002745.2|:c2243289-2242102
24	64	nr2042	ref|NC_002745.2|:c2243848-2243333
24	65	nr2043	ref|NC_002745.2|:2244025-2244285
24	66	nr2044	ref|NC_002745.2|:c2245724-2244354
24	67	nr2045	ref|NC_002745.2|:c2246928-2245960
24	68	nr2046	ref|NC_002745.2|:c2247956-2246925
24	69	nr2047	ref|NC_002745.2|:c2248951-2247968
24	70	nr2048	ref|NC_002745.2|:c2250413-2249343
24	71	nr2049	ref|NC_002745.2|:c2252177-2250420
24	72	nr2050	ref|NC_002745.2|:c2253357-2252164
24	73	nr2051	ref|NC_002745.2|:2253459-2255435
24	74	nr2052	ref|NC_002745.2|:c2256105-2255596
24	75	nr2053	ref|NC_002745.2|:c2256407-2256168
24	76	nr2054	ref|NC_002745.2|:c2256956-2256420
24	77	nr2055	ref|NC_002745.2|:c2258693-2257131
24	78	nr2056	ref|NC_002745.2|:c2260008-2259001
24	79	nr2057	ref|NC_002745.2|:c2261244-2260243
24	80	nr2058	ref|NC_002745.2|:c2262391-2261522
24	81	nr2059	ref|NC_002745.2|:c2264069-2262657
24	82	nr2060	ref|NC_002745.2|:c2265799-2264087
24	83	nr2061	ref|NC_002745.2|:c2266116-2265805
24	84	nr2062	ref|NC_002745.2|:c2267118-2266138
24	85	nr2063	ref|NC_002745.2|:c2268054-2267122
24	86	nr2064	ref|NC_002745.2|:c2268582-2268067
25	1	nr2065	ref|NC_002745.2|:c2269026-2268598
25	2	nr2066	ref|NC_002745.2|:c2270148-2269393
25	3	nr2067	ref|NC_002745.2|:2270399-2271130
25	4	nr2068	ref|NC_002745.2|:2271179-2271328
25	5	nr2069	ref|NC_002745.2|:c2271618-2271415
25	6	nr2070	ref|NC_002745.2|:c2272699-2271851
25	7	nr2071	ref|NC_002745.2|:c2273185-2272769
25	8	nr2072	ref|NC_002745.2|:2273593-2276022
25	9	nr2073	ref|NC_002745.2|:c2276936-2276082
25	10	nr2074	ref|NC_002745.2|:c2277962-2277147
25	11	nr2075	ref|NC_002745.2|:2278257-2278682
25	12	nr2076	ref|NC_002745.2|:c2279770-2279066
25	13	nr2077	ref|NC_002745.2|:c2281471-2279807
25	14	nr2078	ref|NC_002745.2|:2281823-2281993
25	15	nr2079	ref|NC_002745.2|:c2283737-2282721
25	16	nr2080	ref|NC_002745.2|:c2284647-2283982
25	17	nr2081	ref|NC_002745.2|:c2285258-2284611
25	18	nr2082	ref|NC_002745.2|:c2286832-2286509
25	19	nr2083	ref|NC_002745.2|:c2287368-2287213
25	20	nr2084	ref|NC_002745.2|:c2287510-2287346

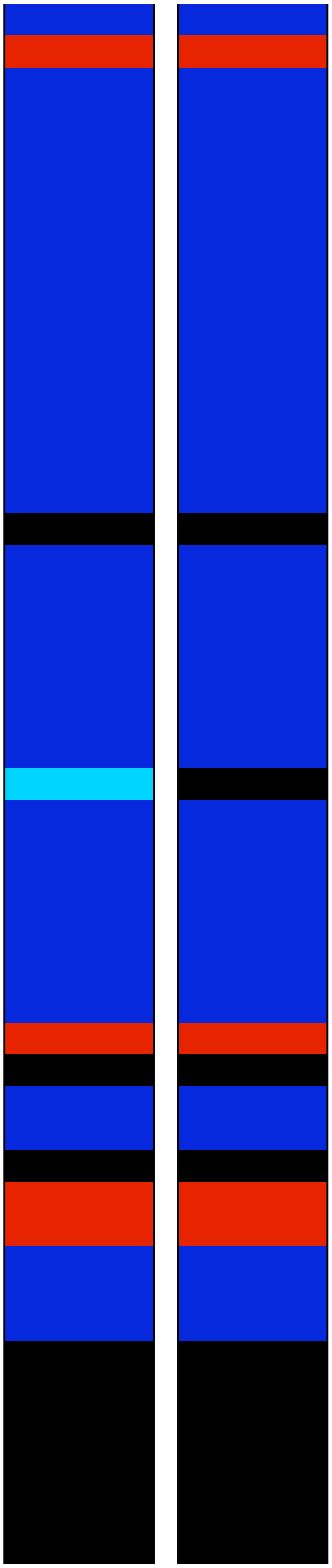

25	21	nr2085	ref|NC_002745.2|:c2287859-2287533
25	22	nr2086	ref|NC_002745.2|:2288704-2289651
25	23	nr2087	ref|NC_002745.2|:c2290221-2289829
25	24	nr2088	ref|NC_002745.2|:c2290678-2290241
25	25	nr2089	ref|NC_002745.2|:c2291721-2290918
25	26	nr2090	ref|NC_002745.2|:c2292532-2291726
25	27	nr2091	ref|NC_002745.2|:c2293382-2292522
25	28	nr2092	ref|NC_002745.2|:c2294188-2293379
25	29	nr2093	ref|NC_002745.2|:c2295094-2294726
25	30	nr2094	ref|NC_002745.2|:c2296055-2295111
25	31	nr2095	ref|NC_002745.2|:c2296519-2296130
25	32	nr2096	ref|NC_002745.2|:c2296908-2296543
25	33	nr2097	ref|NC_002745.2|:c2297044-2296931
25	34	nr2098	ref|NC_002745.2|:c2297294-2297076
25	35	nr2099	ref|NC_002745.2|:c2298134-2297487
25	36	nr2100	ref|NC_002745.2|:c2299443-2298151
25	37	nr2101	ref|NC_002745.2|:c2299883-2299443
25	38	nr2102	ref|NC_002745.2|:c2300079-2299900
25	39	nr2103	ref|NC_002745.2|:c2300596-2300096
25	40	nr2104	ref|NC_002745.2|:c2300976-2300617
25	41	nr2105	ref|NC_002745.2|:c2301543-2301007
25	42	nr2106	ref|NC_002745.2|:c2301966-2301568
25	43	nr2107	ref|NC_002745.2|:c2302183-2301998
25	44	nr2108	ref|NC_002745.2|:c2302745-2302206
25	45	nr2109	ref|NC_002745.2|:c2303089-2302772
25	46	nr2110	ref|NC_002745.2|:c2303493-2303125
25	47	nr2111	ref|NC_002745.2|:c2303788-2303525
25	48	nr2112	ref|NC_002745.2|:c2304021-2303812
25	49	nr2113	ref|NC_002745.2|:c2304445-2304011
25	50	nr2114	ref|NC_002745.2|:c2305101-2304448
25	51	nr2115	ref|NC_002745.2|:c2305478-2305125
25	52	nr2116	ref|NC_002745.2|:c2305785-2305507
25	53	nr2117	ref|NC_002745.2|:c2306685-2305852
25	54	nr2118	ref|NC_002745.2|:c2306993-2306718
25	55	nr2119	ref|NC_002745.2|:c2307616-2306993
25	56	nr2120	ref|NC_002745.2|:c2308305-2307643
25	57	nr2121	ref|NC_002745.2|:c2308641-2308333
25	58	nr2122	ref|NC_002745.2|:2308998-2309387
25	59	nr2123	ref|NC_002745.2|:c2310912-2309578
25	60	nr2124	ref|NC_002745.2|:c2313160-2311025
25	61	nr2125	ref|NC_002745.2|:2313245-2314222
25	62	nr2126	ref|NC_002745.2|:2314403-2315266
25	63	nr2127	ref|NC_002745.2|:2315550-2315669
25	64	nr2128	ref|NC_002745.2|:c2316829-2315921
25	65	nr2129	ref|NC_002745.2|:2316962-2317126
25	66	nr2130	ref|NC_002745.2|:2317371-2317688
25	67	nr2131	ref|NC_002745.2|:c2321039-2317872
25	68	nr2132	ref|NC_002745.2|:c2322421-2321156
25	69	nr2133	ref|NC_002745.2|:2322810-2323061

25	70	nr2134	ref|NC_002745.2|:c2323817-2323053
25	71	nr2135	ref|NC_002745.2|:2323979-2324419
25	72	nr2136	ref|NC_002745.2|:2324412-2325623
25	73	nr2137	ref|NC_002745.2|:c2326075-2325725
25	74	nr2138	ref|NC_002745.2|:c2327674-2326652
25	75	nr2139	ref|NC_002745.2|:c2328293-2327694
25	76	nr2140	ref|NC_002745.2|:c2328533-2328300
25	77	nr2141	ref|NC_002745.2|:c2328985-2328539
25	78	nr2142	ref|NC_002745.2|:c2329484-2328999
25	79	nr2143	ref|NC_002745.2|:c2330740-2329481
25	80	nr2144	ref|NC_002745.2|:2330803-2331297
25	81	nr2145	ref|NC_002745.2|:c2331805-2331299
25	82	nr2146	ref|NC_002745.2|:c2332840-2331836
25	83	nr2147	ref|NC_002745.2|:c2333615-2333010
25	84	nr2148	ref|NC_002745.2|:c2334287-2333616
25	85	nr2149	ref|NC_002745.2|:c2335083-2334301
25	86	nr2150	ref|NC_002745.2|:2335372-2336169
26	1	nr2151	ref|NC_002745.2|:c2337111-2336341
26	2	nr2152	ref|NC_002745.2|:c2337742-2337188
26	3	nr2153	ref|NC_002745.2|:c2338804-2337863
26	4	nr2154	ref|NC_002745.2|:c2340110-2339202
26	5	nr2155	ref|NC_002745.2|:c2341478-2340324
26	6	nr2156	ref|NC_002745.2|:c2341617-2341525
26	7	nr2157	ref|NC_002745.2|:c2342600-2341695
26	8	nr2158	ref|NC_002745.2|:2342860-2343162
26	9	nr2159	ref|NC_002745.2|:2343176-2343586
26	10	nr2160	ref|NC_002745.2|:2343583-2345298
26	11	nr2161	ref|NC_002745.2|:2345311-2345763
26	12	nr2162	ref|NC_002745.2|:2345756-2346445
26	13	nr2163	ref|NC_002745.2|:2346458-2347072
26	14	nr2164	ref|NC_002745.2|:2347264-2347908
26	15	nr2165	ref|NC_002745.2|:c2348444-2348097
26	16	nr2166	ref|NC_002745.2|:c2349198-2348872
26	17	nr2167	ref|NC_002745.2|:c2350005-2349262
26	18	nr2168	ref|NC_002745.2|:c2351986-2350031
26	19	nr2169	ref|NC_002745.2|:2352596-2353399
26	20	nr2170	ref|NC_002745.2|:c2353545-2353417
26	21	nr2171	ref|NC_002745.2|:c2355233-2353833
26	22	nr2172	ref|NC_002745.2|:c2356410-2355328
26	23	nr2173	ref|NC_002745.2|:2356658-2357080
26	24	nr2174	ref|NC_002745.2|:2357317-2357817
26	25	nr2175	ref|NC_002745.2|:2358178-2359131
26	26	nr2176	ref|NC_002745.2|:c2360347-2359223
26	27	nr2177	ref|NC_002745.2|:c2361434-2360658
26	28	nr2178	ref|NC_002745.2|:c2362390-2361917
26	29	nr2179	ref|NC_002745.2|:c2365344-2362390
26	30	nr2180	ref|NC_002745.2|:c2366894-2365947
26	31	nr2181	ref|NC_002745.2|:c2367824-2367027
26	32	nr2182	ref|NC_002745.2|:2368209-2368901

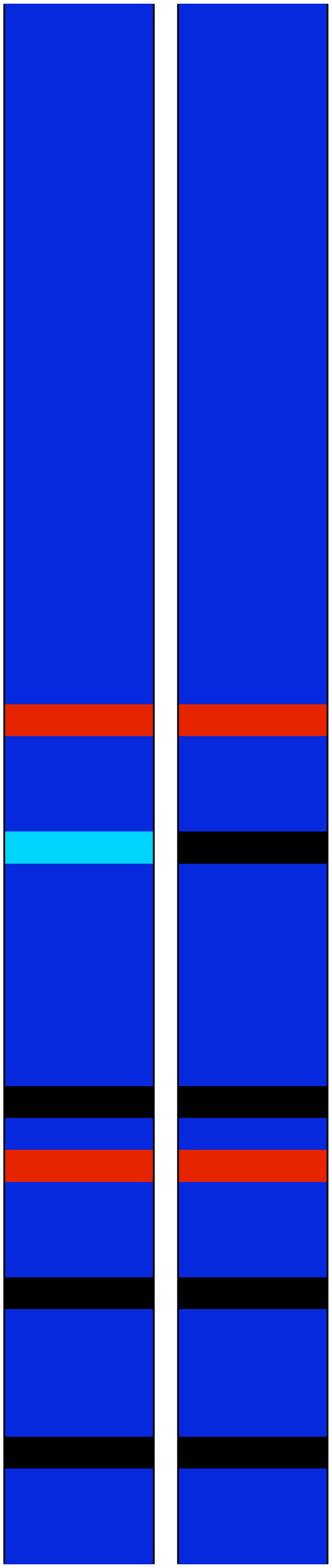

26	33	nr2183	ref|NC_002745.2|:c2369663-2368926
26	34	nr2184	ref|NC_002745.2|:2369712-2370041
26	35	nr2185	ref|NC_002745.2|:2370486-2371358
26	36	nr2186	ref|NC_002745.2|:c2373124-2371745
26	37	nr2187	ref|NC_002745.2|:2373404-2373760
26	38	nr2188	ref|NC_002745.2|:2373757-2373942
26	39	nr2189	ref|NC_002745.2|:c2374229-2374038
26	40	nr2190	ref|NC_002745.2|:c2374875-2374240
26	41	nr2191	ref|NC_002745.2|:c2375867-2374950
26	42	nr2192	ref|NC_002745.2|:2376135-2376665
26	43	nr2193	ref|NC_002745.2|:c2378335-2376731
26	44	nr2194	ref|NC_002745.2|:2378665-2379429
26	45	nr2195	ref|NC_002745.2|:c2379993-2379487
26	46	nr2196	ref|NC_002745.2|:c2381539-2380160
26	47	nr2197	ref|NC_002745.2|:c2382267-2381698
26	48	nr2198	ref|NC_002745.2|:c2383635-2382754
26	49	nr2199	ref|NC_002745.2|:c2384867-2383746
26	50	nr2200	ref|NC_002745.2|:c2386381-2385143
26	51	nr2201	ref|NC_002745.2|:c2388042-2386381
26	52	nr2202	ref|NC_002745.2|:2388202-2389086
26	53	nr2203	ref|NC_002745.2|:2389328-2389747
26	54	nr2204	ref|NC_002745.2|:c2390938-2390003
26	55	nr2205	ref|NC_002745.2|:2391228-2392487
26	56	nr2206	ref|NC_002745.2|:c2393239-2392553
26	57	nr2207	ref|NC_002745.2|:2393413-2394066
26	58	nr2208	ref|NC_002745.2|:c2395321-2394302
26	59	nr2209	ref|NC_002745.2|:c2395677-2395351
26	60	nr2210	ref|NC_002745.2|:c2397127-2395895
26	61	nr2211	ref|NC_002745.2|:c2398019-2397120
26	62	nr2212	ref|NC_002745.2|:c2398870-2398250
26	63	nr2213	ref|NC_002745.2|:2398862-2399053
26	64	nr2214	ref|NC_002745.2|:2399269-2399877
26	65	nr2215	ref|NC_002745.2|:2400210-2401418
26	66	nr2216	ref|NC_002745.2|:c2402515-2401466
26	67	nr2217	ref|NC_002745.2|:c2403493-2402546
26	68	nr2218	ref|NC_002745.2|:2403596-2404081
26	69	nr2219	ref|NC_002745.2|:2404078-2404497
26	70	nr2220	ref|NC_002745.2|:c2405525-2404605
26	71	nr2221	ref|NC_002745.2|:2405733-2406371
26	72	nr2222	ref|NC_002745.2|:c2408548-2406617
26	73	nr2223	ref|NC_002745.2|:c2409208-2408561
26	74	nr2224	ref|NC_002745.2|:2409329-2409883
26	75	nr2225	ref|NC_002745.2|:c2411318-2410110
26	76	nr2226	ref|NC_002745.2|:c2412969-2411587
26	77	nr2227	ref|NC_002745.2|:c2413664-2413209
26	78	nr2228	ref|NC_002745.2|:2413859-2414827
26	79	nr2229	ref|NC_002745.2|:c2415723-2415058
26	80	nr2230	ref|NC_002745.2|:c2416778-2415723
26	81	nr2231	ref|NC_002745.2|:2416914-2417588

26	82	nr2232	ref|NC_002745.2|:2417581-2418954		
26	83	nr2233	ref|NC_002745.2|:2419117-2419560		
26	84	nr2234	ref|NC_002745.2|:2419557-2420012		
26	85	nr2235	ref|NC_002745.2|:c2421613-2420078		
26	86	nr2236	ref|NC_002745.2|:c2423535-2421937		
27	1	nr2237	ref|NC_002745.2|:2423852-2425525		
27	2	nr2238	ref|NC_002745.2|:c2426293-2425679		
27	3	nr2239	ref|NC_002745.2|:c2427122-2426607		
27	4	nr2240	ref|NC_002745.2|:2427313-2428317		
27	5	nr2241	ref|NC_002745.2|:c2428764-2428363		
27	6	nr2242	ref|NC_002745.2|:c2429795-2428800		
27	7	nr2243	ref|NC_002745.2|:c2430498-2430067		
27	8	nr2244	ref|NC_002745.2|:c2431982-2430705		
27	9	nr2245	ref|NC_002745.2|:2432143-2432766		
27	10	nr2246	ref|NC_002745.2|:2432812-2433756		
27	11	nr2247	ref|NC_002745.2|:c2435283-2433841		
27	12	nr2248	ref|NC_002745.2|:2435466-2435849		
27	13	nr2249	ref|NC_002745.2|:2437337-2439442		
27	14	nr2250	ref|NC_002745.2|:2439513-2439935		
27	15	nr2251	ref|NC_002745.2|:2440199-2440555		
27	16	nr2252	ref|NC_002745.2|:c2442150-2440873		
27	17	nr2253	ref|NC_002745.2|:c2442697-2442332		
27	18	nr2254	ref|NC_002745.2|:2443094-2443540		
27	19	nr2255	ref|NC_002745.2|:2443762-2444184		
27	20	nr2256	ref|NC_002745.2|:c2445460-2444291		
27	21	nr2257	ref|NC_002745.2|:2445829-2446743		
27	22	nr2258	ref|NC_002745.2|:2446722-2447462		
27	23	nr2259	ref|NC_002745.2|:c2448373-2447720		
27	24	nr2260	ref|NC_002745.2|:c2449430-2448396		
27	25	nr2261	ref|NC_002745.2|:c2449906-2449454		
27	26	nr2262	ref|NC_002745.2|:c2450603-2449926		
27	27	nr2263	ref|NC_002745.2|:c2451171-2450596		
27	28	nr2264	ref|NC_002745.2|:c2452723-2451164		
27	29	nr2265	ref|NC_002745.2|:c2456402-2452713		
27	30	nr2266	ref|NC_002745.2|:c2457688-2456711		
27	31	nr2267	ref|NC_002745.2|:c2457993-2457679		
27	32	nr2268	ref|NC_002745.2|:c2460402-2457997		
27	33	nr2269	ref|NC_002745.2|:c2461199-2460468		
27	34	nr2270	ref|NC_002745.2|:c2461965-2461429		
27	35	nr2271	ref|NC_002745.2|:c2463023-2462199		
27	36	nr2272	ref|NC_002745.2|:c2463422-2463240		
27	37	nr2273	ref|NC_002745.2|:c2463973-2463506		
27	38	nr2274	ref|NC_002745.2|:c2465706-2464159		
27	39	nr2275	ref|NC_002745.2|:c2466267-2466001		
27	40	nr2276	ref|NC_002745.2|:c2466518-2466267		
27	41	nr2277	ref|NC_002745.2|:c2467390-2466791		
27	42	nr2278	ref|NC_002745.2|:c2467771-2467409		
27	43	nr2279	ref|NC_002745.2|:2468026-2469276		
27	44	nr2280	ref|NC_002745.2|:c2470104-2469373		

27	45	nr2281	ref|NC_002745.2|:c2470595-2470101
27	46	nr2282	ref|NC_002745.2|:c2471580-2470801
27	47	nr2283	ref|NC_002745.2|:c2473140-2471701
27	48	nr2284	ref|NC_002745.2|:c2474258-2473572
27	49	nr2285	ref|NC_002745.2|:c2475451-2474585
27	50	nr2286	ref|NC_002745.2|:2476080-2477390
27	51	nr2287	ref|NC_002745.2|:2477926-2478855
27	52	nr2288	ref|NC_002745.2|:2479422-2480369
27	53	nr2289	ref|NC_002745.2|:2480371-2481348
27	54	nr2290	ref|NC_002745.2|:c2481867-2481400
27	55	nr2291	ref|NC_002745.2|:c2482570-2481878
27	56	nr2292	ref|NC_002745.2|:c2483696-2482581
27	57	nr2293	ref|NC_002745.2|:c2484681-2483674
27	58	nr2294	ref|NC_002745.2|:c2486041-2484683
27	59	nr2295	ref|NC_002745.2|:c2486705-2486019
27	60	nr2296	ref|NC_002745.2|:c2488891-2487158
27	61	nr2297	ref|NC_002745.2|:c2490679-2488916
27	62	nr2298	ref|NC_002745.2|:2491386-2491640
27	63	nr2299	ref|NC_002745.2|:c2492009-2491623
27	64	nr2300	ref|NC_002745.2|:2492277-2493419
27	65	nr2301	ref|NC_002745.2|:2493479-2494138
27	66	nr2302	ref|NC_002745.2|:2494320-2495531
27	67	nr2303	ref|NC_002745.2|:c2496127-2495654
27	68	nr2304	ref|NC_002745.2|:2496240-2496896
27	69	nr2305	ref|NC_002745.2|:c2497765-2496959
27	70	nr2306	ref|NC_002745.2|:2498051-2499091
27	71	nr2307	ref|NC_002745.2|:2499033-2499446
27	72	nr2308	ref|NC_002745.2|:2499585-2501663
27	73	nr2309	ref|NC_002745.2|:c2503633-2501804
27	74	nr2310	ref|NC_002745.2|:2503865-2505361
27	75	nr2311	ref|NC_002745.2|:c2506304-2505453
27	76	nr2312	ref|NC_002745.2|:c2507561-2506626
27	77	nr2313	ref|NC_002745.2|:c2509187-2507787
27	78	nr2314	ref|NC_002745.2|:c2510176-2509481
27	79	nr2315	ref|NC_002745.2|:c2511117-2510176
27	80	nr2316	ref|NC_002745.2|:c2511769-2511134
27	81	nr2317	ref|NC_002745.2|:c2512998-2511766
27	82	nr2318	ref|NC_002745.2|:c2514261-2513662
27	83	nr2319	ref|NC_002745.2|:2514441-2516078
27	84	nr2320	ref|NC_002745.2|:2516387-2517739
27	85	nr2321	ref|NC_002745.2|:c2518988-2517801
27	86	nr2322	ref|NC_002745.2|:c2520160-2519384
28	1	nr2323	ref|NC_002745.2|:c2520815-2520153
28	2	nr2324	ref|NC_002745.2|:c2522139-2521063
28	3	nr2325	ref|NC_002745.2|:c2522760-2522494
28	4	nr2326	ref|NC_002745.2|:c2523017-2522760
28	5	nr2327	ref|NC_002745.2|:2523376-2523831
28	6	nr2328	ref|NC_002745.2|:c2525576-2523999
28	7	nr2329	ref|NC_002745.2|:2525771-2526520

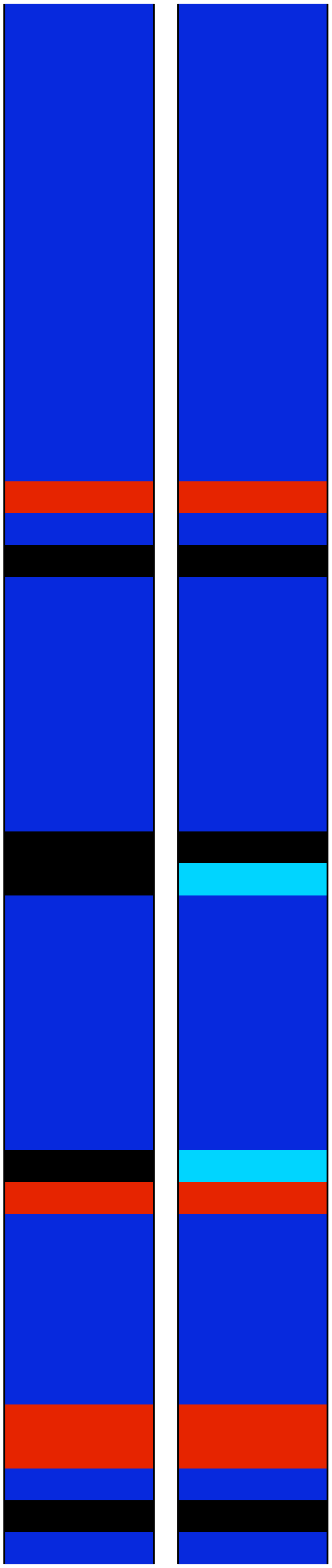

28	8	nr2330	ref|NC_002745.2|:c2526794-2526669
28	9	nr2331	ref|NC_002745.2|:c2528075-2526882
28	10	nr2332	ref|NC_002745.2|:c2528836-2528087
28	11	nr2333	ref|NC_002745.2|:c2529644-2528829
28	12	nr2334	ref|NC_002745.2|:c2530510-2529641
28	13	nr2335	ref|NC_002745.2|:c2531442-2530507
28	14	nr2336	ref|NC_002745.2|:c2533053-2531455
28	15	nr2337	ref|NC_002745.2|:c2534497-2533196
28	16	nr2338	ref|NC_002745.2|:c2535308-2534490
28	17	nr2339	ref|NC_002745.2|:c2536140-2535319
28	18	nr2340	ref|NC_002745.2|:c2537597-2536830
28	19	nr2341	ref|NC_002745.2|:c2538515-2537697
28	20	nr2342	ref|NC_002745.2|:2538750-2540288
28	21	nr2343	ref|NC_002745.2|:c2540823-2540401
28	22	nr2344	ref|NC_002745.2|:c2541694-2541275
28	23	nr2345	ref|NC_002745.2|:c2542131-2541721
28	24	nr2346	ref|NC_002745.2|:c2542910-2542398
28	25	nr2347	ref|NC_002745.2|:c2543765-2543070
28	26	nr2348	ref|NC_002745.2|:2544054-2544335
28	27	nr2349	ref|NC_002745.2|:c2544786-2544595
28	28	nr2350	ref|NC_002745.2|:c2546141-2545347
28	29	nr2351	ref|NC_002745.2|:c2546964-2546677
28	30	nr2352	ref|NC_002745.2|:c2547293-2546967
28	31	nr2353	ref|NC_002745.2|:c2548618-2547293
28	32	nr2354	ref|NC_002745.2|:c2549399-2548605
28	33	nr2355	ref|NC_002745.2|:c2550416-2549643
28	34	nr2356	ref|NC_002745.2|:c2551304-2550528
28	35	nr2357	ref|NC_002745.2|:c2553098-2551608
28	36	nr2358	ref|NC_002745.2|:c2554469-2553138
28	37	nr2359	ref|NC_002745.2|:c2554863-2554471
28	38	nr2360	ref|NC_002745.2|:2555052-2556803
28	39	nr2361	ref|NC_002745.2|:2557087-2557353
28	40	nr2362	ref|NC_002745.2|:2557497-2557805
28	41	nr2363	ref|NC_002745.2|:c2558146-2557913
28	42	nr2364	ref|NC_002745.2|:c2559319-2558243
28	43	nr2365	ref|NC_002745.2|:c2563817-2562483
28	44	nr2366	ref|NC_002745.2|:c2564481-2564122
28	45	nr2367	ref|NC_002745.2|:2564802-2565545
28	46	nr2368	ref|NC_002745.2|:c2566622-2565756
28	47	nr2369	ref|NC_002745.2|:c2568114-2566795
28	48	nr2370	ref|NC_002745.2|:c2571208-2568323
28	49	nr2371	ref|NC_002745.2|:c2575005-2571889
28	50	nr2372	ref|NC_002745.2|:c2575340-2575140
28	51	nr2373	ref|NC_002745.2|:c2576832-2575474
28	52	nr2374	ref|NC_002745.2|:c2578502-2576949
28	53	nr2375	ref|NC_002745.2|:c2579206-2578526
28	54	nr2376	ref|NC_002745.2|:c2580123-2579359
28	55	nr2377	ref|NC_002745.2|:c2580980-2580288
28	56	nr2378	ref|NC_002745.2|:c2581699-2581211

28	57	nr2379	ref|NC_002745.2|:c2583589-2581766
28	58	nr2380	ref|NC_002745.2|:c2585343-2584066
28	59	nr2381	ref|NC_002745.2|:2585890-2586501
28	60	nr2382	ref|NC_002745.2|:2587019-2587714
28	61	nr2383	ref|NC_002745.2|:2587716-2588486
28	62	nr2384	ref|NC_002745.2|:2588859-2590823
28	63	nr2385	ref|NC_002745.2|:2591165-2592244
28	64	nr2386	ref|NC_002745.2|:c2592942-2592349
28	65	nr2387	ref|NC_002745.2|:c2593932-2592973
28	66	nr2388	ref|NC_002745.2|:c2594444-2594010
28	67	nr2389	ref|NC_002745.2|:2594670-2594954
28	68	nr2390	ref|NC_002745.2|:2595125-2595931
28	69	nr2391	ref|NC_002745.2|:c2596723-2596052
28	70	nr2392	ref|NC_002745.2|:2596930-2597985
28	71	nr2393	ref|NC_002745.2|:c2599032-2598232
28	72	nr2394	ref|NC_002745.2|:2599352-2599987
28	73	nr2395	ref|NC_002745.2|:2600002-2600724
28	74	nr2396	ref|NC_002745.2|:c2601506-2600886
28	75	nr2397	ref|NC_002745.2|:2601700-2602191
28	76	nr2398	ref|NC_002745.2|:c2603583-2602684
28	77	nr2399	ref|NC_002745.2|:c2604277-2603597
28	78	nr2400	ref|NC_002745.2|:c2605323-2604280
28	79	nr2401	ref|NC_002745.2|:2605873-2606076
28	80	nr2402	ref|NC_002745.2|:2606268-2607194
28	81	nr2403	ref|NC_002745.2|:c2608243-2607275
28	82	nr2404	ref|NC_002745.2|:c2608720-2608364
28	83	nr2405	ref|NC_002745.2|:c2609172-2608762
28	84	nr2406	ref|NC_002745.2|:c2611618-2609552
28	85	nr2407	ref|NC_002745.2|:c2613658-2611919
28	86	nr2408	ref|NC_002745.2|:c2614398-2613709
29	1	nr2409	ref|NC_002745.2|:c2614786-2614391
29	2	nr2410	ref|NC_002745.2|:c2615886-2614999
29	3	nr2411	ref|NC_002745.2|:2616044-2616262
29	4	nr2412	ref|NC_002745.2|:2616652-2617083
29	5	nr2413	ref|NC_002745.2|:c2618499-2617222
29	6	nr2414	ref|NC_002745.2|:2618754-2619920
29	7	nr2415	ref|NC_002745.2|:c2620606-2620085
29	8	nr2416	ref|NC_002745.2|:2621015-2623120
29	9	nr2417	ref|NC_002745.2|:c2623359-2623189
29	10	nr2418	ref|NC_002745.2|:c2625366-2623372
29	11	nr2419	ref|NC_002745.2|:c2625605-2625378
29	12	nr2420	ref|NC_002745.2|:c2628310-2625821
29	13	nr2421	ref|NC_002745.2|:2628455-2629003
29	14	nr2422	ref|NC_002745.2|:c2630903-2629359
29	15	nr2423	ref|NC_002745.2|:c2631692-2631093
29	16	nr2424	ref|NC_002745.2|:2631931-2632122
29	17	nr2425	ref|NC_002745.2|:2632362-2634770
29	18	nr2426	ref|NC_002745.2|:2635308-2635514
29	19	nr2427	ref|NC_002745.2|:c2636603-2635605

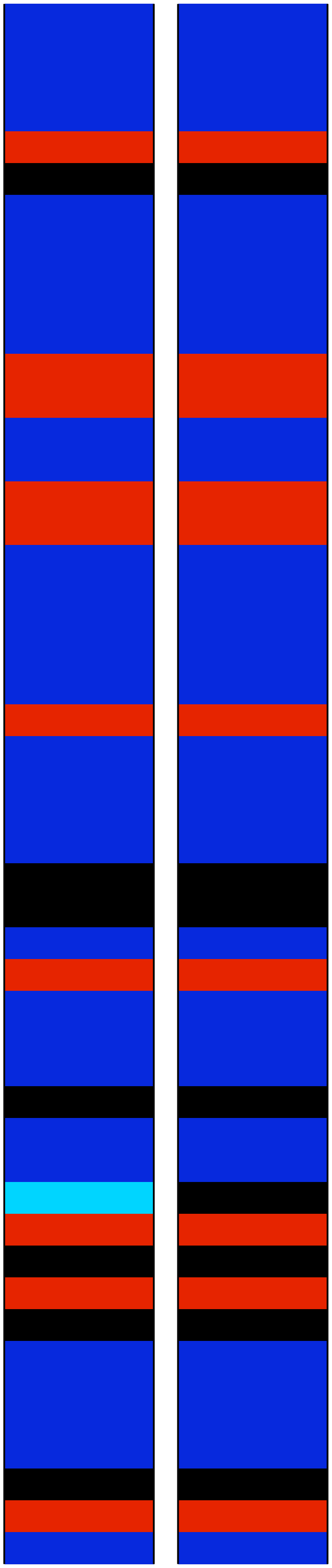

29	20	nr2428	ref|NC_002745.2|:c2637780-2636626	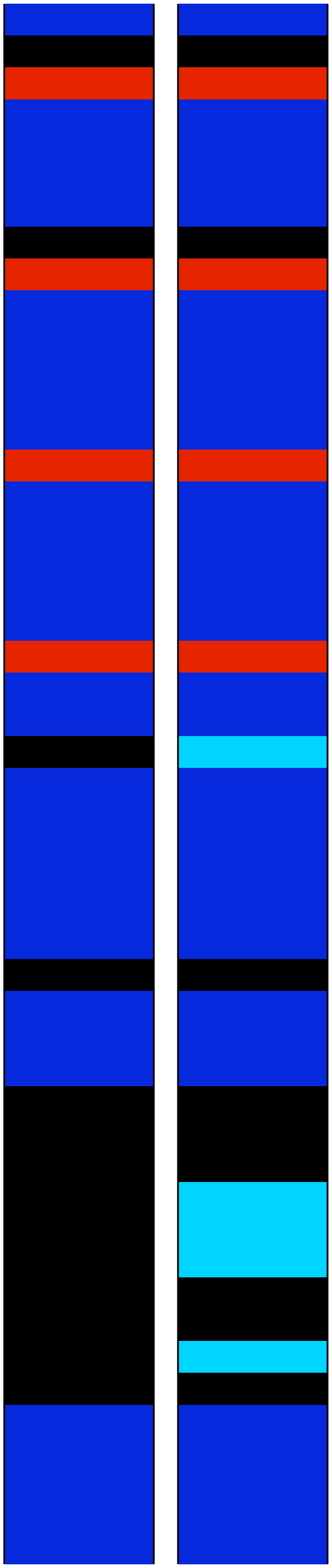
29	21	nr2429	ref|NC_002745.2|:c2639716-2638208	
29	22	nr2430	ref|NC_002745.2|:c2640591-2639728	
29	23	nr2431	ref|NC_002745.2|:c2641755-2640628	
29	24	nr2432	ref|NC_002745.2|:c2643254-2641761	
29	25	nr2433	ref|NC_002745.2|:c2643744-2643247	
29	26	nr2434	ref|NC_002745.2|:c2644677-2643910	
29	27	nr2435	ref|NC_002745.2|:c2646849-2645038	
29	28	nr2436	ref|NC_002745.2|:c2647850-2647362	
29	29	nr2437	ref|NC_002745.2|:c2648827-2648126	
29	30	nr2438	ref|NC_002745.2|:c2650490-2649435	
29	31	nr2439	ref|NC_002745.2|:2650900-2651469	
29	32	nr2440	ref|NC_002745.2|:2651847-2652167	
29	33	nr2441	ref|NC_002745.2|:2652387-2652617	
29	34	nr2442	ref|NC_002745.2|:c2652848-2652690	
29	35	nr2443	ref|NC_002745.2|:c2653296-2652919	
29	36	nr2444	ref|NC_002745.2|:c2654134-2653313	
29	37	nr2445	ref|NC_002745.2|:c2654453-2654154	
29	38	nr2446	ref|NC_002745.2|:2654584-2655141	
29	39	nr2447	ref|NC_002745.2|:2655134-2655838	
29	40	nr2448	ref|NC_002745.2|:2655936-2656946	
29	41	nr2449	ref|NC_002745.2|:2656966-2657796	
29	42	nr2450	ref|NC_002745.2|:c2658833-2657943	
29	43	nr2451	ref|NC_002745.2|:c2660270-2658930	
29	44	nr2452	ref|NC_002745.2|:c2661370-2660264	
29	45	nr2453	ref|NC_002745.2|:c2661999-2661703	
29	46	nr2454	ref|NC_002745.2|:c2662355-2661987	
29	47	nr2455	ref|NC_002745.2|:c2662645-2662370	
29	48	nr2456	ref|NC_002745.2|:c2663855-2662989	
29	49	nr2457	ref|NC_002745.2|:2664073-2665137	
29	50	nr2458	ref|NC_002745.2|:2665292-2665567	
29	51	nr2459	ref|NC_002745.2|:c2666475-2665753	
29	52	nr2460	ref|NC_002745.2|:2666829-2667278	
29	53	nr2461	ref|NC_002745.2|:c2667946-2667359	
29	54	nr2462	ref|NC_002745.2|:2668089-2669771	
29	55	nr2463	ref|NC_002745.2|:c2670504-2669869	
29	56	nr2464	ref|NC_002745.2|:c2671086-2670520	
29	57	nr2465	ref|NC_002745.2|:c2672001-2671339	
29	58	nr2466	ref|NC_002745.2|:2672535-2673266	
29	59	nr2467	ref|NC_002745.2|:c2674174-2673392	
29	60	nr2468	ref|NC_002745.2|:c2674702-2674325	
29	61	nr2469	ref|NC_002745.2|:c2676601-2674709	
29	62	nr2470	ref|NC_002745.2|:c2677683-2676598	
29	63	nr2471	ref|NC_002745.2|:c2678308-2677802	
29	64	nr2472	ref|NC_002745.2|:c2679022-2678651	
29	65	nr2473	ref|NC_002745.2|:c2679887-2679036	
29	66	nr2474	ref|NC_002745.2|:c2680698-2679880	
29	67	nr2475	ref|NC_002745.2|:2680771-2681631	
29	68	nr2476	ref|NC_002745.2|:c2682421-2681717	

29	69	nr2477	ref|NC_002745.2|:c2683658-2682699
29	70	nr2478	ref|NC_002745.2|:2684344-2685792
29	71	nr2479	ref|NC_002745.2|:c2687246-2685873
29	72	nr2480	ref|NC_002745.2|:2687468-2687884
29	73	nr2481	ref|NC_002745.2|:2688007-2688897
29	74	nr2482	ref|NC_002745.2|:c2690587-2689091
29	75	nr2483	ref|NC_002745.2|:2690964-2691212
29	76	nr2484	ref|NC_002745.2|:c2692891-2691272
29	77	nr2485	ref|NC_002745.2|:c2693507-2693064
29	78	nr2486	ref|NC_002745.2|:c2693999-2693787
29	79	nr2487	ref|NC_002745.2|:c2695986-2694277
29	80	nr2488	ref|NC_002745.2|:c2697737-2696247
29	81	nr2489	ref|NC_002745.2|:2697987-2698553
29	82	nr2490	ref|NC_002745.2|:c2700346-2698724
29	83	nr2491	ref|NC_002745.2|:c2701401-2700865
29	84	nr2492	ref|NC_002745.2|:c2703248-2701398
29	85	nr2493	ref|NC_002745.2|:c2704808-2703438
29	86	nr2494	ref|NC_002745.2|:c2705906-2705301
30	1	nr2495	ref|NC_002745.2|:c2707849-2705969
30	2	nr2496	ref|NC_002745.2|:c2708740-2708243
30	3	nr2497	ref|NC_002745.2|:c2712164-2710164
30	4	nr2498	ref|NC_002745.2|:c2712916-2712161
30	5	nr2499	ref|NC_002745.2|:c2713911-2713024
30	6	nr2500	ref|NC_002745.2|:c2714587-2713922
30	7	nr2501	ref|NC_002745.2|:c2714813-2714613
30	8	nr2502	ref|NC_002745.2|:2715011-2716435
30	9	nr2503	ref|NC_002745.2|:c2717191-2716718
30	10	nr2504	ref|NC_002745.2|:c2718179-2717418
30	11	nr2505	ref|NC_002745.2|:c2720928-2718295
30	12	nr2506	ref|NC_002745.2|:c2721982-2721278
30	13	nr2507	ref|NC_002745.2|:c2723049-2722081
30	14	nr2508	ref|NC_002745.2|:c2724469-2723039
30	15	nr2509	ref|NC_002745.2|:c2725570-2724560
30	16	nr2510	ref|NC_002745.2|:c2726838-2725603
30	17	nr2511	ref|NC_002745.2|:c2727634-2727185
30	18	nr2512	ref|NC_002745.2|:c2729530-2728001
30	19	nr2513	ref|NC_002745.2|:c2730481-2729954
30	20	nr2514	ref|NC_002745.2|:2730739-2731197
30	21	nr2515	ref|NC_002745.2|:2731437-2733311
30	22	nr2516	ref|NC_002745.2|:2733387-2735339
30	23	nr2517	ref|NC_002745.2|:2735353-2736291
30	24	nr2518	ref|NC_002745.2|:c2739384-2736403
30	25	nr2519	ref|NC_002745.2|:2739595-2741454
30	26	nr2520	ref|NC_002745.2|:c2742279-2741719
30	27	nr2521	ref|NC_002745.2|:c2744355-2742448
30	28	nr2522	ref|NC_002745.2|:c2745954-2744596
30	29	nr2523	ref|NC_002745.2|:c2747455-2745947
30	30	nr2524	ref|NC_002745.2|:c2749863-2747473
30	31	nr2525	ref|NC_002745.2|:c2750893-2749853

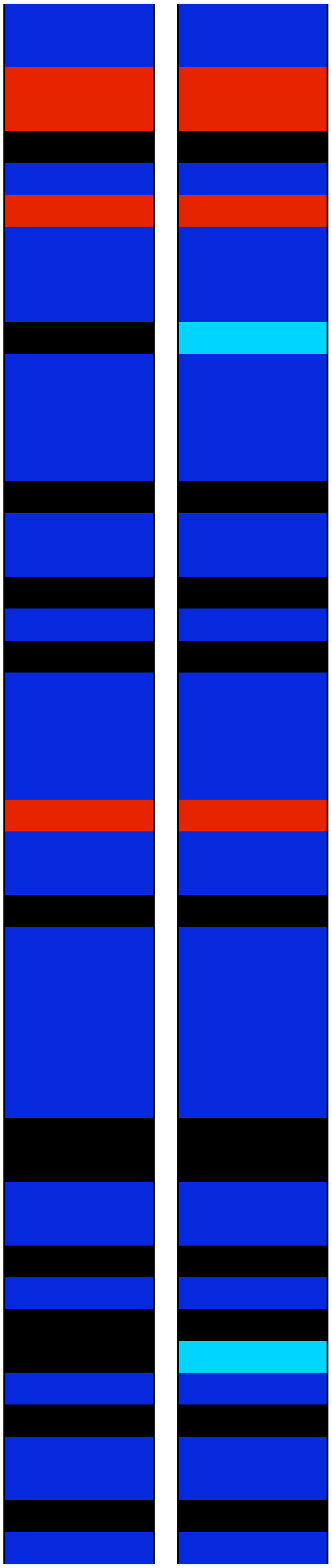

30	32	nr2526	ref|NC_002745.2|:c2752358-2750790
30	33	nr2527	ref|NC_002745.2|:c2753901-2752348
30	34	nr2528	ref|NC_002745.2|:c2755123-2753912
30	35	nr2529	ref|NC_002745.2|:c2762068-2755253
30	36	nr2530	ref|NC_002745.2|:c2763304-2762612
30	37	nr2531	ref|NC_002745.2|:2763624-2763998
30	38	nr2532	ref|NC_002745.2|:2763982-2764287
30	39	nr2533	ref|NC_002745.2|:2764441-2764800
30	40	nr2534	ref|NC_002745.2|:c2765496-2764885
30	41	nr2535	ref|NC_002745.2|:c2766051-2765575
30	42	nr2536	ref|NC_002745.2|:c2766554-2766054
30	43	nr2537	ref|NC_002745.2|:c2767556-2766789
30	44	nr2538	ref|NC_002745.2|:c2768245-2767553
30	45	nr2539	ref|NC_002745.2|:c2768924-2768262
30	46	nr2540	ref|NC_002745.2|:c2770333-2769773
30	47	nr2541	ref|NC_002745.2|:2770497-2771735
30	48	nr2542	ref|NC_002745.2|:2771699-2772004
30	49	nr2543	ref|NC_002745.2|:2772001-2772873
30	50	nr2544	ref|NC_002745.2|:2772860-2773912
30	51	nr2545	ref|NC_002745.2|:2774055-2774165
30	52	nr2546	ref|NC_002745.2|:c2776292-2774247
30	53	nr2547	ref|NC_002745.2|:c2778014-2777382
30	54	nr2548	ref|NC_002745.2|:c2778769-2778011
30	55	nr2549	ref|NC_002745.2|:c2779470-2778766
30	56	nr2550	ref|NC_002745.2|:c2780041-2779463
30	57	nr2551	ref|NC_002745.2|:c2780616-2780038
30	58	nr2552	ref|NC_002745.2|:c2781598-2780585
30	59	nr2553	ref|NC_002745.2|:c2782864-2781614
30	60	nr2554	ref|NC_002745.2|:c2783471-2782857
30	61	nr2555	ref|NC_002745.2|:c2784307-2783489
30	62	nr2556	ref|NC_002745.2|:c2785735-2784611
30	63	nr2557	ref|NC_002745.2|:c2786350-2785853
30	64	nr2558	ref|NC_002745.2|:c2787230-2786397
30	65	nr2559	ref|NC_002745.2|:c2788834-2787227
30	66	nr2560	ref|NC_002745.2|:c2789546-2788992
30	67	nr2561	ref|NC_002745.2|:c2790423-2789575
30	68	nr2562	ref|NC_002745.2|:2790802-2791317
30	69	nr2563	ref|NC_002745.2|:c2792442-2791468
30	70	nr2564	ref|NC_002745.2|:2792683-2793639
30	71	nr2565	ref|NC_002745.2|:2793791-2794429
30	72	nr2566	ref|NC_002745.2|:c2795315-2794548
30	73	nr2567	ref|NC_002745.2|:c2795643-2795305
30	74	nr2568	ref|NC_002745.2|:2795810-2796274
30	75	nr2569	ref|NC_002745.2|:2796512-2797930
30	76	nr2570	ref|NC_002745.2|:c2799252-2798344
30	77	nr2571	ref|NC_002745.2|:2799474-2800049
30	78	nr2572	ref|NC_002745.2|:2800211-2801227
30	79	nr2573	ref|NC_002745.2|:2801464-2802264
30	80	nr2574	ref|NC_002745.2|:c2802910-2802398

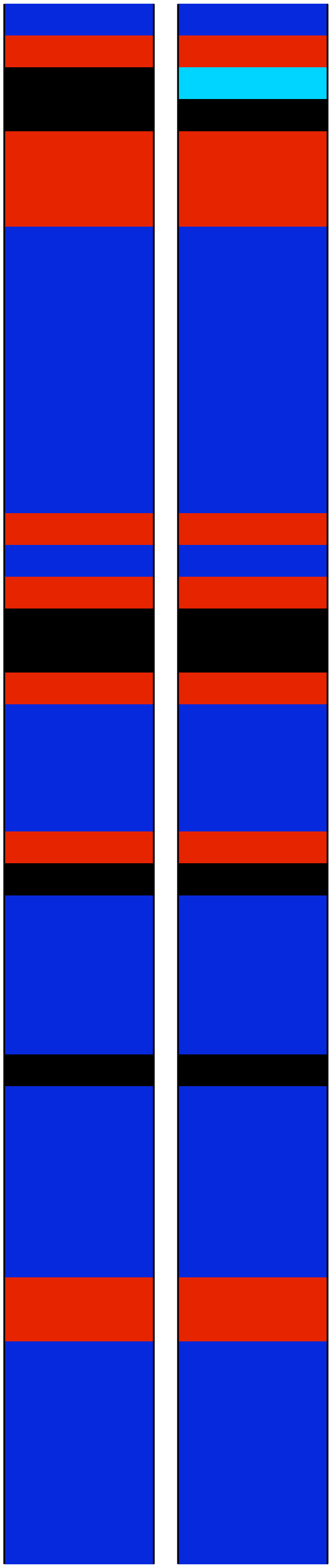

30	81	nr2575	ref|NC_002745.2|:2803193-2803951
30	82	nr2576	ref|NC_002745.2|:2803941-2805821
30	83	nr2577	ref|NC_002745.2|:2805913-2806104
30	84	nr2578	ref|NC_002745.2|:2806496-2806696
30	85	nr2579	ref|NC_002745.2|:c2807383-2806814
30	86	nr2580	ref|NC_002745.2|:c2808038-2807643
31	1	nr2581	ref|NC_002745.2|:c2808459-2808106
31	2	nr2582	ref|NC_002745.2|:2808647-2808796
31	3	nr2583	ref|NC_002745.2|:c2809749-2808910
31	4	nr2584	ref|NC_002745.2|:c2810511-2809792
31	5	nr2585	ref|NC_002745.2|:c2812388-2810511
31	6	nr2586	ref|NC_002745.2|:c2813834-2812455
31	7	nr2587	ref|NC_002745.2|:c2814338-2813991
31	8	nr2588	ref|NC_002745.2|:c2814602-2814465
31	9	nr2589	ref|NC_002758.2|:c37110-36436
31	10	nr2590	ref|NC_002758.2|:c55961-55788
31	11	nr2591	ref|NC_002758.2|:55963-56694
31	12	nr2592	ref|NC_002758.2|:c57602-56820
31	13	nr2593	ref|NC_002758.2|:c58130-57753
31	14	nr2594	ref|NC_002758.2|:c60029-58137
31	15	nr2595	ref|NC_002758.2|:c61111-60026
31	16	nr2596	ref|NC_002758.2|:253951-254607
31	17	nr2597	ref|NC_002758.2|:316523-316723
31	18	nr2598	ref|NC_002758.2|:c382714-382439
31	19	nr2599	ref|NC_002758.2|:426637-427956
31	20	nr2600	ref|NC_002758.2|:c437264-436071
31	21	nr2601	ref|NC_002758.2|:c437491-437291
31	22	nr2602	ref|NC_002758.2|:c438219-437989
31	23	nr2603	ref|NC_002758.2|:c438698-438216
31	24	nr2604	ref|NC_002758.2|:c438937-438827
31	25	nr2605	ref|NC_002758.2|:439169-439522
31	26	nr2606	ref|NC_002758.2|:c441784-439865
31	27	nr2607	ref|NC_002758.2|:c443123-442161
31	28	nr2608	ref|NC_002758.2|:c444129-443107
31	29	nr2609	ref|NC_002758.2|:c446153-444126
31	30	nr2610	ref|NC_002758.2|:c448603-446150
31	31	nr2611	ref|NC_002758.2|:c449024-448587
31	32	nr2612	ref|NC_002758.2|:c449692-449054
31	33	nr2613	ref|NC_002758.2|:c450248-449748
31	34	nr2614	ref|NC_002758.2|:c451088-450309
31	35	nr2615	ref|NC_002758.2|:c451351-451130
31	36	nr2616	ref|NC_002758.2|:c452818-451634
31	37	nr2617	ref|NC_002758.2|:c454403-453000
31	38	nr2618	ref|NC_002758.2|:c455198-454425
31	39	nr2619	ref|NC_002758.2|:c455585-455208
31	40	nr2620	ref|NC_002758.2|:c455920-455606
31	41	nr2621	ref|NC_002758.2|:c457913-456120
31	42	nr2622	ref|NC_002758.2|:c460099-457910
31	43	nr2623	ref|NC_002758.2|:c461423-460233

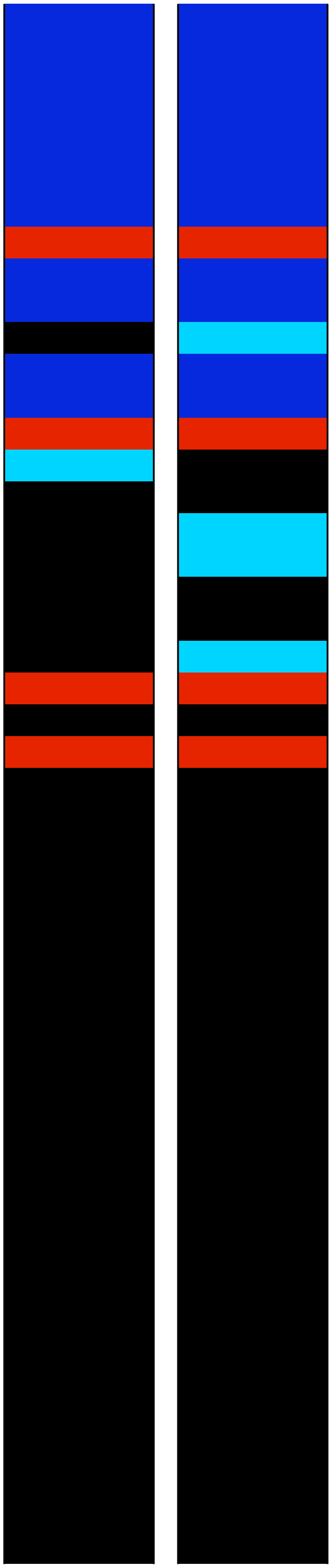

31	44	nr2624	ref|NC_002758.2|:c703868-702549
31	45	nr2625	ref|NC_002758.2|:c869568-868462
31	46	nr2626	ref|NC_002758.2|:c870158-869574
31	47	nr2627	ref|NC_002758.2|:870376-870585
31	48	nr2628	ref|NC_002758.2|:870621-870848
31	49	nr2629	ref|NC_002758.2|:870845-870991
31	50	nr2630	ref|NC_002758.2|:870984-871193
31	51	nr2631	ref|NC_002758.2|:871196-871513
31	52	nr2632	ref|NC_002758.2|:871580-872449
31	53	nr2633	ref|NC_002758.2|:872466-873935
31	54	nr2634	ref|NC_002758.2|:874137-874583
31	55	nr2635	ref|NC_002758.2|:874585-874869
31	56	nr2636	ref|NC_002758.2|:874866-875507
31	57	nr2637	ref|NC_002758.2|:875957-876298
31	58	nr2638	ref|NC_002758.2|:876310-876888
31	59	nr2639	ref|NC_002758.2|:876906-877124
31	60	nr2640	ref|NC_002758.2|:877175-877702
31	61	nr2641	ref|NC_002758.2|:878043-878612
31	62	nr2642	ref|NC_002758.2|:878889-879857
31	63	nr2643	ref|NC_002758.2|:880082-880276
31	64	nr2644	ref|NC_002758.2|:880821-881735
31	65	nr2645	ref|NC_002758.2|:881793-882641
31	66	nr2646	ref|NC_002758.2|:883017-883157
31	67	nr2647	ref|NC_002758.2|:c918556-917507
31	68	nr2648	ref|NC_002758.2|:918668-918847
31	69	nr2649	ref|NC_002758.2|:c919759-918827
31	70	nr2650	ref|NC_002758.2|:c920255-919791
31	71	nr2651	ref|NC_002758.2|:c920597-920268
31	72	nr2652	ref|NC_002758.2|:920758-920949
31	73	nr2653	ref|NC_002758.2|:c921380-921171
31	74	nr2654	ref|NC_002758.2|:921437-922216
31	75	nr2655	ref|NC_002758.2|:922217-922441
31	76	nr2656	ref|NC_002758.2|:922481-922624
31	77	nr2657	ref|NC_002758.2|:c923293-922646
31	78	nr2658	ref|NC_002758.2|:923364-923585
31	79	nr2659	ref|NC_002758.2|:924409-924630
31	80	nr2660	ref|NC_002758.2|:924623-924745
31	81	nr2661	ref|NC_002758.2|:925247-925672
31	82	nr2662	ref|NC_002758.2|:925683-926234
31	83	nr2663	ref|NC_002758.2|:926235-926909
31	84	nr2664	ref|NC_002758.2|:c927330-927049
31	85	nr2665	ref|NC_002758.2|:927395-928126
31	86	nr2666	ref|NC_002758.2|:928139-928924
32	1	nr2667	ref|NC_002758.2|:928921-929079
32	2	nr2668	ref|NC_002758.2|:929092-929313
32	3	nr2669	ref|NC_002758.2|:929323-929727
32	4	nr2670	ref|NC_002758.2|:929919-930290
32	5	nr2671	ref|NC_002758.2|:931368-931574
32	6	nr2672	ref|NC_002758.2|:931571-931765

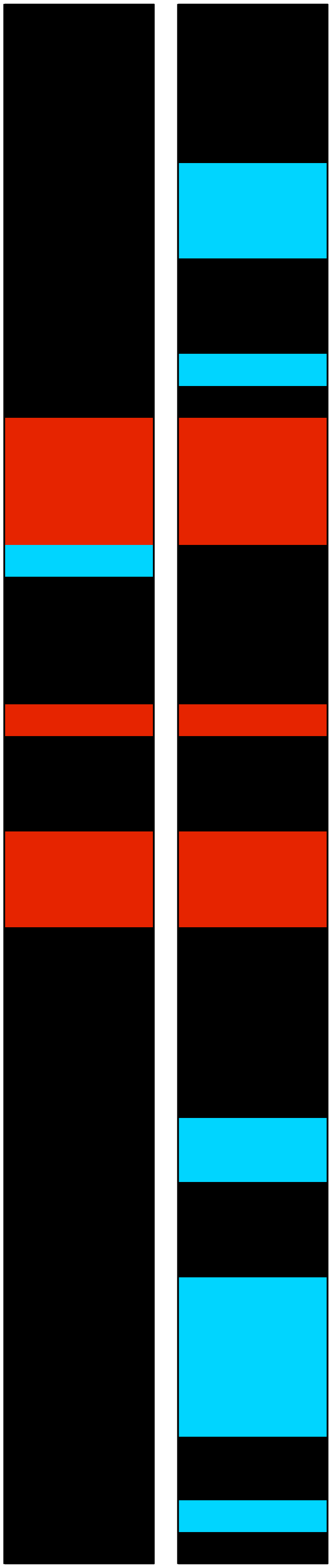

32	7	nr2673	ref|NC_002758.2|:931762-931965
32	8	nr2674	ref|NC_002758.2|:931958-932194
32	9	nr2675	ref|NC_002758.2|:932184-932570
32	10	nr2676	ref|NC_002758.2|:932570-932743
32	11	nr2677	ref|NC_002758.2|:932801-932890
32	12	nr2678	ref|NC_002758.2|:932905-933324
32	13	nr2679	ref|NC_002758.2|:933511-933951
32	14	nr2680	ref|NC_002758.2|:933938-935215
32	15	nr2681	ref|NC_002758.2|:935226-936761
32	16	nr2682	ref|NC_002758.2|:936768-937763
32	17	nr2683	ref|NC_002758.2|:937836-938006
32	18	nr2684	ref|NC_002758.2|:938141-938755
32	19	nr2685	ref|NC_002758.2|:938769-939077
32	20	nr2686	ref|NC_002758.2|:939209-939742
32	21	nr2687	ref|NC_002758.2|:939764-940051
32	22	nr2688	ref|NC_002758.2|:940060-940392
32	23	nr2689	ref|NC_002758.2|:940389-940691
32	24	nr2690	ref|NC_002758.2|:940691-941038
32	25	nr2691	ref|NC_002758.2|:941050-941433
32	26	nr2692	ref|NC_002758.2|:941452-942033
32	27	nr2693	ref|NC_002758.2|:942095-942460
32	28	nr2694	ref|NC_002758.2|:942580-942834
32	29	nr2695	ref|NC_002758.2|:942851-946315
32	30	nr2696	ref|NC_002758.2|:946328-947275
32	31	nr2697	ref|NC_002758.2|:947284-949185
32	32	nr2698	ref|NC_002758.2|:949200-951110
32	33	nr2699	ref|NC_002758.2|:951110-952933
32	34	nr2700	ref|NC_002758.2|:952933-953310
32	35	nr2701	ref|NC_002758.2|:953314-953487
32	36	nr2702	ref|NC_002758.2|:953528-953827
32	37	nr2703	ref|NC_002758.2|:953964-955862
32	38	nr2704	ref|NC_002758.2|:955875-957113
32	39	nr2705	ref|NC_002758.2|:957119-957514
32	40	nr2706	ref|NC_002758.2|:957570-958007
32	41	nr2707	ref|NC_002758.2|:957988-959433
32	42	nr2708	ref|NC_002758.2|:959700-959828
32	43	nr2709	ref|NC_002758.2|:959899-960009
32	44	nr2710	ref|NC_002758.2|:960529-961161
32	45	nr2711	ref|NC_002758.2|:961154-961585
32	46	nr2712	ref|NC_002758.2|:970190-971509
32	47	nr2713	ref|NC_002758.2|:1070500-1070718
32	48	nr2714	ref|NC_002758.2|:1215412-1216731
32	49	nr2715	ref|NC_002758.2|:1394584-1394922
32	50	nr2716	ref|NC_002758.2|:c1762255-1762082
32	51	nr2717	ref|NC_002758.2|:1813637-1814956
32	52	nr2718	ref|NC_002758.2|:1839628-1840947
32	53	nr2719	ref|NC_002758.2|:1886366-1886638
32	54	nr2720	ref|NC_002758.2|:c1887363-1886755
32	55	nr2721	ref|NC_002758.2|:1917398-1917748

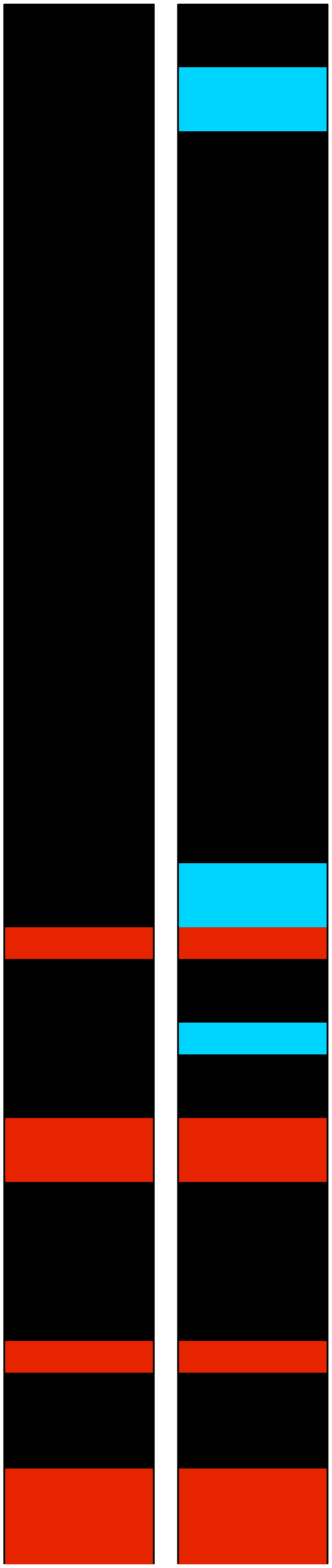

32	56	nr2722	ref|NC_002758.2|:1917924-1918172
32	57	nr2723	ref|NC_002758.2|:1918091-1918870
32	58	nr2724	ref|NC_002758.2|:c1933740-1933171
32	59	nr2725	ref|NC_002758.2|:c1934016-1933762
32	60	nr2726	ref|NC_002758.2|:1992601-1993920
32	61	nr2727	ref|NC_002758.2|:c2110455-2110282
32	62	nr2728	ref|NC_002758.2|:c2111061-2110492
32	63	nr2729	ref|NC_002758.2|:c2111260-2111018
32	64	nr2730	ref|NC_002758.2|:c2111486-2111250
32	65	nr2731	ref|NC_002758.2|:c2111844-2111479
32	66	nr2732	ref|NC_002758.2|:c2112101-2111859
32	67	nr2733	ref|NC_002758.2|:c2118622-2118362
32	68	nr2734	ref|NC_002758.2|:c2118929-2118627
32	69	nr2735	ref|NC_002758.2|:2119549-2120184
32	70	nr2736	ref|NC_002758.2|:2121395-2121934
32	71	nr2737	ref|NC_002758.2|:c2122218-2121958
32	72	nr2738	ref|NC_002758.2|:c2122474-2122232
32	73	nr2739	ref|NC_002758.2|:2123366-2124223
32	74	nr2740	ref|NC_002758.2|:2124550-2125014
32	75	nr2741	ref|NC_002758.2|:c2139146-2138934
32	76	nr2742	ref|NC_002758.2|:c2212165-2210846
32	77	nr2743	ref|NC_002758.2|:c2310073-2309600
32	78	nr2744	ref|NC_002758.2|:c2506666-2505347
32	79	nr2745	ref|NC_002758.2|:2506776-2506868
32	80	nr2746	ref|NC_002758.2|:c2631487-2630897
32	81	nr2747	ref|NC_002758.2|:2861041-2861643
32	82	nr2748	ref|NC_002774.1|:315-1250
32	83	nr2749	ref|NC_002774.1|:c2000-1731
32	84	nr2750	ref|NC_002774.1|:2209-4131
32	85	nr2751	ref|NC_002774.1|:4405-4818
32	86	nr2752	ref|NC_002774.1|:4895-5128
33	1	nr2753	ref|NC_002774.1|:c5881-5177
33	2	nr2754	ref|NC_002774.1|:c6730-6032
33	3	nr2755	ref|NC_002774.1|:6564-6833
33	4	nr2756	ref|NC_002774.1|:6894-6992
33	5	nr2757	ref|NC_002774.1|:c8080-7169
33	6	nr2758	ref|NC_002774.1|:8314-8433
33	7	nr2759	ref|NC_002774.1|:c8738-8499
33	8	nr2760	ref|NC_002774.1|:c8647-8546
33	9	nr2761	ref|NC_002774.1|:c9482-8808
33	10	nr2762	ref|NC_002774.1|:c10194-9520
33	11	nr2763	ref|NC_002774.1|:c11107-10328
33	12	nr2764	ref|NC_002774.1|:c11946-11389
33	13	nr2765	ref|NC_002774.1|:12241-12657
33	14	nr2766	ref|NC_002774.1|:c13535-12699
33	15	nr2767	ref|NC_002774.1|:c13824-13522
33	16	nr2768	ref|NC_002774.1|:c13987-13670
33	17	nr2769	ref|NC_002774.1|:c14651-14037
33	18	nr2770	ref|NC_002774.1|:c15455-14781

33	19	nr2771	ref|NC_002774.1|:15986-16651
33	20	nr2772	ref|NC_002774.1|:16762-17094
33	21	nr2773	ref|NC_002774.1|:c18663-17224
33	22	nr2774	ref|NC_002774.1|:c19068-18664
33	23	nr2775	ref|NC_002774.1|:c19397-19113
33	24	nr2776	ref|NC_002774.1|:c20598-20260
33	25	nr2777	ref|NC_002774.1|:c21415-20849
33	26	nr2778	ref|NC_002774.1|:21593-23137
33	27	nr2779	ref|NC_002774.1|:23342-23902
33	28	nr2780	ref|NC_002774.1|:c24982-24221
33	29	nr2781	ref|NC_002951.2|:22263-22355
33	30	nr2782	ref|NC_002951.2|:37550-37780
33	31	nr2783	ref|NC_002951.2|:42707-42967
33	32	nr2784	ref|NC_002951.2|:c44481-42958
33	33	nr2785	ref|NC_002951.2|:c45543-45451
33	34	nr2786	ref|NC_002951.2|:51771-53348
33	35	nr2787	ref|NC_002951.2|:c54756-53428
33	36	nr2788	ref|NC_002951.2|:c55842-54775
33	37	nr2789	ref|NC_002951.2|:56265-56996
33	38	nr2790	ref|NC_002951.2|:57212-61858
33	39	nr2791	ref|NC_002951.2|:61982-63598
33	40	nr2792	ref|NC_002951.2|:c65269-63758
33	41	nr2793	ref|NC_002951.2|:68407-68499
33	42	nr2794	ref|NC_002951.2|:c70937-70485
33	43	nr2795	ref|NC_002951.2|:c76316-76215
33	44	nr2796	ref|NC_002951.2|:c79750-78530
33	45	nr2797	ref|NC_002951.2|:c80204-79743
33	46	nr2798	ref|NC_002951.2|:80406-81266
33	47	nr2799	ref|NC_002951.2|:c82154-81621
33	48	nr2800	ref|NC_002951.2|:82254-82988
33	49	nr2801	ref|NC_002951.2|:90216-90986
33	50	nr2802	ref|NC_002951.2|:91050-91214
33	51	nr2803	ref|NC_002951.2|:91227-91823
33	52	nr2804	ref|NC_002951.2|:97875-97982
33	53	nr2805	ref|NC_002951.2|:125074-125481
33	54	nr2806	ref|NC_002951.2|:200496-200597
33	55	nr2807	ref|NC_002951.2|:211184-212026
33	56	nr2808	ref|NC_002951.2|:212028-212951
33	57	nr2809	ref|NC_002951.2|:212938-214050
33	58	nr2810	ref|NC_002951.2|:242952-243146
33	59	nr2811	ref|NC_002951.2|:248021-248161
33	60	nr2812	ref|NC_002951.2|:c263496-263371
33	61	nr2813	ref|NC_002951.2|:c269736-269602
33	62	nr2814	ref|NC_002951.2|:307286-307939
33	63	nr2815	ref|NC_002951.2|:307973-308560
33	64	nr2816	ref|NC_002951.2|:326535-326975
33	65	nr2817	ref|NC_002951.2|:327492-327983
33	66	nr2818	ref|NC_002951.2|:328193-328876
33	67	nr2819	ref|NC_002951.2|:329462-329962

33	68	nr2820	ref|NC_002951.2|:329974-330474
33	69	nr2821	ref|NC_002951.2|:330485-330985
33	70	nr2822	ref|NC_002951.2|:331507-332007
33	71	nr2823	ref|NC_002951.2|:332529-333017
33	72	nr2824	ref|NC_002951.2|:c355990-354785
33	73	nr2825	ref|NC_002951.2|:c356897-356184
33	74	nr2826	ref|NC_002951.2|:358218-358436
33	75	nr2827	ref|NC_002951.2|:358451-358759
33	76	nr2828	ref|NC_002951.2|:c358915-358763
33	77	nr2829	ref|NC_002951.2|:359972-360217
33	78	nr2830	ref|NC_002951.2|:c360551-360186
33	79	nr2831	ref|NC_002951.2|:361220-361411
33	80	nr2832	ref|NC_002951.2|:c361640-361389
33	81	nr2833	ref|NC_002951.2|:361695-361958
33	82	nr2834	ref|NC_002951.2|:362222-362539
33	83	nr2835	ref|NC_002951.2|:364751-365551
33	84	nr2836	ref|NC_002951.2|:365551-365907
33	85	nr2837	ref|NC_002951.2|:365904-367145
33	86	nr2838	ref|NC_002951.2|:367142-367357
34	1	nr2839	ref|NC_002951.2|:367591-368016
34	2	nr2840	ref|NC_002951.2|:368422-368607
34	3	nr2841	ref|NC_002951.2|:368608-368865
34	4	nr2842	ref|NC_002951.2|:369493-369699
34	5	nr2843	ref|NC_002951.2|:369702-370103
34	6	nr2844	ref|NC_002951.2|:370100-370447
34	7	nr2845	ref|NC_002951.2|:372375-372575
34	8	nr2846	ref|NC_002951.2|:372532-372882
34	9	nr2847	ref|NC_002951.2|:372895-373332
34	10	nr2848	ref|NC_002951.2|:373489-373803
34	11	nr2849	ref|NC_002951.2|:373932-374237
34	12	nr2850	ref|NC_002951.2|:374227-375918
34	13	nr2851	ref|NC_002951.2|:375923-377161
34	14	nr2852	ref|NC_002951.2|:377145-377918
34	15	nr2853	ref|NC_002951.2|:377930-379093
34	16	nr2854	ref|NC_002951.2|:379162-379440
34	17	nr2855	ref|NC_002951.2|:379452-379784
34	18	nr2856	ref|NC_002951.2|:379781-380182
34	19	nr2857	ref|NC_002951.2|:380183-380578
34	20	nr2858	ref|NC_002951.2|:380613-381254
34	21	nr2859	ref|NC_002951.2|:381346-381801
34	22	nr2860	ref|NC_002951.2|:381859-382209
34	23	nr2861	ref|NC_002951.2|:382251-382409
34	24	nr2862	ref|NC_002951.2|:382423-388623
34	25	nr2863	ref|NC_002951.2|:388623-389447
34	26	nr2864	ref|NC_002951.2|:389456-391039
34	27	nr2865	ref|NC_002951.2|:391039-391329
34	28	nr2866	ref|NC_002951.2|:391345-393255
34	29	nr2867	ref|NC_002951.2|:393255-394721
34	30	nr2868	ref|NC_002951.2|:394721-395110

34	31	nr2869	ref|NC_002951.2|:395103-395267
34	32	nr2870	ref|NC_002951.2|:395748-396050
34	33	nr2871	ref|NC_002951.2|:398286-398408
34	34	nr2872	ref|NC_002951.2|:400845-400937
34	35	nr2873	ref|NC_002951.2|:418756-419052
34	36	nr2874	ref|NC_002951.2|:473486-473623
34	37	nr2875	ref|NC_002951.2|:477049-478605
34	38	nr2876	ref|NC_002951.2|:484842-485618
34	39	nr2877	ref|NC_002951.2|:500802-500918
34	40	nr2878	ref|NC_002951.2|:c541939-541784
34	41	nr2879	ref|NC_002951.2|:549748-549879
34	42	nr2880	ref|NC_002951.2|:570262-570354
34	43	nr2881	ref|NC_002951.2|:627661-627834
34	44	nr2882	ref|NC_002951.2|:673534-673635
34	45	nr2883	ref|NC_002951.2|:675514-676140
34	46	nr2884	ref|NC_002951.2|:676266-676907
34	47	nr2885	ref|NC_002951.2|:676995-677612
34	48	nr2886	ref|NC_002951.2|:677687-678316
34	49	nr2887	ref|NC_002951.2|:679951-680580
34	50	nr2888	ref|NC_002951.2|:681033-681662
34	51	nr2889	ref|NC_002951.2|:681777-682427
34	52	nr2890	ref|NC_002951.2|:682464-683099
34	53	nr2891	ref|NC_002951.2|:683334-683960
34	54	nr2892	ref|NC_002951.2|:690028-690135
34	55	nr2893	ref|NC_002951.2|:c700617-700498
34	56	nr2894	ref|NC_002951.2|:728216-728308
34	57	nr2895	ref|NC_002951.2|:809587-809748
34	58	nr2896	ref|NC_002951.2|:814958-815224
34	59	nr2897	ref|NC_002951.2|:819062-819166
34	60	nr2898	ref|NC_002951.2|:841589-841693
34	61	nr2899	ref|NC_002951.2|:843126-843233
34	62	nr2900	ref|NC_002951.2|:c863351-863205
34	63	nr2901	ref|NC_002951.2|:c877671-877525
34	64	nr2902	ref|NC_002951.2|:879027-879182
34	65	nr2903	ref|NC_002951.2|:c904711-903491
34	66	nr2904	ref|NC_002951.2|:c906280-905552
34	67	nr2905	ref|NC_002951.2|:c906781-906350
34	68	nr2906	ref|NC_002951.2|:c907600-907268
34	69	nr2907	ref|NC_002951.2|:907793-908056
34	70	nr2908	ref|NC_002951.2|:908049-908321
34	71	nr2909	ref|NC_002951.2|:918606-918806
34	72	nr2910	ref|NC_002951.2|:926954-927091
34	73	nr2911	ref|NC_002951.2|:937560-937670
34	74	nr2912	ref|NC_002951.2|:c968399-968265
34	75	nr2913	ref|NC_002951.2|:997602-997700
34	76	nr2914	ref|NC_002951.2|:1036544-1036639
34	77	nr2915	ref|NC_002951.2|:1050523-1050810
34	78	nr2916	ref|NC_002951.2|:1050816-1052297
34	79	nr2917	ref|NC_002951.2|:c1054355-1054224

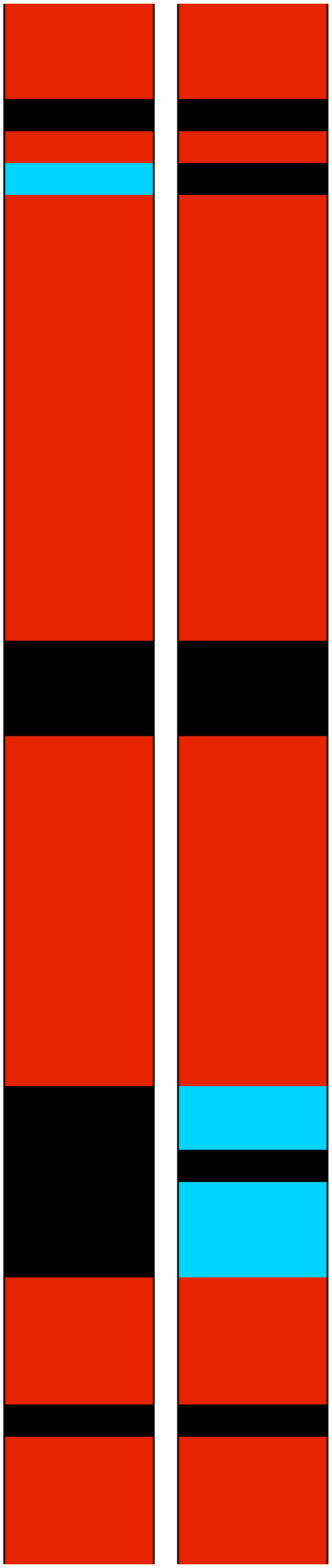

34	80	nr2918	ref|NC_002951.2|:1056491-1056595
34	81	nr2919	ref|NC_002951.2|:1139025-1139228
34	82	nr2920	ref|NC_002951.2|:c1166060-1165965
34	83	nr2921	ref|NC_002951.2|:1174698-1174805
34	84	nr2922	ref|NC_002951.2|:1178161-1178406
34	85	nr2923	ref|NC_002951.2|:1181706-1181852
34	86	nr2924	ref|NC_002951.2|:c1182313-1182065
35	1	nr2925	ref|NC_002951.2|:1182290-1182421
35	2	nr2926	ref|NC_002951.2|:1192217-1192351
35	3	nr2927	ref|NC_002951.2|:1192408-1192542
35	4	nr2928	ref|NC_002951.2|:c1255289-1255191
35	5	nr2929	ref|NC_002951.2|:1285396-1285509
35	6	nr2930	ref|NC_002951.2|:1348415-1348612
35	7	nr2931	ref|NC_002951.2|:1349301-1349525
35	8	nr2932	ref|NC_002951.2|:1350726-1350836
35	9	nr2933	ref|NC_002951.2|:1351961-1352065
35	10	nr2934	ref|NC_002951.2|:1352098-1352193
35	11	nr2935	ref|NC_002951.2|:1352650-1353075
35	12	nr2936	ref|NC_002951.2|:1353196-1353444
35	13	nr2937	ref|NC_002951.2|:1353574-1353675
35	14	nr2938	ref|NC_002951.2|:1353721-1353987
35	15	nr2939	ref|NC_002951.2|:1354324-1354458
35	16	nr2940	ref|NC_002951.2|:1354549-1354839
35	17	nr2941	ref|NC_002951.2|:1354850-1355044
35	18	nr2942	ref|NC_002951.2|:1355094-1355435
35	19	nr2943	ref|NC_002951.2|:1367008-1367115
35	20	nr2944	ref|NC_002951.2|:c1377515-1377411
35	21	nr2945	ref|NC_002951.2|:1382822-1382920
35	22	nr2946	ref|NC_002951.2|:c1453277-1452885
35	23	nr2947	ref|NC_002951.2|:1527488-1527613
35	24	nr2948	ref|NC_002951.2|:c1560228-1560112
35	25	nr2949	ref|NC_002951.2|:c1566806-1565892
35	26	nr2950	ref|NC_002951.2|:c1567027-1566821
35	27	nr2951	ref|NC_002951.2|:c1572048-1571179
35	28	nr2952	ref|NC_002951.2|:1593168-1593347
35	29	nr2953	ref|NC_002951.2|:c1595076-1594978
35	30	nr2954	ref|NC_002951.2|:c1609591-1609250
35	31	nr2955	ref|NC_002951.2|:c1610237-1609647
35	32	nr2956	ref|NC_002951.2|:c1611290-1610244
35	33	nr2957	ref|NC_002951.2|:c1613127-1611280
35	34	nr2958	ref|NC_002951.2|:c1614490-1613132
35	35	nr2959	ref|NC_002951.2|:c1617039-1614544
35	36	nr2960	ref|NC_002951.2|:c1617457-1617074
35	37	nr2961	ref|NC_002951.2|:c1617729-1617469
35	38	nr2962	ref|NC_002951.2|:c1618789-1617734
35	39	nr2963	ref|NC_002951.2|:c1619941-1618850
35	40	nr2964	ref|NC_002951.2|:c1620418-1620116
35	41	nr2965	ref|NC_002951.2|:c1620752-1620432
35	42	nr2966	ref|NC_002951.2|:c1621187-1620903

35	43	nr2967	ref|NC_002951.2|:c1797368-1797270
35	44	nr2968	ref|NC_002951.2|:1805783-1805923
35	45	nr2969	ref|NC_002951.2|:c1871374-1871255
35	46	nr2970	ref|NC_002951.2|:1880090-1880485
35	47	nr2971	ref|NC_002951.2|:c1895344-1894859
35	48	nr2972	ref|NC_002951.2|:c1904985-1904344
35	49	nr2973	ref|NC_002951.2|:c1905462-1905286
35	50	nr2974	ref|NC_002951.2|:c1905856-1905473
35	51	nr2975	ref|NC_002951.2|:c1907983-1907606
35	52	nr2976	ref|NC_002951.2|:c1909064-1908504
35	53	nr2977	ref|NC_002951.2|:c1912319-1909269
35	54	nr2978	ref|NC_002951.2|:c1912616-1912386
35	55	nr2979	ref|NC_002951.2|:c1916512-1916378
35	56	nr2980	ref|NC_002951.2|:c1918163-1917447
35	57	nr2981	ref|NC_002951.2|:c1924961-1924200
35	58	nr2982	ref|NC_002951.2|:c1925650-1924958
35	59	nr2983	ref|NC_002951.2|:c1927046-1925673
35	60	nr2984	ref|NC_002951.2|:c1927574-1927056
35	61	nr2985	ref|NC_002951.2|:c1931820-1928827
35	62	nr2986	ref|NC_002951.2|:c1932028-1931885
35	63	nr2987	ref|NC_002951.2|:1932920-1934239
35	64	nr2988	ref|NC_002951.2|:1937391-1937930
35	65	nr2989	ref|NC_002951.2|:1941836-1942063
35	66	nr2990	ref|NC_002951.2|:c1945607-1945497
35	67	nr2991	ref|NC_002951.2|:1965661-1966023
35	68	nr2992	ref|NC_002951.2|:c2011174-2011064
35	69	nr2993	ref|NC_002951.2|:c2014272-2014180
35	70	nr2994	ref|NC_002951.2|:c2036307-2036134
35	71	nr2995	ref|NC_002951.2|:c2041665-2041543
35	72	nr2996	ref|NC_002951.2|:c2046854-2046681
35	73	nr2997	ref|NC_002951.2|:c2059472-2059374
35	74	nr2998	ref|NC_002951.2|:c2073662-2073219
35	75	nr2999	ref|NC_002951.2|:c2074205-2073768
35	76	nr3000	ref|NC_002951.2|:c2075113-2074523
35	77	nr3001	ref|NC_002951.2|:2083704-2083844
35	78	nr3002	ref|NC_002951.2|:c2129151-2129035
35	79	nr3003	ref|NC_002951.2|:c2133584-2133474
35	80	nr3004	ref|NC_002951.2|:c2147078-2146986
35	81	nr3005	ref|NC_002951.2|:c2203302-2202166
35	82	nr3006	ref|NC_002951.2|:c2269004-2268888
35	83	nr3007	ref|NC_002951.2|:c2271877-2271269
35	84	nr3008	ref|NC_002951.2|:c2283866-2283267
35	85	nr3009	ref|NC_002951.2|:c2284672-2283899
35	86	nr3010	ref|NC_002951.2|:c2285937-2284801
36	1	nr3011	ref|NC_002951.2|:c2286729-2285938
36	2	nr3012	ref|NC_002951.2|:c2314372-2314241
36	3	nr3013	ref|NC_002951.2|:2324826-2324996
36	4	nr3014	ref|NC_002951.2|:c2360443-2360192
36	5	nr3015	ref|NC_002951.2|:2367992-2368306

36	6	nr3016	ref|NC_002951.2|:c2393847-2393734
36	7	nr3017	ref|NC_002951.2|:c2397725-2397627
36	8	nr3018	ref|NC_002951.2|:c2411125-2410997
36	9	nr3019	ref|NC_002951.2|:2430972-2431064
36	10	nr3020	ref|NC_002951.2|:c2440576-2440454
36	11	nr3021	ref|NC_002951.2|:c2467296-2467198
36	12	nr3022	ref|NC_002951.2|:2473830-2473934
36	13	nr3023	ref|NC_002951.2|:2479557-2479682
36	14	nr3024	ref|NC_002951.2|:c2487708-2487565
36	15	nr3025	ref|NC_002951.2|:c2514014-2513895
36	16	nr3026	ref|NC_002951.2|:c2515000-2514830
36	17	nr3027	ref|NC_002951.2|:c2524192-2523998
36	18	nr3028	ref|NC_002951.2|:c2537156-2537025
36	19	nr3029	ref|NC_002951.2|:c2537985-2537620
36	20	nr3030	ref|NC_002951.2|:c2542242-2541823
36	21	nr3031	ref|NC_002951.2|:2545770-2545892
36	22	nr3032	ref|NC_002951.2|:c2549948-2549163
36	23	nr3033	ref|NC_002951.2|:c2569041-2568919
36	24	nr3034	ref|NC_002951.2|:c2585253-2584090
36	25	nr3035	ref|NC_002951.2|:c2585710-2585246
36	26	nr3036	ref|NC_002951.2|:c2601921-2601751
36	27	nr3037	ref|NC_002951.2|:c2602081-2601983
36	28	nr3038	ref|NC_002951.2|:c2617514-2617257
36	29	nr3039	ref|NC_002951.2|:c2630122-2629955
36	30	nr3040	ref|NC_002951.2|:2656321-2656428
36	31	nr3041	ref|NC_002951.2|:2689522-2689617
36	32	nr3042	ref|NC_002951.2|:c2691143-2690457
36	33	nr3043	ref|NC_002951.2|:c2697965-2697870
36	34	nr3044	ref|NC_002951.2|:2700784-2700876
36	35	nr3045	ref|NC_002951.2|:c2702289-2702176
36	36	nr3046	ref|NC_002951.2|:c2709337-2709236
36	37	nr3047	ref|NC_002951.2|:c2755354-2755244
36	38	nr3048	ref|NC_002951.2|:c2767097-2767032
36	39	nr3049	ref|NC_002951.2|:c2770100-2769888
36	40	nr3050	ref|NC_002951.2|:2799000-2799536
36	41	nr3051	ref|NC_002951.2|:c2800093-2799596
36	42	nr3052	ref|NC_002951.2|:2800172-2800522
36	43	nr3053	ref|NC_002951.2|:2800551-2800742
36	44	nr3054	ref|NC_002952.2|:c37077-36403
36	45	nr3055	ref|NC_002952.2|:49263-49466
36	46	nr3056	ref|NC_002952.2|:c55356-54694
36	47	nr3057	ref|NC_002952.2|:55890-56621
36	48	nr3058	ref|NC_002952.2|:c57529-56747
36	49	nr3059	ref|NC_002952.2|:c58057-57680
36	50	nr3060	ref|NC_002952.2|:c59956-58064
36	51	nr3061	ref|NC_002952.2|:c61038-59953
36	52	nr3062	ref|NC_002952.2|:87145-89265
36	53	nr3063	ref|NC_002952.2|:89496-89795
36	54	nr3064	ref|NC_002952.2|:90831-91346

36	55	nr3065	ref|NC_002952.2|:c92298-91351
36	56	nr3066	ref|NC_002952.2|:92484-92858
36	57	nr3067	ref|NC_002952.2|:93145-94848
36	58	nr3068	ref|NC_002952.2|:94841-95881
36	59	nr3069	ref|NC_002952.2|:96110-96208
36	60	nr3070	ref|NC_002952.2|:96312-96470
36	61	nr3071	ref|NC_002952.2|:c96909-96778
36	62	nr3072	ref|NC_002952.2|:c98280-97309
36	63	nr3073	ref|NC_002952.2|:c98704-98333
36	64	nr3074	ref|NC_002952.2|:c100085-98724
36	65	nr3075	ref|NC_002952.2|:101081-101656
36	66	nr3076	ref|NC_002952.2|:102126-102599
36	67	nr3077	ref|NC_002952.2|:175909-176988
36	68	nr3078	ref|NC_002952.2|:176981-178375
36	69	nr3079	ref|NC_002952.2|:178372-178929
36	70	nr3080	ref|NC_002952.2|:178938-180176
36	71	nr3081	ref|NC_002952.2|:269254-269925
36	72	nr3082	ref|NC_002952.2|:279841-279987
36	73	nr3083	ref|NC_002952.2|:c291171-291010
36	74	nr3084	ref|NC_002952.2|:291336-291461
36	75	nr3085	ref|NC_002952.2|:303513-305804
36	76	nr3086	ref|NC_002952.2|:334649-334915
36	77	nr3087	ref|NC_002952.2|:334938-335333
36	78	nr3088	ref|NC_002952.2|:335355-337025
36	79	nr3089	ref|NC_002952.2|:337685-338140
36	80	nr3090	ref|NC_002952.2|:338160-338591
36	81	nr3091	ref|NC_002952.2|:c410063-409344
36	82	nr3092	ref|NC_002952.2|:c412174-411551
36	83	nr3093	ref|NC_002952.2|:412269-412487
36	84	nr3094	ref|NC_002952.2|:412912-413058
36	85	nr3095	ref|NC_002952.2|:413116-413499
36	86	nr3096	ref|NC_002952.2|:418894-419547
37	1	nr3097	ref|NC_002952.2|:422460-422861
37	2	nr3098	ref|NC_002952.2|:422979-423491
37	3	nr3099	ref|NC_002952.2|:c425456-424449
37	4	nr3100	ref|NC_002952.2|:430027-430134
37	5	nr3101	ref|NC_002952.2|:437550-437651
37	6	nr3102	ref|NC_002952.2|:c448947-448771
37	7	nr3103	ref|NC_002952.2|:c450871-450752
37	8	nr3104	ref|NC_002952.2|:461364-462920
37	9	nr3105	ref|NC_002952.2|:477501-477629
37	10	nr3106	ref|NC_002952.2|:509760-509825
37	11	nr3107	ref|NC_002952.2|:c520212-519424
37	12	nr3108	ref|NC_002952.2|:c520991-520236
37	13	nr3109	ref|NC_002952.2|:c565974-565186
37	14	nr3110	ref|NC_002952.2|:c566753-565998
37	15	nr3111	ref|NC_002952.2|:586205-586348
37	16	nr3112	ref|NC_002952.2|:643752-643949
37	17	nr3113	ref|NC_002952.2|:655260-655529

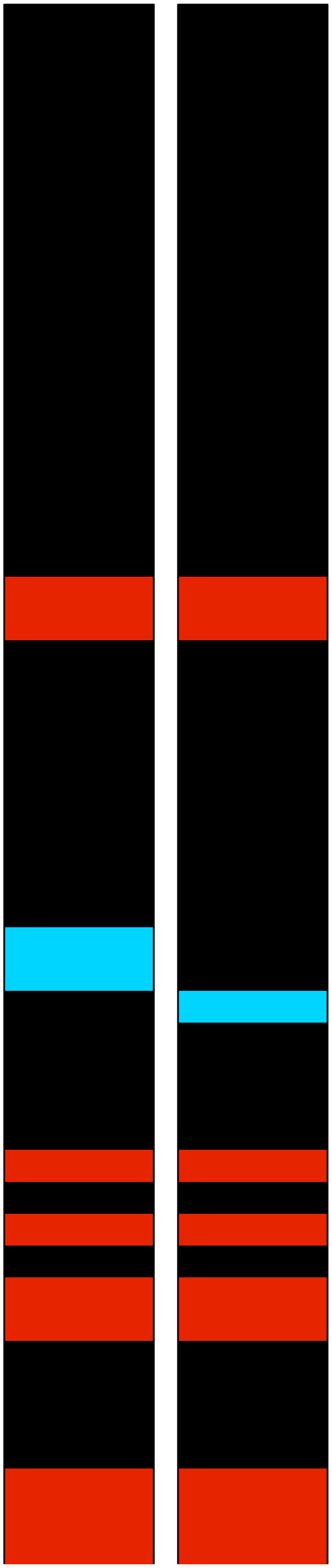

37	18	nr3114	ref|NC_002952.2|:c686150-685002
37	19	nr3115	ref|NC_002952.2|:734448-735122
37	20	nr3116	ref|NC_002952.2|:c735369-735157
37	21	nr3117	ref|NC_002952.2|:736085-736399
37	22	nr3118	ref|NC_002952.2|:736399-737688
37	23	nr3119	ref|NC_002952.2|:738308-738466
37	24	nr3120	ref|NC_002952.2|:738481-738774
37	25	nr3121	ref|NC_002952.2|:738849-740783
37	26	nr3122	ref|NC_002952.2|:740787-741098
37	27	nr3123	ref|NC_002952.2|:741110-741736
37	28	nr3124	ref|NC_002952.2|:742098-743744
37	29	nr3125	ref|NC_002952.2|:c745432-745289
37	30	nr3126	ref|NC_002952.2|:745474-746334
37	31	nr3127	ref|NC_002952.2|:746421-746762
37	32	nr3128	ref|NC_002952.2|:746836-746967
37	33	nr3129	ref|NC_002952.2|:c747081-746977
37	34	nr3130	ref|NC_002952.2|:748482-748598
37	35	nr3131	ref|NC_002952.2|:750428-750637
37	36	nr3132	ref|NC_002952.2|:c751142-750819
37	37	nr3133	ref|NC_002952.2|:751371-751463
37	38	nr3134	ref|NC_002952.2|:c752586-751765
37	39	nr3135	ref|NC_002952.2|:752711-753706
37	40	nr3136	ref|NC_002952.2|:c754616-754041
37	41	nr3137	ref|NC_002952.2|:754883-756928
37	42	nr3138	ref|NC_002952.2|:756943-758376
37	43	nr3139	ref|NC_002952.2|:c762632-760452
37	44	nr3140	ref|NC_002952.2|:c762990-762625
37	45	nr3141	ref|NC_002952.2|:c763605-763228
37	46	nr3142	ref|NC_002952.2|:763700-764374
37	47	nr3143	ref|NC_002952.2|:815730-815864
37	48	nr3144	ref|NC_002952.2|:866923-868557
37	49	nr3145	ref|NC_002952.2|:884556-885170
37	50	nr3146	ref|NC_002952.2|:973598-975244
37	51	nr3147	ref|NC_002952.2|:c999020-998073
37	52	nr3148	ref|NC_002952.2|:c1005037-1004249
37	53	nr3149	ref|NC_002952.2|:c1005815-1005258
37	54	nr3150	ref|NC_002952.2|:1006111-1007757
37	55	nr3151	ref|NC_002952.2|:1027295-1028020
37	56	nr3152	ref|NC_002952.2|:1028022-1028873
37	57	nr3153	ref|NC_002952.2|:1051201-1051308
37	58	nr3154	ref|NC_002952.2|:1155096-1155203
37	59	nr3155	ref|NC_002952.2|:1178795-1179019
37	60	nr3156	ref|NC_002952.2|:c1179594-1179346
37	61	nr3157	ref|NC_002952.2|:1182701-1184347
37	62	nr3158	ref|NC_002952.2|:c1216624-1215677
37	63	nr3159	ref|NC_002952.2|:c1368361-1367414
37	64	nr3160	ref|NC_002952.2|:1370725-1370976
37	65	nr3161	ref|NC_002952.2|:1371704-1371901
37	66	nr3162	ref|NC_002952.2|:1372328-1372645

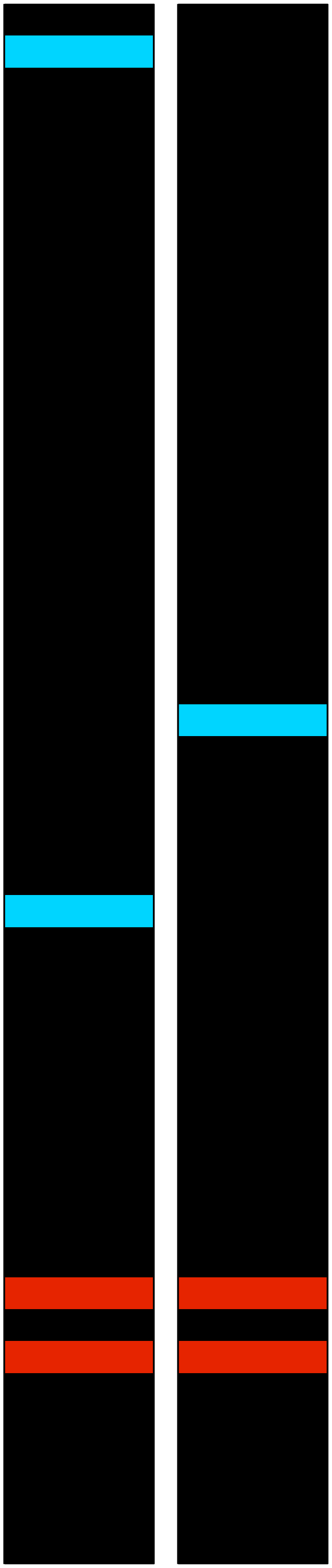

37	67	nr3163	ref|NC_002952.2|:1372972-1373169
37	68	nr3164	ref|NC_002952.2|:1374311-1374436
37	69	nr3165	ref|NC_002952.2|:1375435-1375572
37	70	nr3166	ref|NC_002952.2|:1375775-1375975
37	71	nr3167	ref|NC_002952.2|:1376455-1376565
37	72	nr3168	ref|NC_002952.2|:1408278-1409033
37	73	nr3169	ref|NC_002952.2|:1409057-1409845
37	74	nr3170	ref|NC_002952.2|:c1433078-1431432
37	75	nr3171	ref|NC_002952.2|:1493459-1494406
37	76	nr3172	ref|NC_002952.2|:c1551194-1551024
37	77	nr3173	ref|NC_002952.2|:c1618627-1618337
37	78	nr3174	ref|NC_002952.2|:c1621415-1618968
37	79	nr3175	ref|NC_002952.2|:c1621742-1621671
37	80	nr3176	ref|NC_002952.2|:c1621888-1621736
37	81	nr3177	ref|NC_002952.2|:c1623382-1623134
37	82	nr3178	ref|NC_002952.2|:c1623997-1623806
37	83	nr3179	ref|NC_002952.2|:c1624643-1624401
37	84	nr3180	ref|NC_002952.2|:c1624858-1624658
37	85	nr3181	ref|NC_002952.2|:c1625519-1625118
37	86	nr3182	ref|NC_002952.2|:c1625704-1625519
38	1	nr3183	ref|NC_002952.2|:c1627678-1625717
38	2	nr3184	ref|NC_002952.2|:c1628294-1627737
38	3	nr3185	ref|NC_002952.2|:c1629486-1628320
38	4	nr3186	ref|NC_002952.2|:c1629845-1629483
38	5	nr3187	ref|NC_002952.2|:c1630183-1629860
38	6	nr3188	ref|NC_002952.2|:c1630423-1630262
38	7	nr3189	ref|NC_002952.2|:c1630941-1630726
38	8	nr3190	ref|NC_002952.2|:c1632206-1632009
38	9	nr3191	ref|NC_002952.2|:c1632678-1632529
38	10	nr3192	ref|NC_002952.2|:c1633378-1633154
38	11	nr3193	ref|NC_002952.2|:1633540-1633920
38	12	nr3194	ref|NC_002952.2|:1634418-1634852
38	13	nr3195	ref|NC_002952.2|:1634881-1635276
38	14	nr3196	ref|NC_002952.2|:1635366-1635488
38	15	nr3197	ref|NC_002952.2|:c1636098-1635475
38	16	nr3198	ref|NC_002952.2|:c1659768-1658869
38	17	nr3199	ref|NC_002952.2|:c1743909-1743730
38	18	nr3200	ref|NC_002952.2|:c1793564-1793445
38	19	nr3201	ref|NC_002952.2|:1887056-1887151
38	20	nr3202	ref|NC_002952.2|:c1909329-1908514
38	21	nr3203	ref|NC_002952.2|:c1910764-1909322
38	22	nr3204	ref|NC_002952.2|:c1911329-1910736
38	23	nr3205	ref|NC_002952.2|:c1911973-1911593
38	24	nr3206	ref|NC_002952.2|:c1913720-1911963
38	25	nr3207	ref|NC_002952.2|:1913827-1914672
38	26	nr3208	ref|NC_002952.2|:1914968-1915084
38	27	nr3209	ref|NC_002952.2|:1951988-1952608
38	28	nr3210	ref|NC_002952.2|:c1971031-1969664
38	29	nr3211	ref|NC_002952.2|:c1972239-1971793

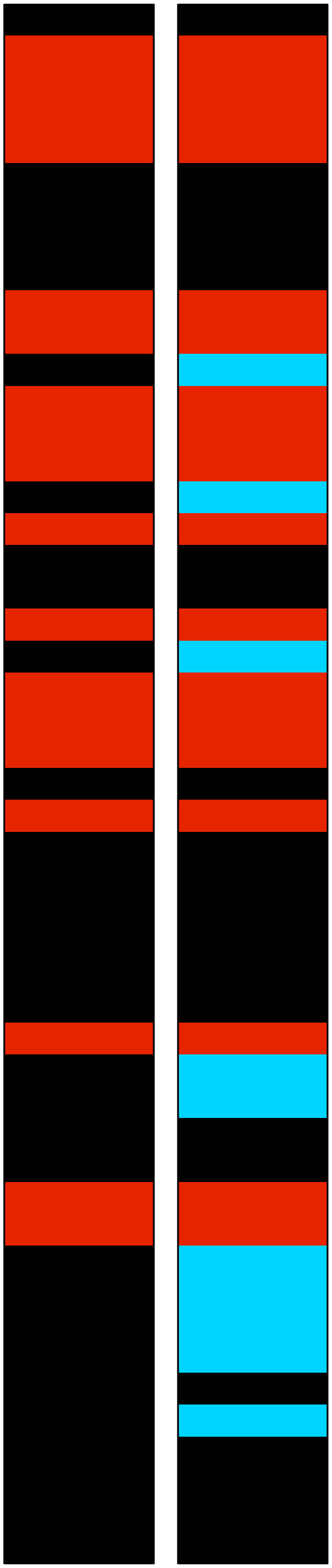

38	30	nr3212	ref|NC_002952.2|:c1973630-1973184
38	31	nr3213	ref|NC_002952.2|:c1974073-1973627
38	32	nr3214	ref|NC_002952.2|:1975037-1977457
38	33	nr3215	ref|NC_002952.2|:c1978619-1978170
38	34	nr3216	ref|NC_002952.2|:1978662-1980272
38	35	nr3217	ref|NC_002952.2|:1980287-1980586
38	36	nr3218	ref|NC_002952.2|:c1982665-1980848
38	37	nr3219	ref|NC_002952.2|:c1983856-1982702
38	38	nr3220	ref|NC_002952.2|:c1989135-1988419
38	39	nr3221	ref|NC_002952.2|:1990279-1990524
38	40	nr3222	ref|NC_002952.2|:1990763-1990894
38	41	nr3223	ref|NC_002952.2|:1990993-1991508
38	42	nr3224	ref|NC_002952.2|:1991755-1993101
38	43	nr3225	ref|NC_002952.2|:1993688-1993924
38	44	nr3226	ref|NC_002952.2|:2010299-2010487
38	45	nr3227	ref|NC_002952.2|:c2121622-2119976
38	46	nr3228	ref|NC_002952.2|:2125437-2125697
38	47	nr3229	ref|NC_002952.2|:c2142645-2142484
38	48	nr3230	ref|NC_002952.2|:c2143353-2143072
38	49	nr3231	ref|NC_002952.2|:c2150942-2150829
38	50	nr3232	ref|NC_002952.2|:c2152846-2152640
38	51	nr3233	ref|NC_002952.2|:c2153600-2153418
38	52	nr3234	ref|NC_002952.2|:c2154007-2153834
38	53	nr3235	ref|NC_002952.2|:c2161066-2160965
38	54	nr3236	ref|NC_002952.2|:2166046-2166387
38	55	nr3237	ref|NC_002952.2|:c2175988-2175647
38	56	nr3238	ref|NC_002952.2|:c2176274-2175978
38	57	nr3239	ref|NC_002952.2|:c2176459-2176271
38	58	nr3240	ref|NC_002952.2|:c2213447-2213031
38	59	nr3241	ref|NC_002952.2|:2254140-2254412
38	60	nr3242	ref|NC_002952.2|:c2293941-2293279
38	61	nr3243	ref|NC_002952.2|:c2306190-2306110
38	62	nr3244	ref|NC_002952.2|:2307489-2307581
38	63	nr3245	ref|NC_002952.2|:c2327010-2325364
38	64	nr3246	ref|NC_002952.2|:c2373759-2373634
38	65	nr3247	ref|NC_002952.2|:c2384255-2384073
38	66	nr3248	ref|NC_002952.2|:c2386784-2385333
38	67	nr3249	ref|NC_002952.2|:2391853-2392608
38	68	nr3250	ref|NC_002952.2|:2392632-2393420
38	69	nr3251	ref|NC_002952.2|:2498195-2498326
38	70	nr3252	ref|NC_002952.2|:c2520432-2519869
38	71	nr3253	ref|NC_002952.2|:2520566-2521294
38	72	nr3254	ref|NC_002952.2|:2521291-2522373
38	73	nr3255	ref|NC_002952.2|:c2545380-2543734
38	74	nr3256	ref|NC_002952.2|:c2645848-2645435
38	75	nr3257	ref|NC_002952.2|:c2646285-2645866
38	76	nr3258	ref|NC_002952.2|:c2676083-2675466
38	77	nr3259	ref|NC_002952.2|:c2677060-2676080
38	78	nr3260	ref|NC_002952.2|:2677159-2678571

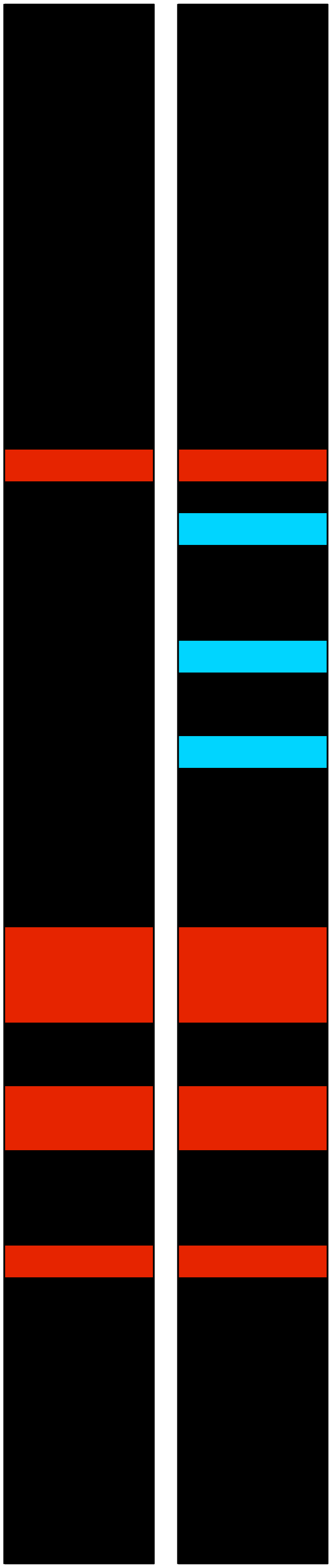

38	79	nr3261	ref|NC_002952.2|:2797873-2798820
38	80	nr3262	ref|NC_002952.2|:c2880339-2876788
38	81	nr3263	ref|NC_002952.2|:c2892961-2892212
38	82	nr3264	ref|NC_002952.2|:c2893783-2892980
38	83	nr3265	ref|NC_002953.3|:c37584-34546
38	84	nr3266	ref|NC_002953.3|:c38898-37588
38	85	nr3267	ref|NC_002953.3|:c40678-38891
38	86	nr3268	ref|NC_002953.3|:c41087-40866
39	1	nr3269	ref|NC_002953.3|:c49740-48253
39	2	nr3270	ref|NC_002953.3|:49912-50616
39	3	nr3271	ref|NC_002953.3|:50640-51482
39	4	nr3272	ref|NC_002953.3|:51926-52234
39	5	nr3273	ref|NC_002953.3|:52820-53458
39	6	nr3274	ref|NC_002953.3|:54144-54773
39	7	nr3275	ref|NC_002953.3|:c55880-55452
39	8	nr3276	ref|NC_002953.3|:55960-56889
39	9	nr3277	ref|NC_002953.3|:60231-60956
39	10	nr3278	ref|NC_002953.3|:82989-83753
39	11	nr3279	ref|NC_002953.3|:85462-85773
39	12	nr3280	ref|NC_002953.3|:165436-165702
39	13	nr3281	ref|NC_002953.3|:c209601-209413
39	14	nr3282	ref|NC_002953.3|:322246-322746
39	15	nr3283	ref|NC_002953.3|:323268-323768
39	16	nr3284	ref|NC_002953.3|:414952-416058
39	17	nr3285	ref|NC_002953.3|:416502-416687
39	18	nr3286	ref|NC_002953.3|:c417076-416684
39	19	nr3287	ref|NC_002953.3|:c417577-417098
39	20	nr3288	ref|NC_002953.3|:c417960-417703
39	21	nr3289	ref|NC_002953.3|:418503-418835
39	22	nr3290	ref|NC_002953.3|:419940-421766
39	23	nr3291	ref|NC_002953.3|:c422726-422304
39	24	nr3292	ref|NC_002953.3|:c423599-423051
39	25	nr3293	ref|NC_002953.3|:424095-424502
39	26	nr3294	ref|NC_002953.3|:424549-425031
39	27	nr3295	ref|NC_002953.3|:425231-425920
39	28	nr3296	ref|NC_002953.3|:c426201-426094
39	29	nr3297	ref|NC_002953.3|:433738-434436
39	30	nr3298	ref|NC_002953.3|:438971-440527
39	31	nr3299	ref|NC_002953.3|:440520-441779
39	32	nr3300	ref|NC_002953.3|:445152-445964
39	33	nr3301	ref|NC_002953.3|:985377-985553
39	34	nr3302	ref|NC_002953.3|:c985789-985550
39	35	nr3303	ref|NC_002953.3|:986351-986512
39	36	nr3304	ref|NC_002953.3|:994876-995400
39	37	nr3305	ref|NC_002953.3|:c996101-995895
39	38	nr3306	ref|NC_002953.3|:1342863-1343075
39	39	nr3307	ref|NC_002953.3|:1343195-1343464
39	40	nr3308	ref|NC_002953.3|:1343477-1343599
39	41	nr3309	ref|NC_002953.3|:1856477-1856656

39	42	nr3310	ref|NC_002953.3|:c1893497-1892253
39	43	nr3311	ref|NC_002953.3|:c1897571-1897428
39	44	nr3312	ref|NC_002953.3|:1929082-1929345
39	45	nr3313	ref|NC_002953.3|:c1972228-1972112
39	46	nr3314	ref|NC_002953.3|:c2042871-2042749
39	47	nr3315	ref|NC_002953.3|:c2043377-2042931
39	48	nr3316	ref|NC_002953.3|:c2044395-2043442
39	49	nr3317	ref|NC_002953.3|:c2044776-2044396
39	50	nr3318	ref|NC_002953.3|:c2045150-2044773
39	51	nr3319	ref|NC_002953.3|:c2045482-2045150
39	52	nr3320	ref|NC_002953.3|:c2045804-2045472
39	53	nr3321	ref|NC_002953.3|:c2045971-2045813
39	54	nr3322	ref|NC_002953.3|:c2047254-2046007
39	55	nr3323	ref|NC_002953.3|:c2047938-2047342
39	56	nr3324	ref|NC_002953.3|:c2049169-2047919
39	57	nr3325	ref|NC_002953.3|:c2049375-2049175
39	58	nr3326	ref|NC_002953.3|:c2051083-2049389
39	59	nr3327	ref|NC_002953.3|:c2051553-2051086
39	60	nr3328	ref|NC_002953.3|:c2052035-2051682
39	61	nr3329	ref|NC_002953.3|:c2052494-2052042
39	62	nr3330	ref|NC_002953.3|:c2053079-2052609
39	63	nr3331	ref|NC_002953.3|:c2053952-2053302
39	64	nr3332	ref|NC_002953.3|:c2054111-2053959
39	65	nr3333	ref|NC_002953.3|:c2064265-2064041
39	66	nr3334	ref|NC_002953.3|:2279611-2279736
39	67	nr3335	ref|NC_002953.3|:c2280113-2280027
39	68	nr3336	ref|NC_002953.3|:2348207-2348416
39	69	nr3337	ref|NC_002953.3|:2419459-2420706
39	70	nr3338	ref|NC_002953.3|:2438104-2438223
39	71	nr3339	ref|NC_002953.3|:c2539153-2538338
39	72	nr3340	ref|NC_002953.3|:c2540085-2539267
39	73	nr3341	ref|NC_002953.3|:c2572361-2572158
39	74	nr3342	ref|NC_002953.3|:2579459-2579536
39	75	nr3343	ref|NC_002953.3|:2639344-2639547
39	76	nr3344	ref|NC_002953.3|:c2773821-2773657
39	77	nr3345	ref|NC_002953.3|:c2783274-2783191
39	78	nr3346	ref|NC_002976.3|:c6459-6313
39	79	nr3347	ref|NC_002976.3|:6522-6683
39	80	nr3348	ref|NC_002976.3|:7397-7936
39	81	nr3349	ref|NC_002976.3|:8156-8725
39	82	nr3350	ref|NC_002976.3|:c9542-8913
39	83	nr3351	ref|NC_002976.3|:9780-10898
39	84	nr3352	ref|NC_002976.3|:12806-12898
39	85	nr3353	ref|NC_002976.3|:13768-15813
39	86	nr3354	ref|NC_002976.3|:c18749-17991
40	1	nr3355	ref|NC_002976.3|:19043-19423
40	2	nr3356	ref|NC_002976.3|:c19659-19519
40	3	nr3357	ref|NC_002976.3|:20149-20727
40	4	nr3358	ref|NC_002976.3|:c22153-20789

40	5	nr3359	ref|NC_002976.3|:23557-23814	[redacted]	[redacted]
40	6	nr3360	ref|NC_002976.3|:c24756-24022	[redacted]	[redacted]
40	7	nr3361	ref|NC_002976.3|:c24956-24753	[redacted]	[redacted]
40	8	nr3362	ref|NC_002976.3|:25242-25442	[redacted]	[redacted]
40	9	nr3363	ref|NC_002976.3|:c25652-25560	[redacted]	[redacted]
40	10	nr3364	ref|NC_002976.3|:c40012-39203	[redacted]	[redacted]
40	11	nr3365	ref|NC_002976.3|:c42242-41982	[redacted]	[redacted]
40	12	nr3366	ref|NC_002976.3|:42835-42930	[redacted]	[redacted]
40	13	nr3367	ref|NC_002976.3|:43190-43822	[redacted]	[redacted]
40	14	nr3368	ref|NC_002976.3|:c45079-44849	[redacted]	[redacted]
40	15	nr3369	ref|NC_002976.3|:45180-45278	[redacted]	[redacted]
40	16	nr3370	ref|NC_002976.3|:45300-45425	[redacted]	[redacted]
40	17	nr3371	ref|NC_002976.3|:46212-46340	[redacted]	[redacted]
40	18	nr3372	ref|NC_002976.3|:c49661-48882	[redacted]	[redacted]
40	19	nr3373	ref|NC_002976.3|:c61405-60500	[redacted]	[redacted]
40	20	nr3374	ref|NC_002976.3|:c63218-62664	[redacted]	[redacted]
40	21	nr3375	ref|NC_002976.3|:c63945-63328	[redacted]	[redacted]
40	22	nr3376	ref|NC_002976.3|:c64260-64144	[redacted]	[redacted]
40	23	nr3377	ref|NC_002976.3|:c65096-64980	[redacted]	[redacted]
40	24	nr3378	ref|NC_002976.3|:66815-67198	[redacted]	[redacted]
40	25	nr3379	ref|NC_002976.3|:c69073-68810	[redacted]	[redacted]
40	26	nr3380	ref|NC_002976.3|:69325-69438	[redacted]	[redacted]
40	27	nr3381	ref|NC_002976.3|:c69548-69480	[redacted]	[redacted]
40	28	nr3382	ref|NC_002976.3|:74236-74313	[redacted]	[redacted]
40	29	nr3383	ref|NC_002976.3|:c74996-74748	[redacted]	[redacted]
40	30	nr3384	ref|NC_002976.3|:75248-75919	[redacted]	[redacted]
40	31	nr3385	ref|NC_002976.3|:76026-76754	[redacted]	[redacted]
40	32	nr3386	ref|NC_002976.3|:76769-77011	[redacted]	[redacted]
40	33	nr3387	ref|NC_002976.3|:c79202-78663	[redacted]	[redacted]
40	34	nr3388	ref|NC_002976.3|:81701-81805	[redacted]	[redacted]
40	35	nr3389	ref|NC_002976.3|:c87505-86000	[redacted]	[redacted]
40	36	nr3390	ref|NC_002976.3|:c88158-87880	[redacted]	[redacted]
40	37	nr3391	ref|NC_002976.3|:89012-89131	[redacted]	[redacted]
40	38	nr3392	ref|NC_002976.3|:c92157-91276	[redacted]	[redacted]
40	39	nr3393	ref|NC_002976.3|:105049-105141	[redacted]	[redacted]
40	40	nr3394	ref|NC_002976.3|:111135-111668	[redacted]	[redacted]
40	41	nr3395	ref|NC_002976.3|:111697-113034	[redacted]	[redacted]
40	42	nr3396	ref|NC_002976.3|:138376-138474	[redacted]	[redacted]
40	43	nr3397	ref|NC_002976.3|:164286-165299	[redacted]	[redacted]
40	44	nr3398	ref|NC_002976.3|:180424-180999	[redacted]	[redacted]
40	45	nr3399	ref|NC_002976.3|:185105-185713	[redacted]	[redacted]
40	46	nr3400	ref|NC_002976.3|:200341-200850	[redacted]	[redacted]
40	47	nr3401	ref|NC_002976.3|:201102-202052	[redacted]	[redacted]
40	48	nr3402	ref|NC_002976.3|:c202349-202140	[redacted]	[redacted]
40	49	nr3403	ref|NC_002976.3|:c211278-209974	[redacted]	[redacted]
40	50	nr3404	ref|NC_002976.3|:c212079-211327	[redacted]	[redacted]
40	51	nr3405	ref|NC_002976.3|:213774-214280	[redacted]	[redacted]
40	52	nr3406	ref|NC_002976.3|:216458-219136	[redacted]	[redacted]
40	53	nr3407	ref|NC_002976.3|:c221772-221374	[redacted]	[redacted]

40	54	nr3408	ref|NC_002976.3|:c223080-221788
40	55	nr3409	ref|NC_002976.3|:c223394-223077
40	56	nr3410	ref|NC_002976.3|:228679-230040
40	57	nr3411	ref|NC_002976.3|:231157-231519
40	58	nr3412	ref|NC_002976.3|:231523-231783
40	59	nr3413	ref|NC_002976.3|:c233864-233685
40	60	nr3414	ref|NC_002976.3|:234297-234452
40	61	nr3415	ref|NC_002976.3|:c236599-235619
40	62	nr3416	ref|NC_002976.3|:c238254-238162
40	63	nr3417	ref|NC_002976.3|:c250814-250392
40	64	nr3418	ref|NC_002976.3|:255075-256019
40	65	nr3419	ref|NC_002976.3|:256369-256938
40	66	nr3420	ref|NC_002976.3|:c257083-256979
40	67	nr3421	ref|NC_002976.3|:257149-257823
40	68	nr3422	ref|NC_002976.3|:c261975-260788
40	69	nr3423	ref|NC_002976.3|:c262748-261972
40	70	nr3424	ref|NC_002976.3|:c263590-262763
40	71	nr3425	ref|NC_002976.3|:c264884-263610
40	72	nr3426	ref|NC_002976.3|:266272-266664
40	73	nr3427	ref|NC_002976.3|:266777-266887
40	74	nr3428	ref|NC_002976.3|:267446-268459
40	75	nr3429	ref|NC_002976.3|:271030-271665
40	76	nr3430	ref|NC_002976.3|:271720-272472
40	77	nr3431	ref|NC_002976.3|:274083-275027
40	78	nr3432	ref|NC_002976.3|:276794-277303
40	79	nr3433	ref|NC_002976.3|:277340-277462
40	80	nr3434	ref|NC_002976.3|:c281164-280211
40	81	nr3435	ref|NC_002976.3|:282382-282486
40	82	nr3436	ref|NC_002976.3|:282518-282664
40	83	nr3437	ref|NC_002976.3|:c283219-283013
40	84	nr3438	ref|NC_002976.3|:289066-289368
40	85	nr3439	ref|NC_002976.3|:290114-290221
40	86	nr3440	ref|NC_002976.3|:c297214-296468
41	1	nr3441	ref|NC_002976.3|:300486-301577
41	2	nr3442	ref|NC_002976.3|:301577-302641
41	3	nr3443	ref|NC_002976.3|:303316-303426
41	4	nr3444	ref|NC_002976.3|:303515-303664
41	5	nr3445	ref|NC_002976.3|:c314433-313927
41	6	nr3446	ref|NC_002976.3|:324467-326641
41	7	nr3447	ref|NC_002976.3|:328084-328560
41	8	nr3448	ref|NC_002976.3|:330790-331008
41	9	nr3449	ref|NC_002976.3|:334098-334586
41	10	nr3450	ref|NC_002976.3|:335770-336192
41	11	nr3451	ref|NC_002976.3|:339499-339963
41	12	nr3452	ref|NC_002976.3|:c345439-345338
41	13	nr3453	ref|NC_002976.3|:346508-346801
41	14	nr3454	ref|NC_002976.3|:346794-346940
41	15	nr3455	ref|NC_002976.3|:346930-347871
41	16	nr3456	ref|NC_002976.3|:c351450-351181

41	17	nr3457	ref|NC_002976.3|:c352064-351447	[redacted]	[redacted]
41	18	nr3458	ref|NC_002976.3|:352059-352244		
41	19	nr3459	ref|NC_002976.3|:353959-354429		
41	20	nr3460	ref|NC_002976.3|:c355223-355125		
41	21	nr3461	ref|NC_002976.3|:367540-367638		
41	22	nr3462	ref|NC_002976.3|:c367885-367730		
41	23	nr3463	ref|NC_002976.3|:c368834-368235		
41	24	nr3464	ref|NC_002976.3|:373430-373636		
41	25	nr3465	ref|NC_002976.3|:c379559-378546		
41	26	nr3466	ref|NC_002976.3|:c380024-379917		
41	27	nr3467	ref|NC_002976.3|:c381138-380323		
41	28	nr3468	ref|NC_002976.3|:c395782-395687		
41	29	nr3469	ref|NC_002976.3|:401654-401920		
41	30	nr3470	ref|NC_002976.3|:417958-419121		
41	31	nr3471	ref|NC_002976.3|:c427834-427742		
41	32	nr3472	ref|NC_002976.3|:434781-435308		
41	33	nr3473	ref|NC_002976.3|:c442228-442037		
41	34	nr3474	ref|NC_002976.3|:444023-444643		
41	35	nr3475	ref|NC_002976.3|:c445052-444816		
41	36	nr3476	ref|NC_002976.3|:c458377-458147		
41	37	nr3477	ref|NC_002976.3|:458978-459166		
41	38	nr3478	ref|NC_002976.3|:c459852-459310		
41	39	nr3479	ref|NC_002976.3|:460587-460757		
41	40	nr3480	ref|NC_002976.3|:c461248-461015		
41	41	nr3481	ref|NC_002976.3|:c463289-463194		
41	42	nr3482	ref|NC_002976.3|:463394-463738		
41	43	nr3483	ref|NC_002976.3|:c464374-463958		
41	44	nr3484	ref|NC_002976.3|:c465366-465190		
41	45	nr3485	ref|NC_002976.3|:466549-466674		
41	46	nr3486	ref|NC_002976.3|:c470016-469792		
41	47	nr3487	ref|NC_002976.3|:c471169-470423		
41	48	nr3488	ref|NC_002976.3|:471450-471686		
41	49	nr3489	ref|NC_002976.3|:c472678-472490		
41	50	nr3490	ref|NC_002976.3|:473071-473208		
41	51	nr3491	ref|NC_002976.3|:473369-473572		
41	52	nr3492	ref|NC_002976.3|:474014-474601		
41	53	nr3493	ref|NC_002976.3|:c475372-474872		
41	54	nr3494	ref|NC_002976.3|:475958-476113		
41	55	nr3495	ref|NC_002976.3|:476098-476253		
41	56	nr3496	ref|NC_002976.3|:479929-480021		
41	57	nr3497	ref|NC_002976.3|:c483686-483588		
41	58	nr3498	ref|NC_002976.3|:c483949-483779		
41	59	nr3499	ref|NC_002976.3|:491410-491556		
41	60	nr3500	ref|NC_002976.3|:491547-491771		
41	61	nr3501	ref|NC_002976.3|:492239-492445		
41	62	nr3502	ref|NC_002976.3|:492542-492901		
41	63	nr3503	ref|NC_002976.3|:492915-493325		
41	64	nr3504	ref|NC_002976.3|:c493507-493340		
41	65	nr3505	ref|NC_002976.3|:501635-502606		

41	66	nr3506	ref|NC_002976.3|:509829-510074	[redacted]	[redacted]
41	67	nr3507	ref|NC_002976.3|:c518266-517787	[redacted]	[redacted]
41	68	nr3508	ref|NC_002976.3|:c528343-527330	[redacted]	[redacted]
41	69	nr3509	ref|NC_002976.3|:537549-538124	[redacted]	[redacted]
41	70	nr3510	ref|NC_002976.3|:538128-538649	[redacted]	[redacted]
41	71	nr3511	ref|NC_002976.3|:548857-549870	[redacted]	[redacted]
41	72	nr3512	ref|NC_002976.3|:550250-550405	[redacted]	[redacted]
41	73	nr3513	ref|NC_002976.3|:558754-559767	[redacted]	[redacted]
41	74	nr3514	ref|NC_002976.3|:c560220-560026	[redacted]	[redacted]
41	75	nr3515	ref|NC_002976.3|:c563141-562770	[redacted]	[redacted]
41	76	nr3516	ref|NC_002976.3|:c572110-571097	[redacted]	[redacted]
41	77	nr3517	ref|NC_002976.3|:573537-574517	[redacted]	[redacted]
41	78	nr3518	ref|NC_002976.3|:c578696-578106	[redacted]	[redacted]
41	79	nr3519	ref|NC_002976.3|:c578848-578711	[redacted]	[redacted]
41	80	nr3520	ref|NC_002976.3|:580800-581654	[redacted]	[redacted]
41	81	nr3521	ref|NC_002976.3|:585628-586251	[redacted]	[redacted]
41	82	nr3522	ref|NC_002976.3|:588630-590888	[redacted]	[redacted]
41	83	nr3523	ref|NC_002976.3|:590900-591028	[redacted]	[redacted]
41	84	nr3524	ref|NC_002976.3|:592507-592878	[redacted]	[redacted]
41	85	nr3525	ref|NC_002976.3|:601599-601844	[redacted]	[redacted]
41	86	nr3526	ref|NC_002976.3|:c611896-611678	[redacted]	[redacted]
42	1	nr3527	ref|NC_002976.3|:c616124-615966	[redacted]	[redacted]
42	2	nr3528	ref|NC_002976.3|:616295-616402	[redacted]	[redacted]
42	3	nr3529	ref|NC_002976.3|:617006-617098	[redacted]	[redacted]
42	4	nr3530	ref|NC_002976.3|:617221-617778	[redacted]	[redacted]
42	5	nr3531	ref|NC_002976.3|:619362-619457	[redacted]	[redacted]
42	6	nr3532	ref|NC_002976.3|:c632326-631904	[redacted]	[redacted]
42	7	nr3533	ref|NC_002976.3|:c632552-632421	[redacted]	[redacted]
42	8	nr3534	ref|NC_002976.3|:c637730-636822	[redacted]	[redacted]
42	9	nr3535	ref|NC_002976.3|:660702-660794	[redacted]	[redacted]
42	10	nr3536	ref|NC_002976.3|:c661204-661070	[redacted]	[redacted]
42	11	nr3537	ref|NC_002976.3|:c667379-667143	[redacted]	[redacted]
42	12	nr3538	ref|NC_002976.3|:674711-675337	[redacted]	[redacted]
42	13	nr3539	ref|NC_002976.3|:687017-687553	[redacted]	[redacted]
42	14	nr3540	ref|NC_002976.3|:693996-695009	[redacted]	[redacted]
42	15	nr3541	ref|NC_002976.3|:c697516-697382	[redacted]	[redacted]
42	16	nr3542	ref|NC_002976.3|:708783-708902	[redacted]	[redacted]
42	17	nr3543	ref|NC_002976.3|:c716143-713669	[redacted]	[redacted]
42	18	nr3544	ref|NC_002976.3|:722554-723075	[redacted]	[redacted]
42	19	nr3545	ref|NC_002976.3|:736115-736279	[redacted]	[redacted]
42	20	nr3546	ref|NC_002976.3|:736335-736469	[redacted]	[redacted]
42	21	nr3547	ref|NC_002976.3|:736521-736655	[redacted]	[redacted]
42	22	nr3548	ref|NC_002976.3|:736711-736845	[redacted]	[redacted]
42	23	nr3549	ref|NC_002976.3|:753923-754585	[redacted]	[redacted]
42	24	nr3550	ref|NC_002976.3|:758955-759050	[redacted]	[redacted]
42	25	nr3551	ref|NC_002976.3|:759899-760369	[redacted]	[redacted]
42	26	nr3552	ref|NC_002976.3|:772066-772272	[redacted]	[redacted]
42	27	nr3553	ref|NC_002976.3|:c772682-772548	[redacted]	[redacted]
42	28	nr3554	ref|NC_002976.3|:780762-781250	[redacted]	[redacted]

42	29	nr3555	ref|NC_002976.3|:c813759-811159	[redacted]	[redacted]
42	30	nr3556	ref|NC_002976.3|:813903-814067		
42	31	nr3557	ref|NC_002976.3|:827327-827422		
42	32	nr3558	ref|NC_002976.3|:870209-870826		
42	33	nr3559	ref|NC_002976.3|:873062-873427		
42	34	nr3560	ref|NC_002976.3|:884189-885115		
42	35	nr3561	ref|NC_002976.3|:892406-892645		
42	36	nr3562	ref|NC_002976.3|:c895935-895252		
42	37	nr3563	ref|NC_002976.3|:897344-897541		
42	38	nr3564	ref|NC_002976.3|:900852-901946		
42	39	nr3565	ref|NC_002976.3|:c902902-902714		
42	40	nr3566	ref|NC_002976.3|:c905540-905415		
42	41	nr3567	ref|NC_002976.3|:911116-911922		
42	42	nr3568	ref|NC_002976.3|:918967-919068		
42	43	nr3569	ref|NC_002976.3|:920030-920263		
42	44	nr3570	ref|NC_002976.3|:922929-923051		
42	45	nr3571	ref|NC_002976.3|:923527-924699		
42	46	nr3572	ref|NC_002976.3|:957833-958456		
42	47	nr3573	ref|NC_002976.3|:961418-961540		
42	48	nr3574	ref|NC_002976.3|:965895-966905		
42	49	nr3575	ref|NC_002976.3|:990377-990589		
42	50	nr3576	ref|NC_002976.3|:991427-991696		
42	51	nr3577	ref|NC_002976.3|:999229-999402		
42	52	nr3578	ref|NC_002976.3|:c1000235-999534		
42	53	nr3579	ref|NC_002976.3|:c1001062-1000232		
42	54	nr3580	ref|NC_002976.3|:1005308-1005418		
42	55	nr3581	ref|NC_002976.3|:c1009396-1008383		
42	56	nr3582	ref|NC_002976.3|:1012226-1012342		
42	57	nr3583	ref|NC_002976.3|:c1016983-1016135		
42	58	nr3584	ref|NC_002976.3|:c1017478-1016993		
42	59	nr3585	ref|NC_002976.3|:c1018476-1017520		
42	60	nr3586	ref|NC_002976.3|:c1019452-1018790		
42	61	nr3587	ref|NC_002976.3|:c1021684-1021490		
42	62	nr3588	ref|NC_002976.3|:c1059673-1059035		
42	63	nr3589	ref|NC_002976.3|:c1062882-1062550		
42	64	nr3590	ref|NC_002976.3|:c1077326-1076733		
42	65	nr3591	ref|NC_002976.3|:c1086059-1085481		
42	66	nr3592	ref|NC_002976.3|:c1098624-1098520		
42	67	nr3593	ref|NC_002976.3|:1113125-1113220		
42	68	nr3594	ref|NC_002976.3|:1119107-1119202		
42	69	nr3595	ref|NC_002976.3|:c1134830-1133703		
42	70	nr3596	ref|NC_002976.3|:c1135570-1134833		
42	71	nr3597	ref|NC_002976.3|:c1137816-1136791		
42	72	nr3598	ref|NC_002976.3|:1140306-1140524		
42	73	nr3599	ref|NC_002976.3|:c1148177-1147686		
42	74	nr3600	ref|NC_002976.3|:c1148394-1148101		
42	75	nr3601	ref|NC_002976.3|:c1148830-1148381		
42	76	nr3602	ref|NC_002976.3|:c1150210-1149143		
42	77	nr3603	ref|NC_002976.3|:c1155344-1154793		

42	78	nr3604	ref|NC_002976.3|:1157913-1158014
42	79	nr3605	ref|NC_002976.3|:1170668-1170766
42	80	nr3606	ref|NC_002976.3|:c1174898-1174542
42	81	nr3607	ref|NC_002976.3|:c1179638-1179528
42	82	nr3608	ref|NC_002976.3|:c1195062-1192846
42	83	nr3609	ref|NC_002976.3|:c1198562-1197987
42	84	nr3610	ref|NC_002976.3|:1235389-1235490
42	85	nr3611	ref|NC_002976.3|:c1249317-1248796
42	86	nr3612	ref|NC_002976.3|:c1252717-1252055
43	1	nr3613	ref|NC_002976.3|:1253109-1253168
43	2	nr3614	ref|NC_002976.3|:1253251-1253982
43	3	nr3615	ref|NC_002976.3|:c1254890-1254108
43	4	nr3616	ref|NC_002976.3|:c1255418-1255041
43	5	nr3617	ref|NC_002976.3|:c1257317-1255425
43	6	nr3618	ref|NC_002976.3|:c1258399-1257314
43	7	nr3619	ref|NC_002976.3|:c1259779-1259069
43	8	nr3620	ref|NC_002976.3|:c1299928-1298756
43	9	nr3621	ref|NC_002976.3|:c1325507-1324368
43	10	nr3622	ref|NC_002976.3|:c1328325-1328209
43	11	nr3623	ref|NC_002976.3|:c1329975-1329229
43	12	nr3624	ref|NC_002976.3|:1330053-1330493
43	13	nr3625	ref|NC_002976.3|:c1347427-1346204
43	14	nr3626	ref|NC_002976.3|:1356033-1356347
43	15	nr3627	ref|NC_002976.3|:c1363901-1363803
43	16	nr3628	ref|NC_002976.3|:1382418-1382981
43	17	nr3629	ref|NC_002976.3|:1393531-1393650
43	18	nr3630	ref|NC_002976.3|:1393706-1394185
43	19	nr3631	ref|NC_002976.3|:1394191-1394634
43	20	nr3632	ref|NC_002976.3|:c1395064-1394621
43	21	nr3633	ref|NC_002976.3|:1396901-1397254
43	22	nr3634	ref|NC_002976.3|:1397898-1398374
43	23	nr3635	ref|NC_002976.3|:c1399155-1398493
43	24	nr3636	ref|NC_002976.3|:1399547-1399606
43	25	nr3637	ref|NC_002976.3|:1399689-1400420
43	26	nr3638	ref|NC_002976.3|:c1401328-1400546
43	27	nr3639	ref|NC_002976.3|:c1401856-1401479
43	28	nr3640	ref|NC_002976.3|:c1403755-1401863
43	29	nr3641	ref|NC_002976.3|:c1404837-1403752
43	30	nr3642	ref|NC_002976.3|:1405027-1405623
43	31	nr3643	ref|NC_002976.3|:c1412960-1412484
43	32	nr3644	ref|NC_002976.3|:c1414268-1413267
43	33	nr3645	ref|NC_002976.3|:1416692-1416838
43	34	nr3646	ref|NC_002976.3|:1416933-1417598
43	35	nr3647	ref|NC_002976.3|:1417760-1418047
43	36	nr3648	ref|NC_002976.3|:1418574-1419308
43	37	nr3649	ref|NC_002976.3|:c1428085-1427468
43	38	nr3650	ref|NC_002976.3|:c1433263-1433159
43	39	nr3651	ref|NC_002976.3|:c1449378-1449223
43	40	nr3652	ref|NC_002976.3|:1469571-1469681

43	41	nr3653	ref|NC_002976.3|:c1473655-1472825	[redacted]	[redacted]
43	42	nr3654	ref|NC_002976.3|:c1482200-1481985	[redacted]	[redacted]
43	43	nr3655	ref|NC_002976.3|:c1487512-1487117	[redacted]	[redacted]
43	44	nr3656	ref|NC_002976.3|:1488590-1489576	[redacted]	[redacted]
43	45	nr3657	ref|NC_002976.3|:c1490263-1489727	[redacted]	[redacted]
43	46	nr3658	ref|NC_002976.3|:c1496162-1495620	[redacted]	[redacted]
43	47	nr3659	ref|NC_002976.3|:c1513117-1512938	[redacted]	[redacted]
43	48	nr3660	ref|NC_002976.3|:1524506-1524643	[redacted]	[redacted]
43	49	nr3661	ref|NC_002976.3|:c1529911-1528541	[redacted]	[redacted]
43	50	nr3662	ref|NC_002976.3|:c1530281-1529916	[redacted]	[redacted]
43	51	nr3663	ref|NC_002976.3|:c1532317-1530290	[redacted]	[redacted]
43	52	nr3664	ref|NC_002976.3|:c1533468-1532356	[redacted]	[redacted]
43	53	nr3665	ref|NC_002976.3|:c1536648-1535812	[redacted]	[redacted]
43	54	nr3666	ref|NC_002976.3|:c1537800-1537255	[redacted]	[redacted]
43	55	nr3667	ref|NC_002976.3|:c1539261-1538530	[redacted]	[redacted]
43	56	nr3668	ref|NC_002976.3|:c1540813-1540154	[redacted]	[redacted]
43	57	nr3669	ref|NC_002976.3|:c1542854-1542759	[redacted]	[redacted]
43	58	nr3670	ref|NC_002976.3|:c1550422-1544501	[redacted]	[redacted]
43	59	nr3671	ref|NC_002976.3|:c1551672-1550599	[redacted]	[redacted]
43	60	nr3672	ref|NC_002976.3|:1558381-1558965	[redacted]	[redacted]
43	61	nr3673	ref|NC_002976.3|:1558949-1559089	[redacted]	[redacted]
43	62	nr3674	ref|NC_002976.3|:c1563902-1563810	[redacted]	[redacted]
43	63	nr3675	ref|NC_002976.3|:c1569306-1567768	[redacted]	[redacted]
43	64	nr3676	ref|NC_002976.3|:c1569877-1569296	[redacted]	[redacted]
43	65	nr3677	ref|NC_002976.3|:c1570100-1569969	[redacted]	[redacted]
43	66	nr3678	ref|NC_002976.3|:c1570684-1570079	[redacted]	[redacted]
43	67	nr3679	ref|NC_002976.3|:c1571051-1570656	[redacted]	[redacted]
43	68	nr3680	ref|NC_002976.3|:c1571799-1571053	[redacted]	[redacted]
43	69	nr3681	ref|NC_002976.3|:c1572051-1571812	[redacted]	[redacted]
43	70	nr3682	ref|NC_002976.3|:c1572315-1572067	[redacted]	[redacted]
43	71	nr3683	ref|NC_002976.3|:c1572958-1572356	[redacted]	[redacted]
43	72	nr3684	ref|NC_002976.3|:c1574008-1572986	[redacted]	[redacted]
43	73	nr3685	ref|NC_002976.3|:c1574317-1574054	[redacted]	[redacted]
43	74	nr3686	ref|NC_002976.3|:c1575425-1574601	[redacted]	[redacted]
43	75	nr3687	ref|NC_002976.3|:c1578488-1575579	[redacted]	[redacted]
43	76	nr3688	ref|NC_002976.3|:c1578956-1578510	[redacted]	[redacted]
43	77	nr3689	ref|NC_002976.3|:c1579369-1578956	[redacted]	[redacted]
43	78	nr3690	ref|NC_002976.3|:c1579551-1579399	[redacted]	[redacted]
43	79	nr3691	ref|NC_002976.3|:c1580271-1579570	[redacted]	[redacted]
43	80	nr3692	ref|NC_002976.3|:c1580783-1580283	[redacted]	[redacted]
43	81	nr3693	ref|NC_002976.3|:c1581514-1580918	[redacted]	[redacted]
43	82	nr3694	ref|NC_002976.3|:c1581744-1581514	[redacted]	[redacted]
43	83	nr3695	ref|NC_002976.3|:c1581940-1581758	[redacted]	[redacted]
43	84	nr3696	ref|NC_002976.3|:c1582254-1581943	[redacted]	[redacted]
43	85	nr3697	ref|NC_002976.3|:c1583336-1582329	[redacted]	[redacted]
43	86	nr3698	ref|NC_002976.3|:c1584028-1583342	[redacted]	[redacted]
44	1	nr3699	ref|NC_002976.3|:c1584209-1584018	[redacted]	[redacted]
44	2	nr3700	ref|NC_002976.3|:c1584813-1584202	[redacted]	[redacted]
44	3	nr3701	ref|NC_002976.3|:c1585010-1584828	[redacted]	[redacted]

44	4	nr3702	ref|NC_002976.3|:c1585261-1585010	[redacted]	[redacted]
44	5	nr3703	ref|NC_002976.3|:c1585591-1585418	[redacted]	[redacted]
44	6	nr3704	ref|NC_002976.3|:c1585859-1585572	[redacted]	[redacted]
44	7	nr3705	ref|NC_002976.3|:c1586118-1585852	[redacted]	[redacted]
44	8	nr3706	ref|NC_002976.3|:c1586303-1586130	[redacted]	[redacted]
44	9	nr3707	ref|NC_002976.3|:c1586806-1586417	[redacted]	[redacted]
44	10	nr3708	ref|NC_002976.3|:c1588192-1586894	[redacted]	[redacted]
44	11	nr3709	ref|NC_002976.3|:c1588516-1588202	[redacted]	[redacted]
44	12	nr3710	ref|NC_002976.3|:c1589034-1588654	[redacted]	[redacted]
44	13	nr3711	ref|NC_002976.3|:c1591632-1589146	[redacted]	[redacted]
44	14	nr3712	ref|NC_002976.3|:c1593543-1592095	[redacted]	[redacted]
44	15	nr3713	ref|NC_002976.3|:c1593930-1593556	[redacted]	[redacted]
44	16	nr3714	ref|NC_002976.3|:1594097-1594657	[redacted]	[redacted]
44	17	nr3715	ref|NC_002976.3|:1595010-1595414	[redacted]	[redacted]
44	18	nr3716	ref|NC_002976.3|:1595497-1595631	[redacted]	[redacted]
44	19	nr3717	ref|NC_002976.3|:c1596303-1595662	[redacted]	[redacted]
44	20	nr3718	ref|NC_002976.3|:c1597131-1596409	[redacted]	[redacted]
44	21	nr3719	ref|NC_002976.3|:c1598040-1597240	[redacted]	[redacted]
44	22	nr3720	ref|NC_002976.3|:c1598538-1598044	[redacted]	[redacted]
44	23	nr3721	ref|NC_002976.3|:c1598864-1598595	[redacted]	[redacted]
44	24	nr3722	ref|NC_002976.3|:c1599063-1598848	[redacted]	[redacted]
44	25	nr3723	ref|NC_002976.3|:c1599737-1599342	[redacted]	[redacted]
44	26	nr3724	ref|NC_002976.3|:c1599904-1599737	[redacted]	[redacted]
44	27	nr3725	ref|NC_002976.3|:c1600037-1599933	[redacted]	[redacted]
44	28	nr3726	ref|NC_002976.3|:c1600648-1600130	[redacted]	[redacted]
44	29	nr3727	ref|NC_002976.3|:c1601298-1600648	[redacted]	[redacted]
44	30	nr3728	ref|NC_002976.3|:c1601618-1601298	[redacted]	[redacted]
44	31	nr3729	ref|NC_002976.3|:c1602333-1601608	[redacted]	[redacted]
44	32	nr3730	ref|NC_002976.3|:c1602554-1602333	[redacted]	[redacted]
44	33	nr3731	ref|NC_002976.3|:c1602750-1602574	[redacted]	[redacted]
44	34	nr3732	ref|NC_002976.3|:c1603285-1602743	[redacted]	[redacted]
44	35	nr3733	ref|NC_002976.3|:c1603573-1603301	[redacted]	[redacted]
44	36	nr3734	ref|NC_002976.3|:c1603754-1603599	[redacted]	[redacted]
44	37	nr3735	ref|NC_002976.3|:c1604035-1603751	[redacted]	[redacted]
44	38	nr3736	ref|NC_002976.3|:c1604236-1604051	[redacted]	[redacted]
44	39	nr3737	ref|NC_002976.3|:c1604400-1604221	[redacted]	[redacted]
44	40	nr3738	ref|NC_002976.3|:c1604898-1604416	[redacted]	[redacted]
44	41	nr3739	ref|NC_002976.3|:c1605298-1605095	[redacted]	[redacted]
44	42	nr3740	ref|NC_002976.3|:c1605569-1605303	[redacted]	[redacted]
44	43	nr3741	ref|NC_002976.3|:c1605866-1605591	[redacted]	[redacted]
44	44	nr3742	ref|NC_002976.3|:c1606128-1605889	[redacted]	[redacted]
44	45	nr3743	ref|NC_002976.3|:c1606374-1606150	[redacted]	[redacted]
44	46	nr3744	ref|NC_002976.3|:c1606727-1606380	[redacted]	[redacted]
44	47	nr3745	ref|NC_002976.3|:c1607134-1606724	[redacted]	[redacted]
44	48	nr3746	ref|NC_002976.3|:c1607437-1607174	[redacted]	[redacted]
44	49	nr3747	ref|NC_002976.3|:c1607748-1607440	[redacted]	[redacted]
44	50	nr3748	ref|NC_002976.3|:c1607864-1607754	[redacted]	[redacted]
44	51	nr3749	ref|NC_002976.3|:c1607967-1607845	[redacted]	[redacted]
44	52	nr3750	ref|NC_002976.3|:c1608315-1608040	[redacted]	[redacted]

44	53	nr3751	ref|NC_002976.3|:c1608525-1608328
44	54	nr3752	ref|NC_002976.3|:c1609375-1608536
44	55	nr3753	ref|NC_002976.3|:1609626-1610300
44	56	nr3754	ref|NC_002976.3|:1612361-1612684
44	57	nr3755	ref|NC_002976.3|:1612762-1613934
44	58	nr3756	ref|NC_002976.3|:c1617125-1615953
44	59	nr3757	ref|NC_002976.3|:1617188-1617610
44	60	nr3758	ref|NC_002976.3|:c1618646-1617909
44	61	nr3759	ref|NC_002976.3|:c1620605-1618839
44	62	nr3760	ref|NC_002976.3|:c1621720-1620608
44	63	nr3761	ref|NC_002976.3|:c1623269-1621737
44	64	nr3762	ref|NC_002976.3|:c1623809-1623291
44	65	nr3763	ref|NC_002976.3|:c1624854-1623886
44	66	nr3764	ref|NC_002976.3|:c1625937-1624978
44	67	nr3765	ref|NC_002976.3|:c1626661-1625981
44	68	nr3766	ref|NC_002976.3|:c1627167-1626679
44	69	nr3767	ref|NC_002976.3|:c1627476-1627183
44	70	nr3768	ref|NC_002976.3|:c1628595-1627591
44	71	nr3769	ref|NC_002976.3|:c1629887-1628607
44	72	nr3770	ref|NC_002976.3|:c1631241-1630198
44	73	nr3771	ref|NC_002976.3|:c1631941-1631834
44	74	nr3772	ref|NC_002976.3|:c1632353-1631925
44	75	nr3773	ref|NC_002976.3|:c1632952-1632431
44	76	nr3774	ref|NC_002976.3|:c1633404-1632952
44	77	nr3775	ref|NC_002976.3|:c1634200-1633520
44	78	nr3776	ref|NC_002976.3|:c1634492-1634229
44	79	nr3777	ref|NC_002976.3|:c1634908-1634585
44	80	nr3778	ref|NC_002976.3|:c1635612-1634953
44	81	nr3779	ref|NC_002976.3|:c1636154-1635765
44	82	nr3780	ref|NC_002976.3|:c1636551-1636291
44	83	nr3781	ref|NC_002976.3|:c1636841-1636626
44	84	nr3782	ref|NC_002976.3|:c1637006-1636893
44	85	nr3783	ref|NC_002976.3|:c1637305-1637021
44	86	nr3784	ref|NC_002976.3|:c1638819-1638658
45	1	nr3785	ref|NC_002976.3|:c1639341-1639084
45	2	nr3786	ref|NC_002976.3|:1640612-1640752
45	3	nr3787	ref|NC_002976.3|:c1642075-1641200
45	4	nr3788	ref|NC_002976.3|:c1644396-1642111
45	5	nr3789	ref|NC_002976.3|:1644532-1646028
45	6	nr3790	ref|NC_002976.3|:1646288-1646617
45	7	nr3791	ref|NC_002976.3|:1646643-1646828
45	8	nr3792	ref|NC_002976.3|:1646837-1648021
45	9	nr3793	ref|NC_002976.3|:1649716-1650600
45	10	nr3794	ref|NC_002976.3|:1650593-1652392
45	11	nr3795	ref|NC_002976.3|:1652410-1653972
45	12	nr3796	ref|NC_002976.3|:1653965-1655344
45	13	nr3797	ref|NC_002976.3|:1655372-1655812
45	14	nr3798	ref|NC_002976.3|:1655851-1656852
45	15	nr3799	ref|NC_002976.3|:1656927-1657478

45	16	nr3800	ref|NC_002976.3|:1657496-1657930	[redacted]	[redacted]
45	17	nr3801	ref|NC_002976.3|:1657920-1658237		
45	18	nr3802	ref|NC_002976.3|:1658241-1658906		
45	19	nr3803	ref|NC_002976.3|:1658896-1659417		
45	20	nr3804	ref|NC_002976.3|:1659423-1660295		
45	21	nr3805	ref|NC_002976.3|:1660334-1661125		
45	22	nr3806	ref|NC_002976.3|:1661209-1661766		
45	23	nr3807	ref|NC_002976.3|:1661766-1662239		
45	24	nr3808	ref|NC_002976.3|:1662255-1663271		
45	25	nr3809	ref|NC_002976.3|:1663368-1671641		
45	26	nr3810	ref|NC_002976.3|:1671663-1673744		
45	27	nr3811	ref|NC_002976.3|:1673805-1681850		
45	28	nr3812	ref|NC_002976.3|:1681853-1682227		
45	29	nr3813	ref|NC_002976.3|:1682220-1682387		
45	30	nr3814	ref|NC_002976.3|:1682429-1685098		
45	31	nr3815	ref|NC_002976.3|:1685145-1685606		
45	32	nr3816	ref|NC_002976.3|:1685588-1686100		
45	33	nr3817	ref|NC_002976.3|:1686122-1686451		
45	34	nr3818	ref|NC_002976.3|:1686503-1688896		
45	35	nr3819	ref|NC_002976.3|:1689084-1690475		
45	36	nr3820	ref|NC_002976.3|:c1690751-1690551		
45	37	nr3821	ref|NC_002976.3|:c1691307-1691131		
45	38	nr3822	ref|NC_002976.3|:c1693092-1691446		
45	39	nr3823	ref|NC_002976.3|:c1693840-1693235		
45	40	nr3824	ref|NC_002976.3|:c1694824-1694543		
45	41	nr3825	ref|NC_002976.3|:c1698167-1698060		
45	42	nr3826	ref|NC_002976.3|:1706411-1706644		
45	43	nr3827	ref|NC_002976.3|:c1713810-1713700		
45	44	nr3828	ref|NC_002976.3|:c1720374-1719919		
45	45	nr3829	ref|NC_002976.3|:c1727987-1727634		
45	46	nr3830	ref|NC_002976.3|:c1728544-1728035		
45	47	nr3831	ref|NC_002976.3|:c1730036-1728531		
45	48	nr3832	ref|NC_002976.3|:c1730508-1730029		
45	49	nr3833	ref|NC_002976.3|:c1737217-1737008		
45	50	nr3834	ref|NC_002976.3|:c1741718-1741077		
45	51	nr3835	ref|NC_002976.3|:c1760060-1759641		
45	52	nr3836	ref|NC_002976.3|:c1761228-1761076		
45	53	nr3837	ref|NC_002976.3|:c1762099-1761263		
45	54	nr3838	ref|NC_002976.3|:c1768792-1767779		
45	55	nr3839	ref|NC_002976.3|:c1782089-1781739		
45	56	nr3840	ref|NC_002976.3|:c1785397-1785278		
45	57	nr3841	ref|NC_002976.3|:c1789387-1789160		
45	58	nr3842	ref|NC_002976.3|:c1790824-1790675		
45	59	nr3843	ref|NC_002976.3|:c1794096-1793365		
45	60	nr3844	ref|NC_002976.3|:1794229-1795380		
45	61	nr3845	ref|NC_002976.3|:c1799038-1798883		
45	62	nr3846	ref|NC_002976.3|:c1812037-1811567		
45	63	nr3847	ref|NC_002976.3|:1816844-1817158		
45	64	nr3848	ref|NC_002976.3|:c1829136-1828897		

45	65	nr3849	ref|NC_002976.3|:c1862665-1862552	[redacted]	[redacted]
45	66	nr3850	ref|NC_002976.3|:c1867131-1866982	[redacted]	[redacted]
45	67	nr3851	ref|NC_002976.3|:1867995-1868786	[redacted]	[redacted]
45	68	nr3852	ref|NC_002976.3|:1870031-1870201	[redacted]	[redacted]
45	69	nr3853	ref|NC_002976.3|:c1876504-1875743	[redacted]	[redacted]
45	70	nr3854	ref|NC_002976.3|:1876667-1877107	[redacted]	[redacted]
45	71	nr3855	ref|NC_002976.3|:c1881772-1881539	[redacted]	[redacted]
45	72	nr3856	ref|NC_002976.3|:c1882698-1882222	[redacted]	[redacted]
45	73	nr3857	ref|NC_002976.3|:c1886813-1886193	[redacted]	[redacted]
45	74	nr3858	ref|NC_002976.3|:c1890160-1889405	[redacted]	[redacted]
45	75	nr3859	ref|NC_002976.3|:1893274-1893408	[redacted]	[redacted]
45	76	nr3860	ref|NC_002976.3|:c1894592-1893690	[redacted]	[redacted]
45	77	nr3861	ref|NC_002976.3|:1899070-1899906	[redacted]	[redacted]
45	78	nr3862	ref|NC_002976.3|:c1901847-1901119	[redacted]	[redacted]
45	79	nr3863	ref|NC_002976.3|:1908567-1909061	[redacted]	[redacted]
45	80	nr3864	ref|NC_002976.3|:c1910014-1909922	[redacted]	[redacted]
45	81	nr3865	ref|NC_002976.3|:1910009-1910335	[redacted]	[redacted]
45	82	nr3866	ref|NC_002976.3|:1910341-1910658	[redacted]	[redacted]
45	83	nr3867	ref|NC_002976.3|:1913066-1913233	[redacted]	[redacted]
45	84	nr3868	ref|NC_002976.3|:c1914410-1914135	[redacted]	[redacted]
45	85	nr3869	ref|NC_002976.3|:1919092-1919427	[redacted]	[redacted]
45	86	nr3870	ref|NC_002976.3|:1919607-1920659	[redacted]	[redacted]
46	1	nr3871	ref|NC_002976.3|:c1925578-1925480	[redacted]	[redacted]
46	2	nr3872	ref|NC_002976.3|:1925764-1926120	[redacted]	[redacted]
46	3	nr3873	ref|NC_002976.3|:c1926807-1926619	[redacted]	[redacted]
46	4	nr3874	ref|NC_002976.3|:1931485-1932498	[redacted]	[redacted]
46	5	nr3875	ref|NC_002976.3|:c1936164-1935577	[redacted]	[redacted]
46	6	nr3876	ref|NC_002976.3|:c1936819-1936208	[redacted]	[redacted]
46	7	nr3877	ref|NC_002976.3|:1942195-1942302	[redacted]	[redacted]
46	8	nr3878	ref|NC_002976.3|:c1942587-1942408	[redacted]	[redacted]
46	9	nr3879	ref|NC_002976.3|:1942565-1942750	[redacted]	[redacted]
46	10	nr3880	ref|NC_002976.3|:1946198-1947808	[redacted]	[redacted]
46	11	nr3881	ref|NC_002976.3|:c1948156-1948055	[redacted]	[redacted]
46	12	nr3882	ref|NC_002976.3|:1948267-1948377	[redacted]	[redacted]
46	13	nr3883	ref|NC_002976.3|:c1950725-1949922	[redacted]	[redacted]
46	14	nr3884	ref|NC_002976.3|:c1951282-1950722	[redacted]	[redacted]
46	15	nr3885	ref|NC_002976.3|:c1951599-1951279	[redacted]	[redacted]
46	16	nr3886	ref|NC_002976.3|:1955901-1956290	[redacted]	[redacted]
46	17	nr3887	ref|NC_002976.3|:1961133-1961684	[redacted]	[redacted]
46	18	nr3888	ref|NC_002976.3|:c1964378-1962990	[redacted]	[redacted]
46	19	nr3889	ref|NC_002976.3|:c1965113-1964658	[redacted]	[redacted]
46	20	nr3890	ref|NC_002976.3|:1965301-1966257	[redacted]	[redacted]
46	21	nr3891	ref|NC_002976.3|:1972366-1973511	[redacted]	[redacted]
46	22	nr3892	ref|NC_002976.3|:1975667-1977346	[redacted]	[redacted]
46	23	nr3893	ref|NC_002976.3|:c1978125-1977478	[redacted]	[redacted]
46	24	nr3894	ref|NC_002976.3|:c1981230-1980691	[redacted]	[redacted]
46	25	nr3895	ref|NC_002976.3|:1989454-1989549	[redacted]	[redacted]
46	26	nr3896	ref|NC_002976.3|:1989550-1989660	[redacted]	[redacted]
46	27	nr3897	ref|NC_002976.3|:1993044-1993394	[redacted]	[redacted]

46	28	nr3898	ref|NC_002976.3|:c1993601-1993479	[redacted]
46	29	nr3899	ref|NC_002976.3|:c1995487-1995122	[redacted]
46	30	nr3900	ref|NC_002976.3|:c1996240-1995611	[redacted]
46	31	nr3901	ref|NC_002976.3|:c2009060-2008746	[redacted]
46	32	nr3902	ref|NC_002976.3|:c2011848-2011744	[redacted]
46	33	nr3903	ref|NC_002976.3|:c2012625-2011906	[redacted]
46	34	nr3904	ref|NC_002976.3|:c2012731-2012615	[redacted]
46	35	nr3905	ref|NC_002976.3|:2014631-2014786	[redacted]
46	36	nr3906	ref|NC_002976.3|:c2017591-2017121	[redacted]
46	37	nr3907	ref|NC_002976.3|:c2018469-2017873	[redacted]
46	38	nr3908	ref|NC_002976.3|:c2018855-2018493	[redacted]
46	39	nr3909	ref|NC_002976.3|:2020849-2020995	[redacted]
46	40	nr3910	ref|NC_002976.3|:2023570-2023674	[redacted]
46	41	nr3911	ref|NC_002976.3|:c2027839-2026367	[redacted]
46	42	nr3912	ref|NC_002976.3|:c2040196-2040104	[redacted]
46	43	nr3913	ref|NC_002976.3|:2040235-2041071	[redacted]
46	44	nr3914	ref|NC_002976.3|:2044375-2046150	[redacted]
46	45	nr3915	ref|NC_002976.3|:2046131-2046889	[redacted]
46	46	nr3916	ref|NC_002976.3|:c2050008-2049064	[redacted]
46	47	nr3917	ref|NC_002976.3|:2052409-2053461	[redacted]
46	48	nr3918	ref|NC_002976.3|:2056056-2057411	[redacted]
46	49	nr3919	ref|NC_002976.3|:c2060720-2060538	[redacted]
46	50	nr3920	ref|NC_002976.3|:c2061631-2060849	[redacted]
46	51	nr3921	ref|NC_002976.3|:c2063873-2063406	[redacted]
46	52	nr3922	ref|NC_002976.3|:2065150-2065338	[redacted]
46	53	nr3923	ref|NC_002976.3|:2066632-2068164	[redacted]
46	54	nr3924	ref|NC_002976.3|:2068227-2070392	[redacted]
46	55	nr3925	ref|NC_002976.3|:c2071815-2070559	[redacted]
46	56	nr3926	ref|NC_002976.3|:2072959-2073105	[redacted]
46	57	nr3927	ref|NC_002976.3|:c2073579-2073388	[redacted]
46	58	nr3928	ref|NC_002976.3|:c2078746-2077256	[redacted]
46	59	nr3929	ref|NC_002976.3|:c2088187-2087471	[redacted]
46	60	nr3930	ref|NC_002976.3|:c2088304-2088206	[redacted]
46	61	nr3931	ref|NC_002976.3|:c2089148-2088273	[redacted]
46	62	nr3932	ref|NC_002976.3|:2089454-2090371	[redacted]
46	63	nr3933	ref|NC_002976.3|:c2091869-2091456	[redacted]
46	64	nr3934	ref|NC_002976.3|:c2094315-2094025	[redacted]
46	65	nr3935	ref|NC_002976.3|:2096555-2097013	[redacted]
46	66	nr3936	ref|NC_002976.3|:2097006-2098124	[redacted]
46	67	nr3937	ref|NC_002976.3|:2098108-2098308	[redacted]
46	68	nr3938	ref|NC_002976.3|:2098310-2099077	[redacted]
46	69	nr3939	ref|NC_002976.3|:2099079-2100077	[redacted]
46	70	nr3940	ref|NC_002976.3|:c2104947-2103160	[redacted]
46	71	nr3941	ref|NC_002976.3|:2105085-2105213	[redacted]
46	72	nr3942	ref|NC_002976.3|:2106578-2108017	[redacted]
46	73	nr3943	ref|NC_002976.3|:c2109035-2108088	[redacted]
46	74	nr3944	ref|NC_002976.3|:c2113111-2112980	[redacted]
46	75	nr3945	ref|NC_002976.3|:2118445-2118600	[redacted]
46	76	nr3946	ref|NC_002976.3|:c2118794-2118693	[redacted]

46	77	nr3947	ref|NC_002976.3|:2121760-2121873	[redacted]	[redacted]
46	78	nr3948	ref|NC_002976.3|:c2122909-2122124	[redacted]	[redacted]
46	79	nr3949	ref|NC_002976.3|:c2124674-2123184	[redacted]	[redacted]
46	80	nr3950	ref|NC_002976.3|:c2129109-2127505	[redacted]	[redacted]
46	81	nr3951	ref|NC_002976.3|:c2129288-2129112	[redacted]	[redacted]
46	82	nr3952	ref|NC_002976.3|:c2130336-2129263	[redacted]	[redacted]
46	83	nr3953	ref|NC_002976.3|:c2130812-2130360	[redacted]	[redacted]
46	84	nr3954	ref|NC_002976.3|:c2131971-2130814	[redacted]	[redacted]
46	85	nr3955	ref|NC_002976.3|:2132420-2132737	[redacted]	[redacted]
46	86	nr3956	ref|NC_002976.3|:c2134720-2133872	[redacted]	[redacted]
47	1	nr3957	ref|NC_002976.3|:2135452-2136504	[redacted]	[redacted]
47	2	nr3958	ref|NC_002976.3|:c2137336-2136569	[redacted]	[redacted]
47	3	nr3959	ref|NC_002976.3|:c2142765-2142373	[redacted]	[redacted]
47	4	nr3960	ref|NC_002976.3|:c2148588-2148070	[redacted]	[redacted]
47	5	nr3961	ref|NC_002976.3|:c2149357-2148893	[redacted]	[redacted]
47	6	nr3962	ref|NC_002976.3|:c2149722-2149540	[redacted]	[redacted]
47	7	nr3963	ref|NC_002976.3|:c2151964-2151737	[redacted]	[redacted]
47	8	nr3964	ref|NC_002976.3|:c2155169-2154300	[redacted]	[redacted]
47	9	nr3965	ref|NC_002976.3|:2155285-2155578	[redacted]	[redacted]
47	10	nr3966	ref|NC_002976.3|:2160907-2161047	[redacted]	[redacted]
47	11	nr3967	ref|NC_002976.3|:c2161862-2161089	[redacted]	[redacted]
47	12	nr3968	ref|NC_002976.3|:c2164269-2162461	[redacted]	[redacted]
47	13	nr3969	ref|NC_002976.3|:c2166107-2166003	[redacted]	[redacted]
47	14	nr3970	ref|NC_002976.3|:c2167561-2166137	[redacted]	[redacted]
47	15	nr3971	ref|NC_002976.3|:c2170401-2169022	[redacted]	[redacted]
47	16	nr3972	ref|NC_002976.3|:2172847-2173116	[redacted]	[redacted]
47	17	nr3973	ref|NC_002976.3|:c2176009-2174678	[redacted]	[redacted]
47	18	nr3974	ref|NC_002976.3|:c2177023-2176013	[redacted]	[redacted]
47	19	nr3975	ref|NC_002976.3|:c2186037-2185156	[redacted]	[redacted]
47	20	nr3976	ref|NC_002976.3|:2189763-2190194	[redacted]	[redacted]
47	21	nr3977	ref|NC_002976.3|:c2191176-2190298	[redacted]	[redacted]
47	22	nr3978	ref|NC_002976.3|:2191498-2192088	[redacted]	[redacted]
47	23	nr3979	ref|NC_002976.3|:c2192271-2192083	[redacted]	[redacted]
47	24	nr3980	ref|NC_002976.3|:c2192725-2192579	[redacted]	[redacted]
47	25	nr3981	ref|NC_002976.3|:2192892-2194253	[redacted]	[redacted]
47	26	nr3982	ref|NC_002976.3|:c2199425-2198607	[redacted]	[redacted]
47	27	nr3983	ref|NC_002976.3|:c2202054-2199427	[redacted]	[redacted]
47	28	nr3984	ref|NC_002976.3|:c2202272-2202180	[redacted]	[redacted]
47	29	nr3985	ref|NC_002976.3|:c2205030-2204812	[redacted]	[redacted]
47	30	nr3986	ref|NC_002976.3|:c2214765-2213467	[redacted]	[redacted]
47	31	nr3987	ref|NC_002976.3|:c2215114-2214989	[redacted]	[redacted]
47	32	nr3988	ref|NC_002976.3|:c2218135-2217830	[redacted]	[redacted]
47	33	nr3989	ref|NC_002976.3|:c2219058-2218459	[redacted]	[redacted]
47	34	nr3990	ref|NC_002976.3|:c2220252-2219074	[redacted]	[redacted]
47	35	nr3991	ref|NC_002976.3|:c2221175-2220276	[redacted]	[redacted]
47	36	nr3992	ref|NC_002976.3|:c2222662-2221883	[redacted]	[redacted]
47	37	nr3993	ref|NC_002976.3|:c2224402-2222684	[redacted]	[redacted]
47	38	nr3994	ref|NC_002976.3|:c2227295-2226564	[redacted]	[redacted]
47	39	nr3995	ref|NC_002976.3|:2227498-2227617	[redacted]	[redacted]

47	40	nr3996	ref|NC_002976.3|:c2228085-2227609
47	41	nr3997	ref|NC_002976.3|:c2229441-2228098
47	42	nr3998	ref|NC_002976.3|:c2229898-2229458
47	43	nr3999	ref|NC_002976.3|:c2230867-2230118
47	44	nr4000	ref|NC_002976.3|:c2233452-2232343
47	45	nr4001	ref|NC_002976.3|:c2234016-2233912
47	46	nr4002	ref|NC_002976.3|:c2236355-2234763
47	47	nr4003	ref|NC_002976.3|:c2237467-2236877
47	48	nr4004	ref|NC_002976.3|:c2242222-2242043
47	49	nr4005	ref|NC_002976.3|:c2245656-2244112
47	50	nr4006	ref|NC_002976.3|:c2248180-2245847
47	51	nr4007	ref|NC_002976.3|:c2250222-2248885
47	52	nr4008	ref|NC_002976.3|:2251501-2251632
47	53	nr4009	ref|NC_002976.3|:2251642-2251923
47	54	nr4010	ref|NC_002976.3|:2252357-2252542
47	55	nr4011	ref|NC_002976.3|:c2253673-2252735
47	56	nr4012	ref|NC_002976.3|:c2255003-2253837
47	57	nr4013	ref|NC_002976.3|:c2256008-2255022
47	58	nr4014	ref|NC_002976.3|:2257002-2257433
47	59	nr4015	ref|NC_002976.3|:2257793-2258410
47	60	nr4016	ref|NC_002976.3|:2258429-2258776
47	61	nr4017	ref|NC_002976.3|:c2259416-2259315
47	62	nr4018	ref|NC_002976.3|:c2259763-2259614
47	63	nr4019	ref|NC_002976.3|:c2260124-2259780
47	64	nr4020	ref|NC_002976.3|:c2260847-2260680
47	65	nr4021	ref|NC_002976.3|:c2261120-2260878
47	66	nr4022	ref|NC_002976.3|:c2263774-2262635
47	67	nr4023	ref|NC_002976.3|:2263927-2264748
47	68	nr4024	ref|NC_002976.3|:2265275-2266006
47	69	nr4025	ref|NC_002976.3|:2266411-2267010
47	70	nr4026	ref|NC_002976.3|:c2267580-2267485
47	71	nr4027	ref|NC_002976.3|:c2268628-2268146
47	72	nr4028	ref|NC_002976.3|:c2271429-2271304
47	73	nr4029	ref|NC_002976.3|:c2272624-2271458
47	74	nr4030	ref|NC_002976.3|:c2273636-2272878
47	75	nr4031	ref|NC_002976.3|:c2274614-2273928
47	76	nr4032	ref|NC_002976.3|:2276328-2276468
47	77	nr4033	ref|NC_002976.3|:c2279648-2279187
47	78	nr4034	ref|NC_002976.3|:c2283362-2282118
47	79	nr4035	ref|NC_002976.3|:2283725-2284201
47	80	nr4036	ref|NC_002976.3|:2285300-2285470
47	81	nr4037	ref|NC_002976.3|:2287012-2287146
47	82	nr4038	ref|NC_002976.3|:2287464-2289335
47	83	nr4039	ref|NC_002976.3|:c2300148-2298118
47	84	nr4040	ref|NC_002976.3|:2300398-2300826
47	85	nr4041	ref|NC_002976.3|:c2301039-2300851
47	86	nr4042	ref|NC_002976.3|:2301140-2301316
48	1	nr4043	ref|NC_002976.3|:c2301670-2301515
48	2	nr4044	ref|NC_002976.3|:c2304090-2302918

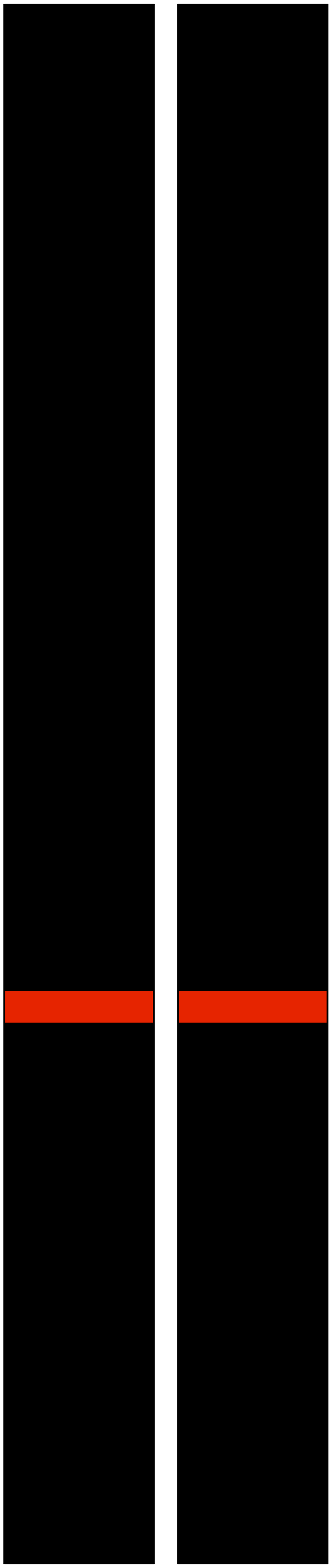

48	3	nr4045	ref|NC_002976.3|:c2305416-2304937	[redacted]	[redacted]
48	4	nr4046	ref|NC_002976.3|:c2305933-2305586	[redacted]	[redacted]
48	5	nr4047	ref|NC_002976.3|:c2316162-2314963	[redacted]	[redacted]
48	6	nr4048	ref|NC_002976.3|:c2323638-2322337	[redacted]	[redacted]
48	7	nr4049	ref|NC_002976.3|:c2332063-2330888	[redacted]	[redacted]
48	8	nr4050	ref|NC_002976.3|:2332514-2332627	[redacted]	[redacted]
48	9	nr4051	ref|NC_002976.3|:2333271-2333378	[redacted]	[redacted]
48	10	nr4052	ref|NC_002976.3|:2336580-2337647	[redacted]	[redacted]
48	11	nr4053	ref|NC_002976.3|:2339963-2340103	[redacted]	[redacted]
48	12	nr4054	ref|NC_002976.3|:c2343871-2343293	[redacted]	[redacted]
48	13	nr4055	ref|NC_002976.3|:c2346413-2345799	[redacted]	[redacted]
48	14	nr4056	ref|NC_002976.3|:c2347250-2346438	[redacted]	[redacted]
48	15	nr4057	ref|NC_002976.3|:c2347903-2347487	[redacted]	[redacted]
48	16	nr4058	ref|NC_002976.3|:2348006-2348416	[redacted]	[redacted]
48	17	nr4059	ref|NC_002976.3|:c2349650-2349132	[redacted]	[redacted]
48	18	nr4060	ref|NC_002976.3|:2350001-2351497	[redacted]	[redacted]
48	19	nr4061	ref|NC_002976.3|:c2352370-2352266	[redacted]	[redacted]
48	20	nr4062	ref|NC_002976.3|:c2356968-2356576	[redacted]	[redacted]
48	21	nr4063	ref|NC_002976.3|:c2357136-2356984	[redacted]	[redacted]
48	22	nr4064	ref|NC_002976.3|:2357317-2358909	[redacted]	[redacted]
48	23	nr4065	ref|NC_002976.3|:c2359586-2359074	[redacted]	[redacted]
48	24	nr4066	ref|NC_002976.3|:2359691-2359798	[redacted]	[redacted]
48	25	nr4067	ref|NC_002976.3|:c2360017-2359862	[redacted]	[redacted]
48	26	nr4068	ref|NC_002976.3|:c2361546-2360269	[redacted]	[redacted]
48	27	nr4069	ref|NC_002976.3|:c2362600-2361560	[redacted]	[redacted]
48	28	nr4070	ref|NC_002976.3|:c2363624-2362671	[redacted]	[redacted]
48	29	nr4071	ref|NC_002976.3|:c2365018-2363666	[redacted]	[redacted]
48	30	nr4072	ref|NC_002976.3|:c2366873-2366772	[redacted]	[redacted]
48	31	nr4073	ref|NC_002976.3|:2367078-2367989	[redacted]	[redacted]
48	32	nr4074	ref|NC_002976.3|:c2369637-2369134	[redacted]	[redacted]
48	33	nr4075	ref|NC_002976.3|:c2371429-2369726	[redacted]	[redacted]
48	34	nr4076	ref|NC_002976.3|:c2372924-2371584	[redacted]	[redacted]
48	35	nr4077	ref|NC_002976.3|:2372970-2373065	[redacted]	[redacted]
48	36	nr4078	ref|NC_002976.3|:c2375389-2373203	[redacted]	[redacted]
48	37	nr4079	ref|NC_002976.3|:c2376383-2375760	[redacted]	[redacted]
48	38	nr4080	ref|NC_002976.3|:c2384995-2383754	[redacted]	[redacted]
48	39	nr4081	ref|NC_002976.3|:c2386935-2385517	[redacted]	[redacted]
48	40	nr4082	ref|NC_002976.3|:c2387744-2387496	[redacted]	[redacted]
48	41	nr4083	ref|NC_002976.3|:2388076-2389524	[redacted]	[redacted]
48	42	nr4084	ref|NC_002976.3|:c2389978-2389604	[redacted]	[redacted]
48	43	nr4085	ref|NC_002976.3|:c2392693-2391590	[redacted]	[redacted]
48	44	nr4086	ref|NC_002976.3|:c2395663-2395235	[redacted]	[redacted]
48	45	nr4087	ref|NC_002976.3|:c2395991-2395767	[redacted]	[redacted]
48	46	nr4088	ref|NC_002976.3|:2398597-2400162	[redacted]	[redacted]
48	47	nr4089	ref|NC_002976.3|:c2401888-2401241	[redacted]	[redacted]
48	48	nr4090	ref|NC_002976.3|:c2403504-2402869	[redacted]	[redacted]
48	49	nr4091	ref|NC_002976.3|:c2404688-2403501	[redacted]	[redacted]
48	50	nr4092	ref|NC_002976.3|:c2405311-2404823	[redacted]	[redacted]
48	51	nr4093	ref|NC_002976.3|:c2406273-2405308	[redacted]	[redacted]

48	52	nr4094	ref|NC_002976.3|:c2407169-2406273	[redacted]	[redacted]
48	53	nr4095	ref|NC_002976.3|:c2407844-2407305	[redacted]	[redacted]
48	54	nr4096	ref|NC_002976.3|:2424381-2424482	[redacted]	[redacted]
48	55	nr4097	ref|NC_002976.3|:2424484-2424930	[redacted]	[redacted]
48	56	nr4098	ref|NC_002976.3|:c2426535-2425762	[redacted]	[redacted]
48	57	nr4099	ref|NC_002976.3|:c2428080-2426704	[redacted]	[redacted]
48	58	nr4100	ref|NC_002976.3|:c2431126-2428109	[redacted]	[redacted]
48	59	nr4101	ref|NC_002976.3|:c2432126-2431209	[redacted]	[redacted]
48	60	nr4102	ref|NC_002976.3|:2436232-2437113	[redacted]	[redacted]
48	61	nr4103	ref|NC_002976.3|:2440410-2440529	[redacted]	[redacted]
48	62	nr4104	ref|NC_002976.3|:2441883-2442200	[redacted]	[redacted]
48	63	nr4105	ref|NC_002976.3|:c2449578-2442370	[redacted]	[redacted]
48	64	nr4106	ref|NC_002976.3|:c2453742-2453071	[redacted]	[redacted]
48	65	nr4107	ref|NC_002976.3|:c2454233-2454081	[redacted]	[redacted]
48	66	nr4108	ref|NC_002976.3|:2461814-2461912	[redacted]	[redacted]
48	67	nr4109	ref|NC_002976.3|:c2462254-2462102	[redacted]	[redacted]
48	68	nr4110	ref|NC_002976.3|:c2463194-2462460	[redacted]	[redacted]
48	69	nr4111	ref|NC_002976.3|:2463271-2463432	[redacted]	[redacted]
48	70	nr4112	ref|NC_002976.3|:2463875-2464216	[redacted]	[redacted]
48	71	nr4113	ref|NC_002976.3|:c2471446-2471339	[redacted]	[redacted]
48	72	nr4114	ref|NC_002976.3|:c2472520-2471753	[redacted]	[redacted]
48	73	nr4115	ref|NC_002976.3|:c2474778-2473294	[redacted]	[redacted]
48	74	nr4116	ref|NC_002976.3|:2476275-2476634	[redacted]	[redacted]
48	75	nr4117	ref|NC_002976.3|:2476845-2476940	[redacted]	[redacted]
48	76	nr4118	ref|NC_002976.3|:c2477180-2477064	[redacted]	[redacted]
48	77	nr4119	ref|NC_002976.3|:c2478104-2477247	[redacted]	[redacted]
48	78	nr4120	ref|NC_002976.3|:c2479528-2479343	[redacted]	[redacted]
48	79	nr4121	ref|NC_002976.3|:c2480083-2479838	[redacted]	[redacted]
48	80	nr4122	ref|NC_002976.3|:c2481056-2480169	[redacted]	[redacted]
48	81	nr4123	ref|NC_002976.3|:c2482195-2481410	[redacted]	[redacted]
48	82	nr4124	ref|NC_002976.3|:c2483315-2482536	[redacted]	[redacted]
48	83	nr4125	ref|NC_002976.3|:c2483698-2483570	[redacted]	[redacted]
48	84	nr4126	ref|NC_002976.3|:c2484596-2483712	[redacted]	[redacted]
48	85	nr4127	ref|NC_002976.3|:c2484977-2484684	[redacted]	[redacted]
48	86	nr4128	ref|NC_002976.3|:2485525-2485872	[redacted]	[redacted]
49	1	nr4129	ref|NC_002976.3|:2485886-2487583	[redacted]	[redacted]
49	2	nr4130	ref|NC_002976.3|:c2490829-2490569	[redacted]	[redacted]
49	3	nr4131	ref|NC_002976.3|:c2493582-2493466	[redacted]	[redacted]
49	4	nr4132	ref|NC_002976.3|:c2494403-2493858	[redacted]	[redacted]
49	5	nr4133	ref|NC_002976.3|:c2497350-2496907	[redacted]	[redacted]
49	6	nr4134	ref|NC_002976.3|:c2499297-2497936	[redacted]	[redacted]
49	7	nr4135	ref|NC_002976.3|:2499425-2499577	[redacted]	[redacted]
49	8	nr4136	ref|NC_002976.3|:c2502058-2501711	[redacted]	[redacted]
49	9	nr4137	ref|NC_002976.3|:c2502826-2502065	[redacted]	[redacted]
49	10	nr4138	ref|NC_002976.3|:c2503196-2502918	[redacted]	[redacted]
49	11	nr4139	ref|NC_002976.3|:c2503532-2503206	[redacted]	[redacted]
49	12	nr4140	ref|NC_002976.3|:c2506105-2505920	[redacted]	[redacted]
49	13	nr4141	ref|NC_002976.3|:c2506887-2506108	[redacted]	[redacted]
49	14	nr4142	ref|NC_002976.3|:c2508177-2507320	[redacted]	[redacted]

49	15	nr4143	ref|NC_002976.3|:c2509765-2509031
49	16	nr4144	ref|NC_002976.3|:c2511030-2509762
49	17	nr4145	ref|NC_002976.3|:c2512052-2511030
49	18	nr4146	ref|NC_002976.3|:c2512969-2512055
49	19	nr4147	ref|NC_002976.3|:c2513618-2512974
49	20	nr4148	ref|NC_002976.3|:c2514006-2513620
49	21	nr4149	ref|NC_002976.3|:c2516321-2514048
49	22	nr4150	ref|NC_002976.3|:c2516639-2516334
49	23	nr4151	ref|NC_002976.3|:c2517544-2516639
49	24	nr4152	ref|NC_002976.3|:c2518672-2518526
49	25	nr4153	ref|NC_002976.3|:c2519564-2518785
49	26	nr4154	ref|NC_002976.3|:c2520420-2520289
49	27	nr4155	ref|NC_002976.3|:2520514-2521017
49	28	nr4156	ref|NC_002976.3|:c2522638-2521511
49	29	nr4157	ref|NC_002976.3|:2523162-2523659
49	30	nr4158	ref|NC_002976.3|:c2525116-2523971
49	31	nr4159	ref|NC_002976.3|:c2527352-2526666
49	32	nr4160	ref|NC_002976.3|:c2532159-2530339
49	33	nr4161	ref|NC_002976.3|:c2533448-2532765
49	34	nr4162	ref|NC_002976.3|:c2534899-2533580
49	35	nr4163	ref|NC_002976.3|:c2536519-2535278
49	36	nr4164	ref|NC_002976.3|:2546650-2546793
49	37	nr4165	ref|NC_002976.3|:c2552173-2552039
49	38	nr4166	ref|NC_002976.3|:2557181-2557282
49	39	nr4167	ref|NC_002976.3|:2560476-2560577
49	40	nr4168	ref|NC_002976.3|:c2591922-2591830
49	41	nr4169	ref|NC_002976.3|:c2601172-2600843
49	42	nr4170	ref|NC_002976.3|:2602224-2603228
49	43	nr4171	ref|NC_002976.3|:2605087-2605179
49	44	nr4172	ref|NC_003140.1|:1196-1516
49	45	nr4173	ref|NC_003140.1|:c3080-2388
49	46	nr4174	ref|NC_003140.1|:3190-3384
49	47	nr4175	ref|NC_003140.1|:c6876-5905
49	48	nr4176	ref|NC_003140.1|:7000-7419
49	49	nr4177	ref|NC_003140.1|:19996-20373
49	50	nr4178	ref|NC_003140.1|:20313-20639
49	51	nr4179	ref|NC_003140.1|:20652-21206
49	52	nr4180	ref|NC_003140.1|:c23371-23261
49	53	nr4181	ref|NC_003140.1|:23315-23500
49	54	nr4182	ref|NC_003923.1|:c51677-51387
49	55	nr4183	ref|NC_003923.1|:51816-52865
49	56	nr4184	ref|NC_003923.1|:53483-54973
49	57	nr4185	ref|NC_003923.1|:c55660-55346
49	58	nr4186	ref|NC_003923.1|:56854-57225
49	59	nr4187	ref|NC_003923.1|:57354-57974
49	60	nr4188	ref|NC_003923.1|:58513-59769
49	61	nr4189	ref|NC_003923.1|:c61148-61038
49	62	nr4190	ref|NC_003923.1|:62816-63004
49	63	nr4191	ref|NC_003923.1|:140106-140591

49	64	nr4192	ref|NC_003923.1|:140576-140866
49	65	nr4193	ref|NC_003923.1|:205906-206100
49	66	nr4194	ref|NC_003923.1|:418835-418987
49	67	nr4195	ref|NC_003923.1|:c839693-839478
49	68	nr4196	ref|NC_003923.1|:c841563-840826
49	69	nr4197	ref|NC_003923.1|:842700-845072
49	70	nr4198	ref|NC_003923.1|:845745-846089
49	71	nr4199	ref|NC_003923.1|:846314-846520
49	72	nr4200	ref|NC_003923.1|:846507-847565
49	73	nr4201	ref|NC_003923.1|:847747-848238
49	74	nr4202	ref|NC_003923.1|:848599-849576
49	75	nr4203	ref|NC_003923.1|:849592-850152
49	76	nr4204	ref|NC_003923.1|:860063-860539
49	77	nr4205	ref|NC_003923.1|:1007245-1007613
49	78	nr4206	ref|NC_003923.1|:1175411-1175596
49	79	nr4207	ref|NC_003923.1|:1314085-1314207
49	80	nr4208	ref|NC_003923.1|:1348193-1348681
49	81	nr4209	ref|NC_003923.1|:1384999-1385235
49	82	nr4210	ref|NC_003923.1|:c1413755-1413561
49	83	nr4211	ref|NC_003923.1|:c1529132-1528947
49	84	nr4212	ref|NC_003923.1|:c1530358-1529381
49	85	nr4213	ref|NC_003923.1|:c1531298-1530360
49	86	nr4214	ref|NC_003923.1|:1558153-1558275
50	1	nr4215	ref|NC_003923.1|:c1561657-1561457
50	2	nr4216	ref|NC_003923.1|:c1562808-1562647
50	3	nr4217	ref|NC_003923.1|:c1563090-1562809
50	4	nr4218	ref|NC_003923.1|:c1563264-1563091
50	5	nr4219	ref|NC_003923.1|:c1570956-1570708
50	6	nr4220	ref|NC_003923.1|:1571120-1571443
50	7	nr4221	ref|NC_003923.1|:c1765527-1765006
50	8	nr4222	ref|NC_003923.1|:1893639-1893764
50	9	nr4223	ref|NC_003923.1|:2100364-2100711
50	10	nr4224	ref|NC_003923.1|:c2176635-2176579
50	11	nr4225	ref|NC_003923.1|:c2240143-2238083
50	12	nr4226	ref|NC_003923.1|:2459236-2459760
50	13	nr4227	ref|NC_003923.1|:c2579974-2577143
50	14	nr4228	ref|NC_004461.1|:c32786-32604
50	15	nr4229	ref|NC_004461.1|:33035-35362
50	16	nr4230	ref|NC_004461.1|:c36551-36423
50	17	nr4231	ref|NC_004461.1|:c37226-36816
50	18	nr4232	ref|NC_004461.1|:c38453-38142
50	19	nr4233	ref|NC_004461.1|:c38547-38455
50	20	nr4234	ref|NC_004461.1|:c38890-38540
50	21	nr4235	ref|NC_004461.1|:c40180-39497
50	22	nr4236	ref|NC_004461.1|:40427-41650
50	23	nr4237	ref|NC_004461.1|:49457-50611
50	24	nr4238	ref|NC_004461.1|:50641-51441
50	25	nr4239	ref|NC_004461.1|:c53219-52968
50	26	nr4240	ref|NC_004461.1|:c53784-53287

50	27	nr4241	ref|NC_004461.1|:c54191-54063
50	28	nr4242	ref|NC_004461.1|:c54866-54456
50	29	nr4243	ref|NC_004461.1|:c55279-55172
50	30	nr4244	ref|NC_004461.1|:c55467-55264
50	31	nr4245	ref|NC_004461.1|:c56624-55611
50	32	nr4246	ref|NC_004461.1|:c57327-56998
50	33	nr4247	ref|NC_004461.1|:c60220-58592
50	34	nr4248	ref|NC_004461.1|:c61578-60217
50	35	nr4249	ref|NC_004461.1|:c62346-61765
50	36	nr4250	ref|NC_004461.1|:62622-62741
50	37	nr4251	ref|NC_004461.1|:c63212-62853
50	38	nr4252	ref|NC_004461.1|:63848-63946
50	39	nr4253	ref|NC_004461.1|:64066-66000
50	40	nr4254	ref|NC_004461.1|:c67245-67123
50	41	nr4255	ref|NC_004461.1|:c67488-67312
50	42	nr4256	ref|NC_004461.1|:c67579-67457
50	43	nr4257	ref|NC_004461.1|:c67673-67566
50	44	nr4258	ref|NC_004461.1|:c67992-67822
50	45	nr4259	ref|NC_004461.1|:68194-68763
50	46	nr4260	ref|NC_004461.1|:68808-69965
50	47	nr4261	ref|NC_004461.1|:70136-70372
50	48	nr4262	ref|NC_004461.1|:70397-71071
50	49	nr4263	ref|NC_004461.1|:c71476-71099
50	50	nr4264	ref|NC_004461.1|:71924-72112
50	51	nr4265	ref|NC_004461.1|:72366-73133
50	52	nr4266	ref|NC_004461.1|:77768-79282
50	53	nr4267	ref|NC_004461.1|:79510-80172
50	54	nr4268	ref|NC_004461.1|:c81354-80680
50	55	nr4269	ref|NC_004461.1|:81622-82983
50	56	nr4270	ref|NC_004461.1|:83283-83690
50	57	nr4271	ref|NC_004461.1|:83707-84060
50	58	nr4272	ref|NC_004461.1|:84057-84737
50	59	nr4273	ref|NC_004461.1|:84863-85210
50	60	nr4274	ref|NC_004461.1|:85268-86911
50	61	nr4275	ref|NC_004461.1|:86993-87643
50	62	nr4276	ref|NC_004461.1|:c88386-87838
50	63	nr4277	ref|NC_004461.1|:88583-88798
50	64	nr4278	ref|NC_004461.1|:88823-89104
50	65	nr4279	ref|NC_004461.1|:90096-90206
50	66	nr4280	ref|NC_004461.1|:90561-91256
50	67	nr4281	ref|NC_004461.1|:c91874-91329
50	68	nr4282	ref|NC_004461.1|:93874-94821
50	69	nr4283	ref|NC_004461.1|:94836-95441
50	70	nr4284	ref|NC_004461.1|:96362-96604
50	71	nr4285	ref|NC_004461.1|:97062-97193
50	72	nr4286	ref|NC_004461.1|:c98942-98013
50	73	nr4287	ref|NC_004461.1|:c99960-98962
50	74	nr4288	ref|NC_004461.1|:c100687-99998
50	75	nr4289	ref|NC_004461.1|:c102150-100729

50	76	nr4290	ref|NC_004461.1|:c103471-102236
50	77	nr4291	ref|NC_004461.1|:c104186-103740
50	78	nr4292	ref|NC_004461.1|:105274-105441
50	79	nr4293	ref|NC_004461.1|:105473-105568
50	80	nr4294	ref|NC_004461.1|:c107304-107041
50	81	nr4295	ref|NC_004461.1|:c108839-107415
50	82	nr4296	ref|NC_004461.1|:109771-109947
50	83	nr4297	ref|NC_004461.1|:c111738-111343
50	84	nr4298	ref|NC_004461.1|:c111888-111751
50	85	nr4299	ref|NC_004461.1|:112049-112954
50	86	nr4300	ref|NC_004461.1|:112929-114050
51	1	nr4301	ref|NC_004461.1|:114016-114627
51	2	nr4302	ref|NC_004461.1|:114705-115373
51	3	nr4303	ref|NC_004461.1|:115361-116452
51	4	nr4304	ref|NC_004461.1|:116959-117099
51	5	nr4305	ref|NC_004461.1|:119612-119770
51	6	nr4306	ref|NC_004461.1|:c127096-126974
51	7	nr4307	ref|NC_004461.1|:134847-135632
51	8	nr4308	ref|NC_004461.1|:c141076-140495
51	9	nr4309	ref|NC_004461.1|:c142598-142488
51	10	nr4310	ref|NC_004461.1|:144362-144460
51	11	nr4311	ref|NC_004461.1|:c147805-147704
51	12	nr4312	ref|NC_004461.1|:c148126-148001
51	13	nr4313	ref|NC_004461.1|:c149939-149562
51	14	nr4314	ref|NC_004461.1|:159229-159360
51	15	nr4315	ref|NC_004461.1|:c161869-159572
51	16	nr4316	ref|NC_004461.1|:161289-161408
51	17	nr4317	ref|NC_004461.1|:163010-163282
51	18	nr4318	ref|NC_004461.1|:c168846-168673
51	19	nr4319	ref|NC_004461.1|:c169250-169158
51	20	nr4320	ref|NC_004461.1|:175180-177111
51	21	nr4321	ref|NC_004461.1|:177252-177650
51	22	nr4322	ref|NC_004461.1|:177776-178036
51	23	nr4323	ref|NC_004461.1|:c180414-179032
51	24	nr4324	ref|NC_004461.1|:c181514-180408
51	25	nr4325	ref|NC_004461.1|:181771-182766
51	26	nr4326	ref|NC_004461.1|:193418-193510
51	27	nr4327	ref|NC_004461.1|:c196332-196216
51	28	nr4328	ref|NC_004461.1|:223191-223283
51	29	nr4329	ref|NC_004461.1|:257220-257336
51	30	nr4330	ref|NC_004461.1|:c261295-260657
51	31	nr4331	ref|NC_004461.1|:c268621-268214
51	32	nr4332	ref|NC_004461.1|:c277179-277075
51	33	nr4333	ref|NC_004461.1|:280023-281036
51	34	nr4334	ref|NC_004461.1|:299575-299694
51	35	nr4335	ref|NC_004461.1|:c330243-330145
51	36	nr4336	ref|NC_004461.1|:c337142-335640
51	37	nr4337	ref|NC_004461.1|:c338466-338290
51	38	nr4338	ref|NC_004461.1|:338772-338870

51	39	nr4339	ref|NC_004461.1|:343250-343897	[redacted]	[redacted]
51	40	nr4340	ref|NC_004461.1|:345250-345375	[redacted]	[redacted]
51	41	nr4341	ref|NC_004461.1|:374948-375088	[redacted]	[redacted]
51	42	nr4342	ref|NC_004461.1|:c389301-388372	[redacted]	[redacted]
51	43	nr4343	ref|NC_004461.1|:470098-470190	[redacted]	[redacted]
51	44	nr4344	ref|NC_004461.1|:c474727-474611	[redacted]	[redacted]
51	45	nr4345	ref|NC_004461.1|:479653-480261	[redacted]	[redacted]
51	46	nr4346	ref|NC_004461.1|:485083-485199	[redacted]	[redacted]
51	47	nr4347	ref|NC_004461.1|:c485624-485430	[redacted]	[redacted]
51	48	nr4348	ref|NC_004461.1|:c486442-485621	[redacted]	[redacted]
51	49	nr4349	ref|NC_004461.1|:c500794-500633	[redacted]	[redacted]
51	50	nr4350	ref|NC_004461.1|:c565705-565031	[redacted]	[redacted]
51	51	nr4351	ref|NC_004461.1|:c572319-572125	[redacted]	[redacted]
51	52	nr4352	ref|NC_004461.1|:c582911-582783	[redacted]	[redacted]
51	53	nr4353	ref|NC_004461.1|:583325-583441	[redacted]	[redacted]
51	54	nr4354	ref|NC_004461.1|:598427-598522	[redacted]	[redacted]
51	55	nr4355	ref|NC_004461.1|:613761-614774	[redacted]	[redacted]
51	56	nr4356	ref|NC_004461.1|:c616550-614871	[redacted]	[redacted]
51	57	nr4357	ref|NC_004461.1|:635887-636159	[redacted]	[redacted]
51	58	nr4358	ref|NC_004461.1|:636213-636497	[redacted]	[redacted]
51	59	nr4359	ref|NC_004461.1|:c646498-646400	[redacted]	[redacted]
51	60	nr4360	ref|NC_004461.1|:656620-657633	[redacted]	[redacted]
51	61	nr4361	ref|NC_004461.1|:658052-658168	[redacted]	[redacted]
51	62	nr4362	ref|NC_004461.1|:c661035-660643	[redacted]	[redacted]
51	63	nr4363	ref|NC_004461.1|:666519-667532	[redacted]	[redacted]
51	64	nr4364	ref|NC_004461.1|:c679877-678864	[redacted]	[redacted]
51	65	nr4365	ref|NC_004461.1|:c694818-694615	[redacted]	[redacted]
51	66	nr4366	ref|NC_004461.1|:695126-695314	[redacted]	[redacted]
51	67	nr4367	ref|NC_004461.1|:695710-695820	[redacted]	[redacted]
51	68	nr4368	ref|NC_004461.1|:695903-696280	[redacted]	[redacted]
51	69	nr4369	ref|NC_004461.1|:696399-696641	[redacted]	[redacted]
51	70	nr4370	ref|NC_004461.1|:698893-698994	[redacted]	[redacted]
51	71	nr4371	ref|NC_004461.1|:c699428-699228	[redacted]	[redacted]
51	72	nr4372	ref|NC_004461.1|:699986-700201	[redacted]	[redacted]
51	73	nr4373	ref|NC_004461.1|:713461-714267	[redacted]	[redacted]
51	74	nr4374	ref|NC_004461.1|:719763-719858	[redacted]	[redacted]
51	75	nr4375	ref|NC_004461.1|:722260-722496	[redacted]	[redacted]
51	76	nr4376	ref|NC_004461.1|:723254-723670	[redacted]	[redacted]
51	77	nr4377	ref|NC_004461.1|:801301-801573	[redacted]	[redacted]
51	78	nr4378	ref|NC_004461.1|:801627-801974	[redacted]	[redacted]
51	79	nr4379	ref|NC_004461.1|:c822406-822203	[redacted]	[redacted]
51	80	nr4380	ref|NC_004461.1|:842644-842778	[redacted]	[redacted]
51	81	nr4381	ref|NC_004461.1|:868609-868716	[redacted]	[redacted]
51	82	nr4382	ref|NC_004461.1|:c920167-919880	[redacted]	[redacted]
51	83	nr4383	ref|NC_004461.1|:c952018-951626	[redacted]	[redacted]
51	84	nr4384	ref|NC_004461.1|:967879-968244	[redacted]	[redacted]
51	85	nr4385	ref|NC_004461.1|:c998674-998429	[redacted]	[redacted]
51	86	nr4386	ref|NC_004461.1|:1000840-1001217	[redacted]	[redacted]
52	1	nr4387	ref|NC_004461.1|:1001224-1001412	[redacted]	[redacted]

52	2	nr4388	ref|NC_004461.1|:1005651-1006529
52	3	nr4389	ref|NC_004461.1|:1008107-1008358
52	4	nr4390	ref|NC_004461.1|:1010531-1010674
52	5	nr4391	ref|NC_004461.1|:1018572-1019171
52	6	nr4392	ref|NC_004461.1|:c1056215-1056099
52	7	nr4393	ref|NC_004461.1|:c1075799-1075461
52	8	nr4394	ref|NC_004461.1|:c1095668-1094919
52	9	nr4395	ref|NC_004461.1|:c1096549-1095677
52	10	nr4396	ref|NC_004461.1|:c1096937-1096527
52	11	nr4397	ref|NC_004461.1|:1096864-1096962
52	12	nr4398	ref|NC_004461.1|:c1097524-1096943
52	13	nr4399	ref|NC_004461.1|:c1114716-1113949
52	14	nr4400	ref|NC_004461.1|:c1116048-1114723
52	15	nr4401	ref|NC_004461.1|:c1116889-1116035
52	16	nr4402	ref|NC_004461.1|:c1117811-1116903
52	17	nr4403	ref|NC_004461.1|:c1119509-1117815
52	18	nr4404	ref|NC_004461.1|:c1120471-1119515
52	19	nr4405	ref|NC_004461.1|:c1165921-1163816
52	20	nr4406	ref|NC_004461.1|:c1188655-1188557
52	21	nr4407	ref|NC_004461.1|:c1199318-1198914
52	22	nr4408	ref|NC_004461.1|:c1201960-1201460
52	23	nr4409	ref|NC_004461.1|:c1254307-1254038
52	24	nr4410	ref|NC_004461.1|:1258916-1259179
52	25	nr4411	ref|NC_004461.1|:c1320838-1320239
52	26	nr4412	ref|NC_004461.1|:1350920-1351036
52	27	nr4413	ref|NC_004461.1|:c1364435-1363755
52	28	nr4414	ref|NC_004461.1|:1393615-1393713
52	29	nr4415	ref|NC_004461.1|:c1393803-1393654
52	30	nr4416	ref|NC_004461.1|:c1394040-1393924
52	31	nr4417	ref|NC_004461.1|:1422917-1423024
52	32	nr4418	ref|NC_004461.1|:c1434555-1434406
52	33	nr4419	ref|NC_004461.1|:c1460142-1460044
52	34	nr4420	ref|NC_004461.1|:1501935-1502948
52	35	nr4421	ref|NC_004461.1|:c1503294-1503178
52	36	nr4422	ref|NC_004461.1|:1517225-1517380
52	37	nr4423	ref|NC_004461.1|:c1518788-1518462
52	38	nr4424	ref|NC_004461.1|:c1518908-1518816
52	39	nr4425	ref|NC_004461.1|:1519417-1519509
52	40	nr4426	ref|NC_004461.1|:c1520315-1519755
52	41	nr4427	ref|NC_004461.1|:c1521685-1520354
52	42	nr4428	ref|NC_004461.1|:1521977-1522891
52	43	nr4429	ref|NC_004461.1|:c1523913-1523086
52	44	nr4430	ref|NC_004461.1|:c1526440-1524104
52	45	nr4431	ref|NC_004461.1|:c1526664-1526506
52	46	nr4432	ref|NC_004461.1|:c1527555-1526794
52	47	nr4433	ref|NC_004461.1|:c1528045-1527566
52	48	nr4434	ref|NC_004461.1|:c1530170-1528014
52	49	nr4435	ref|NC_004461.1|:c1531516-1530212
52	50	nr4436	ref|NC_004461.1|:c1531871-1531521

52	51	nr4437	ref|NC_004461.1|:c1532188-1531868
52	52	nr4438	ref|NC_004461.1|:c1532363-1532244
52	53	nr4439	ref|NC_004461.1|:c1533251-1532394
52	54	nr4440	ref|NC_004461.1|:c1533850-1533248
52	55	nr4441	ref|NC_004461.1|:c1534941-1533871
52	56	nr4442	ref|NC_004461.1|:c1536894-1534945
52	57	nr4443	ref|NC_004461.1|:c1537516-1536920
52	58	nr4444	ref|NC_004461.1|:c1537848-1537522
52	59	nr4445	ref|NC_004461.1|:c1538296-1537895
52	60	nr4446	ref|NC_004461.1|:c1540593-1538296
52	61	nr4447	ref|NC_004461.1|:c1543169-1540590
52	62	nr4448	ref|NC_004461.1|:c1543861-1543169
52	63	nr4449	ref|NC_004461.1|:c1546108-1543862
52	64	nr4450	ref|NC_004461.1|:c1546480-1546178
52	65	nr4451	ref|NC_004461.1|:c1546866-1546597
52	66	nr4452	ref|NC_004461.1|:c1549905-1546867
52	67	nr4453	ref|NC_004461.1|:c1551169-1549922
52	68	nr4454	ref|NC_004461.1|:1551280-1552170
52	69	nr4455	ref|NC_004461.1|:c1552470-1552210
52	70	nr4456	ref|NC_004461.1|:c1552900-1552475
52	71	nr4457	ref|NC_004461.1|:c1553321-1552872
52	72	nr4458	ref|NC_004461.1|:c1553764-1553318
52	73	nr4459	ref|NC_004461.1|:c1555578-1555042
52	74	nr4460	ref|NC_004461.1|:c1555811-1555611
52	75	nr4461	ref|NC_004461.1|:1556778-1557857
52	76	nr4462	ref|NC_004461.1|:c1584545-1584357
52	77	nr4463	ref|NC_004461.1|:c1596668-1596555
52	78	nr4464	ref|NC_004461.1|:1611509-1611649
52	79	nr4465	ref|NC_004461.1|:1670975-1671232
52	80	nr4466	ref|NC_004461.1|:c1687924-1686467
52	81	nr4467	ref|NC_004461.1|:c1695490-1695347
52	82	nr4468	ref|NC_004461.1|:c1721137-1721024
52	83	nr4469	ref|NC_004461.1|:1724495-1724620
52	84	nr4470	ref|NC_004461.1|:c1740767-1740630
52	85	nr4471	ref|NC_004461.1|:c1760738-1760388
52	86	nr4472	ref|NC_004461.1|:c1773763-1772750
53	1	nr4473	ref|NC_004461.1|:1793964-1794089
53	2	nr4474	ref|NC_004461.1|:c1810455-1810342
53	3	nr4475	ref|NC_004461.1|:1833225-1833695
53	4	nr4476	ref|NC_004461.1|:1837680-1838726
53	5	nr4477	ref|NC_004461.1|:1861830-1862252
53	6	nr4478	ref|NC_004461.1|:1883622-1883726
53	7	nr4479	ref|NC_004461.1|:1893498-1893887
53	8	nr4480	ref|NC_004461.1|:c1898622-1898524
53	9	nr4481	ref|NC_004461.1|:1909425-1910198
53	10	nr4482	ref|NC_004461.1|:c1936281-1936165
53	11	nr4483	ref|NC_004461.1|:c1937713-1936700
53	12	nr4484	ref|NC_004461.1|:c1952964-1952791
53	13	nr4485	ref|NC_004461.1|:c1953851-1953378

53	14	nr4486	ref|NC_004461.1|:c1953950-1953858
53	15	nr4487	ref|NC_004461.1|:1955903-1956070
53	16	nr4488	ref|NC_004461.1|:c1963310-1963155
53	17	nr4489	ref|NC_004461.1|:c1988946-1988683
53	18	nr4490	ref|NC_004461.1|:c1989275-1989006
53	19	nr4491	ref|NC_004461.1|:c1989756-1989301
53	20	nr4492	ref|NC_004461.1|:c1989989-1989759
53	21	nr4493	ref|NC_004461.1|:c1990534-1990130
53	22	nr4494	ref|NC_004461.1|:c2021034-2020021
53	23	nr4495	ref|NC_004461.1|:c2032933-2032547
53	24	nr4496	ref|NC_004461.1|:2042752-2042913
53	25	nr4497	ref|NC_004461.1|:c2054308-2053613
53	26	nr4498	ref|NC_004461.1|:c2077841-2077149
53	27	nr4499	ref|NC_004461.1|:2123720-2123827
53	28	nr4500	ref|NC_004461.1|:c2129942-2129838
53	29	nr4501	ref|NC_004461.1|:2143907-2144017
53	30	nr4502	ref|NC_004461.1|:c2144563-2144183
53	31	nr4503	ref|NC_004461.1|:c2180324-2180208
53	32	nr4504	ref|NC_004461.1|:c2183278-2182973
53	33	nr4505	ref|NC_004461.1|:c2196670-2196464
53	34	nr4506	ref|NC_004461.1|:c2199203-2199054
53	35	nr4507	ref|NC_004461.1|:2201426-2201521
53	36	nr4508	ref|NC_004461.1|:c2203464-2202541
53	37	nr4509	ref|NC_004461.1|:c2220345-2220238
53	38	nr4510	ref|NC_004461.1|:c2255928-2255827
53	39	nr4511	ref|NC_004461.1|:2256022-2256189
53	40	nr4512	ref|NC_004461.1|:c2259210-2256589
53	41	nr4513	ref|NC_004461.1|:2259574-2259726
53	42	nr4514	ref|NC_004461.1|:c2260432-2260094
53	43	nr4515	ref|NC_004461.1|:2277920-2278822
53	44	nr4516	ref|NC_004461.1|:c2295134-2295018
53	45	nr4517	ref|NC_004461.1|:c2296256-2296083
53	46	nr4518	ref|NC_004461.1|:c2305351-2304779
53	47	nr4519	ref|NC_004461.1|:2310176-2311771
53	48	nr4520	ref|NC_004461.1|:2311895-2314273
53	49	nr4521	ref|NC_004461.1|:c2328730-2327717
53	50	nr4522	ref|NC_004461.1|:c2333159-2332926
53	51	nr4523	ref|NC_004461.1|:c2334693-2334580
53	52	nr4524	ref|NC_004461.1|:c2379424-2379311
53	53	nr4525	ref|NC_004461.1|:2425660-2425908
53	54	nr4526	ref|NC_004461.1|:2425928-2426455
53	55	nr4527	ref|NC_004461.1|:2463809-2464651
53	56	nr4528	ref|NC_004461.1|:2465440-2465670
53	57	nr4529	ref|NC_004461.1|:c2467280-2466732
53	58	nr4530	ref|NC_005003.1|:51-245
53	59	nr4531	ref|NC_005003.1|:341-1141
53	60	nr4532	ref|NC_005003.1|:1477-1863
53	61	nr4533	ref|NC_005003.1|:1842-2042
53	62	nr4534	ref|NC_005003.1|:2029-3039

53	63	nr4535	ref|NC_005003.1|:2738-3547
53	64	nr4536	ref|NC_005003.1|:c3850-3650
53	65	nr4537	ref|NC_005003.1|:c5908-5297
53	66	nr4538	ref|NC_005003.1|:5965-6162
53	67	nr4539	ref|NC_005003.1|:c6150-5977
53	68	nr4540	ref|NC_005004.1|:c449-195
53	69	nr4541	ref|NC_005004.1|:509-2227
53	70	nr4542	ref|NC_005004.1|:4385-4708
53	71	nr4543	ref|NC_005004.1|:13637-14617
53	72	nr4544	ref|NC_005004.1|:c16232-15000
53	73	nr4545	ref|NC_005004.1|:17727-18038
53	74	nr4546	ref|NC_005004.1|:18028-18690
53	75	nr4547	ref|NC_005004.1|:18696-19334
53	76	nr4548	ref|NC_005004.1|:19440-19958
53	77	nr4549	ref|NC_005004.1|:19702-19884
53	78	nr4550	ref|NC_005004.1|:20036-20908
53	79	nr4551	ref|NC_005004.1|:21048-21482
53	80	nr4552	ref|NC_005005.1|:89-931
53	81	nr4553	ref|NC_005005.1|:3588-3827
53	82	nr4554	ref|NC_005005.1|:6296-6484
53	83	nr4555	ref|NC_005005.1|:6783-7838
53	84	nr4556	ref|NC_005005.1|:8087-8944
53	85	nr4557	ref|NC_005005.1|:c11435-10848
53	86	nr4558	ref|NC_005005.1|:11504-12955
54	1	nr4559	ref|NC_005005.1|:13765-15600
54	2	nr4560	ref|NC_005005.1|:16576-17124
54	3	nr4561	ref|NC_005006.1|:361-3657
54	4	nr4562	ref|NC_005006.1|:3767-3979
54	5	nr4563	ref|NC_005006.1|:4215-4859
54	6	nr4564	ref|NC_005006.1|:4813-5211
54	7	nr4565	ref|NC_005006.1|:5519-5968
54	8	nr4566	ref|NC_005006.1|:6121-7167
54	9	nr4567	ref|NC_005006.1|:7001-7684
54	10	nr4568	ref|NC_005006.1|:7696-8007
54	11	nr4569	ref|NC_005007.1|:598-909
54	12	nr4570	ref|NC_005007.1|:c1481-930
54	13	nr4571	ref|NC_005007.1|:2705-2926
54	14	nr4572	ref|NC_005008.1|:36-1415
54	15	nr4573	ref|NC_005951.1|:9466-10440
54	16	nr4574	ref|NC_006663.1|:c1632-988
54	17	nr4575	ref|NC_006663.1|:5952-6095
54	18	nr4576	ref|NC_006663.1|:c7412-7077
54	19	nr4577	ref|NC_006663.1|:c8482-7688
54	20	nr4578	ref|NC_006663.1|:c9117-8575
54	21	nr4579	ref|NC_006663.1|:c9953-9114
54	22	nr4580	ref|NC_006663.1|:c10080-9967
54	23	nr4581	ref|NC_006663.1|:c10789-10055
54	24	nr4582	ref|NC_006663.1|:c11639-10770
54	25	nr4583	ref|NC_006663.1|:c12659-12009

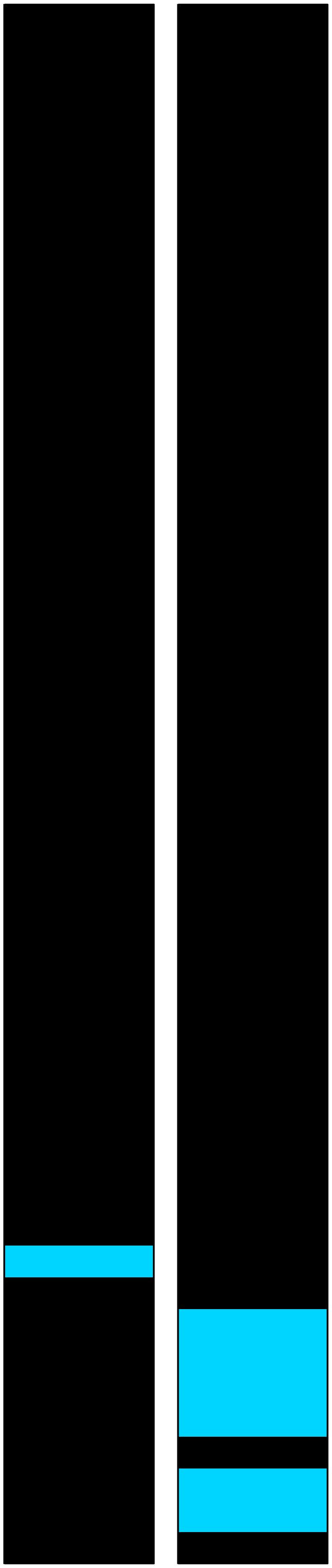

54	26	nr4584	ref|NC_006663.1|:c13799-12669
54	27	nr4585	ref|NC_006663.1|:c14505-13801
54	28	nr4586	ref|NC_006663.1|:c15649-14756
54	29	nr4587	ref|NC_006663.1|:15693-16367
54	30	nr4588	ref|NC_006663.1|:16413-17039
54	31	nr4589	ref|NC_006663.1|:c19591-19178
54	32	nr4590	ref|NC_006663.1|:c22031-21798
54	33	nr4591	ref|NC_006663.1|:22705-22905
54	34	nr4592	ref|NC_006663.1|:24376-24567
54	35	nr4593	ref|NC_007168.1|:13067-13762
54	36	nr4594	ref|NC_007168.1|:13759-14088
54	37	nr4595	ref|NC_007168.1|:25706-27037
54	38	nr4596	ref|NC_007168.1|:27853-27948
54	39	nr4597	ref|NC_007168.1|:29835-30020
54	40	nr4598	ref|NC_007168.1|:c30804-30130
54	41	nr4599	ref|NC_007168.1|:30884-31855
54	42	nr4600	ref|NC_007168.1|:c32215-32117
54	43	nr4601	ref|NC_007168.1|:c34335-33019
54	44	nr4602	ref|NC_007168.1|:42776-42922
54	45	nr4603	ref|NC_007168.1|:43499-43651
54	46	nr4604	ref|NC_007168.1|:49188-50702
54	47	nr4605	ref|NC_007168.1|:50695-52014
54	48	nr4606	ref|NC_007168.1|:c53925-52978
54	49	nr4607	ref|NC_007168.1|:c54927-54061
54	50	nr4608	ref|NC_007168.1|:c56104-54914
54	51	nr4609	ref|NC_007168.1|:56315-56458
54	52	nr4610	ref|NC_007168.1|:56624-56716
54	53	nr4611	ref|NC_007168.1|:56738-56989
54	54	nr4612	ref|NC_007168.1|:58521-58619
54	55	nr4613	ref|NC_007168.1|:58649-60637
54	56	nr4614	ref|NC_007168.1|:60832-61941
54	57	nr4615	ref|NC_007168.1|:61934-62302
54	58	nr4616	ref|NC_007168.1|:62302-63918
54	59	nr4617	ref|NC_007168.1|:64143-65819
54	60	nr4618	ref|NC_007168.1|:66170-66508
54	61	nr4619	ref|NC_007168.1|:67807-68373
54	62	nr4620	ref|NC_007168.1|:c72776-71553
54	63	nr4621	ref|NC_007168.1|:74430-74537
54	64	nr4622	ref|NC_007168.1|:74494-74562
54	65	nr4623	ref|NC_007168.1|:74710-75429
54	66	nr4624	ref|NC_007168.1|:c76322-75426
54	67	nr4625	ref|NC_007168.1|:c78646-78107
54	68	nr4626	ref|NC_007168.1|:c82213-80495
54	69	nr4627	ref|NC_007168.1|:c83300-82302
54	70	nr4628	ref|NC_007168.1|:c83965-83480
54	71	nr4629	ref|NC_007168.1|:84288-84944
54	72	nr4630	ref|NC_007168.1|:c85561-85196
54	73	nr4631	ref|NC_007168.1|:c85857-85480
54	74	nr4632	ref|NC_007168.1|:86765-86887

54	75	nr4633	ref|NC_007168.1|:c88222-86906
54	76	nr4634	ref|NC_007168.1|:88421-88990
54	77	nr4635	ref|NC_007168.1|:88987-90081
54	78	nr4636	ref|NC_007168.1|:90323-90535
54	79	nr4637	ref|NC_007168.1|:90523-90777
54	80	nr4638	ref|NC_007168.1|:c92876-91146
54	81	nr4639	ref|NC_007168.1|:c94356-92860
54	82	nr4640	ref|NC_007168.1|:100329-101003
54	83	nr4641	ref|NC_007168.1|:c101634-101287
54	84	nr4642	ref|NC_007168.1|:101712-102395
54	85	nr4643	ref|NC_007168.1|:102417-103082
54	86	nr4644	ref|NC_007168.1|:104099-104464
55	1	nr4645	ref|NC_007168.1|:105636-105977
55	2	nr4646	ref|NC_007168.1|:c106398-105997
55	3	nr4647	ref|NC_007168.1|:108332-108619
55	4	nr4648	ref|NC_007168.1|:c114208-112916
55	5	nr4649	ref|NC_007168.1|:c114522-114208
55	6	nr4650	ref|NC_007168.1|:117002-117196
55	7	nr4651	ref|NC_007168.1|:119026-119511
55	8	nr4652	ref|NC_007168.1|:c120817-120143
55	9	nr4653	ref|NC_007168.1|:123416-123940
55	10	nr4654	ref|NC_007168.1|:126321-126443
55	11	nr4655	ref|NC_007168.1|:c126726-126529
55	12	nr4656	ref|NC_007168.1|:126750-128120
55	13	nr4657	ref|NC_007168.1|:c128619-128212
55	14	nr4658	ref|NC_007168.1|:128950-129075
55	15	nr4659	ref|NC_007168.1|:129166-129243
55	16	nr4660	ref|NC_007168.1|:130997-132034
55	17	nr4661	ref|NC_007168.1|:132457-134373
55	18	nr4662	ref|NC_007168.1|:134564-135385
55	19	nr4663	ref|NC_007168.1|:135372-136076
55	20	nr4664	ref|NC_007168.1|:136643-136957
55	21	nr4665	ref|NC_007168.1|:137149-138216
55	22	nr4666	ref|NC_007168.1|:138231-140126
55	23	nr4667	ref|NC_007168.1|:146231-146923
55	24	nr4668	ref|NC_007168.1|:146920-148473
55	25	nr4669	ref|NC_007168.1|:148476-149432
55	26	nr4670	ref|NC_007168.1|:149432-150199
55	27	nr4671	ref|NC_007168.1|:150166-150933
55	28	nr4672	ref|NC_007168.1|:150926-151561
55	29	nr4673	ref|NC_007168.1|:151620-152792
55	30	nr4674	ref|NC_007168.1|:153281-154141
55	31	nr4675	ref|NC_007168.1|:c155575-154250
55	32	nr4676	ref|NC_007168.1|:155718-156149
55	33	nr4677	ref|NC_007168.1|:156168-156599
55	34	nr4678	ref|NC_007168.1|:156757-157773
55	35	nr4679	ref|NC_007168.1|:157900-158199
55	36	nr4680	ref|NC_007168.1|:158336-158860
55	37	nr4681	ref|NC_007168.1|:163504-163863

55	38	nr4682	ref|NC_007168.1|:164106-164642	[redacted]	[redacted]
55	39	nr4683	ref|NC_007168.1|:164893-165180	[redacted]	[redacted]
55	40	nr4684	ref|NC_007168.1|:c168082-167987	[redacted]	[redacted]
55	41	nr4685	ref|NC_007168.1|:168359-169222	[redacted]	[redacted]
55	42	nr4686	ref|NC_007168.1|:c170746-170699	[redacted]	[redacted]
55	43	nr4687	ref|NC_007168.1|:c171782-170919	[redacted]	[redacted]
55	44	nr4688	ref|NC_007168.1|:172024-172992	[redacted]	[redacted]
55	45	nr4689	ref|NC_007168.1|:172992-173873	[redacted]	[redacted]
55	46	nr4690	ref|NC_007168.1|:173866-174255	[redacted]	[redacted]
55	47	nr4691	ref|NC_007168.1|:174268-175764	[redacted]	[redacted]
55	48	nr4692	ref|NC_007168.1|:175745-176683	[redacted]	[redacted]
55	49	nr4693	ref|NC_007168.1|:176703-177620	[redacted]	[redacted]
55	50	nr4694	ref|NC_007168.1|:c178512-177664	[redacted]	[redacted]
55	51	nr4695	ref|NC_007168.1|:181009-181047	[redacted]	[redacted]
55	52	nr4696	ref|NC_007168.1|:181048-181911	[redacted]	[redacted]
55	53	nr4697	ref|NC_007168.1|:182806-183657	[redacted]	[redacted]
55	54	nr4698	ref|NC_007168.1|:183671-183973	[redacted]	[redacted]
55	55	nr4699	ref|NC_007168.1|:183998-185293	[redacted]	[redacted]
55	56	nr4700	ref|NC_007168.1|:185309-185626	[redacted]	[redacted]
55	57	nr4701	ref|NC_007168.1|:185633-185920	[redacted]	[redacted]
55	58	nr4702	ref|NC_007168.1|:185921-187009	[redacted]	[redacted]
55	59	nr4703	ref|NC_007168.1|:187023-188027	[redacted]	[redacted]
55	60	nr4704	ref|NC_007168.1|:188169-189035	[redacted]	[redacted]
55	61	nr4705	ref|NC_007168.1|:189171-189722	[redacted]	[redacted]
55	62	nr4706	ref|NC_007168.1|:c190993-189812	[redacted]	[redacted]
55	63	nr4707	ref|NC_007168.1|:193063-193467	[redacted]	[redacted]
55	64	nr4708	ref|NC_007168.1|:193685-194977	[redacted]	[redacted]
55	65	nr4709	ref|NC_007168.1|:195638-197032	[redacted]	[redacted]
55	66	nr4710	ref|NC_007168.1|:197059-197166	[redacted]	[redacted]
55	67	nr4711	ref|NC_007168.1|:197332-197571	[redacted]	[redacted]
55	68	nr4712	ref|NC_007168.1|:197650-197799	[redacted]	[redacted]
55	69	nr4713	ref|NC_007168.1|:197883-199583	[redacted]	[redacted]
55	70	nr4714	ref|NC_007168.1|:199619-200146	[redacted]	[redacted]
55	71	nr4715	ref|NC_007168.1|:200281-200442	[redacted]	[redacted]
55	72	nr4716	ref|NC_007168.1|:201496-203106	[redacted]	[redacted]
55	73	nr4717	ref|NC_007168.1|:203139-204419	[redacted]	[redacted]
55	74	nr4718	ref|NC_007168.1|:c207600-207202	[redacted]	[redacted]
55	75	nr4719	ref|NC_007168.1|:c209409-207751	[redacted]	[redacted]
55	76	nr4720	ref|NC_007168.1|:209570-210952	[redacted]	[redacted]
55	77	nr4721	ref|NC_007168.1|:211123-212196	[redacted]	[redacted]
55	78	nr4722	ref|NC_007168.1|:c214967-214281	[redacted]	[redacted]
55	79	nr4723	ref|NC_007168.1|:215154-215666	[redacted]	[redacted]
55	80	nr4724	ref|NC_007168.1|:215700-215795	[redacted]	[redacted]
55	81	nr4725	ref|NC_007168.1|:215949-217016	[redacted]	[redacted]
55	82	nr4726	ref|NC_007168.1|:217031-217438	[redacted]	[redacted]
55	83	nr4727	ref|NC_007168.1|:222705-224066	[redacted]	[redacted]
55	84	nr4728	ref|NC_007168.1|:224500-224838	[redacted]	[redacted]
55	85	nr4729	ref|NC_007168.1|:224879-225607	[redacted]	[redacted]
55	86	nr4730	ref|NC_007168.1|:c226401-225694	[redacted]	[redacted]

56	1	nr4731	ref|NC_007168.1|:c228636-226768	[redacted]	[redacted]
56	2	nr4732	ref|NC_007168.1|:234499-234627	[redacted]	[redacted]
56	3	nr4733	ref|NC_007168.1|:c236342-236154	[redacted]	[redacted]
56	4	nr4734	ref|NC_007168.1|:236588-239794	[redacted]	[redacted]
56	5	nr4735	ref|NC_007168.1|:240080-240685	[redacted]	[redacted]
56	6	nr4736	ref|NC_007168.1|:240914-242644	[redacted]	[redacted]
56	7	nr4737	ref|NC_007168.1|:242637-242975	[redacted]	[redacted]
56	8	nr4738	ref|NC_007168.1|:243313-245082	[redacted]	[redacted]
56	9	nr4739	ref|NC_007168.1|:245075-246715	[redacted]	[redacted]
56	10	nr4740	ref|NC_007168.1|:246817-250020	[redacted]	[redacted]
56	11	nr4741	ref|NC_007168.1|:251323-251661	[redacted]	[redacted]
56	12	nr4742	ref|NC_007168.1|:251699-252535	[redacted]	[redacted]
56	13	nr4743	ref|NC_007168.1|:252612-253022	[redacted]	[redacted]
56	14	nr4744	ref|NC_007168.1|:253015-253569	[redacted]	[redacted]
56	15	nr4745	ref|NC_007168.1|:253666-253782	[redacted]	[redacted]
56	16	nr4746	ref|NC_007168.1|:253924-254121	[redacted]	[redacted]
56	17	nr4747	ref|NC_007168.1|:254317-254508	[redacted]	[redacted]
56	18	nr4748	ref|NC_007168.1|:254538-255101	[redacted]	[redacted]
56	19	nr4749	ref|NC_007168.1|:255133-255393	[redacted]	[redacted]
56	20	nr4750	ref|NC_007168.1|:255546-255950	[redacted]	[redacted]
56	21	nr4751	ref|NC_007168.1|:c260414-260199	[redacted]	[redacted]
56	22	nr4752	ref|NC_007168.1|:c260592-260437	[redacted]	[redacted]
56	23	nr4753	ref|NC_007168.1|:c260978-260637	[redacted]	[redacted]
56	24	nr4754	ref|NC_007168.1|:c268526-268005	[redacted]	[redacted]
56	25	nr4755	ref|NC_007168.1|:c268719-268552	[redacted]	[redacted]
56	26	nr4756	ref|NC_007168.1|:270524-271723	[redacted]	[redacted]
56	27	nr4757	ref|NC_007168.1|:c272575-271790	[redacted]	[redacted]
56	28	nr4758	ref|NC_007168.1|:275978-276226	[redacted]	[redacted]
56	29	nr4759	ref|NC_007168.1|:c277023-276295	[redacted]	[redacted]
56	30	nr4760	ref|NC_007168.1|:277892-278698	[redacted]	[redacted]
56	31	nr4761	ref|NC_007168.1|:c279623-278757	[redacted]	[redacted]
56	32	nr4762	ref|NC_007168.1|:282436-283254	[redacted]	[redacted]
56	33	nr4763	ref|NC_007168.1|:283419-284027	[redacted]	[redacted]
56	34	nr4764	ref|NC_007168.1|:c284976-284158	[redacted]	[redacted]
56	35	nr4765	ref|NC_007168.1|:285132-285794	[redacted]	[redacted]
56	36	nr4766	ref|NC_007168.1|:c286499-285867	[redacted]	[redacted]
56	37	nr4767	ref|NC_007168.1|:c287259-286486	[redacted]	[redacted]
56	38	nr4768	ref|NC_007168.1|:c287993-287226	[redacted]	[redacted]
56	39	nr4769	ref|NC_007168.1|:c288942-287983	[redacted]	[redacted]
56	40	nr4770	ref|NC_007168.1|:c290448-289024	[redacted]	[redacted]
56	41	nr4771	ref|NC_007168.1|:c292231-291305	[redacted]	[redacted]
56	42	nr4772	ref|NC_007168.1|:c292928-292224	[redacted]	[redacted]
56	43	nr4773	ref|NC_007168.1|:c295682-294708	[redacted]	[redacted]
56	44	nr4774	ref|NC_007168.1|:c296937-295735	[redacted]	[redacted]
56	45	nr4775	ref|NC_007168.1|:302927-304078	[redacted]	[redacted]
56	46	nr4776	ref|NC_007168.1|:c304752-304165	[redacted]	[redacted]
56	47	nr4777	ref|NC_007168.1|:c309874-309089	[redacted]	[redacted]
56	48	nr4778	ref|NC_007168.1|:c312328-311612	[redacted]	[redacted]
56	49	nr4779	ref|NC_007168.1|:c312528-312325	[redacted]	[redacted]

56	50	nr4780	ref|NC_007168.1|:c313155-312673	[redacted]	[redacted]
56	51	nr4781	ref|NC_007168.1|:313311-314231	[redacted]	[redacted]
56	52	nr4782	ref|NC_007168.1|:c314341-314228	[redacted]	[redacted]
56	53	nr4783	ref|NC_007168.1|:c316396-315833	[redacted]	[redacted]
56	54	nr4784	ref|NC_007168.1|:322413-322790	[redacted]	[redacted]
56	55	nr4785	ref|NC_007168.1|:c324228-322912	[redacted]	[redacted]
56	56	nr4786	ref|NC_007168.1|:c325449-324415	[redacted]	[redacted]
56	57	nr4787	ref|NC_007168.1|:325667-326149	[redacted]	[redacted]
56	58	nr4788	ref|NC_007168.1|:339028-340602	[redacted]	[redacted]
56	59	nr4789	ref|NC_007168.1|:342138-342680	[redacted]	[redacted]
56	60	nr4790	ref|NC_007168.1|:c348072-347971	[redacted]	[redacted]
56	61	nr4791	ref|NC_007168.1|:c349246-348248	[redacted]	[redacted]
56	62	nr4792	ref|NC_007168.1|:c349636-349250	[redacted]	[redacted]
56	63	nr4793	ref|NC_007168.1|:c349902-349633	[redacted]	[redacted]
56	64	nr4794	ref|NC_007168.1|:c350115-349924	[redacted]	[redacted]
56	65	nr4795	ref|NC_007168.1|:350366-351538	[redacted]	[redacted]
56	66	nr4796	ref|NC_007168.1|:351665-351793	[redacted]	[redacted]
56	67	nr4797	ref|NC_007168.1|:351815-352606	[redacted]	[redacted]
56	68	nr4798	ref|NC_007168.1|:352674-353030	[redacted]	[redacted]
56	69	nr4799	ref|NC_007168.1|:353105-353581	[redacted]	[redacted]
56	70	nr4800	ref|NC_007168.1|:c355927-354611	[redacted]	[redacted]
56	71	nr4801	ref|NC_007168.1|:c357556-356501	[redacted]	[redacted]
56	72	nr4802	ref|NC_007168.1|:c364256-364089	[redacted]	[redacted]
56	73	nr4803	ref|NC_007168.1|:c365459-364587	[redacted]	[redacted]
56	74	nr4804	ref|NC_007168.1|:365611-366222	[redacted]	[redacted]
56	75	nr4805	ref|NC_007168.1|:366420-368432	[redacted]	[redacted]
56	76	nr4806	ref|NC_007168.1|:c378059-377538	[redacted]	[redacted]
56	77	nr4807	ref|NC_007168.1|:378261-378791	[redacted]	[redacted]
56	78	nr4808	ref|NC_007168.1|:c378808-378764	[redacted]	[redacted]
56	79	nr4809	ref|NC_007168.1|:380463-380927	[redacted]	[redacted]
56	80	nr4810	ref|NC_007168.1|:384154-384837	[redacted]	[redacted]
56	81	nr4811	ref|NC_007168.1|:386086-387081	[redacted]	[redacted]
56	82	nr4812	ref|NC_007168.1|:387241-388089	[redacted]	[redacted]
56	83	nr4813	ref|NC_007168.1|:c389660-389328	[redacted]	[redacted]
56	84	nr4814	ref|NC_007168.1|:393453-394769	[redacted]	[redacted]
56	85	nr4815	ref|NC_007168.1|:395009-395284	[redacted]	[redacted]
56	86	nr4816	ref|NC_007168.1|:395968-396300	[redacted]	[redacted]
57	1	nr4817	ref|NC_007168.1|:396609-398228	[redacted]	[redacted]
57	2	nr4818	ref|NC_007168.1|:c399262-398297	[redacted]	[redacted]
57	3	nr4819	ref|NC_007168.1|:c399762-399262	[redacted]	[redacted]
57	4	nr4820	ref|NC_007168.1|:c400372-399782	[redacted]	[redacted]
57	5	nr4821	ref|NC_007168.1|:401382-401483	[redacted]	[redacted]
57	6	nr4822	ref|NC_007168.1|:402683-403384	[redacted]	[redacted]
57	7	nr4823	ref|NC_007168.1|:403386-404153	[redacted]	[redacted]
57	8	nr4824	ref|NC_007168.1|:410550-411863	[redacted]	[redacted]
57	9	nr4825	ref|NC_007168.1|:411874-412887	[redacted]	[redacted]
57	10	nr4826	ref|NC_007168.1|:412906-414123	[redacted]	[redacted]
57	11	nr4827	ref|NC_007168.1|:414120-415253	[redacted]	[redacted]
57	12	nr4828	ref|NC_007168.1|:415460-416209	[redacted]	[redacted]

57	13	nr4829	ref|NC_007168.1|:418173-418343	[redacted]	[redacted]
57	14	nr4830	ref|NC_007168.1|:418529-418693	[redacted]	[redacted]
57	15	nr4831	ref|NC_007168.1|:421223-423214	[redacted]	[redacted]
57	16	nr4832	ref|NC_007168.1|:423303-424496	[redacted]	[redacted]
57	17	nr4833	ref|NC_007168.1|:425190-426386	[redacted]	[redacted]
57	18	nr4834	ref|NC_007168.1|:427676-428254	[redacted]	[redacted]
57	19	nr4835	ref|NC_007168.1|:428193-428489	[redacted]	[redacted]
57	20	nr4836	ref|NC_007168.1|:434049-434660	[redacted]	[redacted]
57	21	nr4837	ref|NC_007168.1|:439553-439666	[redacted]	[redacted]
57	22	nr4838	ref|NC_007168.1|:439690-440481	[redacted]	[redacted]
57	23	nr4839	ref|NC_007168.1|:440662-440928	[redacted]	[redacted]
57	24	nr4840	ref|NC_007168.1|:448949-451213	[redacted]	[redacted]
57	25	nr4841	ref|NC_007168.1|:454113-455294	[redacted]	[redacted]
57	26	nr4842	ref|NC_007168.1|:455411-455623	[redacted]	[redacted]
57	27	nr4843	ref|NC_007168.1|:c458365-458036	[redacted]	[redacted]
57	28	nr4844	ref|NC_007168.1|:c458805-458371	[redacted]	[redacted]
57	29	nr4845	ref|NC_007168.1|:458926-459321	[redacted]	[redacted]
57	30	nr4846	ref|NC_007168.1|:459488-459808	[redacted]	[redacted]
57	31	nr4847	ref|NC_007168.1|:465373-465507	[redacted]	[redacted]
57	32	nr4848	ref|NC_007168.1|:465687-465971	[redacted]	[redacted]
57	33	nr4849	ref|NC_007168.1|:465990-466142	[redacted]	[redacted]
57	34	nr4850	ref|NC_007168.1|:466127-466537	[redacted]	[redacted]
57	35	nr4851	ref|NC_007168.1|:466550-466867	[redacted]	[redacted]
57	36	nr4852	ref|NC_007168.1|:467042-467206	[redacted]	[redacted]
57	37	nr4853	ref|NC_007168.1|:468842-469324	[redacted]	[redacted]
57	38	nr4854	ref|NC_007168.1|:469521-470267	[redacted]	[redacted]
57	39	nr4855	ref|NC_007168.1|:470584-471909	[redacted]	[redacted]
57	40	nr4856	ref|NC_007168.1|:471952-472101	[redacted]	[redacted]
57	41	nr4857	ref|NC_007168.1|:472185-473873	[redacted]	[redacted]
57	42	nr4858	ref|NC_007168.1|:c474484-473855	[redacted]	[redacted]
57	43	nr4859	ref|NC_007168.1|:c475106-474681	[redacted]	[redacted]
57	44	nr4860	ref|NC_007168.1|:475473-476288	[redacted]	[redacted]
57	45	nr4861	ref|NC_007168.1|:482789-483676	[redacted]	[redacted]
57	46	nr4862	ref|NC_007168.1|:484965-485063	[redacted]	[redacted]
57	47	nr4863	ref|NC_007168.1|:485335-485589	[redacted]	[redacted]
57	48	nr4864	ref|NC_007168.1|:485675-485848	[redacted]	[redacted]
57	49	nr4865	ref|NC_007168.1|:485852-486355	[redacted]	[redacted]
57	50	nr4866	ref|NC_007168.1|:c487481-487188	[redacted]	[redacted]
57	51	nr4867	ref|NC_007168.1|:c490706-489759	[redacted]	[redacted]
57	52	nr4868	ref|NC_007168.1|:c491345-490710	[redacted]	[redacted]
57	53	nr4869	ref|NC_007168.1|:492990-493139	[redacted]	[redacted]
57	54	nr4870	ref|NC_007168.1|:493223-494941	[redacted]	[redacted]
57	55	nr4871	ref|NC_007168.1|:c496099-495155	[redacted]	[redacted]
57	56	nr4872	ref|NC_007168.1|:501142-501858	[redacted]	[redacted]
57	57	nr4873	ref|NC_007168.1|:502025-502540	[redacted]	[redacted]
57	58	nr4874	ref|NC_007168.1|:505183-506040	[redacted]	[redacted]
57	59	nr4875	ref|NC_007168.1|:510032-510127	[redacted]	[redacted]
57	60	nr4876	ref|NC_007168.1|:c513279-513073	[redacted]	[redacted]
57	61	nr4877	ref|NC_007168.1|:517370-518686	[redacted]	[redacted]

57	62	nr4878	ref|NC_007168.1|:520903-521853	[redacted]	[redacted]
57	63	nr4879	ref|NC_007168.1|:c522227-521910	[redacted]	[redacted]
57	64	nr4880	ref|NC_007168.1|:c524274-524161	[redacted]	[redacted]
57	65	nr4881	ref|NC_007168.1|:524389-525540	[redacted]	[redacted]
57	66	nr4882	ref|NC_007168.1|:529043-529192	[redacted]	[redacted]
57	67	nr4883	ref|NC_007168.1|:529276-531033	[redacted]	[redacted]
57	68	nr4884	ref|NC_007168.1|:531097-531546	[redacted]	[redacted]
57	69	nr4885	ref|NC_007168.1|:534215-535531	[redacted]	[redacted]
57	70	nr4886	ref|NC_007168.1|:539247-539627	[redacted]	[redacted]
57	71	nr4887	ref|NC_007168.1|:c541072-540116	[redacted]	[redacted]
57	72	nr4888	ref|NC_007168.1|:541236-542207	[redacted]	[redacted]
57	73	nr4889	ref|NC_007168.1|:c542605-542318	[redacted]	[redacted]
57	74	nr4890	ref|NC_007168.1|:542720-542887	[redacted]	[redacted]
57	75	nr4891	ref|NC_007168.1|:542914-543603	[redacted]	[redacted]
57	76	nr4892	ref|NC_007168.1|:c543921-543622	[redacted]	[redacted]
57	77	nr4893	ref|NC_007168.1|:553632-553835	[redacted]	[redacted]
57	78	nr4894	ref|NC_007168.1|:553991-554251	[redacted]	[redacted]
57	79	nr4895	ref|NC_007168.1|:c554766-554272	[redacted]	[redacted]
57	80	nr4896	ref|NC_007168.1|:560596-561504	[redacted]	[redacted]
57	81	nr4897	ref|NC_007168.1|:c563184-562903	[redacted]	[redacted]
57	82	nr4898	ref|NC_007168.1|:567458-568363	[redacted]	[redacted]
57	83	nr4899	ref|NC_007168.1|:c578722-578129	[redacted]	[redacted]
57	84	nr4900	ref|NC_007168.1|:580754-581038	[redacted]	[redacted]
57	85	nr4901	ref|NC_007168.1|:585608-586330	[redacted]	[redacted]
57	86	nr4902	ref|NC_007168.1|:586397-586456	[redacted]	[redacted]
58	1	nr4903	ref|NC_007168.1|:590120-590212	[redacted]	[redacted]
58	2	nr4904	ref|NC_007168.1|:590273-590545	[redacted]	[redacted]
58	3	nr4905	ref|NC_007168.1|:592544-594016	[redacted]	[redacted]
58	4	nr4906	ref|NC_007168.1|:594122-595360	[redacted]	[redacted]
58	5	nr4907	ref|NC_007168.1|:595460-596155	[redacted]	[redacted]
58	6	nr4908	ref|NC_007168.1|:c597114-596848	[redacted]	[redacted]
58	7	nr4909	ref|NC_007168.1|:607492-607953	[redacted]	[redacted]
58	8	nr4910	ref|NC_007168.1|:c609300-608014	[redacted]	[redacted]
58	9	nr4911	ref|NC_007168.1|:611498-612121	[redacted]	[redacted]
58	10	nr4912	ref|NC_007168.1|:612177-612977	[redacted]	[redacted]
58	11	nr4913	ref|NC_007168.1|:613154-613816	[redacted]	[redacted]
58	12	nr4914	ref|NC_007168.1|:c616124-614658	[redacted]	[redacted]
58	13	nr4915	ref|NC_007168.1|:c616794-616114	[redacted]	[redacted]
58	14	nr4916	ref|NC_007168.1|:c628957-628502	[redacted]	[redacted]
58	15	nr4917	ref|NC_007168.1|:634820-634912	[redacted]	[redacted]
58	16	nr4918	ref|NC_007168.1|:634985-635458	[redacted]	[redacted]
58	17	nr4919	ref|NC_007168.1|:c637285-636452	[redacted]	[redacted]
58	18	nr4920	ref|NC_007168.1|:c638877-637393	[redacted]	[redacted]
58	19	nr4921	ref|NC_007168.1|:645433-646122	[redacted]	[redacted]
58	20	nr4922	ref|NC_007168.1|:646944-647426	[redacted]	[redacted]
58	21	nr4923	ref|NC_007168.1|:c648296-647538	[redacted]	[redacted]
58	22	nr4924	ref|NC_007168.1|:c648982-648314	[redacted]	[redacted]
58	23	nr4925	ref|NC_007168.1|:c651417-650617	[redacted]	[redacted]
58	24	nr4926	ref|NC_007168.1|:652727-654199	[redacted]	[redacted]

58	25	nr4927	ref|NC_007168.1|:c656896-656318	[redacted]	[redacted]
58	26	nr4928	ref|NC_007168.1|:664385-664981		
58	27	nr4929	ref|NC_007168.1|:666972-667487		
58	28	nr4930	ref|NC_007168.1|:667637-668116		
58	29	nr4931	ref|NC_007168.1|:668177-668359		
58	30	nr4932	ref|NC_007168.1|:669505-670230		
58	31	nr4933	ref|NC_007168.1|:680827-681276		
58	32	nr4934	ref|NC_007168.1|:c684877-684461		
58	33	nr4935	ref|NC_007168.1|:c686244-686209		
58	34	nr4936	ref|NC_007168.1|:c690965-688860		
58	35	nr4937	ref|NC_007168.1|:c692350-691976		
58	36	nr4938	ref|NC_007168.1|:c695618-695004		
58	37	nr4939	ref|NC_007168.1|:697220-697648		
58	38	nr4940	ref|NC_007168.1|:698077-699120		
58	39	nr4941	ref|NC_007168.1|:699366-700613		
58	40	nr4942	ref|NC_007168.1|:709938-710453		
58	41	nr4943	ref|NC_007168.1|:c717914-717462		
58	42	nr4944	ref|NC_007168.1|:c718351-717911		
58	43	nr4945	ref|NC_007168.1|:c723446-722490		
58	44	nr4946	ref|NC_007168.1|:723611-724069		
58	45	nr4947	ref|NC_007168.1|:c731293-730646		
58	46	nr4948	ref|NC_007168.1|:c732969-732574		
58	47	nr4949	ref|NC_007168.1|:c733448-732972		
58	48	nr4950	ref|NC_007168.1|:738039-738236		
58	49	nr4951	ref|NC_007168.1|:738229-738978		
58	50	nr4952	ref|NC_007168.1|:c743563-742247		
58	51	nr4953	ref|NC_007168.1|:746191-746958		
58	52	nr4954	ref|NC_007168.1|:746986-747372		
58	53	nr4955	ref|NC_007168.1|:756174-756818		
58	54	nr4956	ref|NC_007168.1|:756823-757014		
58	55	nr4957	ref|NC_007168.1|:c757082-757011		
58	56	nr4958	ref|NC_007168.1|:c757310-757119		
58	57	nr4959	ref|NC_007168.1|:c757660-757310		
58	58	nr4960	ref|NC_007168.1|:c764526-764176		
58	59	nr4961	ref|NC_007168.1|:771808-772587		
58	60	nr4962	ref|NC_007168.1|:c775251-774940		
58	61	nr4963	ref|NC_007168.1|:c775582-775253		
58	62	nr4964	ref|NC_007168.1|:c777242-776814		
58	63	nr4965	ref|NC_007168.1|:c780809-780564		
58	64	nr4966	ref|NC_007168.1|:784353-785213		
58	65	nr4967	ref|NC_007168.1|:785475-786203		
58	66	nr4968	ref|NC_007168.1|:790889-791437		
58	67	nr4969	ref|NC_007168.1|:791472-792236		
58	68	nr4970	ref|NC_007168.1|:c803849-802644		
58	69	nr4971	ref|NC_007168.1|:c804283-803846		
58	70	nr4972	ref|NC_007168.1|:804462-805223		
58	71	nr4973	ref|NC_007168.1|:805290-805406		
58	72	nr4974	ref|NC_007168.1|:c810671-810354		
58	73	nr4975	ref|NC_007168.1|:c814829-813957		

58	74	nr4976	ref|NC_007168.1|:833823-835139
58	75	nr4977	ref|NC_007168.1|:840938-841693
58	76	nr4978	ref|NC_007168.1|:841740-841889
58	77	nr4979	ref|NC_007168.1|:842012-843580
58	78	nr4980	ref|NC_007168.1|:850482-850697
58	79	nr4981	ref|NC_007168.1|:c852373-851372
58	80	nr4982	ref|NC_007168.1|:c858430-857099
58	81	nr4983	ref|NC_007168.1|:c858663-858514
58	82	nr4984	ref|NC_007168.1|:861978-863738
58	83	nr4985	ref|NC_007168.1|:867086-868051
58	84	nr4986	ref|NC_007168.1|:c870028-869714
58	85	nr4987	ref|NC_007168.1|:878035-879351
58	86	nr4988	ref|NC_007168.1|:893160-893309
59	1	nr4989	ref|NC_007168.1|:893393-893884
59	2	nr4990	ref|NC_007168.1|:893881-894669
59	3	nr4991	ref|NC_007168.1|:c896186-895068
59	4	nr4992	ref|NC_007168.1|:c898399-898070
59	5	nr4993	ref|NC_007168.1|:900519-900749
59	6	nr4994	ref|NC_007168.1|:927760-928176
59	7	nr4995	ref|NC_007168.1|:930751-931884
59	8	nr4996	ref|NC_007168.1|:943575-944264
59	9	nr4997	ref|NC_007168.1|:945864-946502
59	10	nr4998	ref|NC_007168.1|:947597-948913
59	11	nr4999	ref|NC_007168.1|:952194-952343
59	12	nr5000	ref|NC_007168.1|:952427-954121
59	13	nr5001	ref|NC_007168.1|:960085-960564
59	14	nr5002	ref|NC_007168.1|:962073-962600
59	15	nr5003	ref|NC_007168.1|:976780-976929
59	16	nr5004	ref|NC_007168.1|:977013-978725
59	17	nr5005	ref|NC_007168.1|:990321-990986
59	18	nr5006	ref|NC_007168.1|:c1004455-1004318
59	19	nr5007	ref|NC_007168.1|:c1013653-1013426
59	20	nr5008	ref|NC_007168.1|:1013943-1015115
59	21	nr5009	ref|NC_007168.1|:1015868-1016569
59	22	nr5010	ref|NC_007168.1|:1016562-1017002
59	23	nr5011	ref|NC_007168.1|:c1018572-1017400
59	24	nr5012	ref|NC_007168.1|:c1019706-1018825
59	25	nr5013	ref|NC_007168.1|:1019734-1019937
59	26	nr5014	ref|NC_007168.1|:c1020989-1020825
59	27	nr5015	ref|NC_007168.1|:c1021394-1021056
59	28	nr5016	ref|NC_007168.1|:c1021489-1021391
59	29	nr5017	ref|NC_007168.1|:c1021830-1021669
59	30	nr5018	ref|NC_007168.1|:c1022290-1021889
59	31	nr5019	ref|NC_007168.1|:1023189-1023587
59	32	nr5020	ref|NC_007168.1|:1025580-1025675
59	33	nr5021	ref|NC_007168.1|:1026593-1027492
59	34	nr5022	ref|NC_007168.1|:1027489-1028172
59	35	nr5023	ref|NC_007168.1|:1030623-1031108
59	36	nr5024	ref|NC_007168.1|:1031193-1031750

59	37	nr5025	ref|NC_007168.1|:1031747-1032589	[redacted]	[redacted]
59	38	nr5026	ref|NC_007168.1|:c1054918-1053602		
59	39	nr5027	ref|NC_007168.1|:c1060454-1060362		
59	40	nr5028	ref|NC_007168.1|:1063096-1063644		
59	41	nr5029	ref|NC_007168.1|:c1069460-1068471		
59	42	nr5030	ref|NC_007168.1|:c1071346-1070489		
59	43	nr5031	ref|NC_007168.1|:c1071690-1071418		
59	44	nr5032	ref|NC_007168.1|:1075550-1076866		
59	45	nr5033	ref|NC_007168.1|:1081413-1081562		
59	46	nr5034	ref|NC_007168.1|:1081646-1082980		
59	47	nr5035	ref|NC_007168.1|:1082944-1083315		
59	48	nr5036	ref|NC_007168.1|:1085503-1085541		
59	49	nr5037	ref|NC_007168.1|:1089362-1090123		
59	50	nr5038	ref|NC_007168.1|:1090201-1091373		
59	51	nr5039	ref|NC_007168.1|:1099547-1100344		
59	52	nr5040	ref|NC_007168.1|:c1101487-1100315		
59	53	nr5041	ref|NC_007168.1|:1101764-1101829		
59	54	nr5042	ref|NC_007168.1|:1117529-1117924		
59	55	nr5043	ref|NC_007168.1|:1139619-1140179		
59	56	nr5044	ref|NC_007168.1|:1144798-1145271		
59	57	nr5045	ref|NC_007168.1|:1145252-1146022		
59	58	nr5046	ref|NC_007168.1|:1147869-1148045		
59	59	nr5047	ref|NC_007168.1|:c1151801-1151448		
59	60	nr5048	ref|NC_007168.1|:c1152163-1151798		
59	61	nr5049	ref|NC_007168.1|:c1152561-1152256		
59	62	nr5050	ref|NC_007168.1|:c1154659-1154213		
59	63	nr5051	ref|NC_007168.1|:1156881-1158197		
59	64	nr5052	ref|NC_007168.1|:1172445-1172756		
59	65	nr5053	ref|NC_007168.1|:1172983-1179453		
59	66	nr5054	ref|NC_007168.1|:1179741-1187570		
59	67	nr5055	ref|NC_007168.1|:c1197928-1197611		
59	68	nr5056	ref|NC_007168.1|:1219887-1220330		
59	69	nr5057	ref|NC_007168.1|:1220531-1221859		
59	70	nr5058	ref|NC_007168.1|:1222082-1223398		
59	71	nr5059	ref|NC_007168.1|:c1234597-1232918		
59	72	nr5060	ref|NC_007168.1|:c1234830-1234681		
59	73	nr5061	ref|NC_007168.1|:1273787-1273891		
59	74	nr5062	ref|NC_007168.1|:1280352-1280459		
59	75	nr5063	ref|NC_007168.1|:1284263-1285186		
59	76	nr5064	ref|NC_007168.1|:c1289121-1288219		
59	77	nr5065	ref|NC_007168.1|:c1289535-1289170		
59	78	nr5066	ref|NC_007168.1|:1296733-1297809		
59	79	nr5067	ref|NC_007168.1|:1302912-1303601		
59	80	nr5068	ref|NC_007168.1|:1304373-1304654		
59	81	nr5069	ref|NC_007168.1|:c1304889-1304710		
59	82	nr5070	ref|NC_007168.1|:1305058-1305534		
59	83	nr5071	ref|NC_007168.1|:1305615-1305737		
59	84	nr5072	ref|NC_007168.1|:1308373-1308483		
59	85	nr5073	ref|NC_007168.1|:1330443-1330577		

59	86	nr5074	ref|NC_007168.1|:1347353-1348357	[redacted]	[redacted]
60	1	nr5075	ref|NC_007168.1|:1362775-1362924	[redacted]	[redacted]
60	2	nr5076	ref|NC_007168.1|:1363008-1364678	[redacted]	[redacted]
60	3	nr5077	ref|NC_007168.1|:1376755-1377465	[redacted]	[redacted]
60	4	nr5078	ref|NC_007168.1|:1378483-1379172	[redacted]	[redacted]
60	5	nr5079	ref|NC_007168.1|:1381016-1381234	[redacted]	[redacted]
60	6	nr5080	ref|NC_007168.1|:1390061-1390750	[redacted]	[redacted]
60	7	nr5081	ref|NC_007168.1|:1406728-1407183	[redacted]	[redacted]
60	8	nr5082	ref|NC_007168.1|:1407164-1407463	[redacted]	[redacted]
60	9	nr5083	ref|NC_007168.1|:1407381-1407872	[redacted]	[redacted]
60	10	nr5084	ref|NC_007168.1|:c1415063-1414482	[redacted]	[redacted]
60	11	nr5085	ref|NC_007168.1|:1431301-1431921	[redacted]	[redacted]
60	12	nr5086	ref|NC_007168.1|:c1432037-1431942	[redacted]	[redacted]
60	13	nr5087	ref|NC_007168.1|:1435088-1435975	[redacted]	[redacted]
60	14	nr5088	ref|NC_007168.1|:1437808-1437903	[redacted]	[redacted]
60	15	nr5089	ref|NC_007168.1|:1443753-1443995	[redacted]	[redacted]
60	16	nr5090	ref|NC_007168.1|:c1452787-1452686	[redacted]	[redacted]
60	17	nr5091	ref|NC_007168.1|:c1465792-1465415	[redacted]	[redacted]
60	18	nr5092	ref|NC_007168.1|:c1467090-1465756	[redacted]	[redacted]
60	19	nr5093	ref|NC_007168.1|:c1467323-1467174	[redacted]	[redacted]
60	20	nr5094	ref|NC_007168.1|:1475585-1476100	[redacted]	[redacted]
60	21	nr5095	ref|NC_007168.1|:1476123-1476722	[redacted]	[redacted]
60	22	nr5096	ref|NC_007168.1|:1490660-1490998	[redacted]	[redacted]
60	23	nr5097	ref|NC_007168.1|:1513083-1516454	[redacted]	[redacted]
60	24	nr5098	ref|NC_007168.1|:1518172-1518423	[redacted]	[redacted]
60	25	nr5099	ref|NC_007168.1|:1535559-1535657	[redacted]	[redacted]
60	26	nr5100	ref|NC_007168.1|:1535755-1535967	[redacted]	[redacted]
60	27	nr5101	ref|NC_007168.1|:1536118-1537290	[redacted]	[redacted]
60	28	nr5102	ref|NC_007168.1|:1537604-1537735	[redacted]	[redacted]
60	29	nr5103	ref|NC_007168.1|:1537783-1538589	[redacted]	[redacted]
60	30	nr5104	ref|NC_007168.1|:1543208-1543357	[redacted]	[redacted]
60	31	nr5105	ref|NC_007168.1|:1543441-1545120	[redacted]	[redacted]
60	32	nr5106	ref|NC_007168.1|:c1547234-1546965	[redacted]	[redacted]
60	33	nr5107	ref|NC_007168.1|:c1549533-1548217	[redacted]	[redacted]
60	34	nr5108	ref|NC_007168.1|:c1558270-1558163	[redacted]	[redacted]
60	35	nr5109	ref|NC_007168.1|:c1570678-1569902	[redacted]	[redacted]
60	36	nr5110	ref|NC_007168.1|:c1575986-1574292	[redacted]	[redacted]
60	37	nr5111	ref|NC_007168.1|:c1576219-1576070	[redacted]	[redacted]
60	38	nr5112	ref|NC_007168.1|:1576325-1576645	[redacted]	[redacted]
60	39	nr5113	ref|NC_007168.1|:c1579205-1578579	[redacted]	[redacted]
60	40	nr5114	ref|NC_007168.1|:c1579984-1579202	[redacted]	[redacted]
60	41	nr5115	ref|NC_007168.1|:1601351-1601650	[redacted]	[redacted]
60	42	nr5116	ref|NC_007168.1|:1631826-1632017	[redacted]	[redacted]
60	43	nr5117	ref|NC_007168.1|:1632257-1632436	[redacted]	[redacted]
60	44	nr5118	ref|NC_007168.1|:c1637797-1637603	[redacted]	[redacted]
60	45	nr5119	ref|NC_007168.1|:c1639192-1639076	[redacted]	[redacted]
60	46	nr5120	ref|NC_007168.1|:c1639939-1639847	[redacted]	[redacted]
60	47	nr5121	ref|NC_007168.1|:c1640043-1639957	[redacted]	[redacted]
60	48	nr5122	ref|NC_007168.1|:c1642293-1641541	[redacted]	[redacted]

60	49	nr5123	ref|NC_007168.1|:1653840-1655012
60	50	nr5124	ref|NC_007168.1|:c1658203-1657031
60	51	nr5125	ref|NC_007168.1|:1692899-1694215
60	52	nr5126	ref|NC_007168.1|:c1722298-1721069
60	53	nr5127	ref|NC_007168.1|:c1725570-1724803
60	54	nr5128	ref|NC_007168.1|:c1745329-1744769
60	55	nr5129	ref|NC_007168.1|:c1751877-1751236
60	56	nr5130	ref|NC_007168.1|:1760367-1760570
60	57	nr5131	ref|NC_007168.1|:c1768143-1767913
60	58	nr5132	ref|NC_007168.1|:c1780557-1780078
60	59	nr5133	ref|NC_007168.1|:c1781356-1780562
60	60	nr5134	ref|NC_007168.1|:c1805132-1804998
60	61	nr5135	ref|NC_007168.1|:c1805330-1805196
60	62	nr5136	ref|NC_007168.1|:c1805521-1805387
60	63	nr5137	ref|NC_007168.1|:c1805710-1805576
60	64	nr5138	ref|NC_007168.1|:c1807135-1807061
60	65	nr5139	ref|NC_007168.1|:c1807982-1807449
60	66	nr5140	ref|NC_007168.1|:c1809608-1808214
60	67	nr5141	ref|NC_007168.1|:c1809869-1809609
60	68	nr5142	ref|NC_007168.1|:c1810318-1809920
60	69	nr5143	ref|NC_007168.1|:c1810721-1810302
60	70	nr5144	ref|NC_007168.1|:c1810907-1810758
60	71	nr5145	ref|NC_007168.1|:c1813401-1810897
60	72	nr5146	ref|NC_007168.1|:c1815226-1813415
60	73	nr5147	ref|NC_007168.1|:c1816071-1815238
60	74	nr5148	ref|NC_007168.1|:c1821674-1820766
60	75	nr5149	ref|NC_007168.1|:c1823889-1821811
60	76	nr5150	ref|NC_007168.1|:c1825027-1824212
60	77	nr5151	ref|NC_007168.1|:c1826462-1825020
60	78	nr5152	ref|NC_007168.1|:c1827027-1826434
60	79	nr5153	ref|NC_007168.1|:c1831297-1831130
60	80	nr5154	ref|NC_007168.1|:c1831952-1831311
60	81	nr5155	ref|NC_007168.1|:c1832360-1831953
60	82	nr5156	ref|NC_007168.1|:c1832779-1832357
60	83	nr5157	ref|NC_007168.1|:c1833426-1833130
60	84	nr5158	ref|NC_007168.1|:c1835551-1834685
60	85	nr5159	ref|NC_007168.1|:c1836689-1835544
60	86	nr5160	ref|NC_007168.1|:c1838370-1836709
61	1	nr5161	ref|NC_007168.1|:c1838726-1838367
61	2	nr5162	ref|NC_007168.1|:c1839264-1838938
61	3	nr5163	ref|NC_007168.1|:c1839825-1839382
61	4	nr5164	ref|NC_007168.1|:c1844433-1844296
61	5	nr5165	ref|NC_007168.1|:c1844672-1844433
61	6	nr5166	ref|NC_007168.1|:c1844887-1844678
61	7	nr5167	ref|NC_007168.1|:c1845117-1844893
61	8	nr5168	ref|NC_007168.1|:c1845335-1845117
61	9	nr5169	ref|NC_007168.1|:c1845500-1845342
61	10	nr5170	ref|NC_007168.1|:c1845851-1845537
61	11	nr5171	ref|NC_007168.1|:c1846573-1846334

61	12	nr5172	ref|NC_007168.1|:c1848330-1847662
61	13	nr5173	ref|NC_007168.1|:c1848550-1848320
61	14	nr5174	ref|NC_007168.1|:c1848794-1848519
61	15	nr5175	ref|NC_007168.1|:c1849049-1848879
61	16	nr5176	ref|NC_007168.1|:1849429-1849605
61	17	nr5177	ref|NC_007168.1|:c1850306-1849767
61	18	nr5178	ref|NC_007168.1|:c1851225-1850299
61	19	nr5179	ref|NC_007168.1|:1851272-1851511
61	20	nr5180	ref|NC_007168.1|:c1851650-1851456
61	21	nr5181	ref|NC_007168.1|:c1852479-1851688
61	22	nr5182	ref|NC_007168.1|:c1852673-1852479
61	23	nr5183	ref|NC_007168.1|:1852953-1853291
61	24	nr5184	ref|NC_007168.1|:1853305-1853976
61	25	nr5185	ref|NC_007168.1|:c1869047-1868526
61	26	nr5186	ref|NC_007168.1|:1878650-1879039
61	27	nr5187	ref|NC_007168.1|:c1879293-1879042
61	28	nr5188	ref|NC_007168.1|:1879463-1880392
61	29	nr5189	ref|NC_007168.1|:c1897514-1897029
61	30	nr5190	ref|NC_007168.1|:c1903706-1903434
61	31	nr5191	ref|NC_007168.1|:c1909698-1909075
61	32	nr5192	ref|NC_007168.1|:1912832-1912981
61	33	nr5193	ref|NC_007168.1|:1913078-1913302
61	34	nr5194	ref|NC_007168.1|:c1913666-1913280
61	35	nr5195	ref|NC_007168.1|:1917528-1917677
61	36	nr5196	ref|NC_007168.1|:1917761-1919095
61	37	nr5197	ref|NC_007168.1|:1919059-1919463
61	38	nr5198	ref|NC_007168.1|:1925168-1925287
61	39	nr5199	ref|NC_007168.1|:c1941184-1941080
61	40	nr5200	ref|NC_007168.1|:c1941590-1941270
61	41	nr5201	ref|NC_007168.1|:1941766-1941915
61	42	nr5202	ref|NC_007168.1|:1941999-1943699
61	43	nr5203	ref|NC_007168.1|:1950920-1952092
61	44	nr5204	ref|NC_007168.1|:1955688-1956110
61	45	nr5205	ref|NC_007168.1|:1960673-1961989
61	46	nr5206	ref|NC_007168.1|:c1963363-1962836
61	47	nr5207	ref|NC_007168.1|:c1972318-1971764
61	48	nr5208	ref|NC_007168.1|:1972505-1972735
61	49	nr5209	ref|NC_007168.1|:1972814-1972915
61	50	nr5210	ref|NC_007168.1|:1974537-1975853
61	51	nr5211	ref|NC_007168.1|:1976228-1976338
61	52	nr5212	ref|NC_007168.1|:1976352-1976825
61	53	nr5213	ref|NC_007168.1|:1976896-1977471
61	54	nr5214	ref|NC_007168.1|:c1985881-1985816
61	55	nr5215	ref|NC_007168.1|:c1987843-1987589
61	56	nr5216	ref|NC_007168.1|:2011741-2011890
61	57	nr5217	ref|NC_007168.1|:2011974-2013581
61	58	nr5218	ref|NC_007168.1|:2013681-2014997
61	59	nr5219	ref|NC_007168.1|:2023961-2024155
61	60	nr5220	ref|NC_007168.1|:c2032838-2032437

61	61	nr5221	ref|NC_007168.1|:c2043302-2042730	[redacted]	[redacted]
61	62	nr5222	ref|NC_007168.1|:c2053956-2053777		
61	63	nr5223	ref|NC_007168.1|:2070475-2070624		
61	64	nr5224	ref|NC_007168.1|:c2086934-2085762		
61	65	nr5225	ref|NC_007168.1|:c2087827-2087147		
61	66	nr5226	ref|NC_007168.1|:c2096575-2094893		
61	67	nr5227	ref|NC_007168.1|:c2096808-2096659		
61	68	nr5228	ref|NC_007168.1|:2097226-2097390		
61	69	nr5229	ref|NC_007168.1|:c2100154-2100011		
61	70	nr5230	ref|NC_007168.1|:c2103922-2103197		
61	71	nr5231	ref|NC_007168.1|:2104770-2105261		
61	72	nr5232	ref|NC_007168.1|:2107033-2107275		
61	73	nr5233	ref|NC_007168.1|:2108021-2108278		
61	74	nr5234	ref|NC_007168.1|:2109642-2109824		
61	75	nr5235	ref|NC_007168.1|:2109839-2109943		
61	76	nr5236	ref|NC_007168.1|:2110057-2110389		
61	77	nr5237	ref|NC_007168.1|:c2111066-2110458		
61	78	nr5238	ref|NC_007168.1|:2112558-2112782		
61	79	nr5239	ref|NC_007168.1|:2112910-2113641		
61	80	nr5240	ref|NC_007168.1|:2113840-2114016		
61	81	nr5241	ref|NC_007168.1|:c2115393-2114077		
61	82	nr5242	ref|NC_007168.1|:c2115715-2115602		
61	83	nr5243	ref|NC_007168.1|:c2117340-2116324		
61	84	nr5244	ref|NC_007168.1|:c2118271-2117855		
61	85	nr5245	ref|NC_007168.1|:c2118940-2118482		
61	86	nr5246	ref|NC_007168.1|:c2120118-2119180		
62	1	nr5247	ref|NC_007168.1|:c2120772-2120119		
62	2	nr5248	ref|NC_007168.1|:c2121211-2120732		
62	3	nr5249	ref|NC_007168.1|:c2121484-2121362		
62	4	nr5250	ref|NC_007168.1|:c2121645-2121550		
62	5	nr5251	ref|NC_007168.1|:2121988-2122143		
62	6	nr5252	ref|NC_007168.1|:c2123502-2122930		
62	7	nr5253	ref|NC_007168.1|:c2123842-2123513		
62	8	nr5254	ref|NC_007168.1|:c2124276-2123839		
62	9	nr5255	ref|NC_007168.1|:c2124540-2124331		
62	10	nr5256	ref|NC_007168.1|:c2125130-2124540		
62	11	nr5257	ref|NC_007168.1|:c2125540-2125208		
62	12	nr5258	ref|NC_007168.1|:c2125820-2125707		
62	13	nr5259	ref|NC_007168.1|:c2128568-2127201		
62	14	nr5260	ref|NC_007168.1|:c2129449-2128580		
62	15	nr5261	ref|NC_007168.1|:c2129843-2129538		
62	16	nr5262	ref|NC_007168.1|:c2130061-2129858		
62	17	nr5263	ref|NC_007168.1|:c2130328-2130074		
62	18	nr5264	ref|NC_007168.1|:c2130533-2130330		
62	19	nr5265	ref|NC_007168.1|:c2130737-2130537		
62	20	nr5266	ref|NC_007168.1|:2131156-2132073		
62	21	nr5267	ref|NC_007168.1|:2133165-2133881		
62	22	nr5268	ref|NC_007168.1|:2147092-2147301		
62	23	nr5269	ref|NC_007168.1|:2148307-2148486		

62	24	nr5270	ref|NC_007168.1|:2148609-2148773
62	25	nr5271	ref|NC_007168.1|:2149684-2149899
62	26	nr5272	ref|NC_007168.1|:c2184618-2183743
62	27	nr5273	ref|NC_007168.1|:2186286-2186603
62	28	nr5274	ref|NC_007168.1|:c2187602-2186661
62	29	nr5275	ref|NC_007168.1|:c2187699-2187538
62	30	nr5276	ref|NC_007168.1|:c2204797-2203283
62	31	nr5277	ref|NC_007168.1|:2212395-2212544
62	32	nr5278	ref|NC_007168.1|:2212628-2212885
62	33	nr5279	ref|NC_007168.1|:2213116-2214378
62	34	nr5280	ref|NC_007168.1|:c2218714-2218508
62	35	nr5281	ref|NC_007168.1|:2223379-2223990
62	36	nr5282	ref|NC_007168.1|:2225319-2226635
62	37	nr5283	ref|NC_007168.1|:2236430-2236849
62	38	nr5284	ref|NC_007168.1|:2237695-2237973
62	39	nr5285	ref|NC_007168.1|:c2241844-2241548
62	40	nr5286	ref|NC_007168.1|:c2242847-2241918
62	41	nr5287	ref|NC_007168.1|:2248339-2249013
62	42	nr5288	ref|NC_007168.1|:c2251747-2251259
62	43	nr5289	ref|NC_007168.1|:c2252262-2251813
62	44	nr5290	ref|NC_007168.1|:2256810-2257913
62	45	nr5291	ref|NC_007168.1|:c2262175-2260028
62	46	nr5292	ref|NC_007168.1|:c2272128-2271205
62	47	nr5293	ref|NC_007168.1|:2274062-2274160
62	48	nr5294	ref|NC_007168.1|:c2287641-2286532
62	49	nr5295	ref|NC_007168.1|:c2288030-2287638
62	50	nr5296	ref|NC_007168.1|:2301106-2301213
62	51	nr5297	ref|NC_007168.1|:2302779-2303018
62	52	nr5298	ref|NC_007168.1|:c2304343-2303171
62	53	nr5299	ref|NC_007168.1|:2304655-2305044
62	54	nr5300	ref|NC_007168.1|:c2311613-2311056
62	55	nr5301	ref|NC_007168.1|:2311756-2311962
62	56	nr5302	ref|NC_007168.1|:2311997-2312218
62	57	nr5303	ref|NC_007168.1|:2312520-2313446
62	58	nr5304	ref|NC_007168.1|:c2316758-2315442
62	59	nr5305	ref|NC_007168.1|:c2317769-2316960
62	60	nr5306	ref|NC_007168.1|:c2324045-2323548
62	61	nr5307	ref|NC_007168.1|:c2324875-2324111
62	62	nr5308	ref|NC_007168.1|:2328060-2328155
62	63	nr5309	ref|NC_007168.1|:c2329389-2329228
62	64	nr5310	ref|NC_007168.1|:2330028-2330246
62	65	nr5311	ref|NC_007168.1|:2330449-2330607
62	66	nr5312	ref|NC_007168.1|:2330705-2330863
62	67	nr5313	ref|NC_007168.1|:2331511-2331606
62	68	nr5314	ref|NC_007168.1|:2333752-2335218
62	69	nr5315	ref|NC_007168.1|:2335317-2336216
62	70	nr5316	ref|NC_007168.1|:c2337363-2336827
62	71	nr5317	ref|NC_007168.1|:c2337958-2337377
62	72	nr5318	ref|NC_007168.1|:c2340402-2339617

62	73	nr5319	ref|NC_007168.1|:2340513-2340623
62	74	nr5320	ref|NC_007168.1|:2340645-2340851
62	75	nr5321	ref|NC_007168.1|:2340856-2341041
62	76	nr5322	ref|NC_007168.1|:c2341293-2341168
62	77	nr5323	ref|NC_007168.1|:c2341463-2341317
62	78	nr5324	ref|NC_007168.1|:c2341911-2341621
62	79	nr5325	ref|NC_007168.1|:c2342207-2341908
62	80	nr5326	ref|NC_007168.1|:2342372-2342488
62	81	nr5327	ref|NC_007168.1|:c2342702-2342625
62	82	nr5328	ref|NC_007168.1|:2343168-2344337
62	83	nr5329	ref|NC_007168.1|:2344921-2345109
62	84	nr5330	ref|NC_007168.1|:2345340-2345957
62	85	nr5331	ref|NC_007168.1|:c2347160-2346486
62	86	nr5332	ref|NC_007168.1|:2352169-2352312
63	1	nr5333	ref|NC_007168.1|:c2355097-2354612
63	2	nr5334	ref|NC_007168.1|:c2359969-2359574
63	3	nr5335	ref|NC_007168.1|:c2360408-2359953
63	4	nr5336	ref|NC_007168.1|:c2360563-2360423
63	5	nr5337	ref|NC_007168.1|:c2360891-2360547
63	6	nr5338	ref|NC_007168.1|:c2362401-2360902
63	7	nr5339	ref|NC_007168.1|:c2365067-2362401
63	8	nr5340	ref|NC_007168.1|:c2367947-2366931
63	9	nr5341	ref|NC_007168.1|:c2371098-2367886
63	10	nr5342	ref|NC_007168.1|:c2371411-2371103
63	11	nr5343	ref|NC_007168.1|:c2371944-2371441
63	12	nr5344	ref|NC_007168.1|:c2372548-2372009
63	13	nr5345	ref|NC_007168.1|:c2372973-2372551
63	14	nr5346	ref|NC_007168.1|:c2373402-2372989
63	15	nr5347	ref|NC_007168.1|:c2373724-2373395
63	16	nr5348	ref|NC_007168.1|:c2374031-2373717
63	17	nr5349	ref|NC_007168.1|:c2374324-2374031
63	18	nr5350	ref|NC_007168.1|:c2375159-2374338
63	19	nr5351	ref|NC_007168.1|:c2375771-2375181
63	20	nr5352	ref|NC_007168.1|:c2376825-2375869
63	21	nr5353	ref|NC_007168.1|:c2378113-2376770
63	22	nr5354	ref|NC_007168.1|:c2379472-2378207
63	23	nr5355	ref|NC_007168.1|:c2379836-2379456
63	24	nr5356	ref|NC_007168.1|:2379938-2380051
63	25	nr5357	ref|NC_007168.1|:c2381016-2380564
63	26	nr5358	ref|NC_007168.1|:c2381197-2381018
63	27	nr5359	ref|NC_007168.1|:c2381357-2381199
63	28	nr5360	ref|NC_007168.1|:c2381562-2381350
63	29	nr5361	ref|NC_007168.1|:c2381778-2381575
63	30	nr5362	ref|NC_007168.1|:c2382509-2382087
63	31	nr5363	ref|NC_007168.1|:c2382797-2382510
63	32	nr5364	ref|NC_007168.1|:c2383182-2382787
63	33	nr5365	ref|NC_007168.1|:c2384203-2383748
63	34	nr5366	ref|NC_007168.1|:c2384768-2384208
63	35	nr5367	ref|NC_007168.1|:c2384967-2384773

63	36	nr5368	ref|NC_007168.1|:c2386531-2385761	[redacted]	[redacted]
63	37	nr5369	ref|NC_007168.1|:c2387320-2386544		
63	38	nr5370	ref|NC_007168.1|:c2388071-2387313		
63	39	nr5371	ref|NC_007168.1|:c2388307-2388077		
63	40	nr5372	ref|NC_007168.1|:c2388971-2388282		
63	41	nr5373	ref|NC_007168.1|:c2389528-2388983		
63	42	nr5374	ref|NC_007168.1|:c2390345-2389560		
63	43	nr5375	ref|NC_007168.1|:c2390595-2390338		
63	44	nr5376	ref|NC_007168.1|:c2390818-2390570		
63	45	nr5377	ref|NC_007168.1|:c2391163-2391038		
63	46	nr5378	ref|NC_007168.1|:2391222-2391434		
63	47	nr5379	ref|NC_007168.1|:c2392145-2391606		
63	48	nr5380	ref|NC_007168.1|:2393114-2393320		
63	49	nr5381	ref|NC_007168.1|:c2393726-2393487		
63	50	nr5382	ref|NC_007168.1|:2393915-2394589		
63	51	nr5383	ref|NC_007168.1|:2394607-2394744		
63	52	nr5384	ref|NC_007168.1|:2394908-2396293		
63	53	nr5385	ref|NC_007168.1|:c2413880-2413620		
63	54	nr5386	ref|NC_007168.1|:c2414250-2413870		
63	55	nr5387	ref|NC_007168.1|:c2422715-2420655		
63	56	nr5388	ref|NC_007168.1|:2430308-2430457		
63	57	nr5389	ref|NC_007168.1|:2430541-2431977		
63	58	nr5390	ref|NC_007168.1|:c2432828-2432229		
63	59	nr5391	ref|NC_007168.1|:c2433383-2432877		
63	60	nr5392	ref|NC_007168.1|:c2434481-2433888		
63	61	nr5393	ref|NC_007168.1|:c2439405-2438986		
63	62	nr5394	ref|NC_007168.1|:c2439704-2439627		
63	63	nr5395	ref|NC_007168.1|:c2441061-2440765		
63	64	nr5396	ref|NC_007168.1|:c2441475-2441341		
63	65	nr5397	ref|NC_007168.1|:c2442108-2441659		
63	66	nr5398	ref|NC_007168.1|:c2443889-2443617		
63	67	nr5399	ref|NC_007168.1|:c2444166-2443909		
63	68	nr5400	ref|NC_007168.1|:c2444985-2444338		
63	69	nr5401	ref|NC_007168.1|:c2445652-2444939		
63	70	nr5402	ref|NC_007168.1|:2448293-2448388		
63	71	nr5403	ref|NC_007168.1|:2452696-2452794		
63	72	nr5404	ref|NC_007168.1|:c2453363-2452944		
63	73	nr5405	ref|NC_007168.1|:c2453881-2453375		
63	74	nr5406	ref|NC_007168.1|:2471746-2472459		
63	75	nr5407	ref|NC_007168.1|:2472536-2473060		
63	76	nr5408	ref|NC_007168.1|:c2476414-2475824		
63	77	nr5409	ref|NC_007168.1|:c2494559-2494203		
63	78	nr5410	ref|NC_007168.1|:c2507468-2507400		
63	79	nr5411	ref|NC_007168.1|:c2513000-2512605		
63	80	nr5412	ref|NC_007168.1|:c2532665-2532417		
63	81	nr5413	ref|NC_007168.1|:c2538273-2536933		
63	82	nr5414	ref|NC_007168.1|:c2547920-2547393		
63	83	nr5415	ref|NC_007168.1|:c2549311-2548586		
63	84	nr5416	ref|NC_007168.1|:c2562603-2562139		

63	85	nr5417	ref|NC_007168.1|:2563129-2563407
63	86	nr5418	ref|NC_007168.1|:c2566943-2566047
64	1	nr5419	ref|NC_007168.1|:2567217-2567747
64	2	nr5420	ref|NC_007168.1|:2567868-2568407
64	3	nr5421	ref|NC_007168.1|:2569939-2570226
64	4	nr5422	ref|NC_007168.1|:c2571529-2570798
64	5	nr5423	ref|NC_007168.1|:c2572453-2571596
64	6	nr5424	ref|NC_007168.1|:c2573172-2572486
64	7	nr5425	ref|NC_007168.1|:c2573924-2573232
64	8	nr5426	ref|NC_007168.1|:c2579669-2579598
64	9	nr5427	ref|NC_007168.1|:2579776-2579955
64	10	nr5428	ref|NC_007168.1|:c2580848-2580333
64	11	nr5429	ref|NC_007168.1|:2581338-2581445
64	12	nr5430	ref|NC_007168.1|:c2581931-2581827
64	13	nr5431	ref|NC_007168.1|:c2584127-2583858
64	14	nr5432	ref|NC_007168.1|:c2585045-2584707
64	15	nr5433	ref|NC_007168.1|:c2585491-2585042
64	16	nr5434	ref|NC_007168.1|:c2585581-2585546
64	17	nr5435	ref|NC_007168.1|:2586066-2587238
64	18	nr5436	ref|NC_007168.1|:2587296-2587892
64	19	nr5437	ref|NC_007168.1|:c2589048-2587876
64	20	nr5438	ref|NC_007168.1|:2589111-2589563
64	21	nr5439	ref|NC_007168.1|:2591133-2591300
64	22	nr5440	ref|NC_007168.1|:2591350-2591544
64	23	nr5441	ref|NC_007168.1|:2591851-2592027
64	24	nr5442	ref|NC_007168.1|:2611334-2611483
64	25	nr5443	ref|NC_007168.1|:c2611951-2611805
64	26	nr5444	ref|NC_007168.1|:2612087-2612617
64	27	nr5445	ref|NC_007168.1|:2612857-2613240
64	28	nr5446	ref|NC_007168.1|:2613379-2613639
64	29	nr5447	ref|NC_007168.1|:2613807-2613953
64	30	nr5448	ref|NC_007168.1|:2614148-2614243
64	31	nr5449	ref|NC_007168.1|:2614437-2615096
64	32	nr5450	ref|NC_007168.1|:2615342-2615944
64	33	nr5451	ref|NC_007168.1|:2619289-2619690
64	34	nr5452	ref|NC_007168.1|:2619704-2620240
64	35	nr5453	ref|NC_007168.1|:c2621159-2620599
64	36	nr5454	ref|NC_007168.1|:2623114-2623257
64	37	nr5455	ref|NC_007168.1|:c2625030-2624620
64	38	nr5456	ref|NC_007168.1|:c2626328-2624994
64	39	nr5457	ref|NC_007168.1|:2626662-2627375
64	40	nr5458	ref|NC_007168.1|:2627452-2627976
64	41	nr5459	ref|NC_007168.1|:c2629280-2629071
64	42	nr5460	ref|NC_007168.1|:2629956-2631026
64	43	nr5461	ref|NC_007168.1|:2633528-2633719
64	44	nr5462	ref|NC_007168.1|:c2646005-2645232
64	45	nr5463	ref|NC_007168.1|:2646213-2646341
64	46	nr5464	ref|NC_007168.1|:2646378-2647997
64	47	nr5465	ref|NC_007168.1|:2648708-2649097

64	48	nr5466	ref|NC_007168.1|:c2650965-2649160	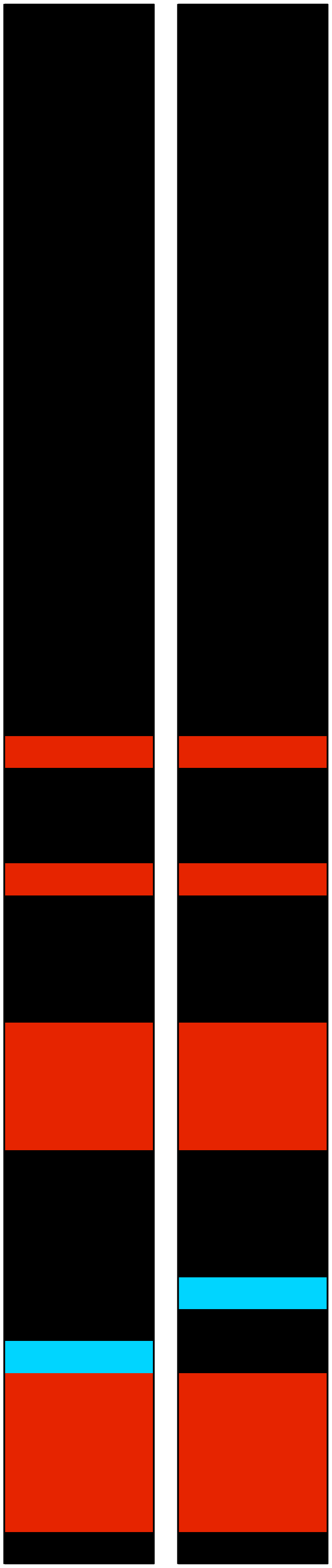
64	49	nr5467	ref|NC_007168.1|:c2651825-2650983	
64	50	nr5468	ref|NC_007168.1|:c2652890-2651844	
64	51	nr5469	ref|NC_007168.1|:c2654306-2652903	
64	52	nr5470	ref|NC_007168.1|:c2655725-2654322	
64	53	nr5471	ref|NC_007168.1|:c2656751-2655741	
64	54	nr5472	ref|NC_007168.1|:c2657394-2656756	
64	55	nr5473	ref|NC_007168.1|:c2659008-2657791	
64	56	nr5474	ref|NC_007168.1|:2659055-2659891	
64	57	nr5475	ref|NC_007168.1|:2659910-2661634	
64	58	nr5476	ref|NC_007168.1|:2661998-2663071	
64	59	nr5477	ref|NC_007168.1|:2663058-2663645	
64	60	nr5478	ref|NC_007168.1|:2663652-2664773	
64	61	nr5479	ref|NC_007168.1|:c2665772-2665710	
64	62	nr5480	ref|NC_007168.1|:c2669097-2668525	
64	63	nr5481	ref|NC_007168.1|:2669450-2669857	
64	64	nr5482	ref|NC_007168.1|:2672540-2672734	
64	65	nr5483	ref|NC_007168.1|:2673058-2676720	
64	66	nr5484	ref|NC_007168.1|:2677407-2677550	
64	67	nr5485	ref|NC_007168.1|:c2678501-2678103	
64	68	nr5486	ref|NC_007168.1|:c2678825-2678520	
64	69	nr5487	ref|NC_007622.1|:34735-35634	
64	70	nr5488	ref|NC_007622.1|:36930-37709	
64	71	nr5489	ref|NC_007622.1|:c38373-37894	
64	72	nr5490	ref|NC_007622.1|:46608-47339	
64	73	nr5491	ref|NC_007622.1|:c98985-98761	
64	74	nr5492	ref|NC_007622.1|:c99547-99008	
64	75	nr5493	ref|NC_007622.1|:255881-256174	
64	76	nr5494	ref|NC_007622.1|:c313915-313262	
64	77	nr5495	ref|NC_007622.1|:320847-321056	
64	78	nr5496	ref|NC_007622.1|:321668-322255	
64	79	nr5497	ref|NC_007622.1|:322252-322707	
64	80	nr5498	ref|NC_007622.1|:c334237-334016	
64	81	nr5499	ref|NC_007622.1|:342058-342351	
64	82	nr5500	ref|NC_007622.1|:361898-362236	
64	83	nr5501	ref|NC_007622.1|:c380692-380315	
64	84	nr5502	ref|NC_007622.1|:c389976-388840	
64	85	nr5503	ref|NC_007622.1|:c390845-390060	
64	86	nr5504	ref|NC_007622.1|:390962-391189	
65	1	nr5505	ref|NC_007622.1|:391235-391495	
65	2	nr5506	ref|NC_007622.1|:392271-392588	
65	3	nr5507	ref|NC_007622.1|:393535-395244	
65	4	nr5508	ref|NC_007622.1|:395575-395955	
65	5	nr5509	ref|NC_007622.1|:395952-396593	
65	6	nr5510	ref|NC_007622.1|:396940-397281	
65	7	nr5511	ref|NC_007622.1|:397889-398107	
65	8	nr5512	ref|NC_007622.1|:398110-398685	
65	9	nr5513	ref|NC_007622.1|:398688-399029	
65	10	nr5514	ref|NC_007622.1|:408432-408599	

65	11	nr5515	ref|NC_007622.1|:c411581-411321
65	12	nr5516	ref|NC_007622.1|:420356-421912
65	13	nr5517	ref|NC_007622.1|:427791-428153
65	14	nr5518	ref|NC_007622.1|:429813-430607
65	15	nr5519	ref|NC_007622.1|:c745696-745469
65	16	nr5520	ref|NC_007622.1|:797835-797993
65	17	nr5521	ref|NC_007622.1|:812840-813385
65	18	nr5522	ref|NC_007622.1|:813995-814930
65	19	nr5523	ref|NC_007622.1|:850702-851628
65	20	nr5524	ref|NC_007622.1|:851630-852598
65	21	nr5525	ref|NC_007622.1|:c892103-891975
65	22	nr5526	ref|NC_007622.1|:898015-898200
65	23	nr5527	ref|NC_007622.1|:c923943-922627
65	24	nr5528	ref|NC_007622.1|:930624-931559
65	25	nr5529	ref|NC_007622.1|:931549-932568
65	26	nr5530	ref|NC_007622.1|:932654-933664
65	27	nr5531	ref|NC_007622.1|:933654-934574
65	28	nr5532	ref|NC_007622.1|:934603-935457
65	29	nr5533	ref|NC_007622.1|:c980364-979690
65	30	nr5534	ref|NC_007622.1|:981279-981593
65	31	nr5535	ref|NC_007622.1|:c992407-992243
65	32	nr5536	ref|NC_007622.1|:c1027539-1027336
65	33	nr5537	ref|NC_007622.1|:c1105918-1105724
65	34	nr5538	ref|NC_007622.1|:1119062-1119274
65	35	nr5539	ref|NC_007622.1|:c1269489-1268707
65	36	nr5540	ref|NC_007622.1|:c1270028-1269528
65	37	nr5541	ref|NC_007622.1|:c1280025-1279804
65	38	nr5542	ref|NC_007622.1|:c1353443-1353231
65	39	nr5543	ref|NC_007622.1|:c1373231-1372614
65	40	nr5544	ref|NC_007622.1|:c1410803-1408971
65	41	nr5545	ref|NC_007622.1|:c1416185-1415268
65	42	nr5546	ref|NC_007622.1|:c1416749-1416357
65	43	nr5547	ref|NC_007622.1|:c1423005-1422064
65	44	nr5548	ref|NC_007622.1|:c1468107-1467916
65	45	nr5549	ref|NC_007622.1|:c1480282-1479068
65	46	nr5550	ref|NC_007622.1|:c1498818-1498609
65	47	nr5551	ref|NC_007622.1|:c1499903-1499295
65	48	nr5552	ref|NC_007622.1|:c1501203-1499878
65	49	nr5553	ref|NC_007622.1|:c1501753-1501190
65	50	nr5554	ref|NC_007622.1|:c1502119-1501772
65	51	nr5555	ref|NC_007622.1|:c1502435-1502127
65	52	nr5556	ref|NC_007622.1|:c1503007-1502495
65	53	nr5557	ref|NC_007622.1|:c1503299-1503162
65	54	nr5558	ref|NC_007622.1|:c1504042-1503308
65	55	nr5559	ref|NC_007622.1|:c1504890-1504039
65	56	nr5560	ref|NC_007622.1|:c1505114-1504962
65	57	nr5561	ref|NC_007622.1|:1505329-1505463
65	58	nr5562	ref|NC_007622.1|:c1573855-1573604
65	59	nr5563	ref|NC_007622.1|:c1722850-1721534

65	60	nr5564	ref|NC_007622.1|:c1725816-1725040		
65	61	nr5565	ref|NC_007622.1|:c1802282-1802088		
65	62	nr5566	ref|NC_007622.1|:c1802530-1802279		
65	63	nr5567	ref|NC_007622.1|:1829951-1830142		
65	64	nr5568	ref|NC_007622.1|:1833270-1833383		
65	65	nr5569	ref|NC_007622.1|:c1833870-1833727		
65	66	nr5570	ref|NC_007622.1|:1838050-1838211		
65	67	nr5571	ref|NC_007622.1|:c1851215-1850910		
65	68	nr5572	ref|NC_007622.1|:c1870817-1869528		
65	69	nr5573	ref|NC_007622.1|:c1871329-1870820		
65	70	nr5574	ref|NC_007622.1|:c1872701-1872456		
65	71	nr5575	ref|NC_007622.1|:c1881136-1880822		
65	72	nr5576	ref|NC_007622.1|:c1881541-1881326		
65	73	nr5577	ref|NC_007622.1|:c1884153-1883938		
65	74	nr5578	ref|NC_007622.1|:1884309-1884977		
65	75	nr5579	ref|NC_007622.1|:1885034-1885534		
65	76	nr5580	ref|NC_007622.1|:1885538-1886053		
65	77	nr5581	ref|NC_007622.1|:1886112-1887176		
65	78	nr5582	ref|NC_007622.1|:c1887465-1887079		
65	79	nr5583	ref|NC_007622.1|:1897321-1897470		
65	80	nr5584	ref|NC_007622.1|:c1965647-1965417		
65	81	nr5585	ref|NC_007622.1|:c1971711-1971358		
65	82	nr5586	ref|NC_007622.1|:1971836-1972795		
65	83	nr5587	ref|NC_007622.1|:1981141-1981440		
65	84	nr5588	ref|NC_007622.1|:c2016450-2016112		
65	85	nr5589	ref|NC_007622.1|:c2016982-2016572		
65	86	nr5590	ref|NC_007622.1|:c2018055-2016985		
66	1	nr5591	ref|NC_007622.1|:c2018689-2018495		
66	2	nr5592	ref|NC_007622.1|:c2023228-2023094		
66	3	nr5593	ref|NC_007622.1|:c2024429-2023929		
66	4	nr5594	ref|NC_007622.1|:2024780-2024896		
66	5	nr5595	ref|NC_007622.1|:2025043-2025726		
66	6	nr5596	ref|NC_007622.1|:c2027544-2027398		
66	7	nr5597	ref|NC_007622.1|:c2029882-2029304		
66	8	nr5598	ref|NC_007622.1|:c2030541-2030416		
66	9	nr5599	ref|NC_007622.1|:c2030726-2030553		
66	10	nr5600	ref|NC_007622.1|:c2034671-2033802		
66	11	nr5601	ref|NC_007622.1|:c2035424-2035056		
66	12	nr5602	ref|NC_007622.1|:c2036274-2036086		
66	13	nr5603	ref|NC_007622.1|:2036444-2037166		
66	14	nr5604	ref|NC_007622.1|:2043598-2043795		
66	15	nr5605	ref|NC_007622.1|:2060506-2060703		
66	16	nr5606	ref|NC_007622.1|:c2185453-2184629		
66	17	nr5607	ref|NC_007622.1|:c2185950-2185450		
66	18	nr5608	ref|NC_007622.1|:c2233795-2232866		
66	19	nr5609	ref|NC_007622.1|:2233832-2234521		
66	20	nr5610	ref|NC_007622.1|:c2239297-2239118		
66	21	nr5611	ref|NC_007622.1|:c2291552-2291448		
66	22	nr5612	ref|NC_007622.1|:2302406-2302522		

66	23	nr5613	ref|NC_007622.1|:2309331-2309831
66	24	nr5614	ref|NC_007622.1|:2309828-2310652
66	25	nr5615	ref|NC_007622.1|:c2312157-2311963
66	26	nr5616	ref|NC_007622.1|:c2348601-2348467
66	27	nr5617	ref|NC_007622.1|:2352646-2352834
66	28	nr5618	ref|NC_007622.1|:c2376430-2376302
66	29	nr5619	ref|NC_007622.1|:2389855-2390028
66	30	nr5620	ref|NC_007622.1|:c2409123-2408998
66	31	nr5621	ref|NC_007622.1|:c2430560-2430378
66	32	nr5622	ref|NC_007622.1|:c2443767-2443573
66	33	nr5623	ref|NC_007622.1|:c2473598-2473470
66	34	nr5624	ref|NC_007622.1|:c2497495-2497340
66	35	nr5625	ref|NC_007622.1|:c2502770-2501562
66	36	nr5626	ref|NC_007622.1|:c2504432-2502840
66	37	nr5627	ref|NC_007622.1|:2542989-2543153
66	38	nr5628	ref|NC_007622.1|:c2557560-2556895
66	39	nr5629	ref|NC_007622.1|:c2582174-2582028
66	40	nr5630	ref|NC_007622.1|:c2591195-2590935
66	41	nr5631	ref|NC_007622.1|:c2598661-2598488
66	42	nr5632	ref|NC_007622.1|:c2622011-2621844
66	43	nr5633	ref|NC_007622.1|:c2637347-2637087
66	44	nr5634	ref|NC_007622.1|:c2700930-2700775
66	45	nr5635	ref|NC_007622.1|:2723861-2724004
66	46	nr5636	ref|NC_007622.1|:c2730109-2729912
66	47	nr5637	ref|NC_007790.1|:1348-1464
66	48	nr5638	ref|NC_007790.1|:c2674-2093
66	49	nr5639	ref|NC_007792.1|:101-1060
66	50	nr5640	ref|NC_007792.1|:2162-5236
66	51	nr5641	ref|NC_007792.1|:5734-6408
66	52	nr5642	ref|NC_007792.1|:6681-7157
66	53	nr5643	ref|NC_007792.1|:c9867-9679
66	54	nr5644	ref|NC_007792.1|:10039-11013
66	55	nr5645	ref|NC_007792.1|:11030-11347
66	56	nr5646	ref|NC_007792.1|:11407-11814
66	57	nr5647	ref|NC_007792.1|:11798-12484
66	58	nr5648	ref|NC_007792.1|:12505-14517
66	59	nr5649	ref|NC_007792.1|:14529-15809
66	60	nr5650	ref|NC_007792.1|:15827-16903
66	61	nr5651	ref|NC_007792.1|:16913-17398
66	62	nr5652	ref|NC_007792.1|:17414-19516
66	63	nr5653	ref|NC_007792.1|:19532-19996
66	64	nr5654	ref|NC_007792.1|:19993-21633
66	65	nr5655	ref|NC_007792.1|:21711-22628
66	66	nr5656	ref|NC_007792.1|:22645-23037
66	67	nr5657	ref|NC_007792.1|:23094-23261
66	68	nr5658	ref|NC_007792.1|:30381-32105
66	69	nr5659	ref|NC_007792.1|:c34144-33233
66	70	nr5660	ref|NC_007792.1|:34288-34620
66	71	nr5661	ref|NC_007792.1|:35220-36260

66	72	nr5662	ref|NC_007792.1|:36263-36592
66	73	nr5663	ref|NC_007792.1|:c36856-36596
66	74	nr5664	ref|NC_007793.1|:59098-60063
66	75	nr5665	ref|NC_007793.1|:60160-60351
66	76	nr5666	ref|NC_007793.1|:60443-60772
66	77	nr5667	ref|NC_007793.1|:c61187-60849
66	78	nr5668	ref|NC_007793.1|:c76225-76073
66	79	nr5669	ref|NC_007793.1|:77130-77945
66	80	nr5670	ref|NC_007793.1|:344015-344506
66	81	nr5671	ref|NC_007793.1|:347772-348158
66	82	nr5672	ref|NC_007793.1|:466923-467693
66	83	nr5673	ref|NC_007793.1|:876641-876754
66	84	nr5674	ref|NC_007793.1|:c885259-884801
66	85	nr5675	ref|NC_007793.1|:988576-988833
66	86	nr5676	ref|NC_007793.1|:1443183-1443668
67	1	nr5677	ref|NC_007793.1|:c1542406-1542287
67	2	nr5678	ref|NC_007793.1|:c1549931-1548477
67	3	nr5679	ref|NC_007793.1|:c1579253-1579047
67	4	nr5680	ref|NC_007793.1|:c1590607-1589993
67	5	nr5681	ref|NC_007793.1|:1924777-1924932
67	6	nr5682	ref|NC_007793.1|:c1938575-1936836
67	7	nr5683	ref|NC_007793.1|:c1978089-1977991
67	8	nr5684	ref|NC_007793.1|:c2090962-2090666
67	9	nr5685	ref|NC_007793.1|:c2111524-2111138
67	10	nr5686	ref|NC_007793.1|:2121059-2121298
67	11	nr5687	ref|NC_007793.1|:c2285693-2282385
67	12	nr5688	ref|NC_007793.1|:2290382-2291620
67	13	nr5689	ref|NC_007793.1|:2510616-2510822
67	14	nr5690	ref|NC_007793.1|:c2613090-2612848
67	15	nr5691	ref|NC_007793.1|:2862145-2862336
67	16	nr5692	ref|NC_007795.1|:c34034-33555
67	17	nr5693	ref|NC_007795.1|:c34509-34375
67	18	nr5694	ref|NC_007795.1|:c37120-37019
67	19	nr5695	ref|NC_007795.1|:c91096-90905
67	20	nr5696	ref|NC_007795.1|:c102452-102228
67	21	nr5697	ref|NC_007795.1|:114927-115214
67	22	nr5698	ref|NC_007795.1|:115867-116088
67	23	nr5699	ref|NC_007795.1|:c213931-213824
67	24	nr5700	ref|NC_007795.1|:c214120-213950
67	25	nr5701	ref|NC_007795.1|:227920-228027
67	26	nr5702	ref|NC_007795.1|:232547-232645
67	27	nr5703	ref|NC_007795.1|:c241857-241570
67	28	nr5704	ref|NC_007795.1|:259676-259810
67	29	nr5705	ref|NC_007795.1|:264380-264784
67	30	nr5706	ref|NC_007795.1|:c300439-300206
67	31	nr5707	ref|NC_007795.1|:c302476-302336
67	32	nr5708	ref|NC_007795.1|:c347893-347735
67	33	nr5709	ref|NC_007795.1|:c363534-363376
67	34	nr5710	ref|NC_007795.1|:c393878-393579

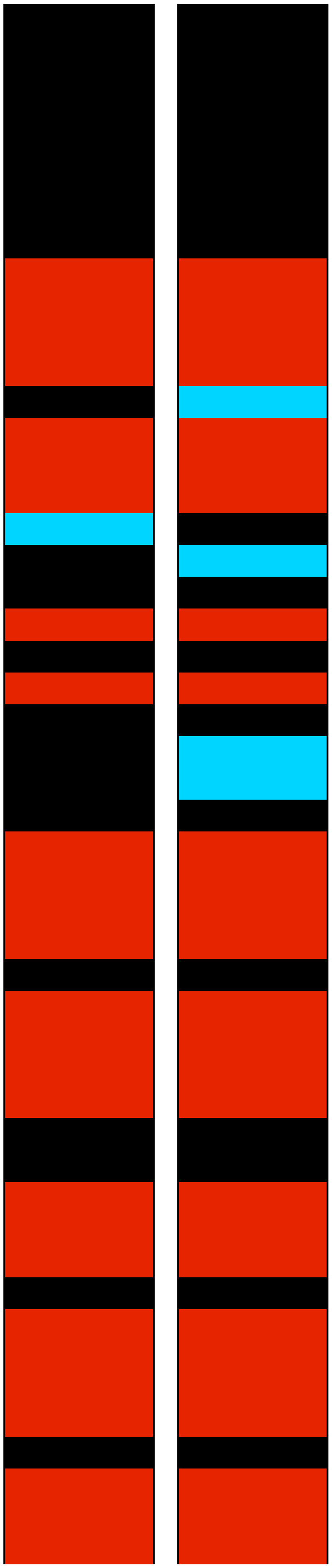

67	35	nr5711	ref|NC_007795.1|:448172-448270
67	36	nr5712	ref|NC_007795.1|:c457338-457162
67	37	nr5713	ref|NC_007795.1|:c472207-472097
67	38	nr5714	ref|NC_007795.1|:c507768-507610
67	39	nr5715	ref|NC_007795.1|:c547865-547608
67	40	nr5716	ref|NC_007795.1|:589698-589814
67	41	nr5717	ref|NC_007795.1|:591777-591905
67	42	nr5718	ref|NC_007795.1|:c649917-649729
67	43	nr5719	ref|NC_007795.1|:666157-666273
67	44	nr5720	ref|NC_007795.1|:668104-668199
67	45	nr5721	ref|NC_007795.1|:c694114-693941
67	46	nr5722	ref|NC_007795.1|:c712393-712235
67	47	nr5723	ref|NC_007795.1|:722569-722670
67	48	nr5724	ref|NC_007795.1|:730850-731005
67	49	nr5725	ref|NC_007795.1|:757511-758527
67	50	nr5726	ref|NC_007795.1|:c758723-758565
67	51	nr5727	ref|NC_007795.1|:758680-759654
67	52	nr5728	ref|NC_007795.1|:795990-796109
67	53	nr5729	ref|NC_007795.1|:811872-811985
67	54	nr5730	ref|NC_007795.1|:826353-826451
67	55	nr5731	ref|NC_007795.1|:860487-860678
67	56	nr5732	ref|NC_007795.1|:942903-943004
67	57	nr5733	ref|NC_007795.1|:947205-947912
67	58	nr5734	ref|NC_007795.1|:963913-964023
67	59	nr5735	ref|NC_007795.1|:976868-976990
67	60	nr5736	ref|NC_007795.1|:c1006364-1006242
67	61	nr5737	ref|NC_007795.1|:c1041955-1041731
67	62	nr5738	ref|NC_007795.1|:1048947-1049240
67	63	nr5739	ref|NC_007795.1|:c1071275-1071183
67	64	nr5740	ref|NC_007795.1|:1074797-1074952
67	65	nr5741	ref|NC_007795.1|:1090331-1090558
67	66	nr5742	ref|NC_007795.1|:c1090661-1090500
67	67	nr5743	ref|NC_007795.1|:1162045-1162206
67	68	nr5744	ref|NC_007795.1|:1180529-1180621
67	69	nr5745	ref|NC_007795.1|:1249705-1249827
67	70	nr5746	ref|NC_007795.1|:c1268229-1268125
67	71	nr5747	ref|NC_007795.1|:1283879-1283986
67	72	nr5748	ref|NC_007795.1|:c1296825-1296727
67	73	nr5749	ref|NC_007795.1|:c1331339-1331232
67	74	nr5750	ref|NC_007795.1|:c1332855-1332694
67	75	nr5751	ref|NC_007795.1|:1346152-1346340
67	76	nr5752	ref|NC_007795.1|:c1349016-1348900
67	77	nr5753	ref|NC_007795.1|:c1362685-1362554
67	78	nr5754	ref|NC_007795.1|:1367130-1367285
67	79	nr5755	ref|NC_007795.1|:c1375701-1375528
67	80	nr5756	ref|NC_007795.1|:1416796-1416948
67	81	nr5757	ref|NC_007795.1|:1445100-1445414
67	82	nr5758	ref|NC_007795.1|:1450171-1450308
67	83	nr5759	ref|NC_007795.1|:c1462718-1462371

67	84	nr5760	ref|NC_007795.1|:c1463799-1462963
67	85	nr5761	ref|NC_007795.1|:c1491310-1491020
67	86	nr5762	ref|NC_007795.1|:c1491503-1491243
68	1	nr5763	ref|NC_007795.1|:c1494307-1494119
68	2	nr5764	ref|NC_007795.1|:c1494886-1494344
68	3	nr5765	ref|NC_007795.1|:c1495127-1494891
68	4	nr5766	ref|NC_007795.1|:c1495428-1495120
68	5	nr5767	ref|NC_007795.1|:c1502416-1502255
68	6	nr5768	ref|NC_007795.1|:c1504015-1503842
68	7	nr5769	ref|NC_007795.1|:1506145-1506372
68	8	nr5770	ref|NC_007795.1|:1506409-1506555
68	9	nr5771	ref|NC_007795.1|:c1507166-1506552
68	10	nr5772	ref|NC_007795.1|:1532809-1532901
68	11	nr5773	ref|NC_007795.1|:c1556727-1556581
68	12	nr5774	ref|NC_007795.1|:c1562910-1562458
68	13	nr5775	ref|NC_007795.1|:1582589-1582765
68	14	nr5776	ref|NC_007795.1|:c1597754-1597626
68	15	nr5777	ref|NC_007795.1|:c1610792-1610631
68	16	nr5778	ref|NC_007795.1|:c1632925-1632623
68	17	nr5779	ref|NC_007795.1|:c1636727-1636551
68	18	nr5780	ref|NC_007795.1|:c1638975-1638847
68	19	nr5781	ref|NC_007795.1|:1664224-1664334
68	20	nr5782	ref|NC_007795.1|:c1680746-1680636
68	21	nr5783	ref|NC_007795.1|:1682384-1682485
68	22	nr5784	ref|NC_007795.1|:c1688013-1687891
68	23	nr5785	ref|NC_007795.1|:1701056-1701274
68	24	nr5786	ref|NC_007795.1|:c1706480-1705674
68	25	nr5787	ref|NC_007795.1|:c1707325-1706498
68	26	nr5788	ref|NC_007795.1|:1735252-1735506
68	27	nr5789	ref|NC_007795.1|:1739623-1739718
68	28	nr5790	ref|NC_007795.1|:c1747288-1747196
68	29	nr5791	ref|NC_007795.1|:c1757521-1757408
68	30	nr5792	ref|NC_007795.1|:c1759531-1759427
68	31	nr5793	ref|NC_007795.1|:c1795592-1795368
68	32	nr5794	ref|NC_007795.1|:c1796080-1795589
68	33	nr5795	ref|NC_007795.1|:1814821-1815429
68	34	nr5796	ref|NC_007795.1|:1833027-1833128
68	35	nr5797	ref|NC_007795.1|:1844134-1844241
68	36	nr5798	ref|NC_007795.1|:1897329-1898531
68	37	nr5799	ref|NC_007795.1|:1899812-1899976
68	38	nr5800	ref|NC_007795.1|:1900176-1900424
68	39	nr5801	ref|NC_007795.1|:1904764-1904943
68	40	nr5802	ref|NC_007795.1|:c1912117-1912025
68	41	nr5803	ref|NC_007795.1|:1917267-1917497
68	42	nr5804	ref|NC_007795.1|:1923456-1923557
68	43	nr5805	ref|NC_007795.1|:c1930486-1930187
68	44	nr5806	ref|NC_007795.1|:c1951095-1950673
68	45	nr5807	ref|NC_007795.1|:c1951642-1951442
68	46	nr5808	ref|NC_007795.1|:c1951805-1951617

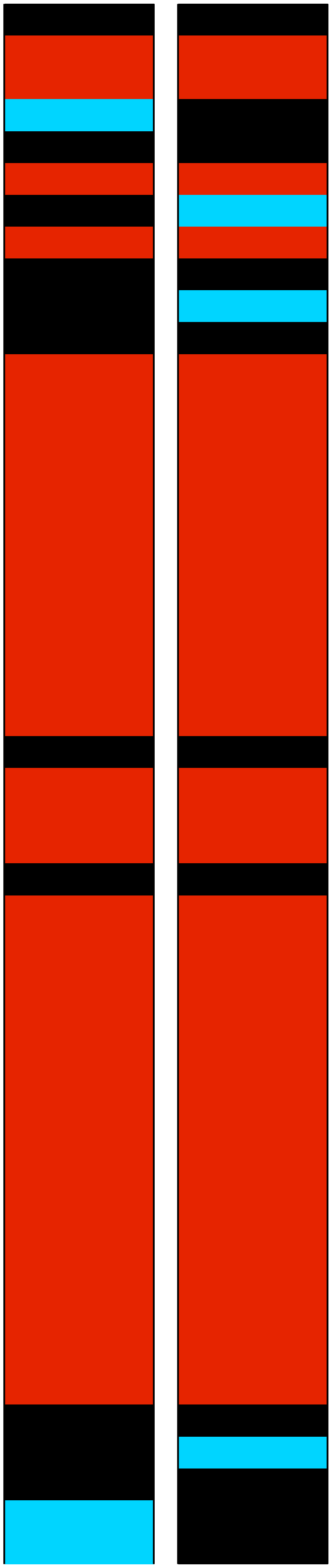

68	47	nr5809	ref|NC_007795.1|:c1953693-1953334
68	48	nr5810	ref|NC_007795.1|:c1954149-1953964
68	49	nr5811	ref|NC_007795.1|:c1958070-1957378
68	50	nr5812	ref|NC_007795.1|:c1958583-1958083
68	51	nr5813	ref|NC_007795.1|:c1959446-1958667
68	52	nr5814	ref|NC_007795.1|:c1959983-1959447
68	53	nr5815	ref|NC_007795.1|:c1961494-1961045
68	54	nr5816	ref|NC_007795.1|:1963392-1964111
68	55	nr5817	ref|NC_007795.1|:1964181-1964348
68	56	nr5818	ref|NC_007795.1|:1964508-1964693
68	57	nr5819	ref|NC_007795.1|:1964729-1965634
68	58	nr5820	ref|NC_007795.1|:c1965771-1965571
68	59	nr5821	ref|NC_007795.1|:c1976960-1976772
68	60	nr5822	ref|NC_007795.1|:c1988265-1988119
68	61	nr5823	ref|NC_007795.1|:2037430-2037537
68	62	nr5824	ref|NC_007795.1|:c2060601-2060407
68	63	nr5825	ref|NC_007795.1|:c2060818-2060564
68	64	nr5826	ref|NC_007795.1|:c2061544-2061197
68	65	nr5827	ref|NC_007795.1|:c2061856-2061608
68	66	nr5828	ref|NC_007795.1|:c2062823-2062419
68	67	nr5829	ref|NC_007795.1|:c2063055-2062834
68	68	nr5830	ref|NC_007795.1|:c2067310-2066531
68	69	nr5831	ref|NC_007795.1|:c2067794-2067534
68	70	nr5832	ref|NC_007795.1|:c2068048-2067887
68	71	nr5833	ref|NC_007795.1|:c2070204-2069848
68	72	nr5834	ref|NC_007795.1|:2071485-2072255
68	73	nr5835	ref|NC_007795.1|:2072672-2072818
68	74	nr5836	ref|NC_007795.1|:2096704-2096811
68	75	nr5837	ref|NC_007795.1|:2111333-2111488
68	76	nr5838	ref|NC_007795.1|:c2122738-2122583
68	77	nr5839	ref|NC_007795.1|:c2128323-2128216
68	78	nr5840	ref|NC_007795.1|:c2134653-2134363
68	79	nr5841	ref|NC_007795.1|:2134741-2134974
68	80	nr5842	ref|NC_007795.1|:c2157702-2157595
68	81	nr5843	ref|NC_007795.1|:c2167101-2167009
68	82	nr5844	ref|NC_007795.1|:c2193879-2193721
68	83	nr5845	ref|NC_007795.1|:2200893-2201021
68	84	nr5846	ref|NC_007795.1|:c2207833-2207609
68	85	nr5847	ref|NC_007795.1|:2214325-2214417
68	86	nr5848	ref|NC_007795.1|:2214493-2214651
69	1	nr5849	ref|NC_007795.1|:2238622-2238726
69	2	nr5850	ref|NC_007795.1|:c2238868-2238752
69	3	nr5851	ref|NC_007795.1|:c2244724-2244539
69	4	nr5852	ref|NC_007795.1|:2264437-2265237
69	5	nr5853	ref|NC_007795.1|:2265255-2265554
69	6	nr5854	ref|NC_007795.1|:2265717-2266061
69	7	nr5855	ref|NC_007795.1|:2284713-2284880
69	8	nr5856	ref|NC_007795.1|:c2296682-2296590
69	9	nr5857	ref|NC_007795.1|:c2297688-2297395

69	10	nr5858	ref|NC_007795.1|:c2298782-2297685
69	11	nr5859	ref|NC_007795.1|:2298943-2299110
69	12	nr5860	ref|NC_007795.1|:c2322780-2322685
69	13	nr5861	ref|NC_007795.1|:c2335942-2335823
69	14	nr5862	ref|NC_007795.1|:c2349891-2349715
69	15	nr5863	ref|NC_007795.1|:2358144-2358323
69	16	nr5864	ref|NC_007795.1|:2391278-2391637
69	17	nr5865	ref|NC_007795.1|:c2420631-2420344
69	18	nr5866	ref|NC_007795.1|:2423112-2423381
69	19	nr5867	ref|NC_007795.1|:c2423618-2423442
69	20	nr5868	ref|NC_007795.1|:2425635-2425991
69	21	nr5869	ref|NC_007795.1|:c2441048-2440932
69	22	nr5870	ref|NC_007795.1|:c2446371-2446156
69	23	nr5871	ref|NC_007795.1|:c2484393-2484250
69	24	nr5872	ref|NC_007795.1|:c2487288-2487184
69	25	nr5873	ref|NC_007795.1|:c2488815-2488633
69	26	nr5874	ref|NC_007795.1|:c2498230-2498063
69	27	nr5875	ref|NC_007795.1|:c2502583-2502476
69	28	nr5876	ref|NC_007795.1|:c2513085-2512984
69	29	nr5877	ref|NC_007795.1|:c2515072-2514968
69	30	nr5878	ref|NC_007795.1|:2517701-2517898
69	31	nr5879	ref|NC_007795.1|:c2524899-2524759
69	32	nr5880	ref|NC_007795.1|:2553422-2553643
69	33	nr5881	ref|NC_007795.1|:2555609-2555794
69	34	nr5882	ref|NC_007795.1|:c2558924-2558631
69	35	nr5883	ref|NC_007795.1|:c2584524-2584423
69	36	nr5884	ref|NC_007795.1|:c2592045-2591200
69	37	nr5885	ref|NC_007795.1|:c2596044-2595946
69	38	nr5886	ref|NC_007795.1|:c2628810-2628706
69	39	nr5887	ref|NC_007795.1|:c2629107-2628973
69	40	nr5888	ref|NC_007795.1|:c2663303-2663175
69	41	nr5889	ref|NC_007795.1|:c2671855-2671337
69	42	nr5890	ref|NC_007795.1|:c2680430-2680332
69	43	nr5891	ref|NC_007795.1|:c2688060-2687866
69	44	nr5892	ref|NC_007795.1|:2705093-2705254
69	45	nr5893	ref|NC_007795.1|:c2705380-2705270
69	46	nr5894	ref|NC_007795.1|:2714168-2714338
69	47	nr5895	ref|NC_007795.1|:2719452-2719625
69	48	nr5896	ref|NC_007795.1|:c2731855-2731688
69	49	nr5897	ref|NC_007795.1|:c2810839-2810600
69	50	nr5898	ref|NC_009477.1|:21895-22569
69	51	nr5899	ref|NC_009487.1|:c49180-48518
69	52	nr5900	ref|NC_009487.1|:49676-50407
69	53	nr5901	ref|NC_009487.1|:c51315-50533
69	54	nr5902	ref|NC_009487.1|:c51843-51466
69	55	nr5903	ref|NC_009487.1|:c53742-51850
69	56	nr5904	ref|NC_009487.1|:c54824-53739
69	57	nr5905	ref|NC_009487.1|:362475-362654
69	58	nr5906	ref|NC_009487.1|:c362776-362669

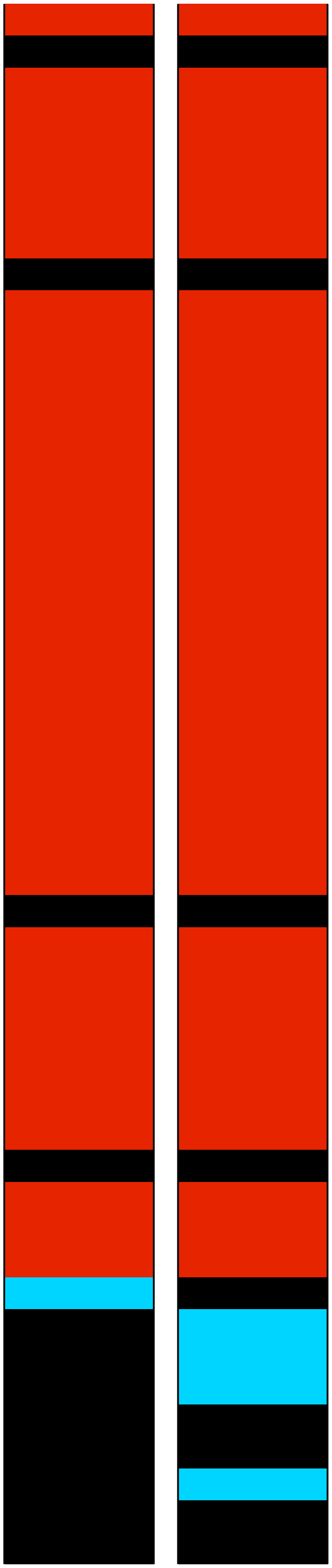

69	59	nr5907	ref|NC_009487.1|:c363271-362780
69	60	nr5908	ref|NC_009487.1|:364516-364677
69	61	nr5909	ref|NC_009487.1|:366312-366530
69	62	nr5910	ref|NC_009487.1|:366560-366823
69	63	nr5911	ref|NC_009487.1|:366835-366996
69	64	nr5912	ref|NC_009487.1|:367091-367393
69	65	nr5913	ref|NC_009487.1|:367398-367658
69	66	nr5914	ref|NC_009487.1|:367667-367930
69	67	nr5915	ref|NC_009487.1|:367939-369882
69	68	nr5916	ref|NC_009487.1|:369884-370804
69	69	nr5917	ref|NC_009487.1|:371503-371973
69	70	nr5918	ref|NC_009487.1|:372003-372896
69	71	nr5919	ref|NC_009487.1|:372903-373121
69	72	nr5920	ref|NC_009487.1|:373130-373534
69	73	nr5921	ref|NC_009487.1|:373547-373915
69	74	nr5922	ref|NC_009487.1|:374166-374381
69	75	nr5923	ref|NC_009487.1|:374384-374785
69	76	nr5924	ref|NC_009487.1|:374782-375129
69	77	nr5925	ref|NC_009487.1|:375427-375675
69	78	nr5926	ref|NC_009487.1|:375668-376204
69	79	nr5927	ref|NC_009487.1|:376241-376477
69	80	nr5928	ref|NC_009487.1|:376502-376738
69	81	nr5929	ref|NC_009487.1|:377288-377653
69	82	nr5930	ref|NC_009487.1|:378574-379068
69	83	nr5931	ref|NC_009487.1|:379061-380269
69	84	nr5932	ref|NC_009487.1|:384176-384502
69	85	nr5933	ref|NC_009487.1|:384502-384816
69	86	nr5934	ref|NC_009487.1|:384809-385144
70	1	nr5935	ref|NC_009487.1|:385131-385544
70	2	nr5936	ref|NC_009487.1|:385981-386541
70	3	nr5937	ref|NC_009487.1|:387118-387459
70	4	nr5938	ref|NC_009487.1|:390446-391381
70	5	nr5939	ref|NC_009487.1|:391392-393278
70	6	nr5940	ref|NC_009487.1|:393291-395189
70	7	nr5941	ref|NC_009487.1|:395189-397012
70	8	nr5942	ref|NC_009487.1|:397012-397389
70	9	nr5943	ref|NC_009487.1|:397390-397566
70	10	nr5944	ref|NC_009487.1|:397607-397906
70	11	nr5945	ref|NC_009487.1|:398043-399917
70	12	nr5946	ref|NC_009487.1|:399930-401102
70	13	nr5947	ref|NC_009487.1|:401559-401996
70	14	nr5948	ref|NC_009487.1|:401977-403422
70	15	nr5949	ref|NC_009487.1|:404190-404312
70	16	nr5950	ref|NC_009487.1|:c720144-718825
70	17	nr5951	ref|NC_009487.1|:c754726-753407
70	18	nr5952	ref|NC_009487.1|:c923573-922848
70	19	nr5953	ref|NC_009487.1|:927045-927347
70	20	nr5954	ref|NC_009487.1|:932611-932916
70	21	nr5955	ref|NC_009487.1|:933404-933646

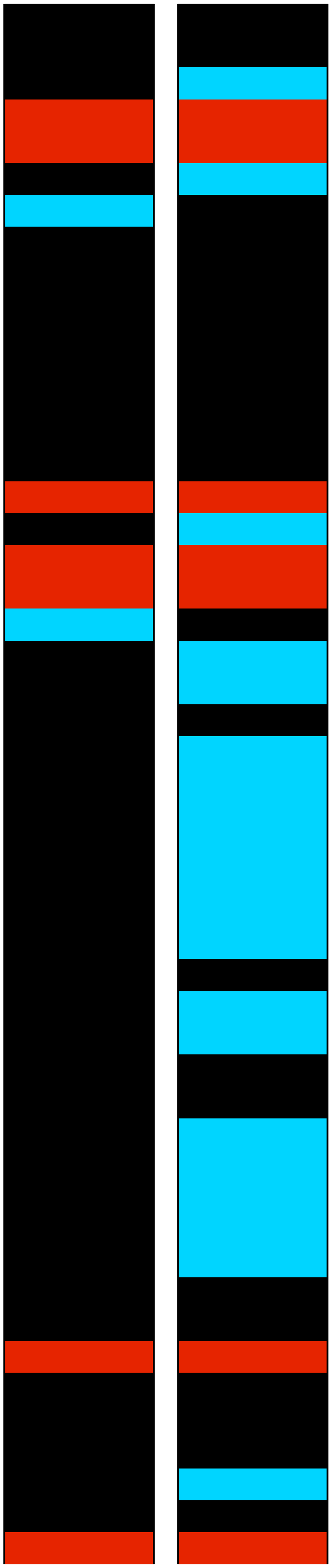

70	22	nr5956	ref|NC_009487.1|:934019-934273
70	23	nr5957	ref|NC_009487.1|:934993-935199
70	24	nr5958	ref|NC_009487.1|:935809-935985
70	25	nr5959	ref|NC_009487.1|:935986-936090
70	26	nr5960	ref|NC_009487.1|:936156-936578
70	27	nr5961	ref|NC_009487.1|:960439-960843
70	28	nr5962	ref|NC_009487.1|:972854-974173
70	29	nr5963	ref|NC_009487.1|:c1077516-1076311
70	30	nr5964	ref|NC_009487.1|:c1079692-1079231
70	31	nr5965	ref|NC_009487.1|:c1080019-1079705
70	32	nr5966	ref|NC_009487.1|:1080171-1080407
70	33	nr5967	ref|NC_009487.1|:1080421-1081197
70	34	nr5968	ref|NC_009487.1|:1088080-1088334
70	35	nr5969	ref|NC_009487.1|:1088340-1088582
70	36	nr5970	ref|NC_009487.1|:1089539-1089928
70	37	nr5971	ref|NC_009487.1|:1090162-1090698
70	38	nr5972	ref|NC_009487.1|:1263662-1264981
70	39	nr5973	ref|NC_009487.1|:c1650269-1650015
70	40	nr5974	ref|NC_009487.1|:2023851-2025170
70	41	nr5975	ref|NC_009487.1|:2038572-2039891
70	42	nr5976	ref|NC_009487.1|:c2165982-2165233
70	43	nr5977	ref|NC_009487.1|:2166039-2166248
70	44	nr5978	ref|NC_009487.1|:2168914-2169060
70	45	nr5979	ref|NC_009487.1|:c2242080-2240761
70	46	nr5980	ref|NC_009487.1|:c2625432-2625334
70	47	nr5981	ref|NC_009619.1|:7086-7760
70	48	nr5982	ref|NC_009619.1|:c27359-26838
70	49	nr5983	ref|NC_009632.1|:c49249-48587
70	50	nr5984	ref|NC_009632.1|:49745-50476
70	51	nr5985	ref|NC_009632.1|:c51384-50602
70	52	nr5986	ref|NC_009632.1|:c51912-51535
70	53	nr5987	ref|NC_009632.1|:c53811-51919
70	54	nr5988	ref|NC_009632.1|:c54893-53808
70	55	nr5989	ref|NC_009632.1|:c363823-363362
70	56	nr5990	ref|NC_009632.1|:c364988-364740
70	57	nr5991	ref|NC_009632.1|:365427-365612
70	58	nr5992	ref|NC_009632.1|:367161-367463
70	59	nr5993	ref|NC_009632.1|:367468-367728
70	60	nr5994	ref|NC_009632.1|:367737-368000
70	61	nr5995	ref|NC_009632.1|:368009-369952
70	62	nr5996	ref|NC_009632.1|:369954-370874
70	63	nr5997	ref|NC_009632.1|:371087-371572
70	64	nr5998	ref|NC_009632.1|:371573-372043
70	65	nr5999	ref|NC_009632.1|:372073-372966
70	66	nr6000	ref|NC_009632.1|:372973-373191
70	67	nr6001	ref|NC_009632.1|:373200-373604
70	68	nr6002	ref|NC_009632.1|:374852-375199
70	69	nr6003	ref|NC_009632.1|:377184-377357
70	70	nr6004	ref|NC_009632.1|:377724-377870

70	71	nr6005	ref|NC_009632.1|:377894-378316
70	72	nr6006	ref|NC_009632.1|:380293-381771
70	73	nr6007	ref|NC_009632.1|:c720020-718701
70	74	nr6008	ref|NC_009632.1|:c754602-753283
70	75	nr6009	ref|NC_009632.1|:926665-926826
70	76	nr6010	ref|NC_009632.1|:927227-927487
70	77	nr6011	ref|NC_009632.1|:929447-929596
70	78	nr6012	ref|NC_009632.1|:933530-933901
70	79	nr6013	ref|NC_009632.1|:c967647-967513
70	80	nr6014	ref|NC_009632.1|:972729-974048
70	81	nr6015	ref|NC_009632.1|:1088470-1088676
70	82	nr6016	ref|NC_009632.1|:1089795-1090043
70	83	nr6017	ref|NC_009632.1|:1119613-1121058
70	84	nr6018	ref|NC_009632.1|:c1121335-1121234
70	85	nr6019	ref|NC_009632.1|:1263536-1264855
70	86	nr6020	ref|NC_009632.1|:1492410-1492514
71	1	nr6021	ref|NC_009632.1|:2023726-2025045
71	2	nr6022	ref|NC_009632.1|:2038447-2039766
71	3	nr6023	ref|NC_009632.1|:c2193882-2193769
71	4	nr6024	ref|NC_009632.1|:c2241955-2240636
71	5	nr6025	ref|NC_009632.1|:2387910-2388095
71	6	nr6026	ref|NC_009632.1|:c2567346-2567254
71	7	nr6027	ref|NC_009641.1|:45582-45884
71	8	nr6028	ref|NC_009641.1|:271653-271775
71	9	nr6029	ref|NC_009641.1|:c323253-322321
71	10	nr6030	ref|NC_009641.1|:c325240-324758
71	11	nr6031	ref|NC_009641.1|:326841-327101
71	12	nr6032	ref|NC_009641.1|:327325-328113
71	13	nr6033	ref|NC_009641.1|:328142-328693
71	14	nr6034	ref|NC_009641.1|:328706-329377
71	15	nr6035	ref|NC_009641.1|:c330340-329483
71	16	nr6036	ref|NC_009641.1|:330405-331175
71	17	nr6037	ref|NC_009641.1|:331185-331964
71	18	nr6038	ref|NC_009641.1|:332360-332767
71	19	nr6039	ref|NC_009641.1|:332953-333312
71	20	nr6040	ref|NC_009641.1|:333312-333566
71	21	nr6041	ref|NC_009641.1|:333572-333814
71	22	nr6042	ref|NC_009641.1|:333829-334230
71	23	nr6043	ref|NC_009641.1|:334418-334867
71	24	nr6044	ref|NC_009641.1|:334864-335148
71	25	nr6045	ref|NC_009641.1|:335382-335918
71	26	nr6046	ref|NC_009641.1|:337331-337732
71	27	nr6047	ref|NC_009641.1|:338570-339793
71	28	nr6048	ref|NC_009641.1|:339790-341214
71	29	nr6049	ref|NC_009641.1|:c342334-342005
71	30	nr6050	ref|NC_009641.1|:342447-343031
71	31	nr6051	ref|NC_009641.1|:343048-343962
71	32	nr6052	ref|NC_009641.1|:344123-344473
71	33	nr6053	ref|NC_009641.1|:346969-347118

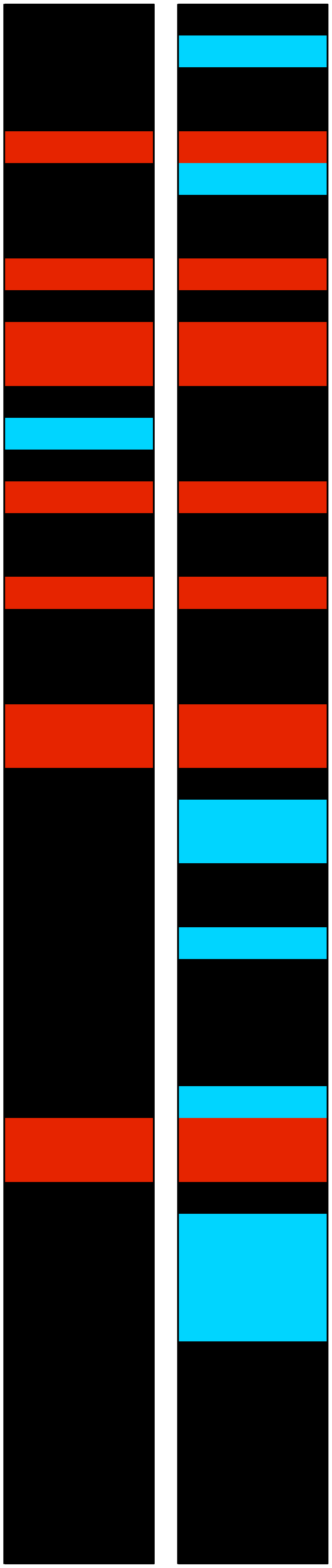

71	34	nr6054	ref|NC_009641.1|:357882-359780
71	35	nr6055	ref|NC_009641.1|:359793-360965
71	36	nr6056	ref|NC_009641.1|:361422-361724
71	37	nr6057	ref|NC_009641.1|:361736-363190
71	38	nr6058	ref|NC_009641.1|:650985-651317
71	39	nr6059	ref|NC_009641.1|:c670099-669950
71	40	nr6060	ref|NC_009641.1|:968294-968395
71	41	nr6061	ref|NC_009641.1|:c1100283-1098898
71	42	nr6062	ref|NC_009641.1|:1105075-1105554
71	43	nr6063	ref|NC_009641.1|:1106357-1106908
71	44	nr6064	ref|NC_009641.1|:1106921-1107592
71	45	nr6065	ref|NC_009641.1|:c1108555-1107698
71	46	nr6066	ref|NC_009641.1|:1108620-1109390
71	47	nr6067	ref|NC_009641.1|:1109400-1110179
71	48	nr6068	ref|NC_009641.1|:1110575-1110982
71	49	nr6069	ref|NC_009641.1|:1111168-1111527
71	50	nr6070	ref|NC_009641.1|:1111527-1111781
71	51	nr6071	ref|NC_009641.1|:1111772-1112029
71	52	nr6072	ref|NC_009641.1|:1112044-1112445
71	53	nr6073	ref|NC_009641.1|:1112633-1113082
71	54	nr6074	ref|NC_009641.1|:1113079-1113363
71	55	nr6075	ref|NC_009641.1|:1113597-1114133
71	56	nr6076	ref|NC_009641.1|:1114880-1115302
71	57	nr6077	ref|NC_009641.1|:1115489-1115929
71	58	nr6078	ref|NC_009641.1|:1115916-1117193
71	59	nr6079	ref|NC_009641.1|:1117204-1118739
71	60	nr6080	ref|NC_009641.1|:1118746-1119741
71	61	nr6081	ref|NC_009641.1|:1120747-1121721
71	62	nr6082	ref|NC_009641.1|:1121743-1122030
71	63	nr6083	ref|NC_009641.1|:1122039-1122371
71	64	nr6084	ref|NC_009641.1|:1122368-1122670
71	65	nr6085	ref|NC_009641.1|:1122667-1123017
71	66	nr6086	ref|NC_009641.1|:1123029-1123412
71	67	nr6087	ref|NC_009641.1|:1123431-1124012
71	68	nr6088	ref|NC_009641.1|:1124075-1124440
71	69	nr6089	ref|NC_009641.1|:1124470-1124814
71	70	nr6090	ref|NC_009641.1|:1129264-1131165
71	71	nr6091	ref|NC_009641.1|:1131180-1133090
71	72	nr6092	ref|NC_009641.1|:1133090-1134913
71	73	nr6093	ref|NC_009641.1|:1134904-1135290
71	74	nr6094	ref|NC_009641.1|:1135508-1135807
71	75	nr6095	ref|NC_009641.1|:1135944-1137842
71	76	nr6096	ref|NC_009641.1|:1137855-1139027
71	77	nr6097	ref|NC_009641.1|:1139024-1139428
71	78	nr6098	ref|NC_009641.1|:1156561-1156632
71	79	nr6099	ref|NC_009641.1|:1164304-1164453
71	80	nr6100	ref|NC_009641.1|:1209938-1210090
71	81	nr6101	ref|NC_009641.1|:c1384374-1384012
71	82	nr6102	ref|NC_009641.1|:c1486435-1476047

71	83	nr6103	ref|NC_009641.1|:c1688561-1688370		
71	84	nr6104	ref|NC_009641.1|:c1983102-1982698		
71	85	nr6105	ref|NC_009641.1|:c1986618-1986319		
71	86	nr6106	ref|NC_009641.1|:c1989036-1987213		
72	1	nr6107	ref|NC_009641.1|:c1993818-1992871		
72	2	nr6108	ref|NC_009641.1|:c1997298-1993831		
72	3	nr6109	ref|NC_009641.1|:c2002009-2001395		
72	4	nr6110	ref|NC_009641.1|:c2009494-2009045		
72	5	nr6111	ref|NC_009641.1|:2013572-2014429		
72	6	nr6112	ref|NC_009641.1|:c2018761-2018015		
72	7	nr6113	ref|NC_009641.1|:2020923-2021855		
72	8	nr6114	ref|NC_009641.1|:c2118738-2118322		
72	9	nr6115	ref|NC_009641.1|:c2124449-2124189		
72	10	nr6116	ref|NC_009641.1|:2221163-2221270		
72	11	nr6117	ref|NC_009641.1|:2322965-2323285		
72	12	nr6118	ref|NC_009641.1|:2323433-2323792		
72	13	nr6119	ref|NC_009641.1|:c2406121-2405933		
72	14	nr6120	ref|NC_009641.1|:c2560147-2560001		
72	15	nr6121	ref|NC_009641.1|:c2635710-2635324		
72	16	nr6122	ref|NC_009641.1|:c2639214-2638828		
