# Supplementary material for: Genome sequencing and analysis reveals possible determinants of Staphylococcus aureus nasal carriage
Source: BMC Genomics. 2008 Sep 22;9:433. doi: 10.1186/1471-2164-9-433 (PMC2566312; doi:10.1186/1471-2164-9-433)
Supplement: Additional file 2 — The file lists unique genes as assessed by this study in both D30(Table 1in Additional file) and 930918-3(Table2in Additional file). These genes are cross referenced by a) their NCBI accession numbers and b) project specific NRID and annotations where applicable are provided. Genes marked in turquoise blue belong to UNF set. [file 1471-2164-9-433-S2.pdf]

Supplementary Information 2:

These conventions are followed in the following tables (Table 1 and Table 2). The NRID was assigned in this study for one representative member of each orthologous gene family in all species and strains of *Staphylococcus* used in the study. The reference genome ID denotes the genome in which this ORF / gene was first described. Since we have only identified the presence or absence of genes, we have assigned gene coordinates from the reference genome (column 3). The annotations, when present were used from the genome sequencing project data (.ptt files). Otherwise they were assigned based on sequence similarity to proteins in the NR database. The highlighted (turquoise blue) rows denote the UNF genes of D30.

Page 2 through 4 (Table 1) lists the genes that are unique to D30 as compared to 930918-3. Page 5 through 12 (Table 2) enlist genes that are unique to 930918-3 w.r.t strain D30.

Table 1: The list of genes unique to strain D30 as compared to 930918-3.

NRID	Reference Genome ID	coordinates in reference Genome	Annotations
nr0027	NC_002745	36435..37109	transposase for IS-like element
nr0036	NC_002745	41781..42455	transposase for IS-like element
nr0162	NC_002745	182802..183977	capsular polysaccharide synthesis enzyme Cap5P
nr0232	NC_002745	273754..275316	hypothetical protein
nr0290	NC_002745	341260..341943	hypothetical protein
nr0328	NC_002745	377808..379763	hypothetical protein
nr0395	NC_002745	443054..444124	superantigen-like protein
nr0396	NC_002745	444488..445366	superantigen-like protein
nr1008	NC_002745	1104658..1106595	hypothetical protein
nr1173	NC_002745	1291028..1293646	DNA mismatch repair protein
nr1331	NC_002745	1493028..1495721	probable ATP-dependent DNA helicase dinG
nr1366	NC_002745	1529669..1530535	hypothetical protein
nr1840	NC_002745	2033608..2034144	hypothetical protein
nr1853	NC_002745	2041163..2041423	hypothetical protein
nr1885	NC_002745	2065678..2066058	BPI ORF-12
nr1886	NC_002745	2066368..2068077	BPI ORF-13:14
nr1887	NC_002745	2068091..2068960	hypothetical protein
nr2025	NC_002745	2210083..2211888	D-fructose-6-phosphate amidotransferase
nr2060	NC_002745	2264087..2265799	PTS system, lactose-specific IIBC component
nr2160	NC_002745	2343583..2345298	urease subunit alpha
nr2236	NC_002745	2421937..2423535	hypothetical protein
nr2249	NC_002745	2437337..2439442	hypothetical protein
nr2336	NC_002745	2531455..2533053	oligopeptide transporter putative substrate binding domain
nr2345	NC_002745	2541721..2542131	hypothetical protein
nr2416	NC_002745	2621015..2623120	ATP-dependent Clp proteinase chain clpL
nr2589	NC_002758	36436..37110	transposase for IS-like element
nr2641	NC_002758	878043..878612	similar to bacteriophage terminase small subunit
nr2761	NC_002774	8808..9482	probable transposase
nr2770	NC_002774	14781..15455	probable transposase
nr2771	NC_002774	15986..16651	probable transposase
nr2801	NC_002951	90216..90986	staphylococcus tandem lipoprotein

NRID	Reference Genome ID	coordinates in reference Genome	Annotations
nr2874	NC_002951	473486..473623	hypothetical protein
nr2954	NC_002951	1609250..1609591	hypothetical protein
nr2955	NC_002951	1609647..1610237	hypothetical protein
nr2956	NC_002951	1610244..1611290	traG protein, putative
nr2957	NC_002951	1611280..1613127	hypothetical protein
nr2958	NC_002951	1613132..1614490	FtsK/SpoIIIE family protein
nr2960	NC_002951	1617074..1617457	hypothetical protein
nr2961	NC_002951	1617469..1617729	hypothetical protein
nr2962	NC_002951	1617734..1618789	hypothetical protein
nr2963	NC_002951	1618850..1619941	replication initiation factor family protein
nr2964	NC_002951	1620116..1620418	hypothetical protein
nr2965	NC_002951	1620432..1620752	hypothetical protein
nr2966	NC_002951	1620903..1621187	hypothetical protein
nr2977	NC_002951	1909269..1912319	hypothetical protein
nr3054	NC_002952	36403..37077	putative transposase
nr3094	NC_002952	412912..413058	hypothetical protein
nr3095	NC_002952	413116..413499	BPI ORF-17
nr3115	NC_002952	734448..735122	putative transposase
nr3142	NC_002952	763700..764374	putative transposase
nr3421	NC_002976	257149..257823	IS431mec-like transposase
nr3753	NC_002976	1609626..1610300	IS431mec-like transposase
nr3754	NC_002976	1612361..1612684	IS431mec-like transposase
nr3757	NC_002976	1617188..1617610	IS431mec-like transposase
nr4215	NC_003923	1561457..1561657	Phi-PVL Hypothetical ORF
nr4262	NC_004461	70397..71071	transposase
nr4268	NC_004461	80680..81354	transposase
nr4574	NC_006663	988..1632	truncated IS431mec transposase
nr4587	NC_006663	15693..16367	IS431mec-like transposase
nr4588	NC_006663	16413..17039	truncated IS431mec transposase
nr4598	NC_007168	30130..30804	transposase for IS431mec
nr4609	NC_007168	56315..56458	transposase for IS431mec
nr4610	NC_007168	56624..56716	transposase for IS431mec
nr4611	NC_007168	56738..56989	transposase for IS431mec
nr4630	NC_007168	85196..85561	transposase for IS431mec
nr4631	NC_007168	85480..85857	transposase for IS431mec
nr4640	NC_007168	100329..101003	transposase for IS431mec

NRID	Reference Genome ID	coordinates in reference Genome	Annotations
nr4652	NC_007168	120143..120817	transposase for IS431mec
nr5331	NC_007168	2346486..2347160	transposase for IS431mec
nr5533	NC_007622	979690..980364	transposase
nr5600	NC_007622	2033802..2034671	hypothetical mobile element-associated protein
nr5678	NC_007793	2120436..2120076	Phage hypothetical protein
nr5763	NC_007795	1494119..1494307	hypothetical protein
nr5807	NC_007795	1951442..1951642	hypothetical protein
nr5808	NC_007795	1951617..1951805	hypothetical protein
nr5898	NC_009477	21895..22569	Integrase, catalytic region
nr5913	NC_009487	367398..367658	protein of unknown function DUF1108
nr5926	NC_009487	375668..376204	dUTPase
nr5971	NC_009487	1090162..1090698	dUTPase
nr5981	NC_009619	7086..7760	Integrase catalytic region
nr5993	NC_009632	367468..367728	protein of unknown function DUF1108
nr6018	NC_009632	1121234..1121335	hypothetical protein
nr6115	NC_009641	2124189..2124449	hypothetical protein

Table 2: Unique genes of strain 930918-3 as compared to strain D30.

NRID	Genome ID	Coordinate in Reference Genome	Annotation
nr0020	NC_002745	27440..28801	hypothetical protein
nr0049	NC_002745	54806..55474	hypothetical protein in transposon Tn554
nr0050	NC_002745	56002..56733	rRNA methylase
nr0051	NC_002745	56859..57641	O-nucleotidyllransferase(9)
nr0054	NC_002745	60065..61150	transposase A for Tn554
nr0094	NC_002745	103853..104623	hypothetical protein
nr0119	NC_002745	135490..137268	hypothetical protein
nr0165	NC_002745	185185..186672	hypothetical protein
nr0223	NC_002745	258866..261115	formate acetyltransferase
nr0250	NC_002745	294674..296368	hypothetical protein
nr0316	NC_002745	365458..367533	glycerol ester hydrolase
nr0425	NC_002745	472424..475129	hypothetical protein
nr0566	NC_002745	644319..645767	hypothetical protein
nr0655	NC_002745	731989..733620	hypothetical protein
nr0729	NC_002745	812935..814926	excinuclease ABC subunit B
nr0782	NC_002745	867008..868093	transposase A for Tn554
nr0785	NC_002745	870517..871299	O-nucleotidyllransferase(9)
nr0786	NC_002745	871425..872156	rRNA methylase
nr0787	NC_002745	872690..873352	hypothetical protein within Tn554
nr0917	NC_002745	1010350..1010595	hypothetical protein
nr1018	NC_002745	1115371..1117773	Phe-tRNA synthetase beta chain
nr1090	NC_002745	1193288..1195696	PriA, primosomal protein
nr1120	NC_002745	1226508..1229114	hypothetical protein
nr1152	NC_002745	1266443..1268539	polynucleotide phosphorylase/polyadenylase
nr1230	NC_002745	1356325..1358727	DNA topoisomerase IV subunit A
nr1326	NC_002745	1486656..1488839	PBP2
nr1528	NC_002745	1684668..1685330	hypothetical protein
nr1529	NC_002745	1685864..1686595	rRNA methylase Erm(A)
nr1530	NC_002745	1686721..1687503	O-nucleotidyllransferase(9)
nr1533	NC_002745	1689927..1691012	transposase A for Tn554
nr1537	NC_002745	1693943..1696573	valyl-tRNA synthetase
nr1556	NC_002745	1714023..1715960	threonyl-tRNA synthetase 1
nr1707	NC_002745	1894739..1897675	hypothetical protein
nr1804	NC_002745	2005721..2005900	hypothetical protein

NRID	Genome ID	Coordinate in Reference Genome	Annotation
nr1806	NC_002745	2006528..2006878	hypothetical protein
nr1809	NC_002745	2008308..2008571	truncated amidase
nr1810	NC_002745	2009086..2009577	STAPHYLOKINASE PRECURSOR
nr1811	NC_002745	2009768..2010523	lytic enzyme
nr1812	NC_002745	2010535..2010789	hypothetical protein
nr1813	NC_002745	2011001..2011135	hypothetical protein
nr1816	NC_002745	2012574..2012948	hypothetical protein
nr1817	NC_002745	2013004..2013333	hypothetical protein
nr1818	NC_002745	2013337..2013489	hypothetical protein
nr1836	NC_002745	2032777..2032980	hypothetical protein
nr1837	NC_002745	2032977..2033126	hypothetical protein
nr1838	NC_002745	2033123..2033329	hypothetical protein
nr1842	NC_002745	2034401..2034649	hypothetical protein
nr1854	NC_002745	2041428..2041730	hypothetical protein
nr1856	NC_002745	2041983..2042306	hypothetical protein
nr1858	NC_002745	2042728..2042925	hypothetical protein
nr1859	NC_002745	2042941..2043693	anti repressor
nr1868	NC_002745	2048483..2049520	integrase
nr1878	NC_002745	2061935..2062504	hypothetical protein
nr1888	NC_002745	2069025..2069351	hypothetical protein
nr1889	NC_002745	2069354..2069563	hypothetical protein
nr2012	NC_002745	2198997..2199659	hypothetical protein
nr2013	NC_002745	2200193..2200924	rRNA methylase Erm(A)
nr2014	NC_002745	2201050..2201832	O-nucleotidylltransferase(9)
nr2017	NC_002745	2204256..2205341	transposition regulatory protein tnpA
nr2308	NC_002745	2499585..2501663	hypothetical protein
nr2317	NC_002745	2511766..2512998	glycine betaine/carnitine/choline ABC transporter opuCA
nr2451	NC_002745	2658930..2660270	hypothetical protein
nr2465	NC_002745	2671339..2672001	hypothetical protein
nr2466	NC_002745	2672535..2673266	rRNA methylase Erm(A)
nr2467	NC_002745	2673392..2674174	O-nucleotidylltransferase(9)
nr2470	NC_002745	2676598..2677683	transposition regulatory protein tnpA
nr2487	NC_002745	2694277..2695986	choline dehydrogenase
nr2519	NC_002745	2739595..2741454	N-acetylmuramoyl-L-alanine amidase
nr2528	NC_002745	2753912..2755123	preprotein translocase subunit SecY
nr2585	NC_002745	2810511..2812388	tRNA uridine 5-carboxymethylaminomethyl modification enzyme GidA

NRID	Genome ID	Coordinate in Reference Genome	Annotation
nr2591	NC_002758	55963..56694	rRNA methylase
nr2592	NC_002758	56820..57602	O-nucleotidylltransferase
nr2595	NC_002758	60026..61111	transposase A
nr2629	NC_002758	870845..870991	hypothetical protein
nr2630	NC_002758	870984..871193	hypothetical protein
nr2631	NC_002758	871196..871513	hypothetical protein
nr2635	NC_002758	874585..874869	hypothetical protein
nr2659	NC_002758	924409..924630	hypothetical protein
nr2660	NC_002758	924623..924745	hypothetical protein
nr2664	NC_002758	927049..927330	hypothetical protein
nr2665	NC_002758	927395..928126	hypothetical protein
nr2666	NC_002758	928139..928924	hypothetical protein
nr2667	NC_002758	928921..929079	hypothetical protein
nr2668	NC_002758	929092..929313	hypothetical protein
nr2671	NC_002758	931368..931574	hypothetical protein
nr2675	NC_002758	932184..932570	hypothetical protein
nr2676	NC_002758	932570..932743	int gene activator RinB
nr2700	NC_002758	952933..953310	phiETA ORF58-like protein
nr2701	NC_002758	953314..953487	phiETA ORF59-like protein
nr2705	NC_002758	957119..957514	phi ETA orf 63-like protein
nr2733	NC_002758	2118362..2118622	phi PVL orf 39-like protein
nr2735	NC_002758	2119549..2120184	hypothetical protein
nr2737	NC_002758	2121958..2122218	phi PVL orf 32-like protein
nr2824	NC_002951	354785..355990	prophage L54a, integrase
nr2826	NC_002951	358218..358436	prophage L54a, Cro-related protein
nr2835	NC_002951	364751..365551	hypothetical protein
nr2836	NC_002951	365551..365907	hypothetical protein
nr2838	NC_002951	367142..367357	hypothetical protein
nr2839	NC_002951	367591..368016	prophage L54a, N-6-adenine-methyltransferase
nr2840	NC_002951	368422..368607	hypothetical protein
nr2864	NC_002951	389456..391039	hypothetical protein
nr2866	NC_002951	391345..393255	hypothetical protein
nr2903	NC_002951	903491..904711	pathogenicity island protein, integrase
nr2904	NC_002951	905552..906280	staphylococcal enterotoxin type I
nr2906	NC_002951	907268..907600	transcriptional regulator, Cro/CI family
nr2907	NC_002951	907793..908056	transcriptional regulator, putative

NRID	Genome ID	Coordinate in Reference Genome	Annotation
nr2908	NC_002951	908049..908321	pathogenicity island protein
nr3056	NC_002952	54694..55356	hypothetical protein
nr3057	NC_002952	55890..56621	rRNA adenine N-6-methyltransferase 1
nr3058	NC_002952	56747..57529	streptomycin 3''-adenylyltransferase 1
nr3061	NC_002952	59953..61038	transposase A 1
nr3096	NC_002952	418894..419547	hypothetical protein
nr3136	NC_002952	754041..754616	putative resolvase
nr3174	NC_002952	1618968..1621415	hypothetical protein
nr3178	NC_002952	1623806..1623997	hypothetical protein
nr3183	NC_002952	1625717..1627678	putative DNA polymerase
nr3196	NC_002952	1635366..1635488	hypothetical protein
nr3197	NC_002952	1635475..1636098	hypothetical protein
nr3202	NC_002952	1908514..1909329	potential ATP-binding protein
nr3203	NC_002952	1909322..1910764	transposase
nr3204	NC_002952	1910736..1911329	resolvase
nr3205	NC_002952	1911593..1911973	penicillinase repressor
nr3207	NC_002952	1913827..1914672	beta-lactamase precursor
nr3228	NC_002952	2125437..2125697	hypothetical protein
nr3232	NC_002952	2152640..2152846	hypothetical protein
nr3235	NC_002952	2160965..2161066	hypothetical protein
nr3301	NC_002953	985377..985553	hypothetical protein
nr3302	NC_002953	985550..985789	hypothetical protein
nr3305	NC_002953	995895..996101	hypothetical protein
nr3314	NC_002953	2042749..2042871	hypothetical protein
nr3315	NC_002953	2042931..2043377	hypothetical protein
nr3319	NC_002953	2045150..2045482	hypothetical protein
nr3320	NC_002953	2045472..2045804	hypothetical protein
nr3321	NC_002953	2045813..2045971	hypothetical protein
nr3325	NC_002953	2049175..2049375	hypothetical protein
nr3327	NC_002953	2051086..2051553	hypothetical protein
nr3328	NC_002953	2051682..2052035	hypothetical protein
nr3329	NC_002953	2052042..2052494	hypothetical protein
nr3332	NC_002953	2053959..2054111	hypothetical protein
nr3333	NC_002953	2064041..2064265	putative regulatory protein
nr3612	NC_002976	1252055..1252717	Tn554, hypothetical protein
nr3613	NC_002976	1253109..1253168	Tn554, peptide L

NRID	Genome ID	Coordinate in Reference Genome	Annotation
nr3614	NC_002976	1253251..1253982	Tn554, rRNA adenine N-6-methyltransferase
nr3615	NC_002976	1254108..1254890	Tn554, streptomycin 3''-adenylyltransferase
nr3618	NC_002976	1257314..1258399	Tn554, transposase A
nr3635	NC_002976	1398493..1399155	Tn554, hypothetical protein
nr3636	NC_002976	1399547..1399606	Tn554, peptide L
nr3637	NC_002976	1399689..1400420	Tn554, rRNA adenine N-6-methyltransferase
nr3638	NC_002976	1400546..1401328	Tn554, streptomycin 3''-adenylyltransferase
nr3641	NC_002976	1403752..1404837	Tn554, transposase A
nr4576	NC_006663	7077..7412	transcriptional regulator, putative
nr4577	NC_006663	7688..8482	aminoglycoside 3'-phosphotransferase
nr4578	NC_006663	8575..9117	streptothricin acetyltransferase
nr4579	NC_006663	9114..9953	aminoglycoside 6-adenylyltransferase
nr4581	NC_006663	10055..10789	methyltransferase, UbiE/COQ5 family
nr4582	NC_006663	10770..11639	hypothetical protein
nr5150	NC_007168	1824212..1825027	ATP-binding protein
nr5151	NC_007168	1825020..1826462	transposase for Tn552
nr5152	NC_007168	1826434..1827027	DNA-invertase in Staphylococcus aureus transposon Tn552
nr5278	NC_007168	2212628..2212885	IS1272 transposase
nr5316	NC_007168	2336827..2337363	hypothetical protein
nr5317	NC_007168	2337377..2337958	hypothetical protein
nr5451	NC_007168	2619289..2619690	hypothetical protein
nr5452	NC_007168	2619704..2620240	hypothetical protein
nr5506	NC_007622	392271..392588	bovine pathogenicity island protein Orf16
nr5599	NC_007622	2030553..2030726	hypothetical protein
nr5674	NC_007793	884801..885259	hypothetical protein
nr5679	NC_007793	1579047..1579253	phiSLT ORF 81b-like protein
nr5685	NC_007793	2111138..2111524	phi77 ORF031-like protein
nr5686	NC_007793	2121059..2121298	hypothetical protein
nr5766	NC_007795	1495120..1495428	PV83 orf 27-like protein
nr5769	NC_007795	1506145..1506372	hypothetical protein
nr5805	NC_007795	1930187..1930486	phi SLT orf 99-like protein
nr5809	NC_007795	1953334..1953693	phi PVL orf 50-like protein
nr5810	NC_007795	1953964..1954149	PV83 orf 23-like protein-related protein
nr5811	NC_007795	1957378..1958070	phi PV83 orf 19-like protein
nr5812	NC_007795	1958083..1958583	single-strand DNA-binding protein, putative
nr5813	NC_007795	1958667..1959446	hypothetical protein

NRID	Genome ID	Coordinate in Reference Genome	Annotation
nr5816	NC_007795	1963392..1964111	phage repressor protein, putative
nr5817	NC_007795	1964181..1964348	hypothetical protein
nr5823	NC_007795	2037430..2037537	hypothetical protein
nr5828	NC_007795	2062419..2062823	phi ETA orf 25-like protein
nr5829	NC_007795	2062834..2063055	hypothetical protein
nr5830	NC_007795	2066531..2067310	hypothetical protein
nr5831	NC_007795	2067534..2067794	phi PVL orf 39-like protein
nr5833	NC_007795	2069848..2070204	anti repressor
nr5834	NC_007795	2071485..2072255	repressor
nr5899	NC_009487	48518..49180	Methyltransferase type 12
nr5900	NC_009487	49676..50407	rRNA (adenine-N(6)-)-methyltransferase
nr5901	NC_009487	50533..51315	3''-adenylyltransferase
nr5904	NC_009487	53739..54824	phage integrase family protein
nr5909	NC_009487	366312..366530	hypothetical protein
nr5912	NC_009487	367091..367393	hypothetical protein
nr5923	NC_009487	374384..374785	hypothetical protein
nr5927	NC_009487	376241..376477	protein of unknown function DUF1381
nr5928	NC_009487	376502..376738	hypothetical protein
nr5930	NC_009487	378574..379068	hypothetical protein
nr5931	NC_009487	379061..380269	phage terminase, large subunit, PBSX family
nr5932	NC_009487	384176..384502	Rho termination factor domain protein
nr5933	NC_009487	384502..384816	hypothetical protein
nr5934	NC_009487	384809..385144	phage head-tail adaptor, putative
nr5935	NC_009487	385131..385544	phage protein, HK97 gp10 family
nr5936	NC_009487	385981..386541	hypothetical protein
nr5938	NC_009487	390446..391381	hypothetical protein
nr5939	NC_009487	391392..393278	phage minor structural protein
nr5942	NC_009487	397012..397389	hypothetical protein
nr5943	NC_009487	397390..397566	phage uncharacterized protein, XkdX family
nr5944	NC_009487	397607..397906	hypothetical protein
nr5945	NC_009487	398043..399917	Mannosyl-glycoprotein endo-beta-N-acetylglucosaminidase
nr5946	NC_009487	399930..401102	hypothetical protein
nr5953	NC_009487	927045..927347	hypothetical protein
nr5957	NC_009487	934993..935199	protein of unknown function DUF1381
nr5958	NC_009487	935809..935985	transcriptional activator RinB
nr5961	NC_009487	960439..960843	hypothetical protein

NRID	Genome ID	Coordinate in Reference Genome	Annotation
nr5964	NC_009487	1079231..1079692	protein of unknown function DUF955
nr5965	NC_009487	1079705..1080019	transcriptional regulator, XRE family
nr5966	NC_009487	1080171..1080407	hypothetical protein
nr5976	NC_009487	2165233..2165982	phage antirepressor protein
nr5983	NC_009632	48587..49249	Methyltransferase type 12
nr5984	NC_009632	49745..50476	rRNA (adenine-N(6)-)-methyltransferase
nr5985	NC_009632	50602..51384	3'-adenylyltransferase
nr5988	NC_009632	53808..54893	phage integrase family protein
nr5989	NC_009632	363362..363823	protein of unknown function DUF955
nr5991	NC_009632	365427..365612	helix-turn-helix domain protein
nr5992	NC_009632	367161..367463	hypothetical protein
nr6003	NC_009632	377184..377357	transcriptional activator RinB
nr6006	NC_009632	380293..381771	phage portal protein, SPP1 family
nr6010	NC_009632	927227..927487	protein of unknown function DUF1108
nr6030	NC_009641	324758..325240	hypothetical protein
nr6031	NC_009641	326841..327101	hypothetical protein
nr6034	NC_009641	328706..329377	hypothetical protein
nr6039	NC_009641	332953..333312	hypothetical protein
nr6043	NC_009641	334418..334867	hypothetical protein
nr6044	NC_009641	334864..335148	hypothetical protein
nr6045	NC_009641	335382..335918	hypothetical protein
nr6046	NC_009641	337331..337732	hypothetical protein
nr6055	NC_009641	359793..360965	phage tail fiber
nr6061	NC_009641	1098898..1100283	integrase
nr6064	NC_009641	1106921..1107592	hypothetical protein
nr6069	NC_009641	1111168..1111527	hypothetical protein
nr6073	NC_009641	1112633..1113082	hypothetical protein
nr6074	NC_009641	1113079..1113363	hypothetical protein
nr6075	NC_009641	1113597..1114133	hypothetical protein
nr6093	NC_009641	1134904..1135290	hypothetical protein
nr6096	NC_009641	1137855..1139027	phage tail fiber
nr6097	NC_009641	1139024..1139428	hypothetical protein
nr6104	NC_009641	1982698..1983102	hypothetical protein
nr6110	NC_009641	2009045..2009494	hypothetical protein
